# *Ex vivo* expanded human regulatory T cells promote cholesterol efflux and PON1 expression in oxLDL-exposed macrophages via gap junction-mediated cAMP transfer

**DOI:** 10.3389/fimmu.2025.1662925

**Published:** 2025-10-16

**Authors:** Caraugh Jane Albany, Daniela Mastronicola, Momchil Popov, Wladislaw Stroukov, Anthony S. Wierzbicki, Rocio Teresa Martinez-Nunez, Giovanna Lombardi, Cristiano Scottà

**Affiliations:** ^1^ ’Peter Gorer’ Department of Immunobiology, King’s College, London, United Kingdom; ^2^ National Institute for Health and Care Research (NIHR) Clinical Research Facility (CRF) Good Manufacturing Practice (GMP) Unit, Guy’s & St Thomas’ Hospitals, NHS Foundation Trust, London, United Kingdom; ^3^ Department of Metabolic Medicine/Chemical Pathology Guy’s & St Thomas’ Hospitals, London, United Kingdom; ^4^ Department of Infectious Diseases, King’s College, London, United Kingdom; ^5^ Department of Life Sciences, Centre for Inflammation Research and Translational Medicine, Brunel University, London, United Kingdom

**Keywords:** cholesterol, atherosclerosis, regulatory T (Treg) cells, macrophages, paraxonase-1

## Abstract

Lipid-driven inflammation contributes to the development of atherosclerosis, and regulatory T cells (Tregs) have been proposed to influence macrophage responses to lipid stress. While adoptive Treg transfer has been shown to be safe in clinical studies, the mechanisms by which Tregs modulate macrophage lipid handling remain incompletely understood. In this study, we investigated the effects of *ex vivo*–expanded human Tregs on primary monocyte-derived M2-like macrophages exposed to oxidized low-density lipoprotein (oxLDL) in an *in vitro* coculture system. We assessed macrophage phenotype, gene expression, and cholesterol accumulation using flow cytometry, RNA sequencing, and western blotting. Our data show that coculture with Tregs attenuated oxLDL-induced pro-inflammatory responses and reduced intracellular lipid accumulation in macrophages. Mechanistically, we found evidence that Tregs transfer cyclic AMP (cAMP) into macrophages, which enhanced the ABCA1-mediated cholesterol efflux pathway and increased expression of paraoxonase-1 (PON1). These findings provide mechanistic insight into how Tregs modulate macrophage responses to oxLDL under controlled *in vitro* conditions. They highlight potential pathways through which Tregs may regulate macrophage lipid metabolism and inflammatory activity. Further *in vivo* studies will be essential to determine the physiological significance and therapeutic potential of these mechanisms.

## Introduction

Atherosclerosis is a progressive disease and leading cause of cardiovascular disease (CVD) worldwide (1). It is characterised by slow progressing inflammation in large and medium-sized arteries where cholesterol-containing modified low-density lipoprotein (LDL) accumulates beneath the endothelial layer (2).

The earliest atherosclerotic lesion starts with the circulating monocytes transmigrating across the endothelium monolayer into intima. There they proliferate and differentiate into macrophages in response to the local inflammatory microenvironment (2). Macrophages form a hugely heterogenous population within the plaque and exhibit enormous plasticity with two prominent phenotypes that are at the ends of a spectrum: pro-inflammatory M1 macrophages and alternative activated M2 macrophages. While M1 macrophages contribute to inflammation and plaque formation, M2 macrophages are associated with tissue repair, anti-inflammatory responses, plaque resolution and stability (3). The delicate balance between pro- and anti-inflammatory macrophages is crucial for plaque stability, and an imbalance may lead to vulnerable plaque regions prone to rupture (4, 5).

Under homeostatic conditions, macrophages efflux lipids via reverse cholesterol transport (RCT) to prevent their excessive accumulation (6). However, under hyperlipidaemic conditions, this process can be overwhelmed and thus unable to balance the excessive lipid-loading (7). These events result in lipid-laden macrophages which gradually become foam cells and form plaques with necrotic cores observed in the pathogenesis of atherosclerosis (7). No M1 and M2 macrophages have been identified as specific precursors for foam cell formation, but several studies have shown that M2 macrophages are more susceptible to foam cell formation (8).

While existing treatments, such as lifestyle modifications and cholesterol-lowering medications, have shown efficacy in managing and preventing the progression of atherosclerosis, the disease remains a significant global concern. There is a growing recognition of the importance to develop new therapies that address inflammation within the arterial walls and actively stabilise existing plaques and promote their regression.

Emerging research, including the use of adoptive cell therapies such as regulatory T cells (Tregs), presents promising opportunities for addressing inflammation and immune dysregulation in atherosclerosis. During atherosclerosis, a reduction in Treg numbers and impaired functions have been observed (9–11). Studies involving adoptive transfer of Tregs in animal models have demonstrated protective effects in atherosclerosis models (1, 11).

Tregs are a subset of T lymphocytes consisting of 5-10% of the circulating CD4^+^ T cell population (12). They are characterised by high expression of CD25 and FOXP3 and low expression of CD127 molecules. Tregs modulate both innate and adaptive immune responses by suppressing inflammatory cells (12). In the last decade, our group and others investigated the manipulation of Tregs (13–16) and their use as a therapeutic tool in several studies ranging from the treatment of autoimmune disorders to preventing solid organ transplant rejection (17–23). Our strategy focused on the isolation of Tregs from the patient followed by their *ex vivo* expansion and adoptive transfer into the same individual to control inflammation and re-establish tissue homeostasis (13, 16, 18).

Previous studies, including our own, have demonstrated that Tregs can directly engage with monocytes, influencing their differentiation into macrophages (24–26). Tregs actively restrain the secretion of pro-inflammatory cytokines and inhibit the differentiation and antigen-presenting function of monocytes (26). When co-cultured with Tregs, monocytes undergo differentiation into M2-like macrophages, characterised by an increase in CD206 expression (24, 26). However, the impact of Tregs on mature human macrophages during atherosclerotic plaque development and their role in macrophage functional stability remain to be fully elucidated.

In this study, we used an *in vitro* coculture model to investigate how ex vivo–expanded, clinical-grade Tregs influence macrophage responses to oxidized low-density lipoprotein (oxLDL). We demonstrate that Tregs can modulate macrophage phenotype, attenuate oxLDL-induced inflammatory responses, and reduce intracellular lipid accumulation. Mechanistically, our data suggest that Tregs promote cholesterol efflux through gap junction–mediated transfer of cyclic AMP, leading to enhanced ABCA1 activity and increased expression of paraoxonase-1 (PON1). These findings provide new mechanistic insight into Treg–macrophage crosstalk under conditions of lipid stress and highlight pathways that could be further explored in future *in vivo* and translational studies.

## Materials and methods

### Peripheral blood mononuclear cells purification and ethical use of human samples

Human PBMC’s were isolated using Lymphocyte separation medium (LSM 1077, PAA, Somerset, UK). PBMC’s were then derived by harvesting the cell interface. All procedures performed on human participants were in accordance with the ethical standards of the Helsinki Declaration and ethically approved by HRA and Health and Care Research Wales (HCRW) with IRAS project ID 236524, REC reference 18/LO/1814. Informed consent was obtained from all individual participants involved in the study.

### T-cell isolation and Treg/Teff enrichment

To isolate CD4^+^ T cells, the RosetteSep™ Human CD4^+^ T Cell Enrichment Cocktail (STEMCELL Technologies UK Ltd, Cambridge, UK) was used as per the manufacturer’s instruction. Tregs were characterised as CD4^+^CD25^+^CD127^low^ and were enriched prior to cell sorting through positive selection with CD25 MicroBeads II (Miltenyi Biotech, Surrey, UK) following manufacturer’s instructions. Two distinct fractions were obtained: a CD25^+^ positive fraction enriched for Tregs and a CD25^-^ negative fraction enriched for conventional T cells (Teffs). Teffs were classified based on the expression of CD4^+^CD25^-^CD127^high^. Samples were then stained with mouse anti-human antibodies for CD4 (BD Pharmingen), CD127 (Biolegend) and CD25 (BioLegend) prior to cell-sorting into a highly pure population of CD4^+^CD25^+^CD127^low^ Tregs.

### Generation of Treg_exp_ cell lines

Isolated Tregs were expanded *in vitro* using the same protocol as our clinical-grade preparation, which was utilised in our previous clinical trials and publications (14, 16–18, 24, 27). Briefly, cells were cultured in X-Vivo (Lonza, UK) supplemented with 5% of Human Serum AB Male (BioWest, France) and 100 nM of rapamycin (LC-Laboratories, USA). Cells were then activated with anti-CD3/CD28 beads (ratio bead:cell of 1:1; Invitrogen, UK). IL-2 (1,000 IU/mL; Proleukin, Novartis, UK) was added at day 4 post activation and replenished every 2 days. Cells were re-stimulated every 10–12 days and used after 24 days from the first activation. Expanded cells were frozen and used when needed.

### CD14^+^ isolation and generation of M1- and M2-like macrophages

CD14^+^ monocytes were isolated from total PBMCs using CD14 MicroBeads (Miltenyi Biotech, Surrey, UK) following manufacturer’s instructions. Macrophages were generated from isolated CD14^+^ cells by culturing in RPMI 1640 (Gibco) supplemented with 10% FCS, 2mM L-glutamine (Gibco) and 1% penicillin/streptomycin (Sigma). M1-like macrophages (M_LPS_) were generated in the presence of 10ng/mL GM-CSF (R&D systems) and M2-like macrophages (M_IL4_) in the presence of 25ng/mL M-CSF (R&D Systems) using a 5-day culture period (see **Table 1**). Maturation was subsequently achieved by culturing M_LPS_ macrophages with 100ng/mL LPS (Sigma) and 20ng/mL IFNγ (R&D systems) and M_IL4_ macrophages with 20ng/mL IL-4 (R&D systems) for 48h.

**Table 1:** Macrophage types and polarising culture conditions.

Macrophage name	Differentiating molecules	Main phenotypic profile
M_LPS_	LPS, IFNγ, GM-CSF	CD14^low^, CD80^high^, CD86^high^
M_IL4_	IL4, M-CSF	CD14^high^, CD80^low^, CD86^low^

_LPS, Lipopolysaccharides; IFNγ, Interferon gamma; GM-CSF, Granulocyte-macrophage colony-stimulating factor; IL4, interleukin 4; M-CSF, Macrophage colony-stimulating factor._

### Co-culture assay

All the experiments were performed by co-culturing macrophages with autologous Tregs at a ratio of 1:1 in serum free RPMI for 24h. Where indicated macrophages were incubated for 1h with either the connexin mimetic peptide GAP27 (Cambridge Biosciences, Cat Number HY-P0139) at 300 µM or PKA inhibitor H89 at 5 µM before starting the coculture.

### oxLDL experiments

To assess the phenotypic effect of oxLDL on both M_IL4_ and M_LPS_ macrophages, the cells were cultured for 24h either alone or in the presence of 10μg/mL of oxLDL (Invitrogen, cat. Number L34357) in either the presence or absence of Tregs.

To assess uptake of both LDL and oxLDL following the 24h co-culture period, native LDL (Dil-LDL; Invitrogen, cat number L3482) or oxLDL (Dil-oxLDL; Invitrogen, cat number L34358) complexed with a fluorescent lipophilic cationic indocarbocyanine dye (1,1’-Dioctadecyl-3,3,3’,3’-Tetramethylindocarbocyanine Perchlorate fluorescent dye; Ex 554/Em 571) were added at a concentration of 10µg/mL for 6h. After this incubation, the cells were detached using StemPro^®^Accutase^®^. Uptake was analysed using flow cytometry.

To assess whether the observed effects of the co-culture experiments were contact-dependent, Transwell inserts were utilised to spatially separate cell types. Macrophages were seeded into 24-well plates whilst Tregs were separated from the macrophage monolayer using 6.5mm Transwell with 0.4µm Pore Polyester Membrane Insert, Sterile (Corning).

### Cholesterol efflux assay

To determine whether Tregs affected the reverse cholesterol transport, a cholesterol efflux assay was performed using the cholesterol efflux assay Kit from Sigma-Aldrich (Cat number MAK192) to the manufacture’s specifications. Both supernatant and cell lysate samples were read using a SpectraMax i3® (Molecular Devices) fluorescent plate reader (Excitation 485nm Emission 523nm). The rate of cholesterol efflux was calculated using C = [Fm/(Fm +Fc)] × 100% where: Fm = fluorescent intensity of supernatant Fc = fluorescent intensity of cell lysate. To test whether the observed effects of the co-culture experiments were contact-dependent, a Transwell insert was utilised to spatially separate cell types as previously described (28).

### Phagocytosis of zymosan particles

To determine whether any effects of T cells on oxLDL uptake by macrophages was specific to this molecule, a phagocytosis assay was performed. Macrophages were cultured for 24h in either in the presence or absence of T cells at a 1:1 ratio. Following this, cells were cooled to 4°C, 100 particles per macrophage of Alexa Fluor 488 conjugate Zymosan A (*S. cerevisiae*) (BioParticles, cat number Z23373) were added to the cells for 90 mins at 37°C. Cells were then washed five times with ice-cold PBS before being detached with StemPro^®^Accutase^®^. Zymosan uptake was analysed using flow cytometry.

### cAMP ELISA

Cytosolic cAMP concentrations were measured using the Complete cAMP ELISA Kit (Cat. No. ADI-900-163A, ENZO Life Sciences). Briefly, adherent macrophages and T cells from co-culture experiments were separated and washed three times with ice-cold PBS, then lysed in 0.1 M HCl containing 0.1% Triton X-100 (10^6^ cells/ml) for 10 minutes at room temperature. Lysates were centrifuged at ≥600 × g to pellet cellular debris, and the supernatants were collected for analysis. cAMP levels were normalized to total protein content according to the manufacturer’s instructions.

### RNA-sequencing

RNA-sequencing was performed at Novogene. RNA samples were subjected to polyA selection, library preparation and sequencing. Fastq files were trimmed and aligned using kallisto (29), and differential gene expression using DESeq2 (30). The RNA sequencing data has been deposited in GEO database (GSE265832) and can be accessed by token opebwewuzfidxmn. Differentially expressed genes were those that had a p-adjusted value (p-adj) of less than 0.05 (Supplemental **Supplementary Table S1**). Human genes related with cholesterol metabolism and macrophage differentiation were obtained from gene ontology data on AmiGO (release data 2024-01-17) (Supplemental **Supplementary Table S2**) (31–33). Enrichment (Supplemental **Supplementary Table S3**) was calculated employing a Fisher’s two-sided test considering 19,890 human protein coding genes as background (34). Heatmaps and volcano plot were done using R. All packages are available in CRAN.

### Reverse transcription–PCR analysis

Total RNA was extracted from cells deriving from coculture by using AllPrep DNA/RNA/Protein Mini Kit (QIAGEN, Cat Number 80004) according to the manufacturer’s instructions. RNA was reverse-transcribed to single-stranded cDNA using High-Capacity cDNA Reverse Transcription Kit (ThermoFisher, Cat Number 4368814). Quantitative RT-PCRs were performed using primers from Applied Biosystems: ATP-binding cassette A1 (*ABCA1*; Assay ID: Hs01059118_m1), ATP-binding cassette G1 (*ABCG1*; Assay ID: Hs00245154_m1), paraoxonase 1 (*PON1*; Assay ID: Hs00166557_m1) and ubiquitin C (*UBC*; Assay ID: Hs05002522_g1). Samples analysed by quantitative RT-PCR were assessed in triplicates on an Applied Biosystems cycler (ViiA7 Real-time PCR system) using the TaqMan™ Universal Master Mix II, with UNG (ThermoFisher Scientific, Catalog number: 4440038) according to manufacturer’s instructions. To quantify the data, the comparative threshold cycle method was used. Relative quantity was defined as 2^-ΔΔCt^ (35). *UBC* was used as reference gene.

### Western blotting analysis

Cells from co-culture experiments were washed 3 times in ice-cold PBS and lysed in Pierce RIPA buffer (ThermoFisher Scientific; Cat Number 89900) supplemented with Protease and Phosphatase Inhibitor Cocktail (ThermoFisher Scientific; Cat Number 78440). Protein concentration was determined by BCA assay (Pierce™, cat Number 23225). Equal amounts of total protein were separated by sodium dodecyl sulfate-polyacrylamide gel electrophoresis (SDS-PAGE), transferred to polyvinylidene difluoride (PVDF) membrane and probed overnight at 4C with the respective antibodies: monoclonal anti-ABCA1 antibody (CellSignaling; Cat Number 96292), polyclonal anti-PON1 antibody (Proteintech; Cat Number 18155-1-AP), monoclonal anti-GAPDH antibody (CellSignaling; Cat Number 5174), recombinant monoclonal anti-ABCA1 (phosho-S2054) antibody (Abcam; Cat Number ab125064). Immunocomplexes were detected using enhanced chemiluminescence (GE Healthcare). All data were analysed with Image Lab Software (BIO-RAD, Hercules, CA, USA), and GAPDH was used as an internal control.

### Statistical analysis.

Statistical analysis was carried out using GraphPad Prism 9 (GraphPad Software Inc., USA). All measures of variance were expressed as mean ± standard deviation (SD). Datasets were compared using a t-test, one- or two-way ANOVA as indicated. Data were considered statistically significant with p<0.01, p<0.001 or p<0.0001 and represented on the figures as indicated.

### Flow cytometry.

Freshly isolated Tregs, Teff and Treg_exp_ have been phenotypically evaluated by flow cytometry using antibodies listed in Supplemental **Supplementary Table S4** and following previously published procedures (16, 24). After detaching, monocytes were incubated with human TruStain FcX™ (Fc receptor blocking solution, Biolegend, USA) for 10 minutes and then stained with Fixable Viability Stain 780 (BD Biosciences, USA) and extracellular antibodies as listed in Supplemental **Supplementary Table S4** for 30 minutes at 4 °C. Samples were acquired on LSR-Fortessa™ flow cytometer and files analysed using FlowJo™ 10.8.1 (BD Life Sciences, USA). Cytokine secretion was measured employing LEGENDplex™ (BioLegend) assay as per the manufacturer’s instructions using cellular supernatant. Samples were run using a BD FACSCanto™ flow cytometer. Data analysis was performed using LEGENDplex™ Data Analysis Software V8.0.

## Results

### *Ex vivo*–expanded Tregs attenuate oxLDL-induced phenotypic changes in M_IL4_ macrophages

Regulatory T cells (Tregs) are known to play pivotal roles throughout the progression of atherosclerosis. Beyond suppressing inflammation and limiting immune-mediated damage to the arterial wall, Tregs also contribute to plaque stability and tissue repair processes (1, 36). Previous studies, including our own, have shown that Tregs can influence monocyte-to-macrophage differentiation, promoting a more tolerogenic phenotype (24, 26).

Given the impact of lipid accumulation on macrophage phenotype and function, we hypothesised that clinical-grade *ex vivo* expanded Tregs (Treg_exp_) could modulate macrophage responses and mitigate the detrimental effects of intracellular cholesterol buildup.

**Figure 1:**
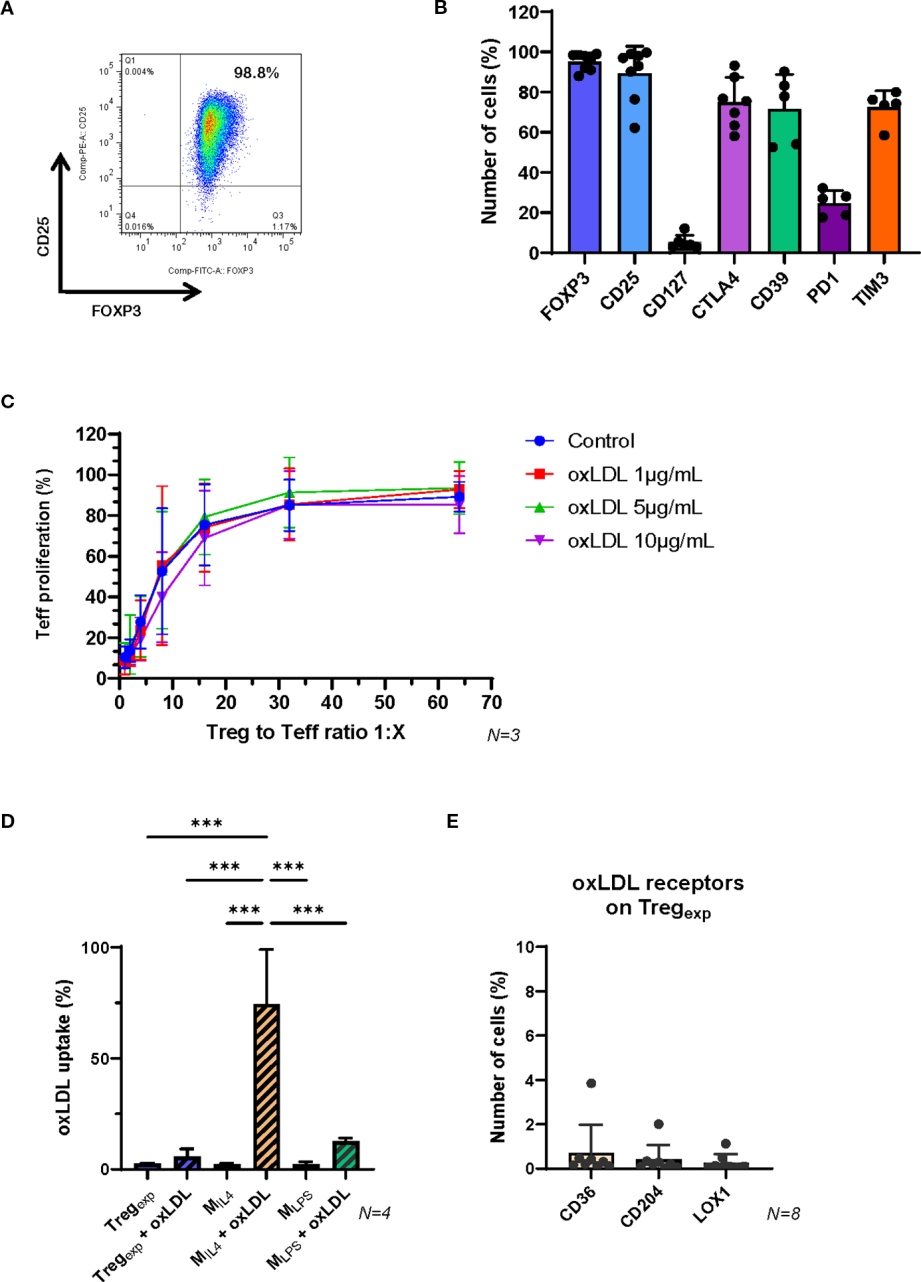
Functional properties of *ex vivo* expanded Treg_exp_ are not affected by the presence of oxLDL. **(A)** Expression of CD25 and FOXP3 in *ex vivo* expanded clinical-grade Treg_exp_ from fresh CD4^+^CD25^hi^ T cells isolated from healthy volunteers. Cells were gated on live CD4^+^ lymphocytes. The numbers in the dot plot indicate the percentage of gated cells co-expressing CD25 and FOXP3. Data are representative of >10 cellular preparations. **(B)** Cumulative data on the expression of FOXP3, CD25, CD127, CTLA4, CD39, PD-1 and TIM3 on Treg_exp_ to establish cell purity before setting the coculture with M_IL4_. Data are representative of >5 cellular preparations. **(C)** Inhibition of Teff proliferation after 5 days of coculture with Treg_exp_ at ratios 1:1, 1:2, 1:4, 1:8, 1:16, 1:32 and 1:64 (Treg_exp_: Teff) and in the presence of different concentrations of oxLDL (0, 1, 5, and 10μg/ml). **(D)** Level of Dil-oxLDL uptake in Treg_exp_, M_IL4_ and M_LPS_ after 6h exposure to 10μg/mL Dil-oxLDL. Results are expressed as percentage of fluorescent cells (Dil^+^ cells) in the total population assessed by flow cytometry. **(E)** Expression of oxLDL-specific receptors CD36, CD204, and LOX1 on Treg_exp_. Statistical analysis was performed using 1-way ANOVA. ***p<0.001.

To generate Treg_exp_, CD4^+^CD25^+^CD127^low^ T cells were isolated from the peripheral blood of healthy donors and expanded using anti-CD3/CD28 stimulation (1:1 bead-to-cell ratio), high-dose IL-2 (1,000 IU/mL), and rapamycin (100 nM), following protocols from our previous clinical trials (16–18). After two weeks, Treg_exp_ were characterised by flow cytometry, confirming high expression of CD25, FOXP3, CTLA-4, CD39, and TIM-3, along with low levels of CD127 and PD-1, hallmarks of a highly suppressive Treg phenotype (16, 37, 38) (**Figures 1A, B**).

To assess their suppressive function, Treg_exp_ were co-cultured with CFSE-labelled conventional T cells (Teff) activated with anti-CD3/CD28 beads. Flow cytometric analysis of CFSE dilution demonstrated potent suppression of Teff proliferation, consistent with our previous findings (14, 16, 24).

We next examined whether Treg_exp_ retained their suppressive capacity in an atherogenic environment. Increasing concentrations of oxLDL (1, 5, and 10 µg/mL) did not impair their ability to suppress Teff proliferation (**Figure 1C**). Supporting this, Treg_exp_ did not uptake Dil-labelled oxLDL (**Figure 1D**), likely due to the absence or very low expression of key oxLDL receptors such as CD36, CD204, and LOX-1 (**Figure 1E**).

**Figure 2:**
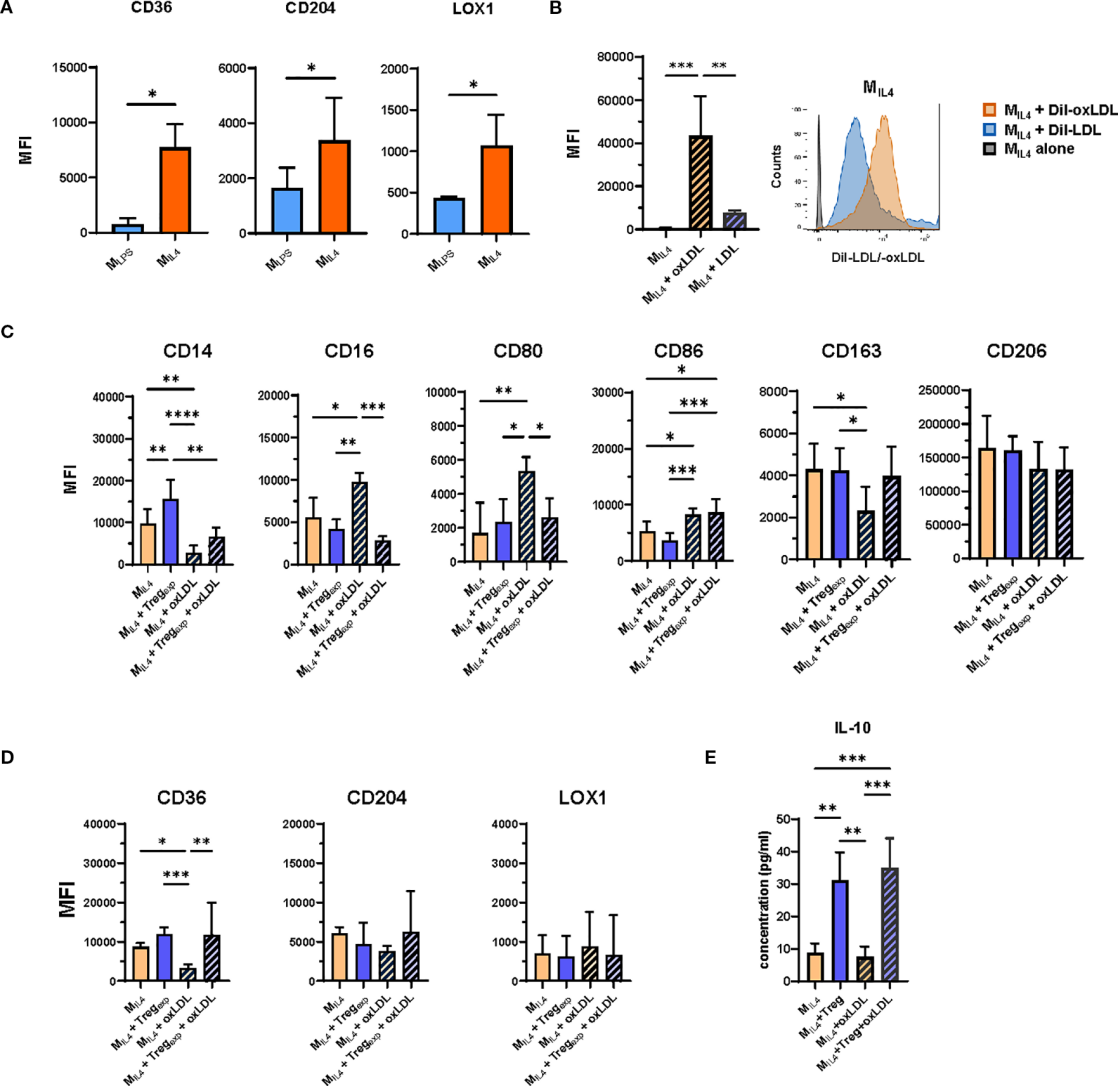
OxLDL accumulation in M_IL4_ and effect of Treg_exp_ on their repolarisation. **(A)** Changes in the expression of CD36, CD204 and LOX1 in M_LPS_ and M_IL4_ following the treatment with 10ug/mL oxLDL. Plotted data show the mean fluorescent intensity (MFI) ± SD of the markers from N = 3 independent experiments analysed by flow cytometry. **(B)** Analysis of modified (oxLDL) and non-modified (LDL) cholesterol uptake in M_IL4_. Cells were incubated for 6h with 10μg/mL of either Dil-oxLDL or Dil-LDL and analysed by flow cytometry. Plotted data show the mean fluorescent intensity (MFI) ± SD of the markers from N = 4 independent experiments analysed by flow cytometry. **(C)** Effect of Treg_exp_ on M_IL4_ (ratio 1:1) in the presence or absence of oxLDL (10μg/mL) on the expression of markers associated with the M2-like signature and **(D)** on oxLDL-specific receptors CD36, CD204, and LOX1. **(E)** Analysis of IL-10 concentration in culture supernatants of M_IL4_ in the presence or absence of oxLDL and Treg_exp_. Data from N = 7 independent experiments. Statistical analysis was performed using 1-way ANOVA. *p<0.05, **p<0.01, ***p<0.001, ****p<0.0001.

To explore the impact of Treg_exp_ on macrophage function under atherogenic conditions, we first generated macrophages using established differentiation protocols (24). Monocytes were cultured with either LPS, IFNγ, and GM-CSF or IL-4 and M-CSF (yielding M_LPS_ and M_IL4_ cells respectively; see **Table 1**; **Supplementary Figure S1**). These phenotypes differed in their surface marker expression (**Figure 2A**; **Supplementary Figure S2**). Notably, M_IL4_ cells showed higher levels of oxLDL receptors (CD36, CD204, LOX-1) and demonstrated greater oxLDL uptake compared to M_LPS_ (**Figure 1D**) or native LDL (**Figure 2B**).

Treg_exp_ were re-activated with anti-CD3/CD28 beads and cultured with IL-2 (500 IU/mL), which was necessary to sustain Treg activity but did not alter the M_IL4_ phenotype (**Supplementary Figure S3**). For the coculture experiments, we used a 1:1 Treg-to-macrophage ratio. Although this ratio is supraphysiological and does not reflect *in vivo* conditions, it was chosen in line with previous mechanistic coculture studies, where comparable proportions were required to observe measurable effects of Tregs on macrophage phenotype and function. Under these conditions, Treg_exp_ modestly increased CD14 expression on M_IL4_ in the absence of oxLDL, while CD16, CD80, CD86, CD163, CD206 and scavenger receptor expression remained largely unchanged (**Figures 2C, D**).

In the presence of oxLDL, M_IL4_ cells underwent phenotypic changes, characterized by increased expression of CD16 and CD80, and decreased levels of CD163 and CD36. Notably, M_IL4_ cells pre-treated with oxLDL were able to expand a population of T_eff_ cells that produced significantly more IFNγ compared to stimulation with untreated M_IL4_ cells (**Figure 2C**; **Supplementary Figure S2D**). Co-culture with Treg_exp_ attenuated these changes, restoring CD16 and CD80 expression toward baseline and counteracting the reduction of CD36 (**Figures 2C, D**).

To confirm the specificity of this effect, we replaced Treg_exp_ with T_eff_ in parallel co-cultures. Unlike Treg_exp_, T_eff_ promoted a shift toward a pro-inflammatory M_LPS_-like phenotype, characterised by reduced CD14 and increased CD80 and CD86 expression (**Supplementary Figure S4A**), without altering scavenger receptor levels.

Additional analyses supported a broader influence of Tregs on maintaining M2-like features. Furthermore, analysis of culture supernatants revealed the presence of IL-10 in Treg–macrophage cocultures, both with and without oxLDL, consistent with the induction of a more tolerogenic environment (**Figure 2E**). These observations are in agreement with our previous work which showed that Tregs can drive macrophages toward an IL-10–producing, alternatively activated phenotype (24).

Collectively, these findings indicate that Tregexp mitigate oxLDL-induced pro-inflammatory changes in macrophages and help maintain features of the M_IL4_ phenotype, supporting the concept that Tregs can modulate macrophage responses under conditions of lipid stress *in vitro*.

### Treg_exp_ control oxLDL accumulation by favouring its efflux from M_IL4_

To further explore the effect of Treg_exp_ on the M_IL4_ macrophages during the co-culture, their transcriptome profile was investigated.

M_IL4_ were cultured alone or in the presence of Treg_exp_ (ratio 1:1) for 24 hours. Then, medium supernatant (containing Treg_exp_ in the coculture condition) was removed and M_IL4_ cells were detached using Accutase™ and further purified by removing any T cell contaminants by fluorescence-activated cell sorting. Total RNA was then isolated from purified M_IL4_ cells and sequenced using Illumina technology.

**Figure 3:**
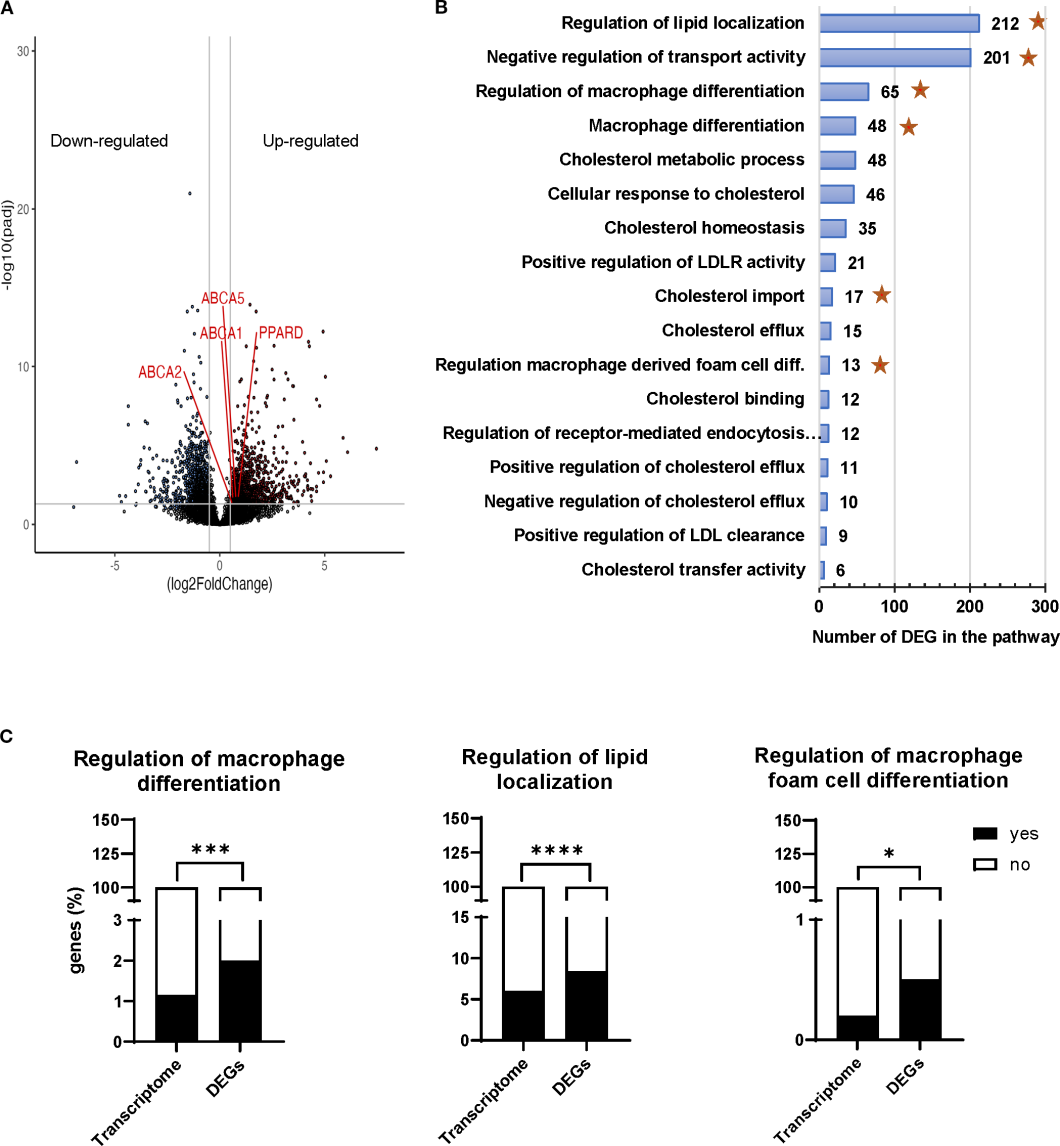
Transcriptomic profiling of M_IL4_ cocultured with Treg_exp_. **(A)** Volcano plot showing 2,395 differentially expressed genes (log2 fold change >1, p adjusted value < 0.05). **(B)** Gene ontology analysis of the biological processes affected in M_IL4_ by the presence of Treg_exp_. The x-axis shows the number of DEG associated to the biological process and the y-axis shows the Gene Ontology pathways. Stars represent the significantly enriched pathways. **(C)** representative Gene Ontology pathways significantly enriched with genes affected by the presence of Treg_exp_. Statistical analysis was performed using 2-way ANOVA and Fisher’s exact test. *p<0.05, ***p<0.001, ****p<0.0001.

The analysis of the transcripts of M_IL4_ cultured either alone or in the presence of Treg_exp_ revealed 2,395 differentially expressed genes (DEG; p-adj <0.05, which provide insights into how Treg_exp_ influenced the M_IL4_ cellular transcriptome (**Figure 3A**; **Supplementary Table S1**).

To interpret the biological significance of the observed gene expression changes in lipid-related or mediated processes, we sought for pathways present in our DEG list that related to cholesterol metabolism, localisation, and macrophage differentiation. We restricted the gene ontology analysis to the biological processes (**Supplementary Table S2**) where 10 or more of our DEGs were present (**Figure 3B**) and performed an enrichment analysis of those pathways over the expected frequency in the human transcriptome. The analysis revealed that the presence of Treg_exp_ was affecting the expression of genes in M_IL4_ cells involved in the biological processes associated not only with the macrophage differentiation but also with the localisation and transport of cholesterol (**Figure 3B**, **Supplementary Figure S5** and **Supplementary Table S3**). The Gene Ontology pathways associated to “Regulation of macrophage differentiation”, “Regulation of lipid localization”, “Regulation of macrophage foam cell differentiation” shown in **Figure 3C** along with “Negative regulation of transport activity” and “Cholesterol import” (**Supplementary Figure S6**) were all significantly enriched with genes affected by the presence of Tregs (**Figure 3C**).

**Figure 4:**
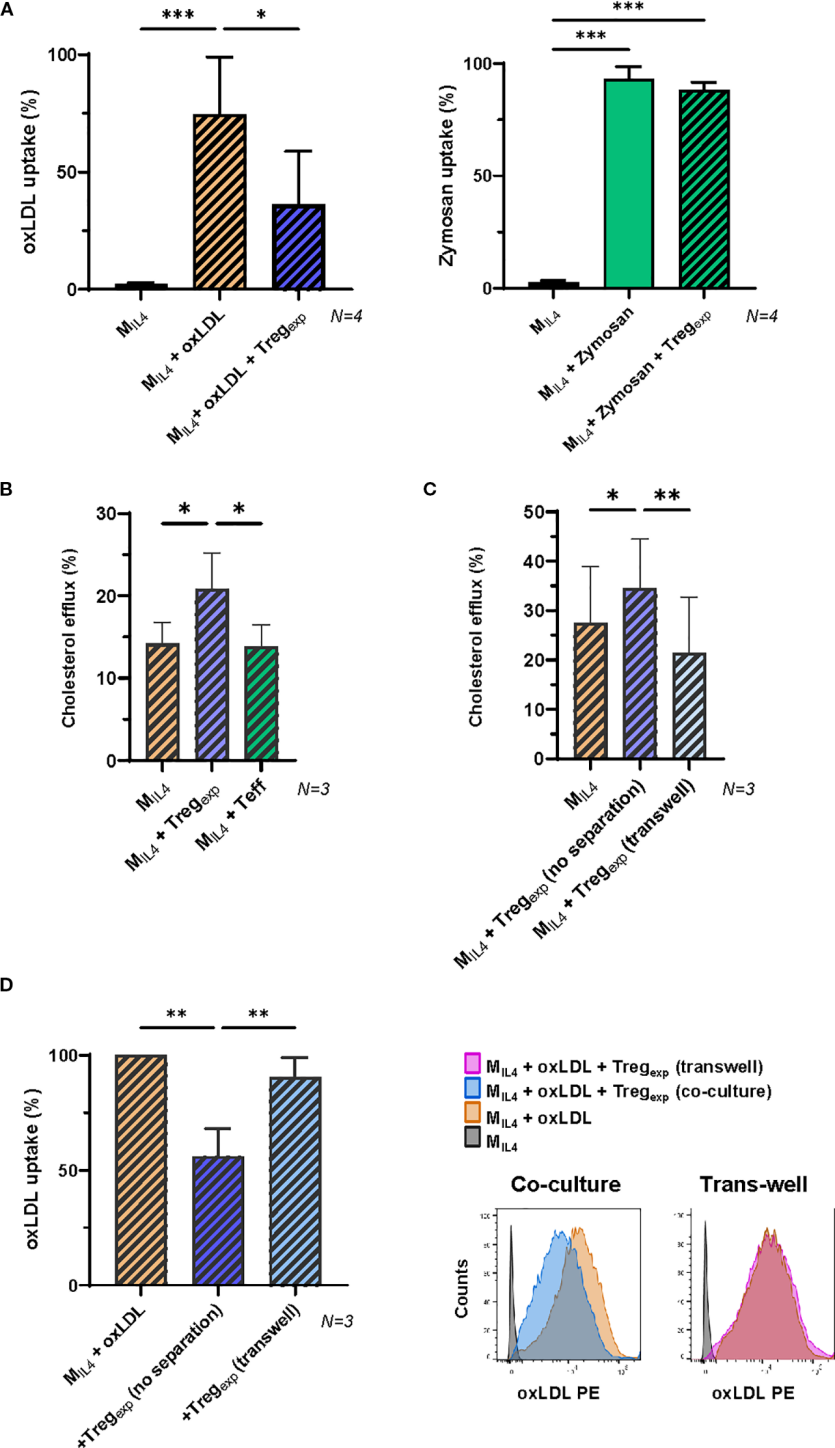
Treg_exp_ specifically affect oxLDL accumulation in M_IL4_ in a contact dependent manner. **(A)** Changes in the accumulation of fluorescent Dil-oxLDL (10μg/mL) and Zymosan (5μg/mL) in M_IL4_ co-cultured or not in the presence of Treg_exp_ (ratio 1:1) for 24h (N = 4). Results were expressed as percentage of fluorescent cells in the parent population. **(B)** Cholesterol efflux in M_IL4_ alone or co-cultured with either Treg_exp_ or Teffs. Both Treg_exp_ and Teffs were co-cultured with M_IL4_ at 1:1 ratio for 24h prior to use Cholesterol Efflux Assay Kit (Sigma-Aldrich Cat number MAK192). **(C)** Cholesterol efflux in M_IL4_ alone, co-cultured at 1:1 ratio with Treg_exp_ (no separation) or co-cultured with Treg_exp_ maintained separated in the same well by a sterile, microporous membrane to avoid cell-cell contact (transwell) for 24h. Data (N = 3) in B and C panels were normalised using the positive control provided in the Assay Kit. **(D)** Quantification of fluorescent Dil-oxLDL (10μg/mL) in M_IL4_ alone or co-cultured at 1:1 ratio with either Treg_exp_ (no separation) or Treg_exp_ (transwell) separated by a sterile microporous membrane for 24h. Data (N = 3) were plotted as percentage of fluorescent M_IL4_ (Dil-oxLDL^+^) in comparison to “M_IL4_ + oxLDL” (100%). Statistical analysis was performed with Repeated Measure One-way ANOVA followed by Tukey’s multiple comparison test to identify specific pairwise differences between conditions. *p<0.05, **p<0.01, ***p<0.001.

These findings prompted us to investigate the capacity of Treg_exp_ to regulate the increase of oxLDL within M_IL4_. We first analysed the capacity of M_IL4_ to accumulate oxLDL in the presence of Treg_exp_ by adding Dil-oxLDL to the co-culture. As shown in **Figure 4A**, M_IL4_ cultured with Treg_exp_ showed a significantly lower oxLDL accumulation than in the absence of Treg_exp_ (approximately 40% reduction, p = 0.03). Reducing the number of Treg_exp_ decreased the inhibition in oxLDL uptake (**Supplementary Figure S2C**). This was an effect specifically associated with oxLDL, as no differences were observed in the uptake of fluorescent zymosan particles in the same culture conditions (**Figure 4A**).

It has been previously published that under homeostatic conditions, macrophages remove the excess of cellular cholesterol via reverse cholesterol transport (RCT) (39). We thus investigated whether the reduced accumulation of oxLDL observed in the presence of Treg_exp_ (**Figure 4A**) could be due to increase cholesterol efflux. After 24h of co-culture with Tregs_exp_, the cholesterol efflux in M_IL4_ was significantly increased (**Figure 4B**). This effect was Treg-specific since M_IL4_ co-cultured with Teff did not show the same outcome (**Figure 4B**).

To investigate the molecular mechanisms used by Treg_exp_ to reduce cholesterol accumulation, we tested whether Tregs required close contact with the target cell or produced soluble factors to affect macrophages. To address this question Treg_exp_ and M_IL4_ were spatially separated by a porous membrane insert. The results in **Figure 4C** showed that when cell-cell contact between the two cells was prevented the increased cholesterol efflux was abolished (**Figure 4C**). Similarly, the previously observed decrease of oxLDL accumulation in M_IL4_ due to the co-culture with Treg_exp_ was abolished by the same spatial separation (**Figure 4D**).

Altogether these findings demonstrate that Treg_exp_ can decrease the accumulation of oxLDL in M_IL4_ by favouring its efflux in a cell contact manner.

### Treg_exp_ activate cAMP/PKA pathway to enhance cholesterol efflux in M_IL4_

**Figure 5:**
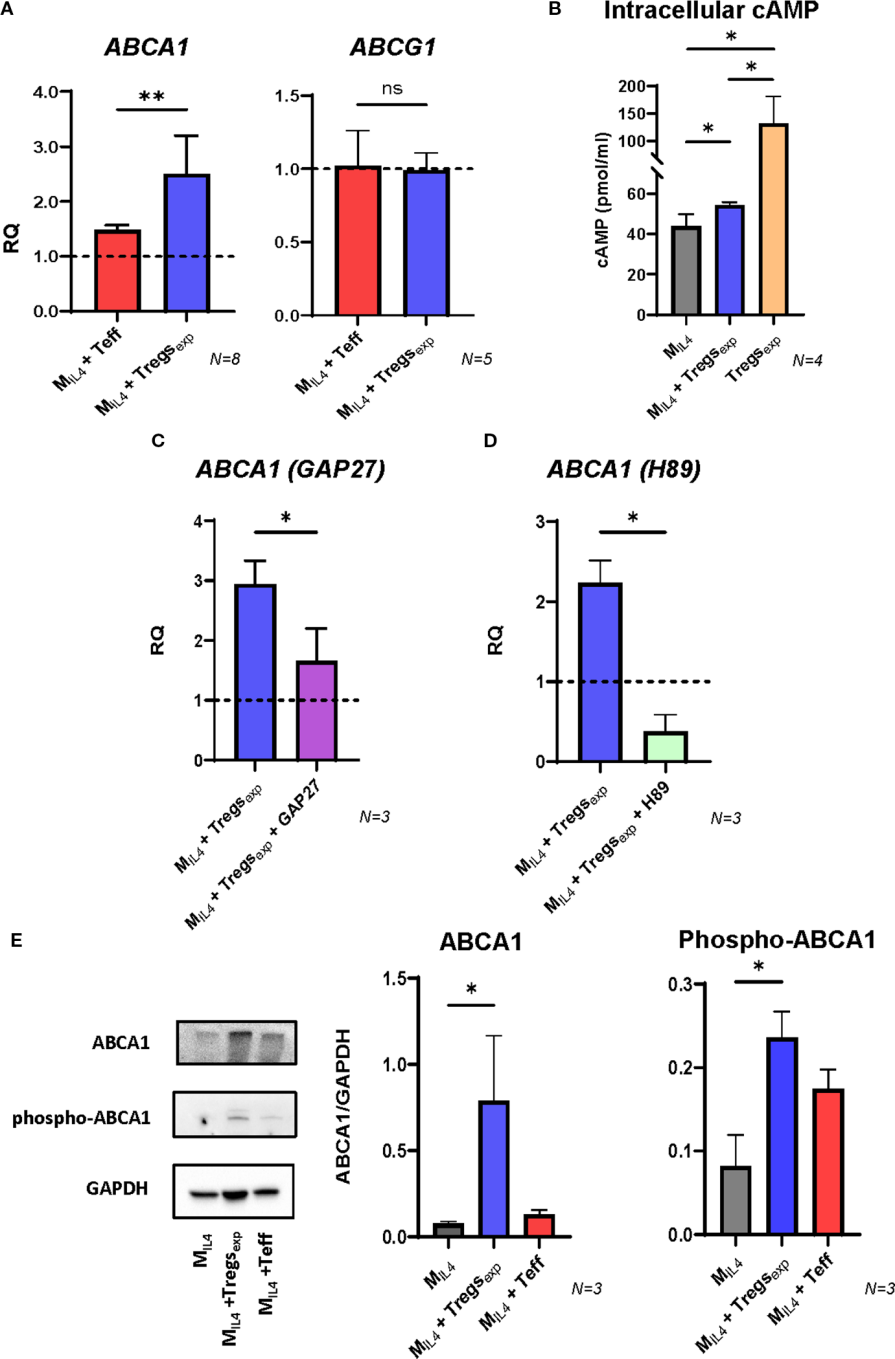
Treg_exp_ increase ABCA1 expression by transferring cAMP into M_IL4_. **(A)** Changes in the expression of *ABCA1 and ABCG1* transcripts in M_IL4_ co-cultured with either Teffs or Treg_exp_. Gene expression quantified by qRT-PCR comparing mRNA levels in M_IL4_ alone (RQ = 1, shown as dashed line) with the same cells co-cultured for 4h with either Teffs or Tregs. *UBC* was used as reference gene. **(B)** Intracellular concentration of cAMP in Tregs_exp_ alone, M_IL4_ alone, or M_IL4_ co-cultured with Tregs_exp_ for 4h. cAMP levels were normalized to total cellular protein content. Statistical analysis was performed only between M_IL4_ and M_IL4_ + Tregs_exp_, as the aim was to assess whether cAMP levels in M_IL4_ increase upon co-culture. The cAMP level in Tregs_exp_ is shown as a reference to highlight the high concentration of this molecule in these cells but was not included in the statistical comparison due to the difference in cell type. **(C)** Changes in the expression of *ABCA1* mRNA in M_IL4_ co-cultured with Treg_exp_ (ratio 1:1) after 1h preincubation or not with GAP27 (300 µM). **(D)** Changes in the expression of *ABCA1* mRNA in M_IL4_ co-cultured with Tregs (ratio 1:1) after 1h preincubation or not with PKA inhibitor H89 (5 µM). **(E)** Western blot analysis of ABCA1 protein level in cell lysates of M_IL4_ alone or M_IL4_ co-cultured (ratio 1:1) with either Treg_exp_ or Teff for 4h. Data were plotted as ABCA1 protein intensity normalised to GAPDH protein intensity. Statistical analysis was performed using one-way ANOVA followed by Tukey’s multiple comparison test. *p<0.05, **p<0.01.

To understand which molecules were involved in the cholesterol efflux enhanced by Treg_exp_, we investigated the RCT system and focused on the membrane adenosine triphosphate (ATP)-binding cassette (ABC) transporters. M_IL4_ were co-cultured with Treg_exp_, or T_effs_ as a control. After 4h, macrophages were separated from the other cells, lysed and mRNA extracted. Then, the gene expression of *ABCA1* and the other gene involved in the RCT system such as *ABCG1* was quantified by qRT-PCR. Results confirmed the RNA sequencing data of the increase of *ABCA1* gene expression in M_IL4_ co-cultured with Treg_exp_ when compared to M_IL4_ alone (2.5-fold increase) and this difference was significantly higher than the increase observed in the same cells co-cultured with T_effs_ (1.4-fold increase) (**Figure 5A**).

The production and transfer of cAMP into the target cell has been described as one of the mechanisms used by Tregs to suppress T cell proliferation (1, 12). As cAMP can stimulate *ABCA1* gene expression and enhance cholesterol efflux in human fibroblasts and THP-1 or RAW264.7 macrophages (40, 41), we investigated whether Treg_exp_ could use this mechanism to influence the same molecular pathway in M_IL4_. We analysed cAMP in Treg_exp_ alone and in M_IL4_ co-cultured or not with Treg_exp_. The results in **Figure 5B** show elevated levels of cAMP in Treg_exp_, and a noteworthy rise in intracellular cAMP in M_IL4_ when co-cultured with Treg_exp_ compared to when these cells are cultured independently. In contrast, co-culturing M_IL4_ with T_effs_ did not show any increase in cAMP (**Supplementary Figure S4B**). To determine if cAMP transfer by the Treg_exp_ to macrophages depends on gap junctions as described in the suppression of conventional CD4^+^ T cell proliferation (42), we performed the same co-culture experiment described above in the presence of the mimetic peptide GAP27 to block gap junctions. The results showed that the preincubation of M_IL4_ with GAP27 drastically inhibited the increase in *ABCA1* mRNA transcript induced by Treg_exp_ (**Figure 5C**). Likewise, preincubating M_IL4_ with the protein kinase A (PKA) inhibitor H89 led to a significant decrease in *ABCA1* transcript levels (**Figure 5D**). Together, these findings showed that *ABCA1* mRNA expression induced by cAMP transfer from Treg_exp_ to M_IL4_ is controlled by the activation of PKA pathway and depends on gap junctions.

To confirm these results at the protein level, we analysed the cell lysates of macrophages co-cultured with either Treg_exp_ or T_effs_ by western blot. The analysis of ABCA1 expression in M_IL4_ after 24h with Treg_exp_ showed a significant increase of this protein (**Figure 5E**). In contrast, the presence of T_eff_ did not produce any increase of ABCA1 (**Figure 5E**). ABCA1 activity is controlled not only at transcriptional level, but also through post-translational modifications. One of these modifications involves the phosphorylation of Ser-2054 of ABCA1 which protects the protein from its rapid degradation (43). The analysis of ABCA1 in **Figure 5E** shows that the presence of Treg_exp_ in the coculture induced the increase of ABCA1 phosphorylated in Ser-2054 in M_IL4_. These findings support that Treg_exp_ regulate cholesterol efflux by increasing both the expression and protein stability of ABCA1.

### Treg_exp_ induce paraoxonase-1 (PON1) expression in M_IL4_ macrophages

**Figure 6:**
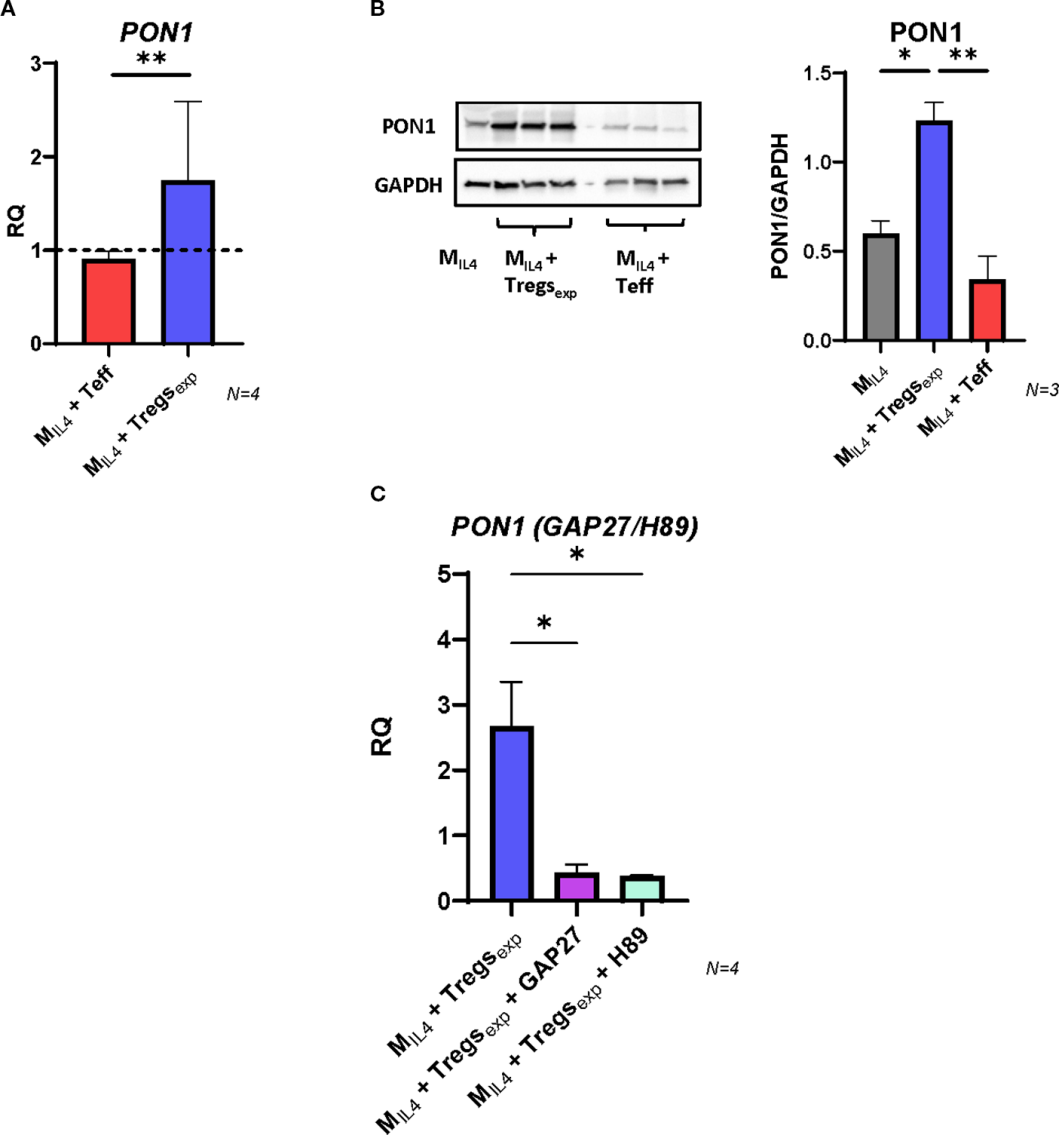
Tregs induce the expression of PON1 in M_IL4_. **(A)** Changes in the expression of *PON1* transcript in M_IL4_ co-cultured with either Teff or Treg_exp_. Gene expression quantified by qRT-PCR comparing mRNA levels in M_IL4_ alone (RQ = 1, shown as dashed line) with the same cells co-cultured for 4h with either Teff or Treg_exp_. *UBC* was used as reference gene. **(B)** Western blot analysis of PON1 protein level in cell lysates of M_IL4_ alone or M_IL4_ co-cultured (ratio 1:1) with either Treg_exp_ or Teffs for 24h. Data were plotted as PON1 protein intensity normalised to GAPDH protein intensity. **(C)** Changes in the expression of *PON1* mRNA in M_IL4_ co-cultured with Treg_exp_ (ratio 1:1) after 1h preincubation or not with either GAP27 (300 µM) or PKA inhibitor H89 (5 µM). Statistical analysis was performed using One-way ANOVA. *p<0.05, **p<0.01.

Human PON1 is a high-density lipoprotein (HDL)-associated lipolactonase which contributes to the antioxidant function of HDL (44). In animal models, Pon1 has been reported to reduce macrophage oxidative stress, prevent LDL oxidation, inhibit cholesterol synthesis, and enhance cholesterol efflux, thereby conferring atheroprotective effects (45, 46). Furthermore, human studies have shown that *PON1* gene expression and serum activity inversely correlate with cardiovascular disease risk (47, 48). To investigate whether Treg_exp_ influence PON1 expression, we analysed M_IL4_ macrophages co-cultured with Tregs or Teff cells. After 4 hours, PON1 mRNA levels were significantly higher in M_IL4_ co-cultured with Treg_exp_ compared to M_IL4_ alone (1.74-fold increase), whereas no induction was observed in the presence of Teff (**Figure 6A**). Consistently, western blot analysis after 24 hours confirmed an increase in PON1 protein levels in Treg-treated M_IL4_ (**Figure 6B**). We next explored whether the induction of PON1 followed the same cAMP-dependent mechanism identified for ABCA1 regulation. When M_IL4_ were co-cultured with Treg_exp_ in the presence of the gap junction blocker GAP27 or the PKA inhibitor H89, PON1 mRNA induction was markedly reduced (**Figure 6C**). These results indicate that Treg_exp_ promote PON1 expression in macrophages through cAMP transfer and activation of the PKA pathway.

Given the diverse functions of PON1 in modulating oxidative stress and cholesterol metabolism, these observations highlight an additional mechanism by which Treg_exp_ can influence macrophage biology *in vitro*. While further *in vivo* validation is required, the ability of Treg_exp_ to induce PON1 suggests a potential contribution to creating a more protective macrophage phenotype under lipid stress conditions.

## Discussion

This study focused on examining how *ex vivo*–expanded, clinical-grade Treg_s_ interact with differentiated macrophages under conditions of lipid stress, particularly exposure to oxLDL. Our findings indicate that Tregs can attenuate oxLDL-induced pro-inflammatory responses and reduce intracellular cholesterol accumulation in macrophages. Importantly, these results provide novel mechanistic insights into how Tregs influence macrophage phenotype and function *in vitro*.

Over the past decade, Tregs have emerged as a promising cellular therapy to restore immune homeostasis in various inflammation-driven diseases. Both our work and that of others have demonstrated the efficacy of Tregs in modulating immune responses across multiple preclinical models (13, 14, 16). Furthermore, Phase I clinical trials have confirmed the safety and potential therapeutic benefits of *in vitro* expanded Tregs in conditions such as graft-*versus*-host disease (GvHD) (20), type 1 diabetes (23), and organ transplantation (17, 18, 27).

Building on our previous work demonstrating that *ex vivo* expansion of Tregs with rapamycin yields a potent tolerogenic product (14, 16, 24, 49), we investigated whether these expanded Tregs (Treg_exp_) could influence macrophage responses to oxLDL in a controlled *in vitro* system. Our results reveal that Treg_exp_ not only dampen inflammatory responses and help preserve M2-like macrophage characteristics but also influence their cholesterol handling through genetic and functional reprogramming (**Figures 3**, **4**). Specifically, Treg_exp_ reduced oxLDL accumulation in macrophages (**Figure 4A**), suggesting a role in limiting the inflammatory stimuli associated with oxLDL exposure and its implications in the progression of diseases like atherosclerosis. Notably, this effect was selective: while oxLDL uptake was diminished, the phagocytosis of zymosan, a glucan particle, remained unaffected (**Figure 4B**), indicating that Treg_exp_ do not broadly suppress macrophage phagocytic function.

Given the central role of scavenger receptors such as CD36, CD204, and LOX-1 in oxLDL uptake, we examined whether Treg_exp_ modulate their expression. While murine studies have shown that freshly isolated Tregs can downregulate these receptors (50), our data indicate that human Treg_exp_ act through a different mechanism. In our system, expression levels of CD36, CD204, and LOX-1 remained unchanged (**Figure 2D**), suggesting that Treg_exp_ influence cholesterol metabolism through alternative pathways.

Indeed, transcriptomic analysis revealed that Treg_exp_ reprogram the gene expression profile of IL-4/M-CSF–polarised macrophages (M_IL4_), altering biological processes involved in intracellular cholesterol accumulation. These findings highlight a novel mechanism by which Tregs may contribute to plaque stabilisation beyond their classical anti-inflammatory role.

Although the mechanism might be more complex, our attempt to investigate this phenomenon revealed that Treg_exp_ regulate the level of oxLDL inside the macrophage by counterbalancing its uptake with an increased efflux (**Figures 4B, C**). We identified the transfer of cAMP through gap junctions as the mechanisms used by Treg_exp_ to boost the reverse cholesterol transport pathway and control its accumulation in these cells.

Our data showed that the increased cAMP level in macrophages during coculture with Treg_exp_ correlated with elevated transcriptional levels of *ABCA1* at the mRNA and protein levels (**Figure 5**). ABCA1 is a key molecule in reverse cholesterol transport pathway because it is responsible for the export of the excess cellular cholesterol to circulating lipid-free ApoA-I and generation of nascent HDL (51). Although ABCA1 is controlled by different cholesterol-dependent pathways, its expression at transcriptional and post-transcriptional level (phosphorylation) can be regulated by cAMP/PKA pathway as previously shown in *in vitro* studies on both human fibroblast and macrophages and *in vivo* work on murine macrophages (40, 51). However, our results contrast with other work performed on human macrophages where the authors were unable to show a significant increase in ABCA1 expression through the activation of cAMP/PKA pathway (52). It is important to note that the conflicting findings may depends on the differences in the experimental settings including type of macrophages used (e.g., freshly isolated, *in vitro* differentiated or cell lines), kinetic of the experiment and method to activate cAMP/PKA pathway.

Our findings have also indicated the increase of PON1 in macrophages as another important effect of expanded Treg_exp_ on these cells (**Figure 6**). PON1 has been described playing a protective role in several physiological contexts including cancer, ageing and inflammatory diseases (44). Human PON1 activity has been inversely correlated to the risk of CVD and in particular to the development of atherosclerosis (47, 48). PON1 activity is closely linked to its localisation on HDL particles and has been shown to prevent LDL oxidation, favour the breakdown of oxLDL, inhibit cholesterol biosynthesis and promote cholesterol efflux from macrophages (45, 46). Therefore, the upregulation of PON1 by Treg_exp_ not only works in combination with the higher expression of ABCA1 to promote the efflux of cholesterol from macrophages but can also reduce the level of oxidative stress which is further linked to the development of atherosclerotic plaque.

Interestingly, in healthy murine and human aortas, PON1 has not been identified at either the mRNA and protein level (53, 54). Previous work showed that only PON2 and PON3 isoforms are expressed in murine macrophages, whereas only PON2 is present in human macrophages (53). However, Marsillach and collaborators showed in an immunohistochemical analysis of sections of human atherosclerotic aortas that PON1 could colocalise with macrophages (55). Its presence was found to positively correlate with the extent of lesion progression. Considering the anti-inflammatory properties of PON1, the authors suggested that its presence could be a protective response to the increased oxidative stress in the aortas (55). What is not clear from these findings is whether the presence of PON1 in macrophages was the consequence of protein transfer into the cells due to the interaction with HDL or *de novo* biosynthesis in response to the increasing inflammation. Our data instead demonstrate a novel finding: Treg_exp_ can directly induce the expression of PON1 in human macrophages at both mRNA and protein levels and add a novel layer to the understanding of macrophage–Treg crosstalk.

Taken together, these findings suggest that Treg_exp_ influence both inflammatory signalling and lipid handling in macrophages, thereby providing a mechanistic framework for how Tregs may shape macrophage behaviour under lipid-rich conditions.

However, several limitations should be considered. The reliance on *ex vivo* models, while useful for controlled observations, may not fully capture the complexities of *in vivo* environments. The experiments were conducted *in vitro* using differentiated M2-like macrophages and a supraphysiological 1:1 Treg-to-macrophage ratio, which may not reflect *in vivo* conditions. The phenotypic analysis was limited to selected surface markers and IL-10, and broader cytokine or functional profiling was not performed. Furthermore, the observed effects may vary in other macrophage subsets or in the presence of additional inflammatory stimuli. Future *in vivo* studies will be essential to determine the physiological relevance of these mechanisms.

In summary, our study demonstrates that Treg_exp_ can attenuate oxLDL-induced inflammatory changes, modulate lipid handling, and promote features of an M2-like phenotype in human macrophages *in vitro*. These findings advance mechanistic understanding of Treg–macrophage interactions and highlight potential pathways, such as cAMP transfer, ABCA1 regulation, and PON1 induction that warrant further exploration in more complex models.

## Data Availability

The RNA sequencing data has been deposited in GEO database (GSE265832).
