# Supplementary material for: Ex vivo expanded human regulatory T cells promote cholesterol efflux and PON1 expression in oxLDL-exposed macrophages via gap junction-mediated cAMP transfer
Source: Front Immunol. 2025 Oct 16;16:1662925. doi: 10.3389/fimmu.2025.1662925 (PMC12571657; doi:10.3389/fimmu.2025.1662925)
Supplement: Supplementary file 1 [file DataSheet1.pdf]

Supplemental Table S1

	DIFFERENTIALLY EXPRESSED GENES OF M_{IL4}+$TREG_{EXP}$ vs M_{IL4}				
**Ensembl gene ID**	**log2 Fold Change**	**lfcSE**	**p-value**	**padj**	**HGNC symbol**
ENSG00000001629	1.61151	0.495561	0.001146	0.012188	ANKIB1
ENSG00000002330	-1.00083	0.35467	0.002996	0.024051	BAD
ENSG00000002586	-1.00083	0.249209	4.26E-06	0.000181	CD99
ENSG00000002822	-1.00083	0.408168	0.00225	0.019632	MAD1L1
ENSG00000004142	-0.50212	0.13641	0.000232	0.003843	POLDIP2
ENSG00000004399	-1.00083	0.327748	0.000643	0.007937	PLXND1
ENSG00000004961	-0.60063	0.13963	0.000017	0.000525	HCCS
ENSG00000005206	1.254032	0.24556	3.28E-07	2.53E-05	SPPL2B
ENSG00000005249	2.005077	0.386566	2.14E-07	1.83E-05	PRKAR2B
ENSG00000005483	1.209359	0.293414	3.76E-05	0.000976	KMT2E
ENSG00000005486	1.847409	0.522863	0.00041	0.005822	RHBDD2
ENSG00000006459	1.844829	0.405551	5.39E-06	0.000219	KDM7A
ENSG00000006530	0.647481	0.224888	0.003988	0.0291	AGK
ENSG00000006625	-0.8251	0.282068	0.003442	0.026523	GGCT
ENSG00000006704	-1.00083	0.204674	1.79E-07	1.59E-05	GTF2IRD1
ENSG00000007202	0.641732	0.209103	0.002148	0.019013	KIAA0100
ENSG00000007264	-1.00083	0.275882	0.00015	0.002764	MATK
ENSG00000007520	-1.00083	0.460874	0.001365	0.013732	TSR3
ENSG00000008018	-1.00083	0.326694	0.001893	0.01737	PSMB1
ENSG00000008294	1.082206	0.411528	0.008545	0.049294	SPAG9
ENSG00000008382	-1.00083	0.358693	0.001633	0.015658	MPND
ENSG00000008516	2.263594	0.601993	0.00017	0.003023	MMP25
ENSG00000008517	2.994794	0.505268	3.08E-09	6.13E-07	IL32
ENSG00000008710	1.11983	0.393215	0.004401	0.031168	PKD1
ENSG00000010219	-1.00083	0.362341	0.001275	0.013088	DYRK4
ENSG00000010244	0.604265	0.165772	0.000267	0.004178	ZNF207
ENSG00000010256	-0.59759	0.139405	1.81E-05	0.000551	UQCRC1
ENSG00000010270	-0.72542	0.134422	6.79E-08	7.77E-06	STARD3NL
ENSG00000010278	-1.00083	0.226408	1.88E-07	1.65E-05	CD9
ENSG00000010292	0.954362	0.284001	0.000778	0.009093	NCAPD2
ENSG00000010322	0.799525	0.217803	0.000242	0.003928	NISCH
ENSG00000010404	0.52223	0.183069	0.004336	0.030811	IDS
ENSG00000010539	0.672142	0.23186	0.003745	0.02791	ZNF200
ENSG00000011009	-1.00083	0.269008	4.26E-05	0.001062	LYPLA2
ENSG00000011114	0.83242	0.283378	0.003309	0.025879	BTBD7
ENSG00000011258	1.043253	0.209472	6.35E-07	4.33E-05	MBTD1
ENSG00000011405	1.206149	0.417398	0.003856	0.028479	PIK3C2A
ENSG00000011454	1.115629	0.375054	0.002934	0.02382	RABGAP1
ENSG00000011600	-1.00083	0.35828	0.005213	0.035167	TYROBP
ENSG00000012211	1.156791	0.245253	2.4E-06	0.000115	PRICKLE3
ENSG00000012817	0.920948	0.306078	0.002622	0.022003	KDM5D

ENSG00000012822	0.712617	0.259426	0.006016	0.038759	CALCOCO1
ENSG00000013275	-0.97607	0.237082	3.84E-05	0.000992	PSMC4
ENSG00000013374	1.74896	0.224545	6.76E-15	6.63E-12	NUB1
ENSG00000013583	-1.00083	0.432285	0.000324	0.004826	HEBP1
ENSG00000013725	1.924863	0.525322	0.000248	0.003998	CD6
ENSG00000014257	1.638923	0.530436	0.002003	0.018081	ACP3
ENSG00000015133	1.717791	0.639535	0.007231	0.043987	CCDC88C
ENSG00000016391	3.021206	0.80317	0.000169	0.003013	CHDH
ENSG00000017260	1.164578	0.373994	0.001846	0.017047	ATP2C1
ENSG00000019169	-1.00083	0.766539	0.000939	0.010491	MARCO
ENSG00000022840	-0.75652	0.223606	0.000716	0.008574	RNF10
ENSG00000023191	-0.76567	0.248642	0.002074	0.018514	RNH1
ENSG00000023445	1.942923	0.395636	9.07E-07	5.56E-05	BIRC3
ENSG00000023516	1.154506	0.251018	4.24E-06	0.000181	AKAP11
ENSG00000023572	-0.84749	0.201814	2.68E-05	0.000753	GLRX2
ENSG00000023734	-1.00083	0.242039	1.83E-05	0.000555	STRAP
ENSG00000023902	-0.58367	0.203147	0.004064	0.029475	PLEKHO1
ENSG00000025039	-0.74085	0.160222	3.77E-06	0.000166	RRAGD
ENSG00000025434	-1.00083	0.406423	0.000626	0.007794	NR1H3
ENSG00000026025	-0.77244	0.147673	1.69E-07	1.52E-05	VIM
ENSG00000026103	1.172553	0.378581	0.001953	0.017784	FAS
ENSG00000026297	-1.00083	0.416676	0.000214	0.003617	RNASET2
ENSG00000026508	1.02924	0.144771	1.17E-12	6.57E-10	CD44
ENSG00000026950	1.391079	0.348639	6.61E-05	0.001488	BTN3A1
ENSG00000027869	2.844315	1.058614	0.007213	0.043917	SH2D2A
ENSG00000028116	0.629684	0.122458	2.72E-07	2.23E-05	VRK2
ENSG00000028137	-0.5598	0.190505	0.003298	0.025845	TNFRSF1B
ENSG00000029153	1.868123	0.527625	0.000399	0.005694	ARNTL2
ENSG00000030066	1.76594	0.603546	0.003434	0.026487	NUP160
ENSG00000030419	3.38603	1.120029	0.002501	0.021251	IKZF2
ENSG00000030582	-0.81959	0.297052	0.005796	0.03766	GRN
ENSG00000031698	-0.78021	0.241392	0.001229	0.012819	SARS1
ENSG00000032219	1.453138	0.453509	0.001354	0.013657	ARID4A
ENSG00000033327	0.566407	0.17786	0.00145	0.014368	GAB2
ENSG00000035141	-0.69649	0.192351	0.000294	0.004484	FAM136A
ENSG00000035862	-0.7561	0.206535	0.000251	0.004022	TIMP2
ENSG00000037241	-0.80372	0.274597	0.003423	0.026434	RPL26L1
ENSG00000038274	0.65863	0.197977	0.000879	0.009919	MAT2B
ENSG00000041357	-0.68966	0.221536	0.001851	0.017081	PSMA4
ENSG00000041880	0.711835	0.259781	0.006141	0.039331	PARP3
ENSG00000041988	-1.00083	0.396084	0.008191	0.047894	THAP3
ENSG00000042286	-1.00083	0.231209	1.22E-06	7.05E-05	AIFM2
ENSG00000042317	-1.00083	0.394721	0.002978	0.023988	SPATA7
ENSG00000042493	-1.00083	0.298964	1.56E-06	8.41E-05	CAPG
ENSG00000042753	-1.00083	0.428923	0.000164	0.002936	AP2S1
ENSG00000043143	1.090332	0.333602	0.001082	0.011685	JADE2

ENSG00000044574	-1.00083	0.218631	1.12E-14	1.02E-11	HSPA5
ENSG00000047249	-1.00083	0.321579	0.001333	0.013511	ATP6V1H
ENSG00000047365	1.873493	0.456952	4.13E-05	0.001039	ARAP2
ENSG00000048028	1.01244	0.371626	0.006443	0.040556	USP28
ENSG00000048392	1.301692	0.476626	0.006313	0.040157	RRM2B
ENSG00000048649	1.539892	0.535473	0.004031	0.0293	RSF1
ENSG00000048740	0.548714	0.197951	0.005572	0.036741	CELF2
ENSG00000049239	1.640819	0.376519	1.31E-05	0.000443	H6PD
ENSG00000049245	-0.53448	0.194474	0.00599	0.038609	VAMP3
ENSG00000049249	2.089979	0.568934	0.000239	0.003915	TNFRSF9
ENSG00000049656	-0.6247	0.212912	0.003345	0.026006	CLPTM1L
ENSG00000049860	-1.00083	0.411973	4.09E-06	0.000177	HEXB
ENSG00000050344	1.091162	0.385998	0.004701	0.032598	NFE2L3
ENSG00000051620	-1.00083	0.551168	0.00604	0.038859	HEBP2
ENSG00000052802	-0.98371	0.336687	0.003481	0.026667	MSMO1
ENSG00000052841	0.593084	0.176655	0.000787	0.009162	TTC17
ENSG00000053254	1.227191	0.3266	0.000172	0.003041	FOXN3
ENSG00000053371	-0.6423	0.126054	3.48E-07	2.64E-05	AKR7A2
ENSG00000053372	-1.00083	0.220474	1.13E-06	6.73E-05	MRTO4
ENSG00000053438	2.672745	0.839246	0.001449	0.014368	NNAT
ENSG00000053501	-1.00083	0.445314	0.00497	0.033965	USE1
ENSG00000054116	-0.60082	0.204713	0.003336	0.025966	TRAPPC3
ENSG00000054219	1.106652	0.399744	0.005633	0.036986	LY75
ENSG00000054267	1.521595	0.414865	0.000245	0.003963	ARID4B
ENSG00000054277	-0.53414	0.13219	5.33E-05	0.001242	OPN3
ENSG00000054282	1.350833	0.484485	0.0053	0.035515	SDCCAG8
ENSG00000054356	-1.00083	0.792088	0.008245	0.04807	PTPRN
ENSG00000054965	1.119694	0.207537	6.85E-08	7.77E-06	FAM168A
ENSG00000055609	1.658419	0.480852	0.000563	0.007188	KMT2C
ENSG00000055950	-0.75458	0.23161	0.001122	0.012018	MRPL43
ENSG00000057657	1.731063	0.203343	1.69E-17	3.33E-14	PRDM1
ENSG00000057663	-0.53538	0.132308	0.000052	0.00122	ATG5
ENSG00000057704	0.819381	0.305942	0.007401	0.044744	TMCC3
ENSG00000057757	-1.00083	0.409648	3.11E-07	2.46E-05	PITHD1
ENSG00000058056	1.6493	0.446409	0.00022	0.003686	USP13
ENSG00000058262	-0.77422	0.160929	1.5E-06	8.32E-05	SEC61A1
ENSG00000058729	-0.82559	0.201801	4.29E-05	0.00107	RIOK2
ENSG00000059377	-0.53877	0.202093	0.007677	0.045844	TBXAS1
ENSG00000059758	1.504594	0.552013	0.006417	0.040508	CDK17
ENSG00000059769	-0.61761	0.187871	0.001011	0.011096	DNAJC25
ENSG00000060971	-0.60708	0.20376	0.002888	0.023532	ACAA1
ENSG00000061918	2.101113	0.772549	0.006534	0.040941	GUCY1B1
ENSG00000061987	1.34344	0.319651	2.64E-05	0.000745	MON2
ENSG00000063046	0.50726	0.163507	0.00192	0.017547	EIF4B
ENSG00000063169	0.680875	0.227406	0.002753	0.022765	BICRA
ENSG00000063241	-1.00083	0.396619	0.002313	0.020062	ISOC2

ENSG00000064012	1.057433	0.175456	1.67E-09	3.65E-07	CASP8
ENSG00000064490	-1.00083	0.46725	0.000927	0.01039	RFXANK
ENSG00000064601	-0.65799	0.231943	0.004556	0.031912	CTSA
ENSG00000064932	1.210006	0.228011	1.12E-07	1.15E-05	SBNO2
ENSG00000065000	-0.65529	0.15421	2.14E-05	0.000629	AP3D1
ENSG00000065268	-1.00083	0.401833	0.002448	0.020923	WDR18
ENSG00000065413	2.120205	0.474818	0.000008	0.000297	ANKRD44
ENSG00000065518	-0.56181	0.122688	4.67E-06	0.000195	NDUFB4
ENSG00000065526	0.596513	0.212503	0.004999	0.034114	SPEN
ENSG00000065600	-0.62561	0.199099	0.001677	0.015948	PACC1
ENSG00000065665	1.177064	0.24866	2.21E-06	0.000109	SEC61A2
ENSG00000065675	2.65819	0.83447	0.001445	0.014356	PRKCQ
ENSG00000065970	0.516077	0.179424	0.004024	0.029294	FOXJ2
ENSG00000065989	0.663961	0.173812	0.000133	0.002513	PDE4A
ENSG00000066084	0.792222	0.208848	0.000149	0.002747	DIP2B
ENSG00000066654	0.950318	0.329696	0.003947	0.028943	THUMPD1
ENSG00000066739	2.186443	0.605459	0.000305	0.004617	ATG2B
ENSG00000067048	1.393816	0.37423	0.000196	0.003359	DDX3Y
ENSG00000067334	-0.92745	0.314082	0.003148	0.024881	DNTTIP2
ENSG00000068120	-1.00083	0.294111	0.000113	0.002202	COASY
ENSG00000068400	0.558003	0.145662	0.000128	0.002429	GRIPAP1
ENSG00000068438	-0.67908	0.225105	0.002555	0.021627	FTSJ1
ENSG00000068650	1.554853	0.36625	2.18E-05	0.000639	ATP11A
ENSG00000068878	-0.88322	0.220162	6.03E-05	0.001379	PSME4
ENSG00000068885	1.783847	0.478863	0.000195	0.003354	IFT80
ENSG00000069869	1.575732	0.539653	0.003501	0.026756	NEDD4
ENSG00000070010	-0.84415	0.209004	5.37E-05	0.001249	UFD1
ENSG00000070061	-0.56076	0.20636	0.00658	0.041141	ELP1
ENSG00000070081	-1.00083	0.394735	0.000889	0.010015	NUCB2
ENSG00000070190	0.8088	0.193755	2.99E-05	0.000821	DAPP1
ENSG00000070444	0.912208	0.298895	0.002274	0.019807	MNT
ENSG00000070476	0.583152	0.182923	0.001433	0.014244	ZXDC
ENSG00000070718	0.848988	0.304032	0.005231	0.035242	AP3M2
ENSG00000070961	0.890848	0.257481	0.00054	0.007007	ATP2B1
ENSG00000071189	1.030808	0.304884	0.000722	0.008623	SNX13
ENSG00000071242	-0.90372	0.256397	0.000424	0.005942	RPS6KA2
ENSG00000071246	0.805541	0.233216	0.000552	0.007103	VASH1
ENSG00000071282	2.935566	0.928447	0.001568	0.015256	LMCD1
ENSG00000071462	-1.00083	0.505249	0.004456	0.031371	BUD23
ENSG00000071553	-0.60989	0.123686	8.18E-07	5.15E-05	ATP6AP1
ENSG00000071859	-1.00083	0.342886	1.42E-05	0.000467	FAM50A
ENSG00000072042	-0.69457	0.259847	0.007517	0.045207	RDH11
ENSG00000072364	1.361546	0.492181	0.005669	0.03714	AFF4
ENSG00000072786	-0.63947	0.189443	0.000737	0.008741	STK10
ENSG00000072864	0.771354	0.271787	0.004539	0.031824	NDE1
ENSG00000073331	0.795369	0.206342	0.000116	0.002251	ALPK1

ENSG00000073756	2.30205	0.539835	0.00002	0.000597	PTGS2
ENSG00000073849	0.972128	0.31092	0.001768	0.016539	ST6GAL1
ENSG00000073861	2.437301	0.867457	0.004959	0.033915	TBX21
ENSG00000073921	0.869392	0.276757	0.001682	0.015958	PICALM
ENSG00000074071	-1.00083	0.323766	0.000998	0.010987	MRPS34
ENSG00000074410	2.165428	0.788563	0.006032	0.038824	CA12
ENSG00000074621	0.998209	0.210913	2.21E-06	0.000109	SLC24A1
ENSG00000074657	0.984421	0.242786	5.02E-05	0.001197	ZNF532
ENSG00000074800	-0.74895	0.175691	2.02E-05	0.0006	ENO1
ENSG00000074842	-1.00083	0.255012	1.14E-07	1.16E-05	MYDGF
ENSG00000074855	1.94756	0.498097	9.23E-05	0.0019	ANO8
ENSG00000075336	-0.65099	0.210861	0.00202	0.01816	TIMM21
ENSG00000075391	1.330188	0.487459	0.006356	0.040307	RASAL2
ENSG00000075415	-0.51398	0.186096	0.005747	0.037485	SLC25A3
ENSG00000075539	0.717363	0.193262	0.000206	0.003504	FRYL
ENSG00000075624	-1.00083	0.316494	0.000133	0.002513	ACTB
ENSG00000075884	-0.84147	0.280411	0.002692	0.02244	ARHGAP15
ENSG00000076108	1.573177	0.440589	0.000356	0.00519	BAZ2A
ENSG00000076513	0.796792	0.144587	3.57E-08	4.95E-06	ANKRD13A
ENSG00000076641	-1.00083	0.324778	0.002055	0.018378	PAG1
ENSG00000076662	0.84294	0.262329	0.001312	0.013386	ICAM3
ENSG00000077254	0.677323	0.186376	0.000279	0.004307	USP33
ENSG00000077420	-0.54562	0.126576	1.63E-05	0.00051	APBB1IP
ENSG00000077458	1.277958	0.326107	0.000089	0.001843	FAM76B
ENSG00000078081	2.200456	0.480953	4.76E-06	0.000198	LAMP3
ENSG00000078140	-0.90493	0.121034	7.63E-14	5.82E-11	UBE2K
ENSG00000078589	2.116748	0.758132	0.005237	0.035247	P2RY10
ENSG00000078699	0.720645	0.186229	0.000109	0.002155	CBFA2T2
ENSG00000078747	2.255958	0.748179	0.002568	0.021692	ITCH
ENSG00000078804	1.957716	0.537176	0.000268	0.004184	TP53INP2
ENSG00000078808	-0.55501	0.19421	0.004266	0.030472	SDF4
ENSG00000078902	-0.5339	0.153604	0.000509	0.006737	TOLLIP
ENSG00000079257	-0.66785	0.241603	0.005705	0.037332	LXN
ENSG00000079387	2.15248	0.614819	0.000464	0.006293	SENP1
ENSG00000079616	-0.92113	0.345819	0.00773	0.046043	KIF22
ENSG00000079785	-0.69188	0.203334	0.000667	0.008122	DDX1
ENSG00000080608	-1.00083	0.379467	0.001133	0.012101	PUM3
ENSG00000080822	0.625643	0.167365	0.000185	0.003237	CLDND1
ENSG00000080824	-0.67481	0.184074	0.000246	0.003979	HSP90AA1
ENSG00000080854	4.179519	1.157443	0.000305	0.004617	IGSF9B
ENSG00000081320	1.327024	0.362554	0.000252	0.004022	STK17B
ENSG00000082074	0.821484	0.312635	0.008599	0.049497	FYB1
ENSG00000082212	-0.51468	0.180066	0.004259	0.030455	ME2
ENSG00000082213	0.790502	0.258299	0.00221	0.01939	C5orf22
ENSG00000082258	1.29659	0.299249	1.47E-05	0.00048	CCNT2
ENSG00000082397	1.041146	0.336184	0.001955	0.017789	EPB41L3

ENSG00000082781	-0.94375	0.282129	0.000823	0.009461	ITGB5
ENSG00000082805	1.727154	0.618424	0.005225	0.035215	ERC1
ENSG00000083093	-0.74862	0.255625	0.003405	0.026381	PALB2
ENSG00000083097	1.705122	0.528161	0.001245	0.012892	DOP1A
ENSG00000083168	1.094833	0.304908	0.00033	0.0049	KAT6A
ENSG00000083457	-1.00083	0.344815	0.000183	0.00321	ITGAE
ENSG00000083642	1.154781	0.395201	0.003478	0.026667	PDS5B
ENSG00000083799	0.642495	0.197983	0.001174	0.012375	CYLD
ENSG00000083896	0.709418	0.232442	0.002273	0.019807	YTHDC1
ENSG00000084072	-0.69773	0.214746	0.001158	0.012273	PPIE
ENSG00000084093	0.826446	0.299252	0.00575	0.037485	REST
ENSG00000084207	-1.00083	0.449658	0.007357	0.044532	GSTP1
ENSG00000084463	0.956349	0.230618	3.37E-05	0.000895	WBP11
ENSG00000084623	-0.71432	0.186161	0.000125	0.002384	EIF3I
ENSG00000084676	1.15766	0.390541	0.003034	0.024248	NCOA1
ENSG00000084754	-0.51404	0.094587	5.49E-08	6.8E-06	HADHA
ENSG00000085063	-0.77529	0.216351	0.000339	0.004995	CD59
ENSG00000085788	1.120409	0.389608	0.004031	0.0293	DDHD2
ENSG00000085832	0.881291	0.174132	4.17E-07	3.01E-05	EPS15
ENSG00000086062	0.573893	0.185912	0.002022	0.018172	B4GALT1
ENSG00000086289	-0.93992	0.286406	0.001032	0.011258	EPDR1
ENSG00000086504	-0.98621	0.344033	0.004149	0.0299	MRPL28
ENSG00000086598	-0.62986	0.2058	0.00221	0.01939	TMED2
ENSG00000087008	-0.80661	0.273108	0.003143	0.024868	ACOX3
ENSG00000087086	-1.00083	0.279398	6.75E-07	0.000045	FTL
ENSG00000087191	-0.86712	0.256503	0.000723	0.008625	PSMC5
ENSG00000087206	-0.66335	0.241986	0.006121	0.039267	UIMC1
ENSG00000087274	-0.83696	0.308223	0.006619	0.041341	ADD1
ENSG00000087299	1.472635	0.543665	0.006754	0.041979	L2HGDH
ENSG00000087302	-0.87757	0.304687	0.003974	0.029064	RTRAF
ENSG00000087338	0.873971	0.228277	0.000129	0.002441	GMCL1
ENSG00000087365	-0.73992	0.207901	0.000372	0.005384	SF3B2
ENSG00000087460	-0.57555	0.128563	7.58E-06	0.000283	GNAS
ENSG00000087470	0.651416	0.227984	0.004273	0.030505	DNM1L
ENSG00000087589	1.715289	0.361574	2.1E-06	0.000106	CASS4
ENSG00000088340	2.006754	0.70696	0.004532	0.031807	FER1L4
ENSG00000088448	1.057473	0.299982	0.000423	0.005942	ANKRD10
ENSG00000088766	-0.614	0.205645	0.002829	0.023242	CRLS1
ENSG00000088986	-0.86914	0.201739	1.65E-05	0.000514	DYNLL1
ENSG00000089012	4.186734	1.133038	0.00022	0.003684	SIRPG
ENSG00000089050	1.024786	0.314746	0.00113	0.012085	RBBP9
ENSG00000089127	-0.93438	0.35145	0.007846	0.046507	OAS1
ENSG00000089220	-0.95164	0.277155	0.000596	0.007522	PEBP1
ENSG00000089248	-1.00083	0.344046	0.001404	0.014036	ERP29
ENSG00000089327	-1.00083	0.28625	1.18E-10	3.26E-08	FXYD5
ENSG00000089335	1.14235	0.331144	0.000561	0.007181	ZNF302

ENSG00000089351	0.922803	0.275455	0.000808	0.009332	GRAMD1A
ENSG00000089682	0.676084	0.240055	0.004857	0.033475	RBM41
ENSG00000090013	-1.00083	0.465257	5.21E-06	0.000212	BLVRB
ENSG00000090273	-1.00083	0.32086	0.000755	0.008904	NUDC
ENSG00000090339	1.258417	0.39006	0.001254	0.012948	ICAM1
ENSG00000090376	1.331481	0.371723	0.000341	0.005014	IRAK3
ENSG00000090382	0.666246	0.237116	0.004957	0.033915	LYZ
ENSG00000090487	-0.51534	0.165193	0.001811	0.016832	SPG21
ENSG00000090520	-1.00083	0.211787	1.79E-06	9.32E-05	DNAJB11
ENSG00000090659	1.612105	0.574354	0.005003	0.034124	CD209
ENSG00000090674	-1.00083	0.492642	0.004259	0.030455	MCOLN1
ENSG00000090857	0.914421	0.290997	0.001676	0.015948	PDPR
ENSG00000090861	-0.69178	0.218196	0.001522	0.014884	AARS1
ENSG00000090924	0.791332	0.247283	0.001374	0.013779	PLEKHG2
ENSG00000091009	1.663056	0.599117	0.005506	0.036481	RBM27
ENSG00000091136	1.75264	0.592268	0.003084	0.024519	LAMB1
ENSG00000091317	0.704044	0.244408	0.003969	0.029064	CMTM6
ENSG00000091483	-0.50969	0.184612	0.005765	0.037545	FH
ENSG00000091527	-0.62354	0.122199	3.35E-07	2.57E-05	CDV3
ENSG00000091640	-0.91592	0.290996	0.001646	0.015741	SPAG7
ENSG00000092067	-1.00083	0.678647	0.001028	0.011241	CEBPE
ENSG00000092108	-0.88519	0.196303	6.5E-06	0.000254	SCFD1
ENSG00000092421	1.053692	0.303504	0.000517	0.006793	SEMA6A
ENSG00000092531	0.678543	0.202422	0.000802	0.009275	SNAP23
ENSG00000092841	-1.00083	0.261702	2.42E-08	3.4E-06	MYL6
ENSG00000093010	-0.66485	0.245407	0.006746	0.041979	COMT
ENSG00000094975	1.072773	0.256554	0.000029	0.000801	SUCO
ENSG00000095380	-1.00083	0.293643	3.82E-06	0.000167	NANS
ENSG00000095383	-1.00083	0.310248	2.2E-06	0.000109	TBC1D2
ENSG00000095564	1.37792	0.359609	0.000127	0.002423	BTAF1
ENSG00000095787	0.672779	0.232644	0.003829	0.028326	WAC
ENSG00000095951	1.184073	0.231413	3.11E-07	2.46E-05	HIVEP1
ENSG00000095970	-1.00083	0.260875	5.42E-09	9.92E-07	TREM2
ENSG00000096060	1.377209	0.278027	7.29E-07	4.77E-05	FKBP5
ENSG00000096968	1.28677	0.338382	0.000143	0.002666	JAK2
ENSG00000097021	0.88832	0.329957	0.007098	0.043443	ACOT7
ENSG00000097033	-0.5379	0.167857	0.001353	0.013657	SH3GLB1
ENSG00000099194	-0.85159	0.312544	0.006436	0.04055	SCD
ENSG00000099377	-0.59341	0.193745	0.002192	0.019306	HSD3B7
ENSG00000099385	-0.94366	0.288132	0.001056	0.011491	BCL7C
ENSG00000099624	-1.00083	0.393777	0.000214	0.003617	ATP5F1D
ENSG00000099795	-1.00083	0.415244	0.002308	0.020042	NDUFB7
ENSG00000099797	-0.77853	0.263226	0.0031	0.02463	TECR
ENSG00000099800	-1.00083	0.388643	0.000137	0.002568	TIMM13
ENSG00000099821	-1.00083	0.431721	0.003393	0.026315	POLRMT
ENSG00000099901	-0.81118	0.306027	0.008033	0.04727	RANBP1

ENSG00000099910	0.861266	0.177961	1.3E-06	7.38E-05	KLHL22
ENSG00000099940	-0.8065	0.197846	4.57E-05	0.001123	SNAP29
ENSG00000099995	0.770944	0.210645	0.000252	0.004022	SF3A1
ENSG00000099998	4.609694	1.350291	0.000641	0.007934	GGT5
ENSG00000100014	1.53668	0.533823	0.003994	0.029121	SPECC1L
ENSG00000100029	-1.00083	0.328443	0.000438	0.006087	PES1
ENSG00000100075	-0.77886	0.27161	0.004136	0.029842	SLC25A1
ENSG00000100097	-1.00083	0.386249	2.19E-06	0.000109	LGALS1
ENSG00000100122	-1.00083	0.851249	0.002514	0.021326	CRYBB1
ENSG00000100142	-1.00083	0.291412	9.68E-05	0.001971	POLR2F
ENSG00000100201	0.738604	0.13465	4.13E-08	5.55E-06	DDX17
ENSG00000100216	-0.58219	0.166615	0.000475	0.006386	TOMM22
ENSG00000100226	0.855438	0.231643	0.000222	0.003703	GTPBP1
ENSG00000100228	-1.00083	0.632492	8.49E-05	0.001794	RAB36
ENSG00000100242	1.058704	0.289982	0.000261	0.004119	SUN2
ENSG00000100285	-0.59592	0.22509	0.00811	0.047541	NEFH
ENSG00000100292	-0.99375	0.314336	0.00157	0.015264	HMOX1
ENSG00000100300	-1.00083	0.31403	0.000726	0.008648	TSPO
ENSG00000100319	-0.99988	0.272331	0.000241	0.003922	ZMAT5
ENSG00000100330	0.961841	0.23689	0.000049	0.001178	MTMR3
ENSG00000100336	2.248114	0.843943	0.007726	0.046037	APOL4
ENSG00000100342	1.240353	0.21732	1.15E-08	1.77E-06	APOL1
ENSG00000100350	0.650109	0.193012	0.000757	0.008904	FOXRED2
ENSG00000100354	2.305929	0.64652	0.000362	0.005252	TNRC6B
ENSG00000100364	-0.99838	0.296998	0.000775	0.009079	KIAA0930
ENSG00000100368	1.779361	0.417278	2.01E-05	0.000597	CSF2RB
ENSG00000100385	4.757326	0.736952	1.08E-10	3.15E-08	IL2RB
ENSG00000100387	-1.00083	0.311054	0.001103	0.011853	RBX1
ENSG00000100393	1.11703	0.364648	0.002189	0.019295	EP300
ENSG00000100425	0.662054	0.175057	0.000156	0.002841	BRD1
ENSG00000100442	-0.75657	0.191468	7.77E-05	0.001672	FKBP3
ENSG00000100504	-0.90615	0.25121	0.00031	0.004671	PYGL
ENSG00000100519	-0.68173	0.214697	0.001497	0.01473	PSMC6
ENSG00000100523	0.772197	0.243544	0.001521	0.014884	DDHD1
ENSG00000100568	-0.94852	0.243928	0.000101	0.002033	VTI1B
ENSG00000100591	-0.56814	0.212871	0.007609	0.045558	AHSA1
ENSG00000100599	-0.55583	0.158523	0.000454	0.006231	RIN3
ENSG00000100600	-1.00083	0.409005	1.01E-06	6.08E-05	LGMN
ENSG00000100632	-0.50769	0.157271	0.001246	0.012892	ERH
ENSG00000100644	0.663082	0.208989	0.00151	0.014805	HIF1A
ENSG00000100647	1.430143	0.284518	4.99E-07	3.52E-05	SUSD6
ENSG00000100697	1.72531	0.573538	0.002628	0.022025	DICER1
ENSG00000100731	1.304279	0.279273	3.01E-06	0.000136	PCNX1
ENSG00000100764	-0.78719	0.224722	0.00046	0.006272	PSMC1
ENSG00000100784	1.901201	0.520861	0.000262	0.004127	RPS6KA5
ENSG00000100804	-0.64569	0.209397	0.002046	0.018321	PSMB5

ENSG00000100867	1.637722	0.503678	0.001148	0.012188	DHRS2
ENSG00000100897	0.582758	0.221988	0.00866	0.049727	DCAF11
ENSG00000100902	-0.96086	0.272383	0.000419	0.005905	PSMA6
ENSG00000100906	1.65517	0.463195	0.000352	0.005153	NFKBIA
ENSG00000100949	-0.87802	0.219355	6.26E-05	0.001421	RABGGTA
ENSG00000100997	-0.7764	0.248478	0.00178	0.016594	ABHD12
ENSG00000101084	-1.00083	0.122838	1.06E-17	2.9E-14	RAB5IF
ENSG00000101150	-0.94632	0.242549	9.56E-05	0.00195	TPD52L2
ENSG00000101160	-1.00083	0.247869	5.39E-05	0.00125	CTSZ
ENSG00000101161	-0.73956	0.250143	0.003111	0.024689	PRPF6
ENSG00000101182	-1.00083	0.267369	8.79E-08	9.58E-06	PSMA7
ENSG00000101189	-0.77931	0.277013	0.004904	0.033664	MRGBP
ENSG00000101236	1.242001	0.250311	6.98E-07	4.61E-05	RNF24
ENSG00000101294	-1.00083	0.230023	9.21E-09	1.52E-06	HM13
ENSG00000101335	-1.00083	0.332342	0.000666	0.008113	MYL9
ENSG00000101343	-1.00083	0.389369	0.001953	0.017784	CRNKL1
ENSG00000101361	-1.00083	0.332816	0.002199	0.019349	NOP56
ENSG00000101384	1.10436	0.3303	0.000827	0.009496	JAG1
ENSG00000101421	-0.95296	0.239356	6.85E-05	0.001525	CHMP4B
ENSG00000101439	-1.00083	0.408942	0.003559	0.027056	CST3
ENSG00000101440	-1.00083	0.970436	0.00364	0.027445	ASIP
ENSG00000101470	-1.00083	1.173434	0.000621	0.007769	TNNC2
ENSG00000101577	0.855435	0.2603	0.001015	0.01113	LPIN2
ENSG00000101596	1.108312	0.25794	1.73E-05	0.000532	SMCHD1
ENSG00000101608	-0.81913	0.232155	0.000418	0.0059	MYL12A
ENSG00000101665	1.361215	0.294227	3.72E-06	0.000165	SMAD7
ENSG00000101752	0.812352	0.246131	0.000965	0.010678	MIB1
ENSG00000101773	-1.00083	0.297964	0.000445	0.006151	RBBP8
ENSG00000101916	1.098538	0.277314	7.45E-05	0.001619	TLR8
ENSG00000101940	-1.00083	0.445733	0.008544	0.049294	WDR13
ENSG00000102007	-0.91996	0.318728	0.003897	0.028719	PLP2
ENSG00000102054	-0.77016	0.265877	0.003772	0.028019	RBBP7
ENSG00000102081	1.040704	0.297541	0.000469	0.006317	FMR1
ENSG00000102172	-1.00083	0.181903	1.7E-17	3.33E-14	SMS
ENSG00000102390	-1.00083	0.258368	2.49E-05	0.000712	PBDC1
ENSG00000102393	-0.93811	0.176782	1.12E-07	1.15E-05	GLA
ENSG00000102554	2.113628	0.797494	0.008041	0.047282	KLF5
ENSG00000102780	1.246686	0.464496	0.007276	0.04416	DGKH
ENSG00000102858	0.604237	0.146665	3.79E-05	0.000982	MGRN1
ENSG00000102898	-1.00083	0.372123	0.004771	0.032982	NUTF2
ENSG00000102908	2.092392	0.537053	9.78E-05	0.00198	NFAT5
ENSG00000102921	1.264851	0.246894	3.01E-07	2.42E-05	N4BP1
ENSG00000103005	0.672913	0.154008	1.25E-05	0.000424	USB1
ENSG00000103035	-0.53424	0.121085	1.02E-05	0.000359	PSMD7
ENSG00000103043	-1.00083	0.324966	1.22E-05	0.000417	VAC14
ENSG00000103064	1.843	0.647901	0.004447	0.031364	SLC7A6

ENSG00000103066	-1.00083	0.266403	7.49E-07	4.83E-05	PLA2G15
ENSG00000103152	-1.00083	0.283285	0.000402	0.005724	MPG
ENSG00000103222	2.150926	0.695501	0.001984	0.017967	ABCC1
ENSG00000103266	-0.99068	0.319291	0.001917	0.017537	STUB1
ENSG00000103335	-1.00083	0.360763	0.000216	0.003633	PIEZO1
ENSG00000103363	-0.93372	0.300732	0.001904	0.017448	ELOB
ENSG00000103479	1.235046	0.408814	0.002519	0.021348	RBL2
ENSG00000103495	-0.7156	0.254699	0.00496	0.033915	MAZ
ENSG00000103496	-0.89168	0.214535	3.23E-05	0.000869	STX4
ENSG00000103855	-1.00083	0.187956	4.33E-09	8.26E-07	CD276
ENSG00000103876	-1.00083	0.394913	7.87E-06	0.000293	FAH
ENSG00000104064	0.818624	0.301865	0.00669	0.04175	GABPB1
ENSG00000104093	0.726974	0.189832	0.000128	0.002434	DMXL2
ENSG00000104133	0.546815	0.200704	0.00644	0.040556	SPG11
ENSG00000104164	1.017938	0.37241	0.006269	0.039937	BLOC1S6
ENSG00000104312	0.692978	0.172876	6.11E-05	0.001393	RIPK2
ENSG00000104388	-0.71109	0.227377	0.001764	0.01652	RAB2A
ENSG00000104738	1.40789	0.358929	8.76E-05	0.001826	MCM4
ENSG00000104808	-1.00083	0.664893	0.001096	0.011815	DHDH
ENSG00000104814	2.036891	0.515095	7.67E-05	0.001656	MAP4K1
ENSG00000104853	-0.80035	0.159516	5.24E-07	3.67E-05	CLPTM1
ENSG00000104888	1.868605	0.64798	0.00393	0.028852	SLC17A7
ENSG00000104894	-0.94413	0.359881	0.008705	0.049899	CD37
ENSG00000104904	-1.00083	0.297175	0.000349	0.005108	OAZ1
ENSG00000104915	-0.70574	0.238686	0.003109	0.024686	STX10
ENSG00000104936	1.765299	0.496765	0.00038	0.005469	DMPK
ENSG00000104938	2.268387	0.784544	0.003836	0.028344	CLEC4M
ENSG00000104946	1.762014	0.387557	5.46E-06	0.000221	TBC1D17
ENSG00000104972	2.081649	0.48639	1.87E-05	0.000564	LILRB1
ENSG00000104979	-1.00083	0.281919	0.000255	0.004056	C19orf53
ENSG00000105185	-0.77594	0.293031	0.008098	0.047506	PDCD5
ENSG00000105223	-0.8115	0.308862	0.008604	0.049509	PLD3
ENSG00000105270	2.056002	0.754189	0.006409	0.040505	CLIP3
ENSG00000105323	0.902548	0.216576	3.08E-05	0.000839	HNRNPUL1
ENSG00000105329	-0.64135	0.1212	1.21E-07	1.21E-05	TGFB1
ENSG00000105364	-0.73122	0.257347	0.004492	0.031571	MRPL4
ENSG00000105379	-1.00083	0.335164	0.000757	0.008904	ETFB
ENSG00000105393	-0.72119	0.191343	0.000164	0.002936	BABAM1
ENSG00000105401	-0.60149	0.197413	0.002312	0.020062	CDC37
ENSG00000105404	-1.00083	0.406175	0.000956	0.010626	RABAC1
ENSG00000105438	-0.56499	0.15272	0.000216	0.00363	KDELR1
ENSG00000105559	2.105937	0.766723	0.00602	0.038768	PLEKHA4
ENSG00000105568	-0.74508	0.203952	0.000259	0.004092	PPP2R1A
ENSG00000105576	1.105827	0.305476	0.000295	0.004489	TNPO2
ENSG00000105619	-1.00083	0.434623	0.000127	0.002423	TFPT
ENSG00000105639	3.071795	0.464401	3.73E-11	1.22E-08	JAK3

ENSG00000105669	-0.98488	0.28289	0.000499	0.006616	COPE
ENSG00000105671	-1.00083	0.337361	0.000624	0.007785	DDX49
ENSG00000105677	-0.84571	0.261963	0.001245	0.012892	TMEM147
ENSG00000105697	-1.00083	0.479698	2.48E-06	0.000117	HAMP
ENSG00000105698	-0.76367	0.262085	0.00357	0.027097	USF2
ENSG00000105701	-0.74323	0.221267	0.000782	0.009132	FKBP8
ENSG00000105856	0.955857	0.274455	0.000496	0.006596	HBP1
ENSG00000105928	-0.78152	0.19923	8.76E-05	0.001826	GSDME
ENSG00000105967	0.629961	0.192317	0.001054	0.011478	TFEC
ENSG00000105993	0.522447	0.192649	0.00669	0.04175	DNAJB6
ENSG00000106028	-0.82214	0.290603	0.004668	0.03247	SSBP1
ENSG00000106100	0.943173	0.23424	5.66E-05	0.001308	NOD1
ENSG00000106153	-0.77596	0.200631	0.00011	0.002162	CHCHD2
ENSG00000106178	4.921468	0.603838	3.63E-16	6.23E-13	CCL24
ENSG00000106245	-1.00083	0.297414	1.18E-05	0.000404	BUD31
ENSG00000106261	1.033083	0.382703	0.006946	0.042741	ZKSCAN1
ENSG00000106263	-0.77222	0.206467	0.000184	0.00322	EIF3B
ENSG00000106346	0.889473	0.252989	0.000438	0.006087	USP42
ENSG00000106355	-1.00083	0.377791	0.005264	0.035359	LSM5
ENSG00000106392	0.670259	0.196637	0.000653	0.008018	C1GALT1
ENSG00000106400	-0.88322	0.305917	0.003888	0.028667	ZNHIT1
ENSG00000106459	0.950407	0.351341	0.006829	0.042287	NRF1
ENSG00000106479	1.400618	0.406339	0.000567	0.007221	ZNF862
ENSG00000106546	2.226684	0.34604	1.24E-10	3.33E-08	AHR
ENSG00000106588	-1.00083	0.329726	0.001909	0.017485	PSMA2
ENSG00000106591	-0.61729	0.220785	0.005176	0.035009	MRPL32
ENSG00000106605	-0.99934	0.329989	0.002459	0.021004	BLVRA
ENSG00000106615	-0.61403	0.091779	2.23E-11	8.49E-09	RHEB
ENSG00000106635	-0.5873	0.150142	9.17E-05	0.00189	BCL7B
ENSG00000106780	0.840759	0.316857	0.007968	0.046988	MEGF9
ENSG00000106803	-0.83999	0.310258	0.006782	0.042072	SEC61B
ENSG00000106804	1.557402	0.456221	0.000641	0.007934	C5
ENSG00000106853	-1.00083	0.43822	0.007601	0.045531	PTGR1
ENSG00000106952	-0.88838	0.309233	0.004068	0.029486	TNFSF8
ENSG00000107130	-1.00083	0.276797	3.15E-05	0.000852	NCS1
ENSG00000107223	-0.87101	0.310431	0.005019	0.034179	EDF1
ENSG00000107262	-0.96436	0.264034	0.00026	0.0041	BAG1
ENSG00000107263	0.701879	0.257173	0.006349	0.040296	RAPGEF1
ENSG00000107331	0.596558	0.226317	0.00839	0.048687	ABCA2
ENSG00000107485	3.296142	1.094876	0.002608	0.021913	GATA3
ENSG00000107593	-1.00083	0.729806	2.34E-09	4.86E-07	PKD2L1
ENSG00000107643	1.047523	0.328894	0.001448	0.014368	MAPK8
ENSG00000107742	3.473022	0.500333	3.88E-12	1.72E-09	SPOCK2
ENSG00000107779	1.213716	0.412339	0.003245	0.025541	BMPR1A
ENSG00000107863	0.691498	0.228747	0.002503	0.021251	ARHGAP21
ENSG00000107864	0.797873	0.288588	0.005697	0.037297	CPEB3

ENSG00000107949	-0.8539	0.227401	0.000173	0.003066	BCCIP
ENSG00000107957	0.699206	0.231103	0.002482	0.021126	SH3PXD2A
ENSG00000108010	-0.9024	0.172212	1.61E-07	1.46E-05	GLRX3
ENSG00000108064	0.923689	0.345497	0.007506	0.045177	TFAM
ENSG00000108298	-0.99873	0.378287	0.008287	0.048252	RPL19
ENSG00000108349	0.52634	0.13587	0.000107	0.002125	CASC3
ENSG00000108389	1.622393	0.560698	0.003809	0.0282	MTMR4
ENSG00000108405	-1.00083	0.273775	2.37E-05	0.000684	P2RX1
ENSG00000108443	0.917338	0.315021	0.003591	0.027187	RPS6KB1
ENSG00000108582	1.810679	0.627761	0.003922	0.028841	CPD
ENSG00000108641	-1.00083	0.397154	0.004614	0.032228	B9D1
ENSG00000108651	-0.69596	0.230028	0.002482	0.021126	UTP6
ENSG00000108654	0.693835	0.119748	6.87E-09	1.21E-06	DDX5
ENSG00000108671	-0.84827	0.299892	0.004676	0.032485	PSMD11
ENSG00000108688	-1.00083	0.57348	1.86E-09	3.99E-07	CCL7
ENSG00000108788	-0.51123	0.166241	0.002103	0.018692	MLX
ENSG00000108826	-1.00083	0.319226	6.88E-05	0.001527	MRPL27
ENSG00000108828	-0.912	0.219323	3.21E-05	0.000865	VAT1
ENSG00000108829	-0.57184	0.203451	0.004943	0.033866	LRRC59
ENSG00000108846	0.955995	0.135601	1.79E-12	9.09E-10	ABCC3
ENSG00000108854	0.984378	0.236772	3.22E-05	0.000866	SMURF2
ENSG00000108861	-0.83962	0.316369	0.007956	0.046941	DUSP3
ENSG00000108953	-0.56431	0.133618	2.41E-05	0.000694	YWHAE
ENSG00000109016	-0.78547	0.241557	0.001147	0.012188	DHRS7B
ENSG00000109046	1.168833	0.389218	0.002673	0.022323	WSB1
ENSG00000109118	0.520752	0.168359	0.001981	0.01795	PHF12
ENSG00000109184	0.719356	0.27227	0.00824	0.04807	DCUN1D4
ENSG00000109320	0.936428	0.20096	3.17E-06	0.000142	NFKB1
ENSG00000109390	-0.69867	0.264884	0.008348	0.048523	NDUFC1
ENSG00000109452	3.000143	0.676268	9.15E-06	0.000326	INPP4B
ENSG00000109519	-0.92153	0.284392	0.001194	0.012531	GRPEL1
ENSG00000109618	1.119417	0.363835	0.002093	0.018621	SEPSECS
ENSG00000109685	1.566659	0.373725	2.76E-05	0.000773	NSD2
ENSG00000109790	0.726574	0.244688	0.002984	0.024013	KLHL5
ENSG00000109920	1.222945	0.367258	0.000869	0.009856	FNBP4
ENSG00000109971	-0.99667	0.177525	1.97E-08	2.85E-06	HSPA8
ENSG00000110075	1.572039	0.515154	0.002276	0.019817	PPP6R3
ENSG00000110104	-0.67302	0.213891	0.001652	0.015773	CCDC86
ENSG00000110200	-1.00083	0.356496	0.000107	0.002119	ANAPC15
ENSG00000110318	2.871176	0.869094	0.000954	0.010618	CEP126
ENSG00000110324	0.918291	0.257104	0.000355	0.005181	IL10RA
ENSG00000110328	2.602534	0.983311	0.008128	0.047627	GALNT18
ENSG00000110442	-1.00083	0.270969	0.000048	0.001161	COMMD9
ENSG00000110448	2.1074	0.795249	0.008049	0.047308	CD5
ENSG00000110514	-0.62324	0.182549	0.00064	0.007934	MADD
ENSG00000110536	-0.81805	0.210764	0.000104	0.002082	PTPMT1

ENSG00000110583	0.784103	0.251118	0.001794	0.016706	NAA40
ENSG00000110717	-1.00083	0.270245	6.21E-05	0.001411	NDUFS8
ENSG00000110719	-0.78825	0.22251	0.000396	0.005662	TCIRG1
ENSG00000110799	-1.00083	0.735484	0.006812	0.042206	VWF
ENSG00000110844	1.735535	0.607952	0.004307	0.030645	PRPF40B
ENSG00000110888	0.874513	0.240173	0.000271	0.004225	CAPRIN2
ENSG00000110955	-0.79556	0.175065	5.51E-06	0.000222	ATP5F1B
ENSG00000111011	0.576538	0.181488	0.001489	0.01467	RSRC2
ENSG00000111186	2.903383	1.023179	0.004545	0.031854	WNT5B
ENSG00000111224	0.801692	0.245106	0.001072	0.011621	PARP11
ENSG00000111249	-1.00083	0.586128	1.33E-09	3E-07	CUX2
ENSG00000111266	1.001142	0.367404	0.006432	0.04055	DUSP16
ENSG00000111328	-0.7988	0.241148	0.000925	0.010372	CDK2AP1
ENSG00000111371	2.655249	0.590882	0.000007	0.000267	SLC38A1
ENSG00000111424	1.38684	0.432653	0.001349	0.013634	VDR
ENSG00000111596	0.870457	0.250695	0.000516	0.006789	CNOT2
ENSG00000111640	-0.94983	0.182862	2.06E-07	1.77E-05	GAPDH
ENSG00000111669	-0.62481	0.18869	0.000929	0.010398	TPI1
ENSG00000111679	-0.97952	0.298805	0.001045	0.011395	PTPN6
ENSG00000111716	-0.76699	0.227418	0.000745	0.008813	LDHB
ENSG00000111775	-0.58358	0.196085	0.002919	0.023711	COX6A1
ENSG00000111801	1.626196	0.243982	2.64E-11	9.43E-09	BTN3A3
ENSG00000111802	-1.00083	0.275312	0.000235	0.003879	TDP2
ENSG00000111816	1.295234	0.455317	0.004446	0.031364	FRK
ENSG00000111845	-1.00083	0.198552	1.19E-07	0.000012	PAK1IP1
ENSG00000111877	0.828037	0.281501	0.003266	0.025668	MCM9
ENSG00000111906	-1.00083	0.315052	8.69E-06	0.000317	HDDC2
ENSG00000112033	0.875409	0.288269	0.002391	0.020569	PPARD
ENSG00000112038	-0.96935	0.337018	0.004024	0.029294	OPRM1
ENSG00000112110	-1.00083	0.358202	0.003325	0.02592	MRPL18
ENSG00000112159	-1.00083	0.348552	0.000463	0.006293	MDN1
ENSG00000112304	-1.00083	0.315503	3.69E-07	2.75E-05	ACOT13
ENSG00000112335	-0.56071	0.103723	6.45E-08	7.57E-06	SNX3
ENSG00000112473	-1.00083	0.43827	0.001962	0.017822	SLC39A7
ENSG00000112486	3.711959	1.074288	0.00055	0.007088	CCR6
ENSG00000112514	-1.00083	0.42554	0.002785	0.022966	CUTA
ENSG00000112531	0.711588	0.239737	0.002995	0.024051	QKI
ENSG00000112561	-0.98216	0.362962	0.006811	0.042206	TFEB
ENSG00000112651	-0.6526	0.229382	0.00444	0.03136	MRPL2
ENSG00000112695	-0.77936	0.281026	0.00555	0.036651	COX7A2
ENSG00000112715	1.664746	0.343007	1.21E-06	7.03E-05	VEGFA
ENSG00000112739	0.523449	0.152928	0.00062	0.007762	PRPF4B
ENSG00000112763	0.549408	0.20412	0.007111	0.043507	BTN2A1
ENSG00000112773	-1.00083	0.349701	0.002107	0.018712	TENT5A
ENSG00000112787	0.623339	0.179403	0.000512	0.00675	FBRSL1
ENSG00000112799	-1.00083	0.508865	3.99E-05	0.001016	LY86

ENSG00000112902	-1.00083	0.784376	0.007625	0.045593	SEMA5A
ENSG00000112941	0.959081	0.227712	2.53E-05	0.000721	TENT4A
ENSG00000113108	0.928506	0.289795	0.001355	0.013657	APBB3
ENSG00000113263	1.732803	0.460861	0.00017	0.003023	ITK
ENSG00000113269	-0.8174	0.215982	0.000154	0.002822	RNF130
ENSG00000113282	0.89592	0.228343	8.72E-05	0.001823	CLINT1
ENSG00000113312	-0.91762	0.179089	2.99E-07	2.42E-05	TTC1
ENSG00000113369	1.186284	0.421639	0.0049	0.033664	ARRDC3
ENSG00000113441	0.697059	0.182263	0.000131	0.002475	LNPEP
ENSG00000113558	-0.71054	0.137736	2.49E-07	2.09E-05	SKP1
ENSG00000113580	0.81588	0.277573	0.003289	0.025804	NR3C1
ENSG00000113593	0.702537	0.160819	1.25E-05	0.000424	PPWD1
ENSG00000113719	-0.74643	0.155923	1.69E-06	8.97E-05	ERGIC1
ENSG00000113732	-1.00083	0.249603	1.47E-05	0.00048	ATP6V0E1
ENSG00000113810	1.025568	0.369143	0.005465	0.036266	SMC4
ENSG00000113838	1.626753	0.611181	0.007776	0.046202	TBCCD1
ENSG00000113845	-0.92476	0.27119	0.00065	0.007998	TIMMDC1
ENSG00000113916	0.9242	0.243109	0.000144	0.00267	BCL6
ENSG00000114062	0.892206	0.32206	0.0056	0.036802	UBE3A
ENSG00000114120	1.287631	0.40577	0.001507	0.01479	SLC25A36
ENSG00000114127	2.644392	0.469308	1.75E-08	2.59E-06	XRN1
ENSG00000114251	3.761195	0.944981	6.89E-05	0.001527	WNT5A
ENSG00000114439	1.820793	0.432133	2.51E-05	0.000718	BBX
ENSG00000114631	-1.00083	0.556106	0.006142	0.039331	PODXL2
ENSG00000114737	2.656173	0.904089	0.003304	0.025863	CISH
ENSG00000114742	1.256915	0.325462	0.000112	0.0022	WDR48
ENSG00000114745	0.632288	0.187266	0.000734	0.008721	GORASP1
ENSG00000114786	1.922514	0.589592	0.001111	0.011918	ABHD14A-ACY1
ENSG00000114796	0.949688	0.319456	0.002951	0.0239	KLHL24
ENSG00000114850	-0.82501	0.226154	0.000264	0.004151	SSR3
ENSG00000114867	-0.71352	0.134865	1.22E-07	1.21E-05	EIF4G1
ENSG00000114902	-0.55737	0.154742	0.000316	0.004729	SPCS1
ENSG00000114978	0.565667	0.211711	0.007543	0.04532	MOB1A
ENSG00000115020	1.848965	0.589343	0.001705	0.016121	PIKFYVE
ENSG00000115128	-0.77441	0.234106	0.00094	0.010491	SF3B6
ENSG00000115137	1.199227	0.334143	0.000332	0.004921	DNAJC27
ENSG00000115145	0.922218	0.323489	0.00436	0.030937	STAM2
ENSG00000115170	0.532649	0.179972	0.00308	0.024501	ACVR1
ENSG00000115194	1.556206	0.572967	0.006607	0.041285	SLC30A3
ENSG00000115216	-0.73631	0.165742	8.89E-06	0.00032	NRBP1
ENSG00000115233	-0.90418	0.192439	2.62E-06	0.000123	PSMD14
ENSG00000115241	-0.64847	0.167854	0.000112	0.002194	PPM1G
ENSG00000115271	0.716152	0.139801	3.01E-07	2.42E-05	GCA
ENSG00000115307	-0.93521	0.222412	2.61E-05	0.000739	AUP1
ENSG00000115310	-0.52338	0.095008	3.61E-08	4.96E-06	RTN4
ENSG00000115317	-0.83553	0.206229	5.09E-05	0.001207	HTRA2

ENSG00000115365	1.083481	0.373511	0.003722	0.027799	LANCL1
ENSG00000115415	2.242366	0.336525	2.68E-11	9.43E-09	STAT1
ENSG00000115419	1.161648	0.295514	8.46E-05	0.00179	GLS
ENSG00000115484	-0.96746	0.290564	0.00087	0.00986	CCT4
ENSG00000115541	-0.87894	0.282548	0.001866	0.017181	HSPE1
ENSG00000115568	0.785764	0.290041	0.006746	0.041979	ZNF142
ENSG00000115590	1.271934	0.426836	0.002883	0.023521	IL1R2
ENSG00000115594	1.923871	0.40637	2.2E-06	0.000109	IL1R1
ENSG00000115657	-1.00083	0.734843	0.003976	0.029064	ABCB6
ENSG00000115677	-1.00083	0.266636	0.000113	0.002202	HDLBP
ENSG00000115756	-1.00083	0.174508	2.12E-12	1.02E-09	HPCAL1
ENSG00000115825	1.483953	0.341758	1.41E-05	0.000467	PRKD3
ENSG00000115896	1.540547	0.316886	1.16E-06	6.86E-05	PLCL1
ENSG00000115942	0.878681	0.294208	0.002821	0.023205	ORC2
ENSG00000115970	-1.00083	0.360052	0.000407	0.005779	THADA
ENSG00000116001	1.169828	0.287693	4.78E-05	0.001159	TIA1
ENSG00000116133	-0.95097	0.352428	0.006969	0.042846	DHCR24
ENSG00000116221	-0.85072	0.169078	4.87E-07	3.44E-05	MRPL37
ENSG00000116337	1.375985	0.30148	5.02E-06	0.000206	AMPD2
ENSG00000116459	-0.65558	0.125499	1.75E-07	1.57E-05	ATP5PB
ENSG00000116514	1.999303	0.561791	0.000373	0.005384	RNF19B
ENSG00000116521	-0.89163	0.301951	0.003148	0.024881	SCAMP3
ENSG00000116586	-1.00083	0.273275	8.03E-05	0.001722	LAMTOR2
ENSG00000116649	-1.00083	0.24727	3.97E-06	0.000173	SRM
ENSG00000116663	0.947098	0.268583	0.000421	0.005928	FBXO6
ENSG00000116667	-0.974	0.247547	8.33E-05	0.001771	C1orf21
ENSG00000116675	-1.00083	0.675488	0.008529	0.049244	DNAJC6
ENSG00000116824	4.32001	0.924062	2.94E-06	0.000134	CD2
ENSG00000116871	-0.68517	0.188275	0.000273	0.004252	MAP7D1
ENSG00000116898	-1.00083	0.307945	2.19E-05	0.000639	MRPS15
ENSG00000116984	1.004653	0.271492	0.000215	0.00362	MTR
ENSG00000117000	0.696635	0.215095	0.001201	0.012573	RLF
ENSG00000117020	0.83688	0.261689	0.001384	0.013868	AKT3
ENSG00000117036	1.480386	0.440534	0.000778	0.009093	ETV3
ENSG00000117090	3.110996	0.919922	0.00072	0.008604	SLAMF1
ENSG00000117091	-1.00083	0.30427	0.000105	0.002102	CD48
ENSG00000117118	-0.58852	0.147949	6.95E-05	0.00154	SDHB
ENSG00000117228	2.272193	0.337888	1.76E-11	7.11E-09	GBP1
ENSG00000117280	0.775143	0.233362	0.000895	0.010071	RAB29
ENSG00000117308	-0.87957	0.205873	1.93E-05	0.000581	GALE
ENSG00000117395	-1.00083	0.264042	4.71E-05	0.001146	EBNA1BP2
ENSG00000117410	-0.97283	0.252799	0.000119	0.002291	ATP6V0B
ENSG00000117419	-0.77483	0.277085	0.005168	0.035003	ERI3
ENSG00000117450	-0.68544	0.248022	0.005716	0.037353	PRDX1
ENSG00000117592	-0.94933	0.293904	0.001238	0.012863	PRDX6
ENSG00000117676	-0.51665	0.194567	0.007922	0.046853	RPS6KA1

ENSG00000117691	-1.00083	0.397801	0.000094	0.001922	NENF
ENSG00000117713	1.019978	0.298628	0.000637	0.007909	ARID1A
ENSG00000117724	1.509443	0.497511	0.002413	0.020709	CENPF
ENSG00000117862	-0.52768	0.18463	0.004263	0.030467	TXNDC12
ENSG00000117899	-0.61639	0.20973	0.003293	0.025818	MESD
ENSG00000117984	-1.00083	0.35544	7.34E-07	4.77E-05	CTSD
ENSG00000118113	-1.00083	0.700087	0.000137	0.002567	MMP8
ENSG00000118363	-0.77992	0.259981	0.002701	0.022457	SPCS2
ENSG00000118482	1.395692	0.386113	0.000301	0.004566	PHF3
ENSG00000118495	0.778806	0.191124	0.000046	0.001127	PLAGL1
ENSG00000118503	2.063916	0.377415	4.54E-08	5.87E-06	TNFAIP3
ENSG00000118513	3.025459	0.74558	4.95E-05	0.001185	MYB
ENSG00000118557	-1.00083	0.136387	1.52E-25	1.05E-21	PMFBP1
ENSG00000118640	-1.00083	0.315103	8.06E-05	0.001727	VAMP8
ENSG00000118680	-1.00083	0.220293	3.38E-07	2.57E-05	MYL12B
ENSG00000118705	-0.95856	0.216488	9.52E-06	0.000338	RPN2
ENSG00000118762	1.584501	0.524942	0.002541	0.02152	PKD2
ENSG00000118900	0.984173	0.327982	0.002694	0.02244	UBN1
ENSG00000119013	-0.86482	0.262885	0.001003	0.011031	NDUFB3
ENSG00000119041	0.764594	0.272244	0.004977	0.03399	GTF3C3
ENSG00000119138	0.832311	0.309453	0.007153	0.043725	KLF9
ENSG00000119139	1.530584	0.328834	3.25E-06	0.000145	TJP2
ENSG00000119285	1.368577	0.518448	0.008297	0.048285	HEATR1
ENSG00000119408	1.165577	0.274245	2.14E-05	0.000629	NEK6
ENSG00000119421	-0.81296	0.266111	0.002251	0.019632	NDUFA8
ENSG00000119487	-0.71915	0.171784	2.83E-05	0.000789	MAPKAP1
ENSG00000119630	-1.00083	0.435985	0.008671	0.049768	PGF
ENSG00000119632	-1.00083	0.371114	0.000871	0.009864	IFI27L2
ENSG00000119638	0.976887	0.261501	0.000187	0.003254	NEK9
ENSG00000119655	-0.91739	0.301196	0.00232	0.020112	NPC2
ENSG00000119681	2.30676	0.487517	2.23E-06	0.00011	LTBP2
ENSG00000119682	1.151194	0.395063	0.003569	0.027097	AREL1
ENSG00000119685	1.316887	0.356882	0.000224	0.003741	TTLL5
ENSG00000119686	1.50212	0.238646	3.09E-10	8E-08	FLVCR2
ENSG00000119705	-1.00083	0.276912	6.58E-06	0.000255	SLIRP
ENSG00000119777	-0.51291	0.124707	3.91E-05	0.001007	TMEM214
ENSG00000119787	1.52848	0.44884	0.000661	0.008075	ATL2
ENSG00000119927	2.787536	0.897794	0.001904	0.017448	GPAM
ENSG00000120162	2.783333	0.470939	3.42E-09	6.7E-07	MOB3B
ENSG00000120262	1.957236	0.606912	0.00126	0.01296	CCDC170
ENSG00000120265	-0.68558	0.183125	0.000181	0.003186	PCMT1
ENSG00000120306	-1.00083	0.258616	1.1E-07	1.15E-05	CYSTM1
ENSG00000120437	-1.00083	0.276431	3.95E-05	0.00101	ACAT2
ENSG00000120451	0.672385	0.187843	0.000344	0.005044	SNX19
ENSG00000120457	-1.00083	0.622613	0.004302	0.030645	KCNJ5
ENSG00000120533	-0.74006	0.249679	0.003036	0.024251	ENY2

ENSG00000120690	1.03599	0.345977	0.00275	0.022757	ELF1
ENSG00000120694	-0.86208	0.150304	9.72E-09	1.57E-06	HSPH1
ENSG00000120697	-0.91761	0.215945	2.14E-05	0.000629	ALG5
ENSG00000120725	-0.83137	0.287171	0.003791	0.028121	SIL1
ENSG00000120802	1.002857	0.359762	0.005311	0.035531	TMPO
ENSG00000120833	2.152084	0.801002	0.007215	0.043917	SOCS2
ENSG00000120889	0.865531	0.246956	0.000457	0.00626	TNFRSF10B
ENSG00000120963	-0.94591	0.201868	2.79E-06	0.000129	ZNF706
ENSG00000121060	0.906815	0.317678	0.00431	0.030645	TRIM25
ENSG00000121207	1.356667	0.501095	0.006781	0.042072	LRAT
ENSG00000121410	-1.00083	0.39336	0.001295	0.013244	A1BG
ENSG00000121769	-1.00083	0.357674	0.000102	0.002051	FABP3
ENSG00000121858	2.169007	0.795686	0.006412	0.040505	TNFSF10
ENSG00000121964	0.918686	0.348902	0.008462	0.049016	GTDC1
ENSG00000122008	1.143516	0.362171	0.001592	0.015391	POLK
ENSG00000122012	-1.00083	1.029372	0.000162	0.002924	SV2C
ENSG00000122026	-1.00083	0.509249	0.003412	0.026407	RPL21
ENSG00000122033	-0.90702	0.325356	0.005307	0.035531	MTIF3
ENSG00000122034	-0.81175	0.286398	0.004592	0.032115	GTF3A
ENSG00000122068	1.226068	0.436873	0.005009	0.034128	FYTTD1
ENSG00000122122	-0.86454	0.233089	0.000208	0.003535	SASH3
ENSG00000122140	-0.5509	0.159162	0.000538	0.006989	MRPS2
ENSG00000122224	-0.86197	0.326296	0.008249	0.04807	LY9
ENSG00000122254	-1.00083	0.563534	1.65E-06	8.81E-05	HS3ST2
ENSG00000122359	-1.00083	0.288654	6.49E-05	0.001467	ANXA11
ENSG00000122515	1.007758	0.290602	0.000525	0.006854	ZMIZ2
ENSG00000122547	-1.00083	0.348796	6.34E-08	7.5E-06	EEPD1
ENSG00000122641	4.609635	0.696778	3.7E-11	1.22E-08	INHBA
ENSG00000122687	-0.94772	0.246705	0.000122	0.002348	MRM2
ENSG00000122705	-1.00083	0.310531	0.000962	0.010654	CLTA
ENSG00000122971	-0.74129	0.249595	0.002978	0.023988	ACADS
ENSG00000123066	1.156581	0.198899	6.07E-09	1.1E-06	MED13L
ENSG00000123095	1.234653	0.34736	0.000379	0.005464	BHLHE41
ENSG00000123104	1.468288	0.335663	1.22E-05	0.000416	ITPR2
ENSG00000123124	0.687862	0.231839	0.003007	0.024103	WWP1
ENSG00000123130	-0.62619	0.182878	0.000617	0.007749	ACOT9
ENSG00000123131	-1.00083	0.276166	1.56E-05	0.000499	PRDX4
ENSG00000123143	-1.00083	0.377851	0.002598	0.021884	PKN1
ENSG00000123146	0.74536	0.239035	0.00182	0.01689	ADGRE5
ENSG00000123213	-1.00083	0.368751	0.006089	0.039137	NLN
ENSG00000123219	1.289295	0.40024	0.001276	0.013094	CENPK
ENSG00000123349	-1.00083	0.387668	0.007713	0.045999	PFDN5
ENSG00000123353	-0.93466	0.268167	0.000491	0.006557	ORMDL2
ENSG00000123395	-1.00083	0.322789	0.00075	0.008863	ATG101
ENSG00000123416	-1.00083	0.247574	1.68E-05	0.000522	TUBA1B
ENSG00000123545	-1.00083	0.666489	0.002886	0.023525	NDUFAF4

ENSG00000124120	0.815995	0.244176	0.000832	0.009538	TTPAL
ENSG00000124155	-0.72343	0.199096	0.00028	0.004307	PIGT
ENSG00000124172	-1.00083	0.380446	0.000661	0.008075	ATP5F1E
ENSG00000124177	1.683158	0.481571	0.000474	0.006371	CHD6
ENSG00000124201	0.773009	0.284008	0.006493	0.040779	ZNFX1
ENSG00000124207	-0.75646	0.222486	0.000674	0.008184	CSE1L
ENSG00000124222	1.344724	0.328061	4.15E-05	0.001041	STX16
ENSG00000124226	0.748501	0.273714	0.006245	0.039827	RNF114
ENSG00000124228	-0.53763	0.170165	0.001581	0.015325	DDX27
ENSG00000124299	-0.90709	0.193613	2.8E-06	0.000129	PEPD
ENSG00000124313	0.781539	0.243529	0.001331	0.013495	IQSEC2
ENSG00000124357	-1.00083	0.407536	1.34E-05	0.000449	NAGK
ENSG00000124406	0.97409	0.367687	0.008067	0.047352	ATP8A1
ENSG00000124466	2.988196	0.631794	2.25E-06	0.00011	LYPD3
ENSG00000124508	1.873667	0.317458	3.59E-09	6.94E-07	BTN2A2
ENSG00000124541	-0.74454	0.23187	0.001323	0.01345	RRP36
ENSG00000124549	0.841594	0.194581	1.52E-05	0.000494	BTN2A3P
ENSG00000124562	-1.00083	0.220894	4.59E-07	3.26E-05	SNRPC
ENSG00000124570	-1.00083	0.38842	0.000235	0.003879	SERPINB6
ENSG00000124731	-1.00083	0.52496	0.003616	0.027309	TREM1
ENSG00000124733	-0.64781	0.232572	0.005346	0.035697	MEA1
ENSG00000124788	0.601236	0.164891	0.000266	0.004167	ATXN1
ENSG00000124795	0.600556	0.194587	0.002027	0.018197	DEK
ENSG00000125046	3.215821	1.054745	0.002297	0.01997	SSUH2
ENSG00000125347	2.584664	0.328981	3.95E-15	4.93E-12	IRF1
ENSG00000125356	-0.76803	0.289246	0.007924	0.046853	NDUFA1
ENSG00000125430	3.61251	0.77174	2.85E-06	0.000131	HS3ST3B1
ENSG00000125445	-0.91239	0.192078	2.03E-06	0.000104	MRPS7
ENSG00000125447	0.805876	0.245426	0.001025	0.011213	GGA3
ENSG00000125449	-1.00083	0.39319	0.000797	0.009231	ARMC7
ENSG00000125630	1.77909	0.588801	0.002515	0.021326	POLR1B
ENSG00000125648	2.111677	0.719072	0.003318	0.025879	SLC25A23
ENSG00000125730	-1.00083	0.445712	0.003478	0.026667	C3
ENSG00000125740	1.378463	0.429102	0.001316	0.013404	FOSB
ENSG00000125743	-1.00083	0.479155	0.001772	0.016539	SNRPD2
ENSG00000125772	1.074068	0.338078	0.001488	0.014667	GPCPD1
ENSG00000125817	-0.69069	0.261745	0.008321	0.048394	CENPB
ENSG00000125835	-0.67691	0.222874	0.002388	0.020563	SNRPB
ENSG00000125844	-0.78056	0.162688	1.6E-06	8.63E-05	RRBP1
ENSG00000125868	-0.92122	0.134034	6.28E-12	2.61E-09	DSTN
ENSG00000125877	-0.99889	0.317617	0.001661	0.015838	ITPA
ENSG00000125912	-0.85168	0.283639	0.002676	0.022334	NCLN
ENSG00000125971	-0.91061	0.293803	0.001939	0.01769	DYNLRB1
ENSG00000125977	-0.98823	0.364735	0.006739	0.041979	EIF2S2
ENSG00000125991	-0.94436	0.315039	0.002721	0.022574	ERGIC3
ENSG00000125995	-1.00083	0.404155	0.001234	0.012842	ROMO1

ENSG00000126005	-0.62864	0.215071	0.003468	0.02664	MMP24OS
ENSG00000126088	-0.78207	0.181388	1.62E-05	0.00051	UROD
ENSG00000126106	-1.00083	0.225269	5.76E-07	3.97E-05	TMEM53
ENSG00000126216	0.902598	0.304128	0.002999	0.024051	TUBGCP3
ENSG00000126247	-0.95601	0.212049	6.53E-06	0.000255	CAPNS1
ENSG00000126254	-0.90254	0.343123	0.008529	0.049244	RBM42
ENSG00000126264	-1.00083	0.31654	9.75E-07	5.95E-05	HCST
ENSG00000126267	-1.00083	0.330245	0.001021	0.011187	COX6B1
ENSG00000126432	-1.00083	0.28989	0.000144	0.00267	PRDX5
ENSG00000126698	-0.79805	0.19112	2.97E-05	0.000817	DNAJC8
ENSG00000126709	-1.00083	0.401582	0.000958	0.01064	IFI6
ENSG00000126749	-0.79121	0.299538	0.008256	0.048088	EMG1
ENSG00000126756	-1.00083	0.350191	0.001599	0.015428	UXT
ENSG00000126934	-1.00083	0.376195	0.000545	0.007046	MAP2K2
ENSG00000127152	3.759212	0.960645	9.11E-05	0.00188	BCL11B
ENSG00000127184	-0.83024	0.301382	0.005873	0.03799	COX7C
ENSG00000127314	0.586462	0.20765	0.004739	0.032808	RAP1B
ENSG00000127419	1.182324	0.442726	0.007573	0.045399	TMEM175
ENSG00000127481	-0.91749	0.277851	0.00096	0.010643	UBR4
ENSG00000127483	0.897706	0.323394	0.005505	0.036481	HP1BP3
ENSG00000127511	0.965413	0.354124	0.006407	0.040505	SIN3B
ENSG00000127527	-1.00083	0.297729	0.000675	0.008184	EPS15L1
ENSG00000127540	-1.00083	0.340636	0.000496	0.006596	UQCR11
ENSG00000127616	-0.86086	0.298374	0.003912	0.028783	SMARCA4
ENSG00000127663	0.982114	0.327024	0.002672	0.022323	KDM4B
ENSG00000127884	-0.91995	0.241836	0.000142	0.002656	ECHS1
ENSG00000127914	0.77632	0.206498	0.00017	0.003024	AKAP9
ENSG00000127922	-1.00083	0.404302	0.000919	0.01032	SEM1
ENSG00000127946	1.222541	0.409921	0.00286	0.023403	HIP1
ENSG00000127948	-1.00083	0.391675	0.00514	0.034845	POR
ENSG00000127951	2.493393	0.340107	2.28E-13	1.57E-10	FGL2
ENSG00000128039	-1.00083	0.264945	1.32E-06	7.44E-05	SRD5A3
ENSG00000128045	-1.00083	0.61926	0.003246	0.025541	RASL11B
ENSG00000128228	-1.00083	0.728724	0.000546	0.007046	SDF2L1
ENSG00000128272	-1.00083	0.27253	0.000159	0.002885	ATF4
ENSG00000128284	2.178993	0.453139	1.52E-06	8.34E-05	APOL3
ENSG00000128294	-1.00083	0.268269	2.69E-05	0.000755	TPST2
ENSG00000128463	-0.5453	0.149141	0.000256	0.004056	EMC4
ENSG00000128512	0.720298	0.162833	9.71E-06	0.000343	DOCK4
ENSG00000128524	-0.64691	0.226289	0.004253	0.03044	ATP6V1F
ENSG00000128602	2.20763	0.72424	0.002302	0.020004	SMO
ENSG00000128694	1.340461	0.375448	0.000357	0.005191	OSGEPL1
ENSG00000128815	-0.59563	0.160698	0.00021	0.003561	WDFY4
ENSG00000128829	-1.00083	0.373803	0.004896	0.033664	EIF2AK4
ENSG00000128872	2.825611	0.660286	1.87E-05	0.000564	TMOD2
ENSG00000128928	0.50621	0.148541	0.000655	0.00803	IVD

ENSG00000129007	-1.00083	0.330444	0.001609	0.015484	CALML4
ENSG00000129084	-0.73562	0.224774	0.001065	0.011579	PSMA1
ENSG00000129219	0.871978	0.292134	0.002837	0.023282	PLD2
ENSG00000129226	-1.00083	0.243534	3.96E-05	0.001011	CD68
ENSG00000129235	-1.00083	0.328142	0.00064	0.007934	TXNDC17
ENSG00000129292	0.900616	0.255014	0.000413	0.005845	PHF20L1
ENSG00000129315	0.873292	0.323399	0.006927	0.042643	CCNT1
ENSG00000129353	1.176877	0.406647	0.003802	0.028173	SLC44A2
ENSG00000129538	-1.00083	0.504665	0.008386	0.048679	RNASE1
ENSG00000129559	-1.00083	0.232588	6.62E-06	0.000256	NEDD8
ENSG00000129562	-1.00083	0.20704	6.56E-10	1.58E-07	DAD1
ENSG00000129625	-0.74919	0.210194	0.000365	0.005295	REEP5
ENSG00000129667	0.855604	0.209759	4.52E-05	0.001116	RHBDF2
ENSG00000129932	-1.00083	0.284939	0.000169	0.003013	DOHH
ENSG00000129933	1.173962	0.383958	0.002232	0.019528	MAU2
ENSG00000129968	-0.85528	0.321943	0.007893	0.046729	ABHD17A
ENSG00000130159	-0.8453	0.257403	0.001024	0.011207	ECSIT
ENSG00000130165	-0.84351	0.274106	0.002089	0.018621	ELOF1
ENSG00000130204	-1.00083	0.311105	3.91E-05	0.001007	TOMM40
ENSG00000130208	-1.00083	0.381074	0.004779	0.033021	APOC1
ENSG00000130227	0.93498	0.236189	7.54E-05	0.001635	XPO7
ENSG00000130299	1.082714	0.320494	0.000729	0.008678	GTPBP3
ENSG00000130303	-0.9899	0.30663	0.001245	0.012892	BST2
ENSG00000130304	-0.77297	0.278156	0.005454	0.036227	SLC27A1
ENSG00000130396	3.146176	0.433775	4.08E-13	2.66E-10	AFDN
ENSG00000130402	0.561488	0.174054	0.001256	0.012951	ACTN4
ENSG00000130511	-0.7431	0.247947	0.002726	0.022603	SSBP4
ENSG00000130513	-1.00083	0.305044	4.3E-11	1.37E-08	GDF15
ENSG00000130529	0.690654	0.223489	0.001999	0.018071	TRPM4
ENSG00000130558	1.498677	0.485184	0.002009	0.018099	OLFM1
ENSG00000130584	1.052808	0.217393	1.28E-06	7.29E-05	ZBTB46
ENSG00000130590	1.296543	0.449885	0.003952	0.02897	SAMD10
ENSG00000130592	-0.98911	0.244413	5.19E-05	0.00122	LSP1
ENSG00000130638	-0.70098	0.1363	2.71E-07	2.23E-05	ATXN10
ENSG00000130640	-1.00083	0.415442	0.001257	0.012958	TUBGCP2
ENSG00000130706	-0.65354	0.212398	0.002091	0.018621	ADRM1
ENSG00000130724	-1.00083	0.299635	0.000239	0.003914	CHMP2A
ENSG00000130725	-0.83346	0.223576	0.000193	0.003326	UBE2M
ENSG00000130770	-1.00083	0.340071	0.00118	0.012429	ATP5IF1
ENSG00000130816	0.745725	0.208033	0.000338	0.004983	DNMT1
ENSG00000130830	-0.97077	0.307448	0.001591	0.015391	MPP1
ENSG00000131023	0.638046	0.227569	0.005051	0.034331	LATS1
ENSG00000131044	3.53452	0.899905	8.58E-05	0.001801	TTLL9
ENSG00000131100	-0.62084	0.150182	3.57E-05	0.000934	ATP6V1E1
ENSG00000131143	-0.79954	0.275642	0.003724	0.027799	COX4I1
ENSG00000131149	1.38029	0.31685	1.32E-05	0.000445	GSE1

ENSG00000131165	-0.61455	0.194087	0.001544	0.015055	CHMP1A
ENSG00000131174	-0.91451	0.257915	0.000391	0.005598	COX7B
ENSG00000131203	1.411399	0.46442	0.002373	0.020466	IDO1
ENSG00000131263	0.956782	0.349487	0.006187	0.039548	RLIM
ENSG00000131374	1.363304	0.423032	0.00127	0.01305	TBC1D5
ENSG00000131378	1.115796	0.348508	0.001366	0.013734	RFTN1
ENSG00000131503	0.693465	0.179876	0.000116	0.002248	ANKHD1
ENSG00000131626	0.82296	0.286308	0.004048	0.02939	PPFIA1
ENSG00000131697	0.891197	0.314854	0.004647	0.032355	NPHP4
ENSG00000131724	1.172877	0.181153	9.51E-11	2.84E-08	IL13RA1
ENSG00000131759	0.801467	0.198022	5.18E-05	0.00122	RARA
ENSG00000131844	1.433661	0.524269	0.006246	0.039827	MCCC2
ENSG00000131966	-0.85637	0.173324	7.78E-07	4.94E-05	ACTR10
ENSG00000131981	-1.00083	0.243753	1.32E-06	7.44E-05	LGALS3
ENSG00000132002	-0.64273	0.147786	1.37E-05	0.000457	DNAJB1
ENSG00000132003	1.69158	0.457061	0.000215	0.003618	ZSWIM4
ENSG00000132170	-0.92835	0.319525	0.003668	0.027583	PPARG
ENSG00000132182	0.913178	0.275137	0.000903	0.01015	NUP210
ENSG00000132205	-1.00083	0.339749	0.000775	0.009079	EMILIN2
ENSG00000132274	0.72993	0.240341	0.002389	0.020563	TRIM22
ENSG00000132275	-0.74615	0.250134	0.002854	0.023381	RRP8
ENSG00000132300	1.671363	0.537171	0.001862	0.017168	PTCD3
ENSG00000132313	-0.8155	0.207153	8.26E-05	0.001761	MRPL35
ENSG00000132334	1.106814	0.27029	4.22E-05	0.001057	PTPRE
ENSG00000132341	-0.62954	0.217305	0.003767	0.028002	RAN
ENSG00000132356	1.311755	0.463192	0.004626	0.032264	PRKAA1
ENSG00000132432	-1.00083	0.349321	0.0001	0.002027	SEC61G
ENSG00000132467	-0.73548	0.246003	0.002792	0.022997	UTP3
ENSG00000132485	0.55007	0.154048	0.000356	0.00519	ZRANB2
ENSG00000132507	-0.69229	0.211644	0.001072	0.011621	EIF5A
ENSG00000132510	1.893771	0.381578	6.94E-07	0.000046	KDM6B
ENSG00000132522	-0.79119	0.251044	0.001624	0.01559	GPS2
ENSG00000132530	1.386184	0.280487	7.73E-07	4.94E-05	XAF1
ENSG00000132535	0.628766	0.214319	0.003349	0.026017	DLG4
ENSG00000132549	0.570451	0.13911	4.12E-05	0.001038	VPS13B
ENSG00000132581	-0.99248	0.345333	0.004053	0.029414	SDF2
ENSG00000132639	4.19728	1.196866	0.000453	0.006231	SNAP25
ENSG00000132694	0.772761	0.291455	0.008016	0.047192	ARHGEF11
ENSG00000132792	-1.00083	0.445877	0.000577	0.007332	CTNNBL1
ENSG00000132842	-0.74129	0.247907	0.002788	0.022976	AP3B1
ENSG00000132963	-1.00083	0.305271	7.43E-05	0.001616	POMP
ENSG00000133026	2.43659	0.839267	0.003693	0.027693	MYH10
ENSG00000133059	0.766741	0.274779	0.005264	0.035359	DSTYK
ENSG00000133111	1.065182	0.343464	0.001927	0.017589	RFXAP
ENSG00000133138	1.039712	0.299624	0.00052	0.006823	TBC1D8B
ENSG00000133142	-0.6396	0.224878	0.004452	0.031371	TCEAL4

ENSG00000133226	0.677946	0.235752	0.004032	0.0293	SRRM1
ENSG00000133246	-0.86147	0.304317	0.004643	0.032341	PRAM1
ENSG00000133265	-0.8749	0.2428	0.000314	0.004717	HSPBP1
ENSG00000133318	-0.56848	0.209899	0.006762	0.042005	RTN3
ENSG00000133561	3.013496	0.572717	1.43E-07	1.35E-05	GIMAP6
ENSG00000133574	1.254499	0.288684	1.39E-05	0.000461	GIMAP4
ENSG00000133704	0.97142	0.309684	0.001708	0.016138	IPO8
ENSG00000133739	1.335863	0.342028	9.39E-05	0.001922	LRRCC1
ENSG00000133805	1.042128	0.194864	8.89E-08	9.62E-06	AMPD3
ENSG00000133816	1.736377	0.549399	0.001575	0.015292	MICAL2
ENSG00000133835	-0.87248	0.211024	3.56E-05	0.000934	HSD17B4
ENSG00000133983	-1.00083	0.313235	0.000512	0.00675	COX16
ENSG00000134001	-0.5997	0.158861	0.00016	0.002894	EIF2S1
ENSG00000134042	-1.00083	0.684912	1.39E-07	1.34E-05	MRO
ENSG00000134049	-0.93719	0.26677	0.000443	0.00613	IER3IP1
ENSG00000134107	0.779557	0.246883	0.001591	0.015391	BHLHE40
ENSG00000134109	1.403448	0.391978	0.000343	0.005032	EDEM1
ENSG00000134153	-0.57252	0.214843	0.007703	0.04596	EMC7
ENSG00000134186	0.556371	0.203134	0.006164	0.039415	PRPF38B
ENSG00000134248	-1.00083	0.225157	2.72E-06	0.000127	LAMTOR5
ENSG00000134330	-0.91294	0.343565	0.007878	0.046678	IAH1
ENSG00000134352	1.873628	0.437774	1.87E-05	0.000564	IL6ST
ENSG00000134375	-1.00083	0.2887	8.84E-05	0.001835	TIMM17A
ENSG00000134444	0.863507	0.265534	0.001146	0.012188	RELCH
ENSG00000134460	8.930663	0.671713	2.46E-40	3.38E-36	IL2RA
ENSG00000134470	1.791679	0.653915	0.006145	0.039333	IL15RA
ENSG00000134480	-0.80924	0.304043	0.007777	0.046202	CCNH
ENSG00000134590	-0.9853	0.260558	0.000156	0.002842	RTL8C
ENSG00000134644	1.186645	0.416865	0.004419	0.031272	PUM1
ENSG00000134668	-1.00083	0.359938	0.000286	0.004387	SPOCD1
ENSG00000134684	-0.73302	0.153378	1.76E-06	9.24E-05	YARS1
ENSG00000134697	-1.00083	0.385906	0.000029	0.000801	GNL2
ENSG00000134744	1.482529	0.426532	0.000509	0.006737	TUT4
ENSG00000134815	-0.62617	0.19079	0.001031	0.011258	DHX34
ENSG00000134853	-1.00083	0.436908	0.000187	0.003254	PDGFRA
ENSG00000134897	1.632925	0.409427	6.65E-05	0.001495	BIVM
ENSG00000134905	-1.00083	0.255025	6.61E-05	0.001488	CARS2
ENSG00000134910	-0.70863	0.129659	4.62E-08	5.87E-06	STT3A
ENSG00000134954	2.493507	0.796434	0.001743	0.016368	ETS1
ENSG00000134996	-0.59903	0.2126	0.004838	0.033359	OSTF1
ENSG00000135047	-1.00083	0.343468	8.62E-08	9.47E-06	CTSL
ENSG00000135052	0.685295	0.252813	0.006714	0.041863	GOLM1
ENSG00000135069	-1.00083	0.655223	5.38E-05	0.001249	PSAT1
ENSG00000135074	5.873789	1.051591	2.33E-08	3.3E-06	ADAM19
ENSG00000135077	-1.00083	0.175286	5.18E-10	1.29E-07	HAVCR2
ENSG00000135094	-1.00083	0.487833	6.42E-05	0.001455	SDS

ENSG00000135124	-0.98886	0.307078	0.001281	0.013133	P2RX4
ENSG00000135148	0.950666	0.236157	5.68E-05	0.001311	TRAFD1
ENSG00000135218	-1.00083	0.418316	0.001742	0.016365	CD36
ENSG00000135363	-0.73799	0.235321	0.001712	0.016163	LMO2
ENSG00000135378	1.015831	0.258391	8.45E-05	0.00179	PRRG4
ENSG00000135404	-1.00083	0.287123	3.22E-07	2.52E-05	CD63
ENSG00000135473	0.835898	0.251188	0.000875	0.0099	PAN2
ENSG00000135476	1.046289	0.356133	0.003304	0.025863	ESPL1
ENSG00000135506	-0.7256	0.214645	0.000724	0.008625	OS9
ENSG00000135604	0.769328	0.179088	1.74E-05	0.000533	STX11
ENSG00000135617	-1.00083	0.271297	4.79E-05	0.001161	PRADC1
ENSG00000135624	-0.99408	0.226254	1.11E-05	0.000385	CCT7
ENSG00000135821	0.757148	0.280032	0.006855	0.042375	GLUL
ENSG00000135838	-0.75312	0.174716	1.63E-05	0.00051	NPL
ENSG00000135842	0.94196	0.315166	0.002801	0.023054	NIBAN1
ENSG00000135862	-0.52894	0.198211	0.007617	0.045567	LAMC1
ENSG00000135900	-0.58962	0.130558	6.3E-06	0.000249	MRPL44
ENSG00000135929	-1.00083	0.30785	5.49E-07	3.82E-05	CYP27A1
ENSG00000135940	-0.89515	0.221048	5.13E-05	0.001212	COX5B
ENSG00000135945	0.810268	0.166714	1.17E-06	6.88E-05	REV1
ENSG00000135968	1.320931	0.443807	0.002917	0.02371	GCC2
ENSG00000136026	-0.90964	0.188412	1.38E-06	0.000077	CKAP4
ENSG00000136051	1.045285	0.388779	0.007175	0.043822	WASHC4
ENSG00000136052	1.376976	0.29363	2.74E-06	0.000127	SLC41A2
ENSG00000136068	-1.00083	0.325633	4.13E-06	0.000177	FLNB
ENSG00000136108	0.676509	0.215408	0.001686	0.015986	CKAP2
ENSG00000136235	-1.00083	0.233832	6.38E-06	0.000252	GPNMB
ENSG00000136240	-0.514	0.167703	0.002177	0.019208	KDELR2
ENSG00000136271	-1.00083	0.362818	0.003315	0.025879	DDX56
ENSG00000136280	-0.81714	0.278439	0.003338	0.025968	CCM2
ENSG00000136381	0.89285	0.27901	0.001374	0.013779	IREB2
ENSG00000136504	1.229724	0.379157	0.001182	0.01243	KAT7
ENSG00000136522	-1.00083	0.250359	4.6E-08	5.87E-06	MRPL47
ENSG00000136531	1.667589	0.550846	0.002467	0.021052	SCN2A
ENSG00000136603	1.724127	0.438082	0.000083	0.001766	SKIL
ENSG00000136634	-1.00083	0.552674	0.00467	0.03247	IL10
ENSG00000136718	-0.91526	0.259091	0.000412	0.005831	IMP4
ENSG00000136720	-0.76461	0.263395	0.003697	0.027706	HS6ST1
ENSG00000136867	0.85414	0.240331	0.000379	0.005466	SLC31A2
ENSG00000136869	1.023343	0.270862	0.000158	0.002874	TLR4
ENSG00000136870	0.761525	0.286409	0.00784	0.046507	ZNF189
ENSG00000136888	-0.66308	0.219346	0.002503	0.021251	ATP6V1G1
ENSG00000136925	1.174722	0.360768	0.001129	0.012084	TSTD2
ENSG00000136930	-0.71241	0.190705	0.000187	0.003254	PSMB7
ENSG00000136950	-1.00083	0.242424	1.79E-05	0.000545	ARPC5L
ENSG00000136960	4.030095	0.970258	3.27E-05	0.000877	ENPP2

ENSG00000137075	0.688178	0.236485	0.003614	0.027306	RNF38
ENSG00000137100	-0.70266	0.235743	0.002877	0.023481	DCTN3
ENSG00000137106	-0.70826	0.23523	0.002605	0.021897	GRHPR
ENSG00000137161	-0.97888	0.35177	0.00539	0.035924	CNPY3
ENSG00000137193	1.457959	0.304427	1.67E-06	8.91E-05	PIM1
ENSG00000137200	0.51723	0.186002	0.005423	0.036044	CMTR1
ENSG00000137210	-0.77183	0.219692	0.000443	0.00613	TMEM14B
ENSG00000137261	-1.00083	0.73044	0.000466	0.006294	KIAA0319
ENSG00000137265	3.248343	0.457145	1.2E-12	6.57E-10	IRF4
ENSG00000137491	1.11777	0.394098	0.004564	0.031955	SLCO2B1
ENSG00000137501	1.986729	0.524051	0.00015	0.002764	SYTL2
ENSG00000137502	1.765065	0.622731	0.004591	0.032115	RAB30
ENSG00000137504	0.57938	0.185987	0.001838	0.016996	CREBZF
ENSG00000137642	2.14505	0.495419	1.49E-05	0.000486	SORL1
ENSG00000137656	-0.65588	0.244751	0.007367	0.044576	BUD13
ENSG00000137692	-0.93146	0.299416	0.001865	0.017181	DCUN1D5
ENSG00000137714	-1.00083	0.259661	4.48E-06	0.000188	FDX1
ENSG00000137747	0.869431	0.218295	6.81E-05	0.00152	TMPRSS13
ENSG00000137770	0.99098	0.319949	0.001953	0.017784	CTDSPL2
ENSG00000137806	-0.54857	0.133413	3.93E-05	0.001007	NDUFAF1
ENSG00000137976	-1.00083	0.803882	0.00045	0.006206	DNASE2B
ENSG00000138031	-1.00083	0.440518	0.004527	0.031789	ADCY3
ENSG00000138069	-0.52983	0.10642	6.4E-07	4.35E-05	RAB1A
ENSG00000138080	-1.00083	0.291944	0.000197	0.003374	EMILIN1
ENSG00000138175	-0.70622	0.255508	0.00571	0.037332	ARL3
ENSG00000138185	1.176602	0.397539	0.003079	0.024501	ENTPD1
ENSG00000138316	3.530407	0.577858	1E-09	2.37E-07	ADAMTS14
ENSG00000138326	-1.00083	0.423531	0.006249	0.03983	RPS24
ENSG00000138363	-1.00083	0.40209	0.002854	0.023381	ATIC
ENSG00000138375	-0.605	0.156396	0.00011	0.002161	SMARCAL1
ENSG00000138381	-0.74754	0.269055	0.005463	0.036266	ASNSD1
ENSG00000138385	-0.59846	0.146301	0.000043	0.00107	SSB
ENSG00000138386	1.643254	0.32539	4.42E-07	3.16E-05	NAB1
ENSG00000138433	-1.00083	0.252519	8.46E-08	9.36E-06	CIR1
ENSG00000138448	1.007223	0.361669	0.005354	0.035733	ITGAV
ENSG00000138593	1.988454	0.61538	0.001232	0.012838	SECISBP2L
ENSG00000138613	-0.77166	0.200245	0.000116	0.002254	APH1B
ENSG00000138688	0.733085	0.188294	9.89E-05	0.002	KIAA1109
ENSG00000138738	1.624437	0.510963	0.001477	0.014578	PRDM5
ENSG00000138755	3.181459	1.037855	0.002174	0.019191	CXCL9
ENSG00000138760	-1.00083	0.270614	3.14E-07	2.47E-05	SCARB2
ENSG00000138764	1.104074	0.396888	0.005405	0.03599	CCNG2
ENSG00000138767	0.879605	0.282813	0.00187	0.017181	CNOT6L
ENSG00000138795	4.941076	1.143394	1.55E-05	0.000498	LEF1
ENSG00000138867	-1.00083	0.379286	0.005818	0.037729	GUCD1
ENSG00000139083	0.639159	0.18259	0.000464	0.006293	ETV6

ENSG00000139168	-0.66155	0.188766	0.000457	0.00626	ZCRB1
ENSG00000139178	3.507089	0.505868	4.13E-12	1.77E-09	C1RL
ENSG00000139193	1.097462	0.287711	0.000136	0.002563	CD27
ENSG00000139233	-0.74666	0.172778	1.55E-05	0.000498	LLPH
ENSG00000139344	-1.00083	0.433544	0.001196	0.012543	AMDHD1
ENSG00000139410	-1.00083	0.370913	0.00276	0.022806	SDSL
ENSG00000139505	0.647311	0.245753	0.008439	0.048905	MTMR6
ENSG00000139597	1.285002	0.28111	4.85E-06	0.000201	N4BP2L1
ENSG00000139618	1.643079	0.535434	0.00215	0.019019	BRCA2
ENSG00000139624	-0.60934	0.230381	0.008171	0.047795	CERS5
ENSG00000139626	1.181571	0.397515	0.002955	0.023905	ITGB7
ENSG00000139631	1.123328	0.276246	4.77E-05	0.001159	CSAD
ENSG00000139637	-0.76365	0.262281	0.003596	0.027187	MYG1
ENSG00000139651	1.499084	0.45317	0.00094	0.010491	ZNF740
ENSG00000139668	1.877755	0.499065	0.000168	0.003007	WDFY2
ENSG00000139899	2.515107	0.838752	0.002712	0.022511	CBLN3
ENSG00000139974	-0.97276	0.238764	4.62E-05	0.001128	SLC38A6
ENSG00000139990	0.644821	0.242876	0.007932	0.046879	DCAF5
ENSG00000140262	1.002479	0.362898	0.005737	0.037473	TCF12
ENSG00000140264	-1.00083	0.331468	0.000232	0.003838	SERF2
ENSG00000140307	-0.80267	0.296316	0.006752	0.041979	GTF2A2
ENSG00000140319	-0.88833	0.14736	1.66E-09	3.65E-07	SRP14
ENSG00000140350	-0.69746	0.172169	0.000051	0.001207	ANP32A
ENSG00000140391	-0.58036	0.158581	0.000253	0.004022	TSPAN3
ENSG00000140396	1.296236	0.324842	0.000066	0.001488	NCOA2
ENSG00000140400	0.558085	0.124485	7.35E-06	0.000278	MAN2C1
ENSG00000140443	2.830037	0.751557	0.000166	0.002974	IGF1R
ENSG00000140511	3.70442	0.977708	0.000151	0.002785	HAPLN3
ENSG00000140577	0.854168	0.132525	1.15E-10	3.26E-08	CRTC3
ENSG00000140632	0.656565	0.234955	0.005199	0.035093	GLYR1
ENSG00000140718	0.926126	0.326975	0.00462	0.032246	FTO
ENSG00000140749	2.149673	0.46741	4.24E-06	0.000181	IGSF6
ENSG00000140853	1.102005	0.22927	1.54E-06	8.36E-05	NLRC5
ENSG00000140990	-0.90409	0.284081	0.00146	0.014427	NDUFB10
ENSG00000141068	2.985758	0.825244	0.000297	0.004518	KSR1
ENSG00000141378	-1.00083	0.254127	1.33E-07	0.000013	PTRH2
ENSG00000141503	1.109381	0.336639	0.000983	0.010844	MINK1
ENSG00000141506	0.793254	0.228682	0.000523	0.006835	PIK3R5
ENSG00000141510	0.622432	0.232939	0.007538	0.045312	TP53
ENSG00000141522	-0.67322	0.184519	0.000264	0.004148	ARHGDIA
ENSG00000141741	-1.00083	0.326318	0.000554	0.007103	MIEN1
ENSG00000141759	-1.00083	0.324262	3.51E-05	0.000925	TXNL4A
ENSG00000141905	0.612495	0.231467	0.008141	0.047665	NFIC
ENSG00000142168	-1.00083	0.234594	1.57E-08	2.37E-06	SOD1
ENSG00000142186	-0.97627	0.285541	0.000628	0.007822	SCYL1
ENSG00000142227	-1.00083	0.346755	2.42E-05	0.000697	EMP3

ENSG00000142327	-0.93361	0.347986	0.007299	0.044261	RNPEPL1
ENSG00000142507	-1.00083	0.262303	0.000103	0.002062	PSMB6
ENSG00000142512	2.163617	0.439357	8.46E-07	5.28E-05	SIGLEC10
ENSG00000142546	-1.00083	0.320548	0.001259	0.01296	NOSIP
ENSG00000142599	0.897447	0.267323	0.000787	0.009162	RERE
ENSG00000142655	-0.51675	0.163169	0.00154	0.015042	PEX14
ENSG00000142669	-1.00083	0.204106	1.86E-08	2.72E-06	SH3BGRL3
ENSG00000142684	-1.00083	0.745362	0.008505	0.049173	ZNF593
ENSG00000142686	-0.62423	0.174197	0.000339	0.004995	C1orf216
ENSG00000142687	0.724066	0.229168	0.00158	0.015325	KIAA0319L
ENSG00000142751	-0.72816	0.243048	0.002736	0.022656	GPN2
ENSG00000142949	2.83949	0.85983	0.000959	0.01064	PTPRF
ENSG00000143033	1.112202	0.414884	0.007346	0.044485	MTF2
ENSG00000143106	-0.72074	0.226396	0.001455	0.014402	PSMA5
ENSG00000143110	-0.98147	0.286441	0.000612	0.007696	C1orf162
ENSG00000143153	-0.6262	0.21027	0.002901	0.023621	ATP1B1
ENSG00000143198	-1.00083	0.273751	3.24E-06	0.000145	MGST3
ENSG00000143207	-1.00083	0.364173	7.22E-05	0.001587	COP1
ENSG00000143222	-0.78201	0.196203	6.73E-05	0.001509	UFC1
ENSG00000143226	1.235274	0.341155	0.000294	0.004484	FCGR2A
ENSG00000143248	1.774979	0.658184	0.007001	0.042988	RGS5
ENSG00000143256	-0.73869	0.20749	0.000371	0.005368	PFDN2
ENSG00000143294	-0.79133	0.290797	0.006504	0.040808	PRCC
ENSG00000143314	-1.00083	0.346125	0.001069	0.011602	MRPL24
ENSG00000143322	0.890799	0.333057	0.007482	0.04507	ABL2
ENSG00000143337	0.689062	0.209289	0.000993	0.010945	TOR1AIP1
ENSG00000143353	-1.00083	0.41791	0.004684	0.032527	LYPLAL1
ENSG00000143369	-0.87028	0.212539	4.23E-05	0.001057	ECM1
ENSG00000143382	1.582508	0.593998	0.007718	0.04601	ADAMTSL4
ENSG00000143384	1.087924	0.239819	5.72E-06	0.00023	MCL1
ENSG00000143390	1.809624	0.287289	3E-10	7.91E-08	RFX5
ENSG00000143398	1.393581	0.373899	0.000194	0.003332	PIP5K1A
ENSG00000143401	0.9577	0.35521	0.007015	0.043031	ANP32E
ENSG00000143418	-0.63206	0.194208	0.001136	0.012124	CERS2
ENSG00000143437	1.293167	0.357913	0.000303	0.00459	ARNT
ENSG00000143442	0.929407	0.296464	0.001719	0.016195	POGZ
ENSG00000143515	1.597146	0.577845	0.00571	0.037332	ATP8B2
ENSG00000143543	-0.97815	0.279584	0.000468	0.006302	JTB
ENSG00000143621	-0.70281	0.169616	3.42E-05	0.000905	ILF2
ENSG00000143653	-1.00083	0.385963	0.000533	0.006939	SCCPDH
ENSG00000143771	-0.6389	0.224864	0.004493	0.031571	CNIH4
ENSG00000143801	-0.61541	0.192158	0.001362	0.013708	PSEN2
ENSG00000143815	0.628152	0.216758	0.003756	0.027952	LBR
ENSG00000143819	-1.00083	0.336984	0.000568	0.007225	EPHX1
ENSG00000143862	-0.88576	0.196708	6.7E-06	0.000259	ARL8A
ENSG00000143870	-1.00083	0.173479	5E-13	3.12E-10	PDIA6

ENSG00000143924	0.841816	0.26077	0.001246	0.012892	EML4
ENSG00000143970	1.305047	0.403809	0.00123	0.012822	ASXL2
ENSG00000144029	-0.67168	0.227265	0.003121	0.024757	MRPS5
ENSG00000144136	0.959413	0.328586	0.003502	0.026756	SLC20A1
ENSG00000144228	0.906873	0.259208	0.000468	0.006302	SPOPL
ENSG00000144354	2.497058	0.857621	0.003596	0.027187	CDCA7
ENSG00000144406	0.969299	0.349226	0.005511	0.036496	UNC80
ENSG00000144468	0.767891	0.230234	0.000852	0.009725	RHBDD1
ENSG00000144550	-1.00083	0.535226	3.93E-05	0.001007	CPNE9
ENSG00000144644	1.145728	0.402887	0.004458	0.031371	GADL1
ENSG00000144655	1.632119	0.412375	7.56E-05	0.001635	CSRNP1
ENSG00000144681	3.654626	0.877583	3.12E-05	0.000847	STAC
ENSG00000144744	0.68435	0.230105	0.002939	0.02383	UBA3
ENSG00000144747	1.241802	0.410417	0.00248	0.021126	TMF1
ENSG00000144802	2.580291	0.492403	1.6E-07	1.46E-05	NFKBIZ
ENSG00000144815	0.640335	0.187597	0.000642	0.007937	NXPE3
ENSG00000145012	1.03217	0.375094	0.005927	0.038296	LPP
ENSG00000145016	0.910756	0.233675	9.72E-05	0.001974	RUBCN
ENSG00000145050	-1.00083	0.305516	6.22E-11	1.94E-08	MANF
ENSG00000145103	2.739642	0.845215	0.00119	0.012496	ILDR1
ENSG00000145191	-0.50364	0.176644	0.004356	0.030919	EIF2B5
ENSG00000145217	2.575145	0.962199	0.007444	0.044881	SLC26A1
ENSG00000145220	-1.00083	0.226748	8.8E-06	0.000319	LYAR
ENSG00000145348	1.009388	0.318279	0.001517	0.014867	TBCK
ENSG00000145416	0.80815	0.29314	0.005836	0.037816	MARCHF1
ENSG00000145431	1.012445	0.348854	0.003705	0.027724	PDGFC
ENSG00000145494	-1.00083	0.344365	0.002465	0.021044	NDUFS6
ENSG00000145708	-1.00083	0.992391	0.005764	0.037545	CRHBP
ENSG00000145740	-0.86948	0.194118	7.49E-06	0.000283	SLC30A5
ENSG00000145832	2.215903	0.510698	1.43E-05	0.00047	SLC25A48
ENSG00000145850	-1.00083	0.606626	0.000618	0.007759	TIMD4
ENSG00000145936	1.953054	0.560726	0.000496	0.006596	KCNMB1
ENSG00000145982	-0.76958	0.291226	0.008229	0.048032	FARS2
ENSG00000146063	0.683716	0.194929	0.000452	0.006222	TRIM41
ENSG00000146070	-0.72052	0.160459	7.11E-06	0.00027	PLA2G7
ENSG00000146083	1.337394	0.274328	1.09E-06	6.52E-05	RNF44
ENSG00000146192	1.032339	0.367589	0.004979	0.03399	FGD2
ENSG00000146247	1.089793	0.278133	8.92E-05	0.001844	PHIP
ENSG00000146263	1.324299	0.420295	0.001628	0.015617	MMS22L
ENSG00000146278	1.703895	0.242585	2.16E-12	1.02E-09	PNRC1
ENSG00000146386	-1.00083	0.375178	0.003972	0.029064	ABRACL
ENSG00000146409	-1.00083	0.431782	0.000012	0.000412	SLC18B1
ENSG00000146414	0.959649	0.309241	0.001914	0.01752	SHPRH
ENSG00000146540	-0.86477	0.302849	0.004298	0.030634	C7orf50
ENSG00000146574	-0.91292	0.289372	0.001606	0.015472	CCZ1B
ENSG00000146701	-0.71024	0.249686	0.004448	0.031364	MDH2

ENSG00000146731	-0.86499	0.296421	0.003522	0.026831	CCT6A
ENSG00000146904	-1.00083	0.62119	0.002411	0.020709	EPHA1
ENSG00000146963	0.643764	0.180954	0.000374	0.005403	LUC7L2
ENSG00000147119	-0.96121	0.345643	0.00542	0.036044	CHST7
ENSG00000147123	-1.00083	0.364934	0.000562	0.007182	NDUFB11
ENSG00000147130	0.604285	0.203296	0.002954	0.023905	ZMYM3
ENSG00000147155	-1.00083	0.378798	0.000981	0.010837	EBP
ENSG00000147162	0.779998	0.260329	0.002734	0.022649	OGT
ENSG00000147383	-1.00083	0.266138	5.24E-05	0.001223	NSDHL
ENSG00000147408	2.685787	0.554027	1.25E-06	7.17E-05	CSGALNACT1
ENSG00000147421	0.883982	0.211466	2.91E-05	0.000803	HMBOX1
ENSG00000147454	1.36023	0.453423	0.002701	0.022457	SLC25A37
ENSG00000147614	-1.00083	0.554575	0.004792	0.033082	ATP6V0D2
ENSG00000147684	-1.00083	0.343852	4.4E-08	5.76E-06	NDUFB9
ENSG00000147872	-1.00083	0.348938	6.02E-06	0.00024	PLIN2
ENSG00000147996	-0.869	0.283872	0.002204	0.019373	CBWD5
ENSG00000148218	0.844879	0.29099	0.003691	0.027688	ALAD
ENSG00000148296	-0.94056	0.336509	0.005189	0.035052	SURF6
ENSG00000148384	1.387687	0.478635	0.00374	0.027894	INPP5E
ENSG00000148700	1.215058	0.449848	0.006912	0.042607	ADD3
ENSG00000148834	-1.00083	0.283549	7.76E-07	4.94E-05	GSTO1
ENSG00000149196	-1.00083	0.412121	0.003708	0.027724	HIKESHI
ENSG00000149212	1.287962	0.387231	0.000881	0.009936	SESN3
ENSG00000149218	-1.00083	0.396673	0.005546	0.036651	ENDOD1
ENSG00000149311	0.820685	0.225082	0.000266	0.004167	ATM
ENSG00000149357	-0.80405	0.249773	0.001286	0.013165	LAMTOR1
ENSG00000149428	-1.00083	0.267888	1.03E-05	0.000361	HYOU1
ENSG00000149483	-0.9647	0.243257	7.32E-05	0.001602	TMEM138
ENSG00000149532	0.654943	0.180309	0.000281	0.004323	CPSF7
ENSG00000149600	-1.00083	0.258914	2.24E-05	0.00065	COMMD7
ENSG00000149781	-0.84247	0.259412	0.001164	0.012318	FERMT3
ENSG00000149925	-0.97817	0.195919	5.95E-07	4.09E-05	ALDOA
ENSG00000149932	-0.9682	0.329825	0.00333	0.025934	TMEM219
ENSG00000150337	-1.00083	0.61109	0.00145	0.014368	FCGR1A
ENSG00000150347	1.285521	0.229324	2.07E-08	2.97E-06	ARID5B
ENSG00000150510	1.810865	0.660877	0.006142	0.039331	FAM124A
ENSG00000150753	-0.72809	0.13293	4.32E-08	5.7E-06	CCT5
ENSG00000150779	-1.00083	0.283452	0.000046	0.001127	TIMM8B
ENSG00000150938	1.326997	0.473472	0.005068	0.034426	CRIM1
ENSG00000150991	-0.73315	0.237107	0.001988	0.017988	UBC
ENSG00000151012	1.450423	0.324856	8.01E-06	0.000297	SLC7A11
ENSG00000151148	0.799787	0.247702	0.001243	0.012892	UBE3B
ENSG00000151338	2.443662	0.748809	0.001101	0.011844	MIPOL1
ENSG00000151366	-0.9215	0.290653	0.001522	0.014884	NDUFC2
ENSG00000151414	1.036364	0.296144	0.000466	0.006294	NEK7
ENSG00000151465	-0.66643	0.165255	5.51E-05	0.001276	CDC123

ENSG00000151498	0.627469	0.209101	0.002693	0.02244	ACAD8
ENSG00000151503	1.510765	0.286976	1.41E-07	1.34E-05	NCAPD3
ENSG00000151552	-0.99146	0.235017	2.46E-05	0.000704	QDPR
ENSG00000151553	1.195585	0.406695	0.003285	0.025784	FAM160B1
ENSG00000151576	1.057918	0.318567	0.000897	0.01009	QTRT2
ENSG00000151694	0.956467	0.333088	0.004085	0.029596	ADAM17
ENSG00000151718	1.215851	0.281697	1.59E-05	0.000506	WWC2
ENSG00000151726	-0.84092	0.212705	0.000077	0.00166	ACSL1
ENSG00000151748	1.031355	0.356219	0.003788	0.028112	SAV1
ENSG00000151779	-0.8636	0.272682	0.00154	0.015042	NBAS
ENSG00000151849	0.867745	0.29423	0.003186	0.025123	CENPJ
ENSG00000151893	0.85046	0.20405	3.07E-05	0.000839	CACUL1
ENSG00000152061	-0.63325	0.221086	0.00418	0.03009	RABGAP1L
ENSG00000152229	1.542973	0.305367	4.35E-07	3.13E-05	PSTPIP2
ENSG00000152242	0.671828	0.235318	0.004304	0.030645	C18orf25
ENSG00000152439	1.358226	0.508955	0.007615	0.045567	ZNF773
ENSG00000152457	0.737776	0.258047	0.004249	0.030428	DCLRE1C
ENSG00000152495	4.267439	0.545195	4.98E-15	5.26E-12	CAMK4
ENSG00000152556	0.554486	0.200766	0.005747	0.037485	PFKM
ENSG00000152583	-1.00083	0.975601	0.001804	0.016776	SPARCL1
ENSG00000152601	0.98917	0.202179	9.95E-07	6.02E-05	MBNL1
ENSG00000152782	1.208708	0.387823	0.001829	0.016934	PANK1
ENSG00000152784	2.028398	0.641089	0.001556	0.015153	PRDM8
ENSG00000153012	-1.00083	0.817453	3.78E-06	0.000166	LGI2
ENSG00000153037	-0.95601	0.251701	0.000146	0.002697	SRP19
ENSG00000153048	-0.73378	0.228358	0.001312	0.013386	CARHSP1
ENSG00000153066	-0.99497	0.270112	0.00023	0.003814	TXNDC11
ENSG00000153094	2.169609	0.332503	6.8E-11	2.07E-08	BCL2L11
ENSG00000153234	1.638868	0.544828	0.002629	0.022025	NR4A2
ENSG00000153283	4.079854	0.987951	3.63E-05	0.000947	CD96
ENSG00000153317	1.264878	0.356708	0.000391	0.005598	ASAP1
ENSG00000153485	-1.00083	0.359221	3.09E-05	0.000839	TMEM251
ENSG00000153774	-0.96317	0.263036	0.000251	0.004022	CFDP1
ENSG00000153879	-0.70209	0.238167	0.003199	0.025216	CEBPG
ENSG00000153944	1.394542	0.530365	0.008554	0.0493	MSI2
ENSG00000154114	1.269974	0.278391	5.07E-06	0.000207	TBCEL
ENSG00000154217	-1.00083	0.248898	8.39E-08	9.36E-06	PITPNC1
ENSG00000154237	0.946184	0.257482	0.000238	0.00391	LRRK1
ENSG00000154265	0.763743	0.251978	0.002437	0.020863	ABCA5
ENSG00000154277	-1.00083	0.370919	3.95E-07	2.92E-05	UCHL1
ENSG00000154310	1.049047	0.334523	0.001713	0.016163	TNIK
ENSG00000154370	-0.52396	0.191859	0.006315	0.040157	TRIM11
ENSG00000154451	2.583304	0.36401	1.28E-12	6.74E-10	GBP5
ENSG00000154518	-0.82259	0.166648	7.97E-07	5.04E-05	ATP5MC3
ENSG00000154582	-1.00083	0.225639	1.14E-08	1.77E-06	ELOC
ENSG00000154589	-1.00083	0.362305	0.00046	0.006272	LY96

ENSG00000154723	-1.00083	0.33844	0.001138	0.012134	ATP5PF
ENSG00000154760	1.673601	0.317841	1.4E-07	1.34E-05	SLFN13
ENSG00000154874	1.430235	0.437673	0.001084	0.011698	CCDC144B
ENSG00000155100	0.861468	0.276964	0.001868	0.017181	OTUD6B
ENSG00000155111	0.728176	0.183344	7.14E-05	0.001576	CDK19
ENSG00000155115	-1.00083	0.24487	8.7E-07	5.38E-05	GTF3C6
ENSG00000155189	1.338097	0.491022	0.006428	0.04055	AGPAT5
ENSG00000155265	-1.00083	0.641639	1.79E-07	1.59E-05	GOLGA7B
ENSG00000155366	-0.89439	0.307683	0.003651	0.027493	RHOC
ENSG00000155368	-1.00083	0.280482	1.48E-07	1.37E-05	DBI
ENSG00000155506	1.11103	0.346152	0.001329	0.013493	LARP1
ENSG00000155561	-1.00083	0.291222	0.000241	0.003922	NUP205
ENSG00000155657	1.197295	0.233957	3.09E-07	2.46E-05	TTN
ENSG00000155660	-0.68804	0.19814	0.000516	0.006787	PDIA4
ENSG00000155719	-1.00083	0.638524	0.000074	0.001612	OTOA
ENSG00000155729	1.111691	0.41057	0.006776	0.042072	KCTD18
ENSG00000155755	1.361796	0.385657	0.000414	0.005851	TMEM237
ENSG00000155893	2.564754	0.66108	0.000105	0.002094	PXYLP1
ENSG00000155962	0.911759	0.247157	0.000225	0.003747	CLIC2
ENSG00000156030	0.854197	0.256758	0.000878	0.009919	MIDEAS
ENSG00000156050	-1.00083	0.846833	8.22E-05	0.001756	FAM161B
ENSG00000156261	-0.63602	0.153851	3.56E-05	0.000934	CCT8
ENSG00000156411	-1.00083	0.290877	0.000105	0.002096	ATP5MJ
ENSG00000156502	-0.99983	0.343738	0.003629	0.027377	SUPV3L1
ENSG00000156515	-0.5241	0.189193	0.005602	0.036802	HK1
ENSG00000156639	0.517174	0.171838	0.002615	0.021962	ZFAND3
ENSG00000156802	1.036407	0.188715	3.98E-08	5.4E-06	ATAD2
ENSG00000156928	-0.6596	0.225561	0.003452	0.026569	MALSU1
ENSG00000156976	0.531914	0.145055	0.000245	0.003969	EIF4A2
ENSG00000157020	-0.93502	0.165514	1.61E-08	2.41E-06	SEC13
ENSG00000157036	0.883703	0.283345	0.001816	0.016866	EXOG
ENSG00000157150	-1.00083	0.696137	0.00212	0.018813	TIMP4
ENSG00000157212	0.677456	0.247048	0.006103	0.039195	PAXIP1
ENSG00000157350	0.937181	0.274645	0.000644	0.007945	ST3GAL2
ENSG00000157445	-1.00083	1.036692	0.000656	0.008035	CACNA2D3
ENSG00000157540	1.060849	0.326694	0.001165	0.012318	DYRK1A
ENSG00000157617	0.998546	0.210328	2.06E-06	0.000105	C2CD2
ENSG00000157625	0.889008	0.252297	0.000426	0.005944	TAB3
ENSG00000157823	0.993998	0.307067	0.001208	0.012637	AP3S2
ENSG00000157827	0.555226	0.179738	0.002008	0.018098	FMNL2
ENSG00000157933	1.277767	0.346813	0.000229	0.003807	SKI
ENSG00000158042	-0.94858	0.311616	0.002334	0.020179	MRPL17
ENSG00000158062	-1.00083	0.317643	0.001212	0.012669	UBXN11
ENSG00000158089	-1.00083	0.895206	0.004109	0.029723	GALNT14
ENSG00000158156	-0.85932	0.174605	8.59E-07	5.34E-05	XKR8
ENSG00000158163	1.808278	0.367932	8.89E-07	5.47E-05	DZIP1L

ENSG00000158234	-1.00083	0.277317	0.000051	0.001207	FAIM
ENSG00000158258	2.536515	0.885745	0.004187	0.030129	CLSTN2
ENSG00000158270	1.405827	0.457531	0.002122	0.018818	COLEC12
ENSG00000158286	2.439043	0.634278	0.00012	0.002314	RNF207
ENSG00000158417	-0.74355	0.2216	0.000793	0.009197	EIF5B
ENSG00000158473	0.984097	0.278108	0.000402	0.005724	CD1D
ENSG00000158485	1.426618	0.395599	0.000311	0.004682	CD1B
ENSG00000158488	4.040342	0.797334	4.03E-07	2.93E-05	CD1E
ENSG00000158526	-0.5147	0.178178	0.003869	0.028539	TSR2
ENSG00000158615	0.76535	0.268666	0.00439	0.031129	PPP1R15B
ENSG00000158691	1.403307	0.389423	0.000314	0.004717	ZSCAN12
ENSG00000158792	-0.9043	0.334401	0.006846	0.04234	SPATA2L
ENSG00000158882	-0.54737	0.204291	0.007377	0.044615	TOMM40L
ENSG00000158987	1.117913	0.386942	0.003863	0.028517	RAPGEF6
ENSG00000159086	0.594507	0.191207	0.001876	0.017226	PAXBP1
ENSG00000159128	1.218479	0.398246	0.002216	0.019428	IFNGR2
ENSG00000159199	-0.6766	0.242606	0.005289	0.035472	ATP5MC1
ENSG00000159210	-1.00083	0.304764	7.39E-05	0.001612	SNF8
ENSG00000159228	-0.66022	0.219156	0.002591	0.021846	CBR1
ENSG00000159322	0.525051	0.134395	9.35E-05	0.001922	ADPGK
ENSG00000159335	-0.93418	0.271951	0.000592	0.007488	PTMS
ENSG00000159352	-1.00083	0.30009	0.000662	0.008075	PSMD4
ENSG00000159363	-1.00083	0.330984	0.000493	0.00657	ATP13A2
ENSG00000159720	-1.00083	0.227964	4.01E-06	0.000174	ATP6V0D1
ENSG00000159733	-1.00083	0.226765	7.53E-06	0.000283	ZFYVE28
ENSG00000159840	-0.92201	0.17133	7.39E-08	8.31E-06	ZYX
ENSG00000160007	0.668954	0.244941	0.006313	0.040157	ARHGAP35
ENSG00000160014	-1.00083	0.326959	0.000238	0.003912	CALM3
ENSG00000160209	-0.7943	0.282571	0.004939	0.033866	PDXK
ENSG00000160213	-1.00083	0.269775	1.03E-05	0.000361	CSTB
ENSG00000160216	0.649444	0.150985	0.000017	0.000525	AGPAT3
ENSG00000160218	0.973363	0.353246	0.005861	0.037935	TRAPPC10
ENSG00000160271	0.815368	0.297346	0.006104	0.039195	RALGDS
ENSG00000160299	0.687188	0.208253	0.000968	0.010696	PCNT
ENSG00000160584	0.743018	0.264848	0.005025	0.034201	SIK3
ENSG00000160654	2.332889	0.866309	0.007083	0.043374	CD3G
ENSG00000160710	0.626931	0.203317	0.002046	0.018321	ADAR
ENSG00000160752	-1.00083	0.297532	0.000334	0.004946	FDPS
ENSG00000160783	-0.97265	0.361844	0.007187	0.043822	PMF1
ENSG00000160789	-1.00083	0.268773	0.000141	0.002636	LMNA
ENSG00000160799	-0.95725	0.330833	0.00381	0.0282	CCDC12
ENSG00000160818	-1.00083	0.223572	8.81E-09	1.49E-06	GPATCH4
ENSG00000160932	-1.00083	0.376361	0.000136	0.00255	LY6E
ENSG00000161016	-1.00083	0.375512	0.001503	0.014771	RPL8
ENSG00000161526	-0.91873	0.254516	0.000307	0.00463	SAP30BP
ENSG00000161558	1.210321	0.429094	0.004793	0.033082	TMEM143

ENSG00000161692	0.805445	0.264946	0.002365	0.020412	DBF4B
ENSG00000161791	0.630247	0.229136	0.00595	0.038396	FMNL3
ENSG00000161835	2.936862	0.673937	1.31E-05	0.000443	TAMALIN
ENSG00000161847	1.345846	0.366757	0.000243	0.003938	RAVER1
ENSG00000161888	1.583568	0.564657	0.00504	0.03427	SPC24
ENSG00000161905	3.734513	1.353643	0.0058	0.037669	ALOX15
ENSG00000161911	-1.00083	0.239757	1.62E-05	0.00051	TREML1
ENSG00000161929	1.454114	0.49779	0.003488	0.026696	SCIMP
ENSG00000161960	-1.00083	0.212947	1.19E-06	0.000069	EIF4A1
ENSG00000161981	-1.00083	0.30924	1.91E-06	0.000098	SNRNP25
ENSG00000162441	-0.70452	0.149551	2.47E-06	0.000117	LZIC
ENSG00000162444	-1.00083	0.784038	1.33E-05	0.000447	RBP7
ENSG00000162458	-1.00083	0.676674	1.19E-10	3.26E-08	FBLIM1
ENSG00000162496	-1.00083	0.310246	1.54E-05	0.000498	DHRS3
ENSG00000162511	-0.6375	0.191181	0.000854	0.009735	LAPTM5
ENSG00000162520	-1.00083	0.388139	0.000104	0.002082	SYNC
ENSG00000162576	1.116797	0.313561	0.000369	0.005343	MXRA8
ENSG00000162601	1.188548	0.33874	0.00045	0.006206	MYSM1
ENSG00000162654	2.074646	0.55639	0.000192	0.003319	GBP4
ENSG00000162723	-1.00083	0.477716	1.45E-05	0.000474	SLAMF9
ENSG00000162771	-1.00083	0.890868	0.005264	0.035359	FAM71A
ENSG00000162777	-1.00083	0.237879	1.19E-07	0.000012	DENND2D
ENSG00000162869	-1.00083	0.399496	0.008483	0.049084	PPP1R21
ENSG00000162909	-0.90373	0.304654	0.003013	0.024119	CAPN2
ENSG00000162924	1.457965	0.431679	0.000732	0.008697	REL
ENSG00000163013	0.784011	0.267598	0.003392	0.026315	FBXO41
ENSG00000163106	-1.00083	0.943414	0.001963	0.017822	HPGDS
ENSG00000163191	-1.00083	0.255521	2.46E-07	2.08E-05	S100A11
ENSG00000163220	-1.00083	0.445489	1.57E-05	0.000503	S100A9
ENSG00000163328	1.137864	0.407368	0.005219	0.035191	GPR155
ENSG00000163344	-1.00083	0.362251	0.002247	0.019621	PMVK
ENSG00000163357	2.401009	0.900945	0.007699	0.045957	DCST1
ENSG00000163430	3.161011	0.668836	2.29E-06	0.000111	FSTL1
ENSG00000163463	-1.00083	0.35967	0.000685	0.008274	KRTCAP2
ENSG00000163468	-0.73452	0.208894	0.000438	0.006087	CCT3
ENSG00000163479	-1.00083	0.288107	4.04E-05	0.001024	SSR2
ENSG00000163482	1.611383	0.214482	5.78E-14	4.67E-11	STK36
ENSG00000163539	0.722631	0.249586	0.003788	0.028112	CLASP2
ENSG00000163545	1.23671	0.406745	0.002362	0.020393	NUAK2
ENSG00000163564	1.844674	0.586513	0.00166	0.015837	PYHIN1
ENSG00000163600	4.704752	1.096209	1.77E-05	0.000541	ICOS
ENSG00000163606	1.976695	0.446197	9.42E-06	0.000335	CD200R1
ENSG00000163625	0.874716	0.32177	0.006559	0.041061	WDFY3
ENSG00000163635	1.441814	0.418806	0.000576	0.007322	ATXN7
ENSG00000163636	-0.66772	0.160894	3.32E-05	0.000887	PSMD6
ENSG00000163661	-1.00083	0.663431	0.006367	0.040357	PTX3

ENSG00000163686	-0.64607	0.158971	4.82E-05	0.001164	ABHD6
ENSG00000163687	-1.00083	0.619955	0.000775	0.009079	DNASE1L3
ENSG00000163735	2.962816	0.95943	0.002014	0.018123	CXCL5
ENSG00000163743	0.556106	0.184592	0.00259	0.021846	RCHY1
ENSG00000163848	1.514934	0.51346	0.003173	0.025037	ZNF148
ENSG00000163866	-0.67562	0.210041	0.001297	0.013261	SMIM12
ENSG00000163867	0.900869	0.273496	0.000988	0.010896	ZMYM6
ENSG00000163870	-1.00083	0.367076	0.000866	0.009835	TPRA1
ENSG00000163874	1.18876	0.302318	8.42E-05	0.001786	ZC3H12A
ENSG00000163902	-0.98153	0.129085	2.88E-14	2.47E-11	RPN1
ENSG00000163938	-0.645	0.176097	0.00025	0.004011	GNL3
ENSG00000163945	1.113342	0.269766	3.67E-05	0.000955	UVSSA
ENSG00000163956	-0.76613	0.285017	0.007188	0.043822	LRPAP1
ENSG00000163958	-1.00083	0.734884	0.007014	0.043031	ZDHHC19
ENSG00000163995	-1.00083	1.135962	0.004108	0.029723	ABLIM2
ENSG00000164047	-1.00083	0.655139	1.13E-06	6.73E-05	CAMP
ENSG00000164074	0.705435	0.210868	0.000822	0.009461	ABHD18
ENSG00000164081	-0.64553	0.212048	0.002332	0.020178	TEX264
ENSG00000164111	-0.55933	0.192708	0.003702	0.027724	ANXA5
ENSG00000164136	0.965899	0.326093	0.003056	0.024368	IL15
ENSG00000164168	1.258731	0.417952	0.002598	0.021884	TMEM184C
ENSG00000164237	-1.00083	0.667148	0.002652	0.022191	CMBL
ENSG00000164258	-1.00083	0.289321	0.000257	0.004062	NDUFS4
ENSG00000164307	1.012676	0.371664	0.006436	0.04055	ERAP1
ENSG00000164308	0.62585	0.236757	0.008207	0.047947	ERAP2
ENSG00000164327	1.366015	0.397515	0.00059	0.00746	RICTOR
ENSG00000164330	-1.00083	0.784155	1.71E-05	0.000526	EBF1
ENSG00000164338	1.539849	0.536922	0.004132	0.02984	UTP15
ENSG00000164379	4.346597	1.385383	0.001704	0.016121	FOXQ1
ENSG00000164405	-0.97972	0.331821	0.003152	0.024881	UQCRQ
ENSG00000164597	1.501212	0.525508	0.004281	0.030547	COG5
ENSG00000164627	3.337056	0.80573	3.45E-05	0.00091	KIF6
ENSG00000164649	-0.66469	0.248879	0.007568	0.045393	CDCA7L
ENSG00000164663	1.276364	0.305823	0.00003	0.000822	USP49
ENSG00000164687	-1.00083	0.374936	2.17E-07	1.85E-05	FABP5
ENSG00000164713	-1.00083	0.390896	0.000383	0.005513	BRI3
ENSG00000164828	0.747428	0.222619	0.000787	0.009162	SUN1
ENSG00000164889	-1.00083	0.419036	0.001461	0.014427	SLC4A2
ENSG00000164896	-0.90778	0.261902	0.000528	0.006885	FASTK
ENSG00000164919	-1.00083	0.353184	0.001315	0.013401	COX6C
ENSG00000164934	-0.71685	0.214301	0.000823	0.009461	DCAF13
ENSG00000164944	-0.98002	0.340369	0.003986	0.0291	VIRMA
ENSG00000164978	-0.87622	0.332064	0.008322	0.048394	NUDT2
ENSG00000164985	0.546726	0.194533	0.004947	0.033875	PSIP1
ENSG00000165029	0.623298	0.20907	0.00287	0.023442	ABCA1
ENSG00000165102	0.987112	0.218495	6.25E-06	0.000249	HGSNAT

ENSG00000165168	1.512167	0.464886	0.001143	0.012182	CYBB
ENSG00000165175	-0.51584	0.172021	0.002711	0.022511	MID1IP1
ENSG00000165233	-0.87971	0.335201	0.00868	0.049775	CARD19
ENSG00000165272	2.597222	0.737036	0.000425	0.005944	AQP3
ENSG00000165280	-0.5833	0.196404	0.002979	0.023988	VCP
ENSG00000165283	-0.55407	0.208147	0.007769	0.046195	STOML2
ENSG00000165288	1.473278	0.324687	5.69E-06	0.000229	BRWD3
ENSG00000165406	1.333879	0.503759	0.0081	0.047506	MARCHF8
ENSG00000165502	-1.00083	0.411326	0.005596	0.036802	RPL36AL
ENSG00000165629	-0.92291	0.2634	0.000459	0.006272	ATP5F1C
ENSG00000165685	3.085654	0.880016	0.000454	0.006231	TMEM52B
ENSG00000165688	-0.50597	0.177912	0.004456	0.031371	PMPCA
ENSG00000165752	-1.00083	0.357287	0.003929	0.028852	STK32C
ENSG00000165775	-0.88262	0.161127	4.31E-08	5.7E-06	FUNDC2
ENSG00000165795	1.945267	0.562154	0.000539	0.007	NDRG2
ENSG00000165916	-1.00083	0.254621	1.39E-05	0.000461	PSMC3
ENSG00000165995	1.172166	0.37943	0.002006	0.018098	CACNB2
ENSG00000165996	-1.00083	0.455648	0.001356	0.013657	HACD1
ENSG00000165997	1.765099	0.502246	0.000441	0.006112	ARL5B
ENSG00000166024	-0.73033	0.226861	0.001285	0.013165	R3HCC1L
ENSG00000166037	0.527666	0.18328	0.003989	0.0291	CEP57
ENSG00000166130	-1.00083	0.307429	0.000821	0.009461	IKBIP
ENSG00000166136	-0.63291	0.186642	0.000696	0.00837	NDUFB8
ENSG00000166224	1.018089	0.308388	0.000962	0.010654	SGPL1
ENSG00000166225	1.530236	0.380701	5.83E-05	0.001341	FRS2
ENSG00000166226	-0.93798	0.190383	8.36E-07	5.24E-05	CCT2
ENSG00000166266	1.128284	0.338687	0.000864	0.00983	CUL5
ENSG00000166311	-0.84795	0.287107	0.003143	0.024868	SMPD1
ENSG00000166333	-0.71391	0.224933	0.001504	0.014771	ILK
ENSG00000166398	1.394618	0.489959	0.004422	0.031275	GARRE1
ENSG00000166415	2.914895	1.04507	0.005284	0.035457	WDR72
ENSG00000166446	1.149999	0.363844	0.001574	0.015292	CDYL2
ENSG00000166448	2.221824	0.769808	0.003899	0.028719	TMEM130
ENSG00000166473	1.846178	0.340349	5.82E-08	7.07E-06	PKD1L2
ENSG00000166479	1.39861	0.340872	4.08E-05	0.001029	TMX3
ENSG00000166557	-1.00083	0.204673	2.59E-11	9.43E-09	TMED3
ENSG00000166562	-1.00083	0.296839	7.26E-06	0.000275	SEC11C
ENSG00000166598	-1.00083	0.308736	2.19E-05	0.000639	HSP90B1
ENSG00000166681	-1.00083	0.259358	7.62E-06	0.000284	BEX3
ENSG00000166689	3.29183	0.909256	0.000294	0.004488	PLEKHA7
ENSG00000166750	1.480482	0.31566	2.73E-06	0.000127	SLFN5
ENSG00000166794	-1.00083	0.334117	3.04E-06	0.000137	PPIB
ENSG00000166797	-0.55183	0.10172	5.8E-08	7.07E-06	CIAO2A
ENSG00000166828	-1.00083	0.526029	1.76E-06	9.24E-05	SCNN1G
ENSG00000166912	0.733234	0.195597	0.000178	0.003133	MTMR10
ENSG00000166913	-0.5056	0.103275	9.8E-07	5.95E-05	YWHAB

ENSG00000166927	1.147142	0.401683	0.004292	0.030612	MS4A7
ENSG00000167004	-0.98324	0.267904	0.000242	0.003935	PDIA3
ENSG00000167080	1.775769	0.387386	4.56E-06	0.000191	B4GALNT2
ENSG00000167081	-0.57902	0.202491	0.004243	0.030428	PBX3
ENSG00000167114	-1.00083	0.2865	0.000399	0.005694	SLC27A4
ENSG00000167191	-1.00083	0.782775	1.56E-06	8.41E-05	GPRC5B
ENSG00000167202	1.27322	0.300195	2.22E-05	0.000646	TBC1D2B
ENSG00000167207	0.93272	0.307408	0.002412	0.020709	NOD2
ENSG00000167264	-0.51361	0.16653	0.002041	0.018303	DUS2
ENSG00000167280	1.079238	0.265038	4.66E-05	0.001137	ENGASE
ENSG00000167315	-0.82702	0.214584	0.000116	0.002253	ACAA2
ENSG00000167378	-0.82382	0.224741	0.000247	0.00398	IRGQ
ENSG00000167397	-0.82942	0.2281	0.000277	0.004297	VKORC1
ENSG00000167508	-1.00083	0.361299	0.00541	0.036	MVD
ENSG00000167515	-1.00083	0.311262	4.94E-05	0.001183	TRAPPC2L
ENSG00000167524	1.260819	0.160912	4.67E-15	5.26E-12	RSKR
ENSG00000167552	-1.00083	0.352181	0.003317	0.025879	TUBA1A
ENSG00000167553	-1.00083	0.195773	1.29E-07	1.28E-05	TUBA1C
ENSG00000167600	1.439273	0.415489	0.000532	0.006931	CYP2S1
ENSG00000167674	-0.81595	0.298872	0.006331	0.040223	HDGFL2
ENSG00000167705	-0.89309	0.300415	0.00295	0.0239	RILP
ENSG00000167770	-0.6909	0.213555	0.001215	0.012699	OTUB1
ENSG00000167785	1.074365	0.306937	0.000465	0.006293	ZNF558
ENSG00000167797	-1.00083	0.315344	1.16E-06	6.86E-05	CDK2AP2
ENSG00000167851	-1.00083	0.311404	0.00045	0.006206	CD300A
ENSG00000167874	1.792637	0.536886	0.000841	0.009613	TMEM88
ENSG00000167984	1.98482	0.715812	0.005557	0.036667	NLRC3
ENSG00000167986	-0.73892	0.180027	4.05E-05	0.001025	DDB1
ENSG00000168000	-1.00083	0.531021	0.000512	0.00675	BSCL2
ENSG00000168003	-0.6443	0.223674	0.00397	0.029064	SLC3A2
ENSG00000168016	1.026194	0.343741	0.002832	0.023257	TRANK1
ENSG00000168061	-0.90455	0.344641	0.008675	0.049768	SAC3D1
ENSG00000168066	1.117021	0.284367	8.56E-05	0.0018	SF1
ENSG00000168137	2.091132	0.553781	0.000159	0.002885	SETD5
ENSG00000168159	-0.75641	0.202523	0.000188	0.003258	RNF187
ENSG00000168172	1.196478	0.353691	0.000717	0.00858	HOOK3
ENSG00000168246	-0.52812	0.162691	0.00117	0.012343	UBTD2
ENSG00000168259	-1.00083	0.304563	0.000183	0.00321	DNAJC7
ENSG00000168273	-1.00083	0.475441	0.00367	0.027583	SMIM4
ENSG00000168275	-0.97321	0.280504	0.000521	0.006826	COA6
ENSG00000168297	0.672708	0.245858	0.006216	0.039693	PXK
ENSG00000168300	0.902433	0.326228	0.00567	0.03714	PCMTD1
ENSG00000168306	-1.00083	0.690117	8.81E-05	0.001832	ACOX2
ENSG00000168389	-1.00083	0.337704	0.000144	0.002675	MFSD2A
ENSG00000168411	0.992981	0.355275	0.005191	0.035052	RFWD3
ENSG00000168453	4.003981	1.169037	0.000615	0.007729	HR

ENSG00000168556	-0.64705	0.223759	0.003832	0.028327	ING2
ENSG00000168610	0.786191	0.176415	8.33E-06	0.000306	STAT3
ENSG00000168653	-1.00083	0.240972	1.18E-06	6.88E-05	NDUFS5
ENSG00000168672	-1.00083	0.673076	0.000251	0.004022	LRATD2
ENSG00000168685	5.031112	0.701678	7.49E-13	4.47E-10	IL7R
ENSG00000168769	1.35684	0.202596	2.12E-11	8.33E-09	TET2
ENSG00000168795	1.071724	0.289057	0.000209	0.00355	ZBTB5
ENSG00000168807	1.035914	0.385325	0.007179	0.043822	SNTB2
ENSG00000168894	-1.00083	0.328094	0.000655	0.00803	RNF181
ENSG00000168906	0.844286	0.316035	0.007551	0.045352	MAT2A
ENSG00000169019	-0.60094	0.157646	0.000138	0.002579	COMMD8
ENSG00000169020	-1.00083	0.37139	0.002967	0.023958	ATP5ME
ENSG00000169021	-1.00083	0.237914	1.05E-05	0.000365	UQCRFS1
ENSG00000169045	0.880925	0.198268	8.87E-06	0.00032	HNRNPH1
ENSG00000169116	3.1722	0.994124	0.001418	0.014119	PARM1
ENSG00000169189	-1.00083	0.276355	3.58E-05	0.000937	NSMCE1
ENSG00000169217	-0.74509	0.269753	0.005743	0.037485	CD2BP2
ENSG00000169223	-0.6008	0.189267	0.001502	0.014768	LMAN2
ENSG00000169251	0.595651	0.152858	9.75E-05	0.001977	NMD3
ENSG00000169288	-1.00083	0.278129	0.000174	0.003071	MRPL1
ENSG00000169403	0.86278	0.27017	0.001406	0.014046	PTAFR
ENSG00000169413	-0.98389	0.374736	0.008651	0.049713	RNASE6
ENSG00000169429	2.231089	0.579625	0.000119	0.002291	CXCL8
ENSG00000169554	0.998999	0.2349	2.11E-05	0.000624	ZEB2
ENSG00000169612	-0.58907	0.168334	0.000466	0.006294	RAMAC
ENSG00000169682	-1.00083	0.259613	5.53E-07	3.83E-05	SPNS1
ENSG00000169692	-0.90024	0.301952	0.002869	0.023442	AGPAT2
ENSG00000169718	-1.00083	0.358191	0.001596	0.015413	DUS1L
ENSG00000169727	-0.83002	0.272542	0.002323	0.020123	GPS1
ENSG00000169738	-1.00083	0.418488	0.000278	0.004301	DCXR
ENSG00000169750	-1.00083	0.590067	0.002145	0.018998	RAC3
ENSG00000169762	0.944537	0.281297	0.000786	0.009162	TAPT1
ENSG00000169764	0.774108	0.26368	0.003327	0.025924	UGP2
ENSG00000169871	0.941678	0.32305	0.003557	0.027056	TRIM56
ENSG00000169919	-1.00083	0.253673	1.52E-06	8.34E-05	GUSB
ENSG00000169967	1.225634	0.335221	0.000256	0.004056	MAP3K2
ENSG00000169976	-0.91804	0.287606	0.001413	0.014096	SF3B5
ENSG00000170043	-0.90069	0.265109	0.00068	0.008235	TRAPPC1
ENSG00000170144	-0.66937	0.254807	0.008615	0.049548	HNRNPA3
ENSG00000170160	1.613597	0.44768	0.000313	0.004711	CCDC144A
ENSG00000170310	-1.00083	0.350491	0.004114	0.029741	STX8
ENSG00000170315	-0.66202	0.231397	0.004224	0.030375	UBB
ENSG00000170323	-1.00083	0.572688	1.62E-05	0.00051	FABP4
ENSG00000170325	1.251623	0.448572	0.005267	0.035359	PRDM10
ENSG00000170340	-0.79164	0.245477	0.00126	0.01296	B3GNT2
ENSG00000170417	1.616609	0.582802	0.00554	0.036634	TMEM182

ENSG00000170430	-1.00083	0.419977	0.007253	0.044074	MGMT
ENSG00000170468	-0.5451	0.186367	0.003446	0.026533	RIOX1
ENSG00000170515	-0.56401	0.189788	0.002961	0.023929	PA2G4
ENSG00000170542	1.597925	0.430361	0.000205	0.003493	SERPINB9
ENSG00000170606	-1.00083	0.234653	8.74E-09	1.49E-06	HSPA4
ENSG00000170619	-0.73046	0.278102	0.008624	0.049583	COMMD5
ENSG00000170852	0.971768	0.346762	0.005072	0.03444	KBTBD2
ENSG00000170855	-0.86323	0.199529	1.52E-05	0.000492	TRIAP1
ENSG00000170860	-1.00083	0.299618	0.000109	0.002155	LSM3
ENSG00000170871	2.54615	0.75921	0.000797	0.009231	KIAA0232
ENSG00000170903	0.737738	0.267499	0.005817	0.037729	MSANTD4
ENSG00000170909	-0.66727	0.239833	0.005399	0.035961	OSCAR
ENSG00000170949	0.505358	0.185248	0.006372	0.040368	ZNF160
ENSG00000171049	2.29809	0.766324	0.00271	0.022511	FPR2
ENSG00000171051	1.444988	0.538881	0.00733	0.044411	FPR1
ENSG00000171094	-1.00083	0.752199	0.000652	0.008012	ALK
ENSG00000171115	2.338172	0.575871	0.000049	0.001178	GIMAP8
ENSG00000171135	-0.62154	0.231434	0.00724	0.044022	JAGN1
ENSG00000171159	-1.00083	0.281947	8.53E-05	0.001797	C9orf16
ENSG00000171202	-0.92047	0.226876	4.97E-05	0.001186	TMEM126A
ENSG00000171227	-1.00083	0.798809	0.000683	0.008265	TMEM37
ENSG00000171310	0.721594	0.243675	0.003063	0.02441	CHST11
ENSG00000171453	-0.61901	0.179276	0.000555	0.007112	POLR1C
ENSG00000171469	0.884703	0.321497	0.005926	0.038296	ZNF561
ENSG00000171488	1.83474	0.425137	1.59E-05	0.000506	LRRC8C
ENSG00000171492	0.852401	0.157326	6.03E-08	7.19E-06	LRRC8D
ENSG00000171530	-0.75816	0.281393	0.007053	0.04321	TBCA
ENSG00000171659	-0.64194	0.237618	0.006901	0.042581	GPR34
ENSG00000171823	1.041889	0.265448	8.67E-05	0.001818	FBXL14
ENSG00000171861	-0.69195	0.189887	0.000268	0.004184	MRM3
ENSG00000171940	1.103074	0.235651	2.86E-06	0.000131	ZNF217
ENSG00000171943	0.757321	0.273349	0.005597	0.036802	SRGAP2C
ENSG00000172009	-0.85208	0.234352	0.000277	0.004297	THOP1
ENSG00000172037	-1.00083	0.562315	0.007423	0.044835	LAMB2
ENSG00000172175	0.969814	0.239642	5.19E-05	0.00122	MALT1
ENSG00000172236	-1.00083	0.656973	0.005951	0.038396	TPSAB1
ENSG00000172270	-0.73252	0.23313	0.001677	0.015948	BSG
ENSG00000172301	-1.00083	0.324673	4.55E-05	0.00112	COPRS
ENSG00000172322	0.910061	0.341786	0.007752	0.046115	CLEC12A
ENSG00000172354	-1.00083	0.36154	0.004227	0.030382	GNB2
ENSG00000172366	-1.00083	0.446845	0.000504	0.006681	MCRIP2
ENSG00000172466	0.701808	0.23896	0.003315	0.025879	ZNF24
ENSG00000172469	0.644705	0.206231	0.001771	0.016539	MANEA
ENSG00000172531	-0.96891	0.256454	0.000158	0.002874	PPP1CA
ENSG00000172575	2.430574	0.823188	0.003151	0.024881	RASGRP1
ENSG00000172590	-1.00083	0.366035	0.002064	0.018452	MRPL52

ENSG00000172724	4.607091	1.258807	0.000252	0.004022	CCL19
ENSG00000172757	-0.55297	0.101476	5.06E-08	6.31E-06	CFL1
ENSG00000172780	0.875736	0.299052	0.003407	0.026385	RAB43
ENSG00000172785	-0.85844	0.219758	9.37E-05	0.001922	CBWD1
ENSG00000172794	1.953827	0.66131	0.003132	0.024812	RAB37
ENSG00000172840	1.080568	0.33325	0.001185	0.012455	PDP2
ENSG00000172889	-1.00083	0.431218	0.000318	0.004744	EGFL7
ENSG00000172922	-1.00083	0.334241	0.000288	0.004406	RNASEH2C
ENSG00000172943	0.787621	0.287077	0.006077	0.03908	PHF8
ENSG00000173083	-1.00083	0.810863	0.004236	0.030428	HPSE
ENSG00000173113	-0.88203	0.294782	0.00277	0.02287	TRMT112
ENSG00000173120	0.503345	0.159537	0.001605	0.015472	KDM2A
ENSG00000173163	-1.00083	0.363303	0.004163	0.029986	COMMD1
ENSG00000173171	-0.95865	0.230889	0.000033	0.000882	MTX1
ENSG00000173198	1.001334	0.354412	0.004723	0.032733	CYSLTR1
ENSG00000173221	-0.98452	0.263614	0.000188	0.003258	GLRX
ENSG00000173227	2.460928	0.907581	0.006697	0.041776	SYT12
ENSG00000173391	3.204718	0.631927	3.95E-07	2.92E-05	OLR1
ENSG00000173436	-1.00083	0.451797	0.004402	0.031168	MICOS10
ENSG00000173457	-1.00083	0.237773	1.81E-06	0.000094	PPP1R14B
ENSG00000173486	-1.00083	0.353587	2.01E-06	0.000103	FKBP2
ENSG00000173511	-1.00083	0.404754	5.06E-05	0.001204	VEGFB
ENSG00000173517	0.971348	0.361542	0.007217	0.043917	PEAK1
ENSG00000173542	0.966614	0.266706	0.00029	0.004435	MOB1B
ENSG00000173545	-1.00083	0.211485	1.06E-08	1.67E-06	ZNF622
ENSG00000173578	-1.00083	0.917792	0.000691	0.008324	XCR1
ENSG00000173599	-0.90997	0.262172	0.000519	0.006808	PC
ENSG00000173660	-0.78483	0.237557	0.000954	0.010618	UQCRH
ENSG00000173692	-1.00083	0.276432	5.98E-05	0.001371	PSMD1
ENSG00000173757	0.789327	0.225327	0.00046	0.006272	STAT5B
ENSG00000173812	-1.00083	0.355812	0.002989	0.024039	EIF1
ENSG00000173821	1.113383	0.25033	8.68E-06	0.000317	RNF213
ENSG00000173889	0.853357	0.262799	0.001166	0.012318	PHC3
ENSG00000173890	1.195204	0.439455	0.006533	0.040941	GPR160
ENSG00000173915	-1.00083	0.307502	0.000598	0.007551	ATP5MK
ENSG00000174010	1.11661	0.196716	1.38E-08	2.1E-06	KLHL15
ENSG00000174021	-0.82313	0.143289	9.22E-09	1.52E-06	GNG5
ENSG00000174130	1.025065	0.279772	0.000248	0.003998	TLR6
ENSG00000174197	0.907359	0.285557	0.001485	0.014651	MGA
ENSG00000174233	0.821485	0.273035	0.002624	0.022003	ADCY6
ENSG00000174353	0.844517	0.211987	6.78E-05	0.001519	STAG3L3
ENSG00000174405	0.770849	0.278226	0.005596	0.036802	LIG4
ENSG00000174547	-1.00083	0.342975	0.000285	0.004382	MRPL11
ENSG00000174579	0.606086	0.177322	0.000631	0.007845	MSL2
ENSG00000174606	1.186925	0.25835	4.34E-06	0.000183	ANGEL2
ENSG00000174695	-0.59809	0.17989	0.000885	0.009975	TMEM167A

ENSG00000174697	-1.00083	0.783571	4.91E-05	0.001178	LEP
ENSG00000174738	0.78777	0.231478	0.000666	0.008113	NR1D2
ENSG00000174744	-1.00083	0.32931	0.000771	0.009055	BRMS1
ENSG00000174749	1.075704	0.206818	1.98E-07	1.72E-05	FAM241A
ENSG00000174775	-1.00083	0.319888	9.41E-05	0.001922	HRAS
ENSG00000174796	1.10279	0.405202	0.006497	0.040785	THAP6
ENSG00000174799	0.880984	0.333222	0.008197	0.04791	CEP135
ENSG00000174807	-1.00083	0.645983	6.85E-06	0.000263	CD248
ENSG00000174886	-1.00083	0.306812	0.00067	0.008142	NDUFA11
ENSG00000174903	-0.53326	0.145856	0.000256	0.004056	RAB1B
ENSG00000174939	-1.00083	0.409739	0.000675	0.008184	ASPHD1
ENSG00000174946	5.277747	1.272112	3.34E-05	0.000889	GPR171
ENSG00000175040	0.920196	0.34242	0.007203	0.04389	CHST2
ENSG00000175105	0.763088	0.282366	0.006883	0.042487	ZNF654
ENSG00000175155	0.861408	0.288481	0.002826	0.023236	YPEL2
ENSG00000175197	-1.00083	0.300339	3.12E-12	1.43E-09	DDIT3
ENSG00000175198	-1.00083	0.324062	4.53E-05	0.001116	PCCA
ENSG00000175215	1.360804	0.323121	2.54E-05	0.000721	CTDSP2
ENSG00000175216	1.131054	0.426489	0.008001	0.047126	CKAP5
ENSG00000175262	-1.00083	0.898098	0.001418	0.014119	C1orf127
ENSG00000175265	1.236961	0.291061	2.14E-05	0.000629	GOLGA8A
ENSG00000175376	-1.00083	0.345447	0.001075	0.011634	EIF1AD
ENSG00000175390	0.758198	0.221606	0.000623	0.007783	EIF3F
ENSG00000175416	-0.86002	0.322962	0.007747	0.046102	CLTB
ENSG00000175467	-1.00083	0.445979	0.001733	0.016296	SART1
ENSG00000175471	0.90626	0.287816	0.00164	0.015709	MCTP1
ENSG00000175595	1.150334	0.308252	0.00019	0.003291	ERCC4
ENSG00000175727	1.016822	0.378976	0.007295	0.044256	MLXIP
ENSG00000175756	-1.00083	0.329265	0.000175	0.003079	AURKAIP1
ENSG00000175768	-0.88528	0.24387	0.000283	0.004355	TOMM5
ENSG00000175787	1.73672	0.496117	0.000464	0.006293	ZNF169
ENSG00000175893	2.171384	0.622371	0.000485	0.006491	ZDHHC21
ENSG00000175906	-1.00083	0.604833	6.54E-07	0.000044	ARL4D
ENSG00000176014	-0.74154	0.155508	1.86E-06	9.62E-05	TUBB6
ENSG00000176018	0.612287	0.231653	0.008215	0.04797	LYSMD3
ENSG00000176046	-1.00083	0.753922	0.005176	0.035009	NUPR1
ENSG00000176142	1.292714	0.403412	0.001353	0.013657	TMEM39A
ENSG00000176170	-0.73192	0.194072	0.000162	0.002925	SPHK1
ENSG00000176244	2.174309	0.695261	0.001764	0.01652	ACBD7
ENSG00000176261	-1.00083	0.296401	0.000485	0.006491	ZBTB8OS
ENSG00000176340	-0.67929	0.233821	0.003671	0.027583	COX8A
ENSG00000176386	-1.00083	0.272722	0.000163	0.002933	CDC26
ENSG00000176809	1.099853	0.347527	0.001552	0.015121	LRRC37A3
ENSG00000176853	0.782555	0.291117	0.007186	0.043822	FAM91A1
ENSG00000176973	-0.69277	0.195268	0.000388	0.005573	FAM89B
ENSG00000176978	-1.00083	0.456942	0.00168	0.015958	DPP7

ENSG00000177000	0.600191	0.17533	0.000619	0.007759	MTHFR
ENSG00000177025	-1.00083	0.621661	0.002961	0.023929	C19orf18
ENSG00000177058	0.858368	0.249625	0.000585	0.007405	SLC38A9
ENSG00000177156	-1.00083	0.280388	6.78E-06	0.000261	TALDO1
ENSG00000177191	-1.00083	0.644123	7.11E-05	0.001573	B3GNT8
ENSG00000177303	1.012859	0.365226	0.00555	0.036651	CASKIN2
ENSG00000177383	1.115738	0.407178	0.006141	0.039331	MAGEF1
ENSG00000177409	1.237352	0.287142	1.64E-05	0.000512	SAMD9L
ENSG00000177463	1.471847	0.417536	0.000423	0.005942	NR2C2
ENSG00000177494	7.461837	1.430403	1.82E-07	0.000016	ZBED2
ENSG00000177575	-1.00083	0.604078	0.008484	0.049084	CD163
ENSG00000177600	-1.00083	0.457363	0.004397	0.031163	RPLP2
ENSG00000177628	-0.75797	0.149569	4.03E-07	2.93E-05	GBA
ENSG00000177697	-0.96161	0.183017	1.49E-07	1.37E-05	CD151
ENSG00000177700	-0.99607	0.34547	0.003936	0.028883	POLR2L
ENSG00000177731	-1.00083	0.340639	0.000155	0.002841	FLII
ENSG00000177853	0.935649	0.342873	0.006356	0.040307	ZNF518A
ENSG00000177879	-0.6277	0.180755	0.000515	0.006787	AP3S1
ENSG00000177932	1.317173	0.320565	3.98E-05	0.001013	ZNF354C
ENSG00000177981	0.740475	0.278945	0.007941	0.046903	ASB8
ENSG00000177984	2.144578	0.673762	0.001458	0.014418	LCN15
ENSG00000178295	2.245245	0.600562	0.000185	0.003237	GEN1
ENSG00000178449	-0.85322	0.301198	0.004615	0.032228	COX14
ENSG00000178605	-0.74973	0.280053	0.007426	0.044835	GTPBP6
ENSG00000178741	-0.81958	0.22555	0.000279	0.004307	COX5A
ENSG00000178772	2.232642	0.654788	0.00065	0.008	CPN2
ENSG00000178896	-1.00083	0.325449	0.000849	0.009702	EXOSC4
ENSG00000178952	-0.76548	0.213107	0.000328	0.004881	TUFM
ENSG00000178966	0.971006	0.325583	0.00286	0.023403	RMI1
ENSG00000178982	-1.00083	0.334713	0.001544	0.015055	EIF3K
ENSG00000179104	1.54695	0.350155	9.97E-06	0.000351	TMTC2
ENSG00000179144	1.448099	0.549639	0.008423	0.048833	GIMAP7
ENSG00000179163	-1.00083	0.560869	0.002697	0.022451	FUCA1
ENSG00000179218	-1.00083	0.151007	4.81E-18	1.65E-14	CALR
ENSG00000179271	-1.00083	0.348046	0.000241	0.003922	GADD45GIP1
ENSG00000179361	0.898508	0.223246	0.000057	0.001314	ARID3B
ENSG00000179454	1.805844	0.52278	0.000552	0.007103	KLHL28
ENSG00000179526	-0.67563	0.231444	0.003509	0.026767	SHARPIN
ENSG00000179532	0.893446	0.320509	0.00531	0.035531	DNHD1
ENSG00000179912	1.225387	0.337176	0.000279	0.004307	R3HDM2
ENSG00000179918	-0.94254	0.247891	0.000143	0.002668	SEPHS2
ENSG00000179941	0.796762	0.267624	0.002909	0.023662	BBS10
ENSG00000180357	0.928152	0.305478	0.002379	0.020501	ZNF609
ENSG00000180488	0.902999	0.276639	0.001098	0.011821	MIGA1
ENSG00000180530	1.034455	0.363456	0.004425	0.031282	NRIP1
ENSG00000180879	-1.00083	0.392764	0.00056	0.007174	SSR4

ENSG00000181004	0.925331	0.306816	0.002562	0.021664	BBS12
ENSG00000181019	-1.00083	0.300803	2.51E-07	2.11E-05	NQO1
ENSG00000181036	2.57899	0.508933	4.03E-07	2.93E-05	FCRL6
ENSG00000181045	-0.86004	0.30659	0.005029	0.034212	SLC26A11
ENSG00000181192	1.119226	0.363765	0.002093	0.018621	DHTKD1
ENSG00000181284	-0.51924	0.196555	0.008249	0.04807	TMEM102
ENSG00000181350	-1.00083	0.423744	0.007661	0.045773	LRRC75A
ENSG00000181467	-0.95288	0.248523	0.000126	0.002406	RAP2B
ENSG00000181481	-0.76566	0.225255	0.000676	0.008193	RNF135
ENSG00000181555	0.770649	0.254807	0.002491	0.021189	SETD2
ENSG00000181577	1.780644	0.401063	0.000009	0.000323	C6orf223
ENSG00000181785	1.659212	0.471574	0.000434	0.006044	OR5AS1
ENSG00000181789	-0.99788	0.20055	6.5E-07	0.000044	COPG1
ENSG00000181830	-0.54373	0.14057	0.00011	0.002161	SLC35C1
ENSG00000182004	-1.00083	0.457566	0.000696	0.00837	SNRPE
ENSG00000182010	2.053582	0.763904	0.007182	0.043822	RTKN2
ENSG00000182108	-1.00083	0.247003	6.37E-09	1.14E-06	DEXI
ENSG00000182117	-1.00083	0.287403	2.44E-05	0.000702	NOP10
ENSG00000182149	0.558178	0.159404	0.000462	0.006292	IST1
ENSG00000182196	-1.00083	0.415213	0.002073	0.018514	ARL6IP4
ENSG00000182224	1.103761	0.308041	0.000339	0.004995	CYB5D1
ENSG00000182372	0.762424	0.216107	0.000419	0.005902	CLN8
ENSG00000182378	-0.87828	0.300112	0.003428	0.026454	PLCXD1
ENSG00000182544	-0.52299	0.178887	0.00346	0.026612	MFSD5
ENSG00000182568	1.23694	0.436655	0.004615	0.032228	SATB1
ENSG00000182718	-1.00083	0.149517	5.64E-16	8.6E-13	ANXA2
ENSG00000182768	-0.99906	0.253317	8.02E-05	0.001722	NGRN
ENSG00000182782	3.39812	0.866013	8.71E-05	0.001823	HCAR2
ENSG00000182796	0.737053	0.231075	0.001424	0.014171	TMEM198B
ENSG00000182866	3.57631	0.974029	0.000241	0.003922	LCK
ENSG00000182952	0.787536	0.187452	2.65E-05	0.000748	HMGN4
ENSG00000182963	1.576468	0.544018	0.003758	0.027952	GJC1
ENSG00000183023	2.093358	0.517503	5.23E-05	0.001223	SLC8A1
ENSG00000183049	1.045538	0.269794	0.000106	0.002119	CAMK1D
ENSG00000183091	1.009148	0.312714	0.001251	0.012929	NEB
ENSG00000183258	-1.00083	0.341824	0.002218	0.019428	DDX41
ENSG00000183260	-1.00083	0.274319	8.93E-06	0.000321	ABHD16B
ENSG00000183337	0.93772	0.229614	4.43E-05	0.0011	BCOR
ENSG00000183486	0.894611	0.152482	4.44E-09	8.35E-07	MX2
ENSG00000183508	2.980015	0.923501	0.001252	0.012929	TENT5C
ENSG00000183605	-0.62764	0.214867	0.003488	0.026696	SFXN4
ENSG00000183617	-1.00083	0.492529	0.007831	0.046481	MRPL54
ENSG00000183621	-0.86506	0.17857	1.27E-06	7.27E-05	ZNF438
ENSG00000183684	-1.00083	0.277095	6.82E-05	0.00152	ALYREF
ENSG00000183741	1.391421	0.351545	7.56E-05	0.001635	CBX6
ENSG00000183751	-1.00083	0.523423	0.006458	0.040586	TBL3

ENSG00000183762	3.944386	0.722261	4.73E-08	5.96E-06	KREMEN1
ENSG00000183779	-1.00083	0.322814	4.04E-05	0.001024	ZNF703
ENSG00000183808	1.898342	0.422133	6.89E-06	0.000264	RBM12B
ENSG00000183813	3.492279	0.925688	0.000162	0.002918	CCR4
ENSG00000183918	6.08752	1.195518	3.54E-07	2.67E-05	SH2D1A
ENSG00000184007	-0.69067	0.209971	0.001004	0.011035	PTP4A2
ENSG00000184014	0.82763	0.289924	0.004308	0.030645	DENND5A
ENSG00000184047	-0.83056	0.294271	0.004766	0.032965	DIABLO
ENSG00000184056	-1.00083	0.299738	0.000491	0.006554	VPS33B
ENSG00000184076	-0.735	0.259914	0.004686	0.032527	UQCR10
ENSG00000184110	-1.00083	0.423436	0.000045	0.001114	EIF3C
ENSG00000184164	-1.00083	0.317005	0.000214	0.003617	CRELD2
ENSG00000184182	-0.63226	0.221134	0.004248	0.030428	UBE2F
ENSG00000184207	-0.8719	0.327777	0.007813	0.046396	PGP
ENSG00000184209	-0.84362	0.302694	0.005319	0.035572	SNRNP35
ENSG00000184216	-0.83004	0.188389	1.05E-05	0.000366	IRAK1
ENSG00000184292	3.422104	0.891227	0.000123	0.002361	TACSTD2
ENSG00000184319	-1.00083	0.385389	0.002604	0.021897	RPL23AP82
ENSG00000184381	1.540874	0.253193	1.16E-09	2.68E-07	PLA2G6
ENSG00000184470	-0.81476	0.295211	0.005781	0.037582	TXNRD2
ENSG00000184584	1.510667	0.428682	0.000425	0.005944	STING1
ENSG00000184588	2.794144	0.592912	2.45E-06	0.000117	PDE4B
ENSG00000184634	0.911915	0.197985	4.11E-06	0.000177	MED12
ENSG00000184730	-0.74302	0.162705	4.96E-06	0.000204	APOBR
ENSG00000184785	-1.00083	0.572823	0.000119	0.002291	SMIM10
ENSG00000184840	-0.84003	0.251275	0.000829	0.009503	TMED9
ENSG00000184857	-0.63098	0.175793	0.000332	0.004921	TMEM186
ENSG00000184863	0.897717	0.324366	0.005647	0.037059	RBM33
ENSG00000184887	-0.66143	0.193803	0.000643	0.007937	BTBD6
ENSG00000184924	-1.00083	0.311507	0.000553	0.007103	PTRHD1
ENSG00000184983	-0.9769	0.277626	0.000434	0.006043	NDUFA6
ENSG00000184984	2.096306	0.697599	0.002656	0.022203	CHRM5
ENSG00000185088	-1.00083	0.202138	9.87E-08	1.05E-05	RPS27L
ENSG00000185164	-1.00083	0.331896	1.71E-06	9.01E-05	NOMO2
ENSG00000185219	0.828876	0.268876	0.002051	0.018356	ZNF445
ENSG00000185245	2.497593	0.943824	0.008139	0.047665	GP1BA
ENSG00000185261	1.892925	0.562297	0.000762	0.008951	KIAA0825
ENSG00000185291	3.197044	0.957069	0.000836	0.009577	IL3RA
ENSG00000185344	0.724711	0.211466	0.00061	0.007685	ATP6V0A2
ENSG00000185591	0.861657	0.295933	0.003595	0.027187	SP1
ENSG00000185608	-1.00083	0.311632	1.55E-05	0.000498	MRPL40
ENSG00000185621	1.099553	0.399155	0.005875	0.03799	LMLN
ENSG00000185624	-0.86337	0.240093	0.000323	0.004823	P4HB
ENSG00000185627	-1.00083	0.339913	0.000343	0.005032	PSMD13
ENSG00000185651	-0.64158	0.204682	0.001721	0.01621	UBE2L3
ENSG00000185666	1.37911	0.438653	0.001667	0.01588	SYN3

ENSG00000185710	3.228455	0.843019	0.000128	0.002434	SMG1P4
ENSG00000185721	-0.86918	0.289195	0.002651	0.022191	DRG1
ENSG00000185813	-1.00083	0.37113	0.004642	0.032341	PCYT2
ENSG00000185825	-1.00083	0.184453	1.17E-09	2.68E-07	BCAP31
ENSG00000185883	-0.97443	0.185041	1.39E-07	1.34E-05	ATP6V0C
ENSG00000185896	-0.79049	0.16348	1.33E-06	7.45E-05	LAMP1
ENSG00000185900	0.685402	0.250905	0.006301	0.040121	POMK
ENSG00000185909	-1.00083	0.485368	2.55E-06	0.00012	KLHDC8B
ENSG00000185946	2.023385	0.522438	0.000108	0.00213	RNPC3
ENSG00000185947	0.589104	0.20249	0.003622	0.02734	ZNF267
ENSG00000186001	0.89579	0.279239	0.001337	0.013524	LRCH3
ENSG00000186010	-1.00083	0.373463	0.000563	0.007189	NDUFA13
ENSG00000186047	1.467004	0.464537	0.001589	0.015391	DLEU7
ENSG00000186088	0.944115	0.212373	8.77E-06	0.000318	GSAP
ENSG00000186185	2.348511	0.686273	0.000621	0.007769	KIF18B
ENSG00000186318	1.422966	0.475721	0.002779	0.022929	BACE1
ENSG00000186395	-1.00083	0.366638	3.45E-06	0.000153	KRT10
ENSG00000186399	1.787667	0.66869	0.007509	0.045177	GOLGA8R
ENSG00000186451	-1.00083	0.6734	8.51E-05	0.001794	SPATA12
ENSG00000186487	2.022679	0.602812	0.000792	0.009197	MYT1L
ENSG00000186517	-0.5032	0.172143	0.003465	0.026637	ARHGAP30
ENSG00000186522	1.285806	0.408519	0.001647	0.015741	SEPTIN10
ENSG00000186529	2.379967	0.769291	0.001977	0.017924	CYP4F3
ENSG00000186566	0.848115	0.298081	0.004438	0.031356	GPATCH8
ENSG00000186638	1.394383	0.50245	0.005517	0.036522	KIF24
ENSG00000186665	2.529817	0.550403	4.3E-06	0.000182	C17orf58
ENSG00000186812	0.814714	0.297207	0.006121	0.039267	ZNF397
ENSG00000186814	1.059497	0.264388	6.14E-05	0.001398	ZSCAN30
ENSG00000186854	0.930214	0.344441	0.00692	0.042625	TRABD2A
ENSG00000186919	-1.00083	0.830902	0.003978	0.029068	ZACN
ENSG00000187024	-1.00083	0.391025	0.005663	0.03714	PTRH1
ENSG00000187051	-1.00083	0.337564	3.39E-05	0.000898	RPS19BP1
ENSG00000187118	-0.53674	0.156918	0.000625	0.007793	CMC1
ENSG00000187187	1.14535	0.328491	0.000489	0.006537	ZNF546
ENSG00000187189	0.802045	0.215003	0.000191	0.003302	TSPYL4
ENSG00000187605	0.986026	0.207756	2.07E-06	0.000105	TET3
ENSG00000187688	-1.00083	0.238818	6.7E-08	7.77E-06	TRPV2
ENSG00000187742	0.586935	0.197109	0.002904	0.023633	SECISBP2
ENSG00000187764	2.077306	0.483642	1.75E-05	0.000534	SEMA4D
ENSG00000187837	-0.90997	0.290256	0.001718	0.016195	H1-2
ENSG00000187840	-1.00083	0.382965	0.007945	0.046903	EIF4EBP1
ENSG00000188060	-1.00083	0.246397	1.39E-05	0.000461	RAB42
ENSG00000188070	0.890018	0.278512	0.001395	0.013962	C11orf95
ENSG00000188167	1.274095	0.468724	0.006563	0.041071	TMPPE
ENSG00000188186	-1.00083	0.482639	0.000583	0.007388	LAMTOR4
ENSG00000188229	-1.00083	0.233199	2.27E-09	4.79E-07	TUBB4B

ENSG00000188282	5.075554	1.065242	1.89E-06	9.76E-05	RUFY4
ENSG00000188313	1.568264	0.430051	0.000266	0.004167	PLSCR1
ENSG00000188342	-0.69746	0.214934	0.001175	0.012375	GTF2F2
ENSG00000188404	4.226411	0.532247	2.01E-15	2.76E-12	SELL
ENSG00000188483	-1.00083	0.426903	0.002969	0.023958	IER5L
ENSG00000188554	0.589676	0.156103	0.000158	0.002877	NBR1
ENSG00000188566	0.581609	0.179819	0.001219	0.012726	NDOR1
ENSG00000188596	1.449954	0.465208	0.001828	0.016934	CFAP54
ENSG00000188603	-0.97118	0.263239	0.000225	0.003746	CLN3
ENSG00000188641	0.998576	0.326782	0.002245	0.019616	DPYD
ENSG00000188906	1.379778	0.293407	2.57E-06	0.000121	LRRK2
ENSG00000188931	1.632314	0.605558	0.007027	0.043089	CFAP126
ENSG00000188976	-0.84927	0.228672	0.000204	0.003484	NOC2L
ENSG00000188986	-0.6289	0.230553	0.006376	0.040368	NELFB
ENSG00000189043	-0.51069	0.151506	0.00075	0.008863	NDUFA4
ENSG00000189136	1.348647	0.4923	0.006154	0.039367	UBE2Q2P1
ENSG00000189423	1.92443	0.551051	0.000479	0.006427	USP32P3
ENSG00000196072	-1.00083	0.257868	5.95E-06	0.000238	BLOC1S2
ENSG00000196139	-1.00083	0.643534	9.69E-05	0.001971	AKR1C3
ENSG00000196154	-1.00083	0.39482	2.16E-06	0.000109	S100A4
ENSG00000196177	1.152905	0.287257	5.98E-05	0.001371	ACADSB
ENSG00000196182	-0.55466	0.189942	0.003498	0.026756	STK40
ENSG00000196209	1.101342	0.361951	0.002344	0.020252	SIRPB2
ENSG00000196262	-0.89195	0.189044	2.38E-06	0.000115	PPIA
ENSG00000196323	1.419379	0.53576	0.008066	0.047352	ZBTB44
ENSG00000196345	2.100668	0.732291	0.004123	0.029789	ZKSCAN7
ENSG00000196365	-1.00083	0.168638	3.77E-10	9.57E-08	LONP1
ENSG00000196419	-0.68719	0.1535	7.58E-06	0.000283	XRCC6
ENSG00000196547	0.987827	0.247992	0.000068	0.00152	MAN2A2
ENSG00000196628	1.429488	0.344475	3.33E-05	0.000887	TCF4
ENSG00000196704	-0.66821	0.225594	0.003056	0.024368	AMZ2
ENSG00000196712	1.361381	0.468275	0.003646	0.027477	NF1
ENSG00000196754	-1.00083	0.626797	0.000854	0.009735	S100A2
ENSG00000196914	0.875445	0.299871	0.003507	0.026763	ARHGEF12
ENSG00000196924	-0.89756	0.1689	1.07E-07	1.13E-05	FLNA
ENSG00000197037	1.218271	0.237801	3.01E-07	2.42E-05	ZSCAN25
ENSG00000197043	-1.00083	0.384356	0.000112	0.002198	ANXA6
ENSG00000197044	1.509807	0.433677	0.000499	0.006616	ZNF441
ENSG00000197054	0.650108	0.2101	0.001973	0.017902	ZNF763
ENSG00000197121	1.173487	0.4191	0.00511	0.034679	PGAP1
ENSG00000197147	1.517497	0.465223	0.001107	0.011881	LRRC8B
ENSG00000197157	-0.72028	0.205622	0.00046	0.006272	SND1
ENSG00000197183	0.775773	0.286694	0.006811	0.042206	NOL4L
ENSG00000197217	1.551907	0.337029	4.13E-06	0.000177	ENTPD4
ENSG00000197321	1.323285	0.471492	0.005007	0.034128	SVIL
ENSG00000197343	0.764167	0.204534	0.000187	0.003254	ZNF655

ENSG00000197375	1.461859	0.519188	0.004868	0.033514	SLC22A5
ENSG00000197405	-0.8793	0.28271	0.001869	0.017181	C5AR1
ENSG00000197646	2.047639	0.433566	2.33E-06	0.000112	PDCD1LG2
ENSG00000197696	-1.00083	0.276772	6.57E-06	0.000255	NMB
ENSG00000197747	-1.00083	0.224998	2.95E-06	0.000134	S100A10
ENSG00000197753	1.876118	0.408947	4.48E-06	0.000188	LHFPL5
ENSG00000197766	-1.00083	0.564635	0.000933	0.010435	CFD
ENSG00000197769	2.871767	0.984105	0.003521	0.026831	MAP1LC3C
ENSG00000197894	-0.93113	0.20984	9.11E-06	0.000326	ADH5
ENSG00000197943	0.608006	0.231605	0.00866	0.049727	PLCG2
ENSG00000197956	-1.00083	0.317012	3.61E-07	2.71E-05	S100A6
ENSG00000197982	-1.00083	0.347256	0.001959	0.017809	C1orf122
ENSG00000198001	1.304687	0.248248	1.48E-07	1.37E-05	IRAK4
ENSG00000198081	0.655587	0.236592	0.005589	0.036802	ZBTB14
ENSG00000198160	0.699142	0.222099	0.001645	0.015741	MIER1
ENSG00000198162	1.495447	0.492502	0.002394	0.020581	MAN1A2
ENSG00000198171	-1.00083	0.288218	0.000241	0.003922	DDRGK1
ENSG00000198208	-1.00083	0.423577	0.007566	0.045393	RPS6KL1
ENSG00000198246	-0.84169	0.251705	0.000826	0.009489	SLC29A3
ENSG00000198258	-0.85632	0.279548	0.00219	0.019295	UBL5
ENSG00000198265	0.719079	0.26619	0.006905	0.042588	HELZ
ENSG00000198315	1.241082	0.316644	8.87E-05	0.00184	ZKSCAN8
ENSG00000198324	-0.81558	0.277353	0.003276	0.02573	PHETA1
ENSG00000198355	0.724545	0.195266	0.000207	0.003518	PIM3
ENSG00000198356	-0.67005	0.221053	0.002436	0.020863	GET3
ENSG00000198382	1.084947	0.237331	4.84E-06	0.000201	UVRAG
ENSG00000198408	0.640792	0.209639	0.002238	0.019573	OGA
ENSG00000198464	-1.00083	0.453288	0.000778	0.009093	ZNF480
ENSG00000198466	0.966895	0.365529	0.008164	0.047779	ZNF587
ENSG00000198522	-0.53991	0.147513	0.000252	0.004022	GPN1
ENSG00000198546	-1.00083	0.366981	0.000314	0.004717	ZNF511
ENSG00000198561	1.169691	0.308911	0.000153	0.002804	CTNND1
ENSG00000198625	0.906972	0.317157	0.00424	0.030428	MDM4
ENSG00000198690	0.822107	0.204981	6.06E-05	0.001383	FAN1
ENSG00000198692	-0.90133	0.322971	0.005259	0.035359	EIF1AY
ENSG00000198712	-0.52797	0.195203	0.006836	0.042316	MT-CO2
ENSG00000198715	-1.00083	0.520641	0.004535	0.031817	GLMP
ENSG00000198718	1.042639	0.351944	0.003051	0.024356	TOGARAM1
ENSG00000198721	-0.62911	0.172636	0.000268	0.004184	ECI2
ENSG00000198740	-1.00083	0.420481	3.93E-05	0.001007	ZNF652
ENSG00000198795	-1.00083	1.058002	0.00686	0.042381	ZNF521
ENSG00000198805	-1.00083	0.218844	2.28E-06	0.000111	PNP
ENSG00000198814	1.258511	0.304681	3.62E-05	0.000944	GK
ENSG00000198829	1.134306	0.425929	0.007742	0.046091	SUCNR1
ENSG00000198840	-0.73138	0.259708	0.00486	0.033478	MT-ND3
ENSG00000198851	4.175257	1.051812	0.000072	0.001584	CD3E

ENSG00000198862	1.144581	0.350633	0.001097	0.011821	LTN1
ENSG00000198865	-1.00083	1.036808	0.000707	0.008479	CCDC152
ENSG00000198937	-1.00083	0.682792	0.006408	0.040505	CCDC167
ENSG00000198945	0.917905	0.280841	0.001082	0.011685	L3MBTL3
ENSG00000198959	1.562414	0.544233	0.004094	0.029642	TGM2
ENSG00000198960	-0.84751	0.215124	8.16E-05	0.001745	ARMCX6
ENSG00000203668	1.467633	0.407525	0.000317	0.004735	CHML
ENSG00000203710	0.932206	0.285105	0.001077	0.011648	CR1
ENSG00000203782	2.970579	1.080002	0.00595	0.038396	LORICRIN
ENSG00000203805	-1.00083	0.854619	6.28E-06	0.000249	PLPP4
ENSG00000203880	0.773583	0.261251	0.003066	0.024413	PCMTD2
ENSG00000204160	0.731557	0.232436	0.001648	0.015741	ZDHHC18
ENSG00000204177	1.841782	0.545659	0.000737	0.008741	BMS1P1
ENSG00000204217	1.100836	0.33776	0.001117	0.011973	BMPR2
ENSG00000204220	-1.00083	0.321786	0.001317	0.013407	PFDN6
ENSG00000204257	1.742132	0.302273	8.24E-09	1.43E-06	HLA-DMA
ENSG00000204356	-1.00083	0.451292	0.005175	0.035009	NELFE
ENSG00000204531	2.624398	0.990455	0.008057	0.04733	POU5F1
ENSG00000204568	-0.66436	0.156011	2.06E-05	0.000611	MRPS18B
ENSG00000204569	2.548361	0.886227	0.004034	0.0293	PPP1R10
ENSG00000204623	1.350876	0.262166	2.57E-07	2.14E-05	ZNRD1ASP
ENSG00000204681	1.76429	0.240505	2.2E-13	1.57E-10	GABBR1
ENSG00000204967	1.661831	0.5381	0.002013	0.018121	PCDHA4
ENSG00000205084	2.683177	0.730064	0.000238	0.003907	TMEM231
ENSG00000205323	-1.00083	0.300621	0.000565	0.007206	SARNP
ENSG00000205352	-0.81814	0.236396	0.000538	0.006993	PRR13
ENSG00000205629	-0.94372	0.278003	0.000687	0.008283	LCMT1
ENSG00000205746	2.708376	0.647563	2.88E-05	0.000801	PKD1P4
ENSG00000205846	4.939179	1.274956	0.000107	0.002125	CLEC6A
ENSG00000206052	2.219903	0.762409	0.003595	0.027187	DOK6
ENSG00000206149	1.625409	0.453221	0.000335	0.004956	HERC2P9
ENSG00000211455	0.972316	0.276059	0.000428	0.005973	STK38L
ENSG00000211751	3.705843	1.331021	0.005366	0.035796	TRBC1
ENSG00000213047	0.922427	0.298538	0.002003	0.018081	DENND1B
ENSG00000213064	0.659316	0.157684	0.000029	0.000801	SFT2D2
ENSG00000213523	-1.00083	0.381655	0.003907	0.028759	SRA1
ENSG00000213551	-0.91478	0.23913	0.000131	0.002468	DNAJC9
ENSG00000213585	-0.64433	0.146885	1.15E-05	0.000396	VDAC1
ENSG00000213593	-0.51501	0.165301	0.001836	0.016985	TMX2
ENSG00000213614	-0.7946	0.293828	0.006845	0.04234	HEXA
ENSG00000213619	-1.00083	0.273145	8.17E-06	0.000302	NDUFS3
ENSG00000213658	-0.77495	0.265423	0.003504	0.026756	LAT
ENSG00000213699	-0.84761	0.310957	0.006414	0.040505	SLC35F6
ENSG00000213719	-1.00083	0.231901	3.03E-11	1.04E-08	CLIC1
ENSG00000213722	-1.00083	0.462287	0.001845	0.017044	DDAH2
ENSG00000213965	-0.8015	0.18623	1.68E-05	0.000522	NUDT19

ENSG00000214013	0.828197	0.234127	0.000404	0.005743	GANC
ENSG00000214022	0.777334	0.208846	0.000198	0.003383	REPIN1
ENSG00000214026	-1.00083	0.402566	4.49E-05	0.001112	MRPL23
ENSG00000214046	-1.00083	0.416814	0.006752	0.041979	SMIM7
ENSG00000214160	-0.65024	0.197689	0.001005	0.011035	ALG3
ENSG00000214176	0.687726	0.219997	0.001772	0.016539	PLEKHM1P1
ENSG00000214193	-1.00083	0.315768	1.48E-07	1.37E-05	SH3D21
ENSG00000214253	-1.00083	0.294255	0.00028	0.004307	FIS1
ENSG00000214309	-1.00083	0.642264	0.004802	0.033126	MBLAC1
ENSG00000214706	-0.94931	0.349349	0.006581	0.041141	IFRD2
ENSG00000214736	-1.00083	0.219092	7.07E-07	4.64E-05	TOMM6
ENSG00000214837	2.48069	0.674957	0.000238	0.003907	LINC01347
ENSG00000214941	-1.00083	0.401622	0.001693	0.016037	ZSWIM7
ENSG00000214954	2.550742	0.797881	0.001389	0.013911	LRRC69
ENSG00000215012	-0.82885	0.302973	0.006225	0.039729	RTL10
ENSG00000215158	1.570678	0.30746	3.25E-07	2.52E-05	NA
ENSG00000215252	2.102242	0.648912	0.001197	0.012543	GOLGA8B
ENSG00000215472	-0.87392	0.296476	0.003201	0.025216	RPL17-C18orf32
ENSG00000215695	8.107446	1.797006	6.43E-06	0.000253	RSC1A1
ENSG00000215788	2.57484	0.755997	0.000659	0.00807	TNFRSF25
ENSG00000216490	-0.97906	0.33376	0.003352	0.026033	IFI30
ENSG00000216775	4.091733	0.965807	2.27E-05	0.000657	NA
ENSG00000217801	-0.78446	0.29328	0.007478	0.045069	NA
ENSG00000218806	-1.00083	0.728486	0.000579	0.007343	NA
ENSG00000221944	0.991739	0.296963	0.000839	0.009599	TIGD1
ENSG00000221963	1.438821	0.164903	2.66E-18	1.22E-14	APOL6
ENSG00000221983	-1.00083	0.398236	0.002167	0.019157	UBA52
ENSG00000224370	3.389383	1.085924	0.001801	0.016765	NA
ENSG00000224877	-1.00083	0.315799	0.000011	0.00038	NDUFAF8
ENSG00000225422	2.602699	0.831861	0.001755	0.016462	RBMS1P1
ENSG00000225492	2.469863	0.639911	0.000114	0.002211	GBP1P1
ENSG00000225830	0.779241	0.269205	0.003796	0.028143	ERCC6
ENSG00000225921	-0.63698	0.233838	0.006449	0.040575	NOL7
ENSG00000226752	1.088003	0.219831	7.45E-07	4.82E-05	CUTALP
ENSG00000227057	-1.00083	0.733404	0.000542	0.007019	WDR46
ENSG00000227372	0.767757	0.2305	0.000866	0.009835	TP73-AS1
ENSG00000227507	2.278619	0.767591	0.002992	0.024051	LTB
ENSG00000227671	0.911074	0.337523	0.006949	0.042741	ZNF731P
ENSG00000228300	-1.00083	0.385067	3.15E-05	0.000852	FAM174C
ENSG00000228474	-0.9369	0.213381	1.13E-05	0.00039	OST4
ENSG00000228594	-0.5296	0.178646	0.003031	0.024238	FNDC10
ENSG00000229180	-0.69397	0.12863	6.85E-08	7.77E-06	NA
ENSG00000229474	1.418041	0.420971	0.000756	0.008904	PATL2
ENSG00000229833	-1.00083	0.424547	0.003472	0.02666	PET100
ENSG00000229859	-1.00083	1.053288	0.003253	0.025582	PGA3
ENSG00000230359	2.332289	0.672758	0.000527	0.006875	TPI1P2

ENSG00000230395	-1.00083	0.799299	0.007565	0.045393	ANAPC1P3
ENSG00000230715	1.36192	0.401068	0.000684	0.008272	NA
ENSG00000231389	1.6637	0.491438	0.000711	0.008523	HLA-DPA1
ENSG00000232112	-1.00083	0.26466	1.71E-05	0.000526	TMA7
ENSG00000232119	-1.00083	0.535527	0.007406	0.044749	MCTS1
ENSG00000233276	-0.94727	0.30032	0.001609	0.015484	GPX1
ENSG00000233750	1.391608	0.495084	0.004941	0.033866	CICP27
ENSG00000234571	1.293671	0.46396	0.005298	0.035515	H2BP2
ENSG00000235043	-1.00083	1.699082	0.005772	0.03755	TECRP1
ENSG00000235602	2.821556	0.881667	0.001373	0.013779	POU5F1P3
ENSG00000236032	2.926837	0.853517	0.000605	0.007633	OR5H14
ENSG00000237094	1.956796	0.584124	0.000808	0.009332	NA
ENSG00000237296	1.599275	0.370903	1.62E-05	0.00051	SMG1P1
ENSG00000237651	-0.86208	0.275744	0.00177	0.016539	C2orf74
ENSG00000237988	2.400299	0.807961	0.00297	0.023958	OR2I1P
ENSG00000238227	-0.60897	0.130683	3.16E-06	0.000142	TMEM250
ENSG00000239264	-0.64874	0.221603	0.003417	0.026418	TXNDC5
ENSG00000239474	-1.00083	0.901513	0.000255	0.004053	KLHL41
ENSG00000239521	1.599927	0.544798	0.003317	0.025879	CASTOR3
ENSG00000239642	-1.00083	0.40751	7.36E-05	0.001609	MEIKIN
ENSG00000239672	-1.00083	0.432443	0.001068	0.011596	NME1
ENSG00000239713	0.744528	0.177541	2.75E-05	0.000769	APOBEC3G
ENSG00000239883	1.723672	0.508674	0.000703	0.008439	PARGP1
ENSG00000240356	-1.00083	0.564988	0.002437	0.020863	RPL23AP7
ENSG00000241468	-1.00083	0.230212	4.94E-06	0.000204	ATP5MF
ENSG00000241489	1.391982	0.33091	2.59E-05	0.000736	NA
ENSG00000241837	-0.91717	0.253519	0.000297	0.004518	ATP5PO
ENSG00000242028	-1.00083	0.293147	3.83E-06	0.000168	HYPK
ENSG00000242294	1.346435	0.225554	2.38E-09	4.88E-07	STAG3L5P
ENSG00000242372	-1.00083	0.304804	0.000199	0.003402	EIF6
ENSG00000242457	4.427393	1.254141	0.000415	0.005865	RBBP4P2
ENSG00000242485	-0.97135	0.331584	0.003396	0.026325	MRPL20
ENSG00000242802	-0.91106	0.337311	0.006914	0.042607	AP5Z1
ENSG00000242950	1.646943	0.494881	0.000875	0.0099	ERVW-1
ENSG00000243156	1.906553	0.674897	0.004729	0.032755	MICAL3
ENSG00000243302	1.138315	0.417959	0.006459	0.040586	NA
ENSG00000243927	-1.00083	0.224391	9.71E-08	1.04E-05	MRPS6
ENSG00000244242	-1.00083	0.472995	1.62E-06	0.000087	IFITM10
ENSG00000244257	2.310882	0.60046	0.000119	0.002291	PKD1P1
ENSG00000244682	1.815744	0.377189	1.48E-06	8.23E-05	FCGR2C
ENSG00000246596	1.435323	0.540513	0.00792	0.046853	NA
ENSG00000247596	-0.62929	0.215921	0.003563	0.02707	TWF2
ENSG00000248871	-1.00083	1.179347	0.004902	0.033664	TNFSF12-TNFSF13
ENSG00000249141	-1.00083	0.650313	0.001326	0.013472	NA
ENSG00000249915	-0.51592	0.16489	0.001755	0.016462	PDCD6

ENSG00000250151	0.896387	0.271114	0.000945	0.010535	ARPC4-TTLL3
ENSG00000250317	-0.74087	0.275317	0.007125	0.04357	SMIM20
ENSG00000250479	-1.00083	0.411874	0.001621	0.015571	CHCHD10
ENSG00000250644	-1.00083	0.549465	0.000328	0.004881	NA
ENSG00000250995	2.976069	0.861751	0.000553	0.007103	TMEM30BP1
ENSG00000253352	0.664469	0.235362	0.004755	0.032904	TUG1
ENSG00000254093	-0.96906	0.29794	0.001144	0.012183	PINX1
ENSG00000254536	1.411573	0.475832	0.003012	0.024119	NA
ENSG00000254681	2.305593	0.654145	0.000424	0.005942	PKD1P5
ENSG00000254827	-1.00083	0.806134	0.001927	0.017589	SLC22A18AS
ENSG00000254999	-0.64982	0.22971	0.004671	0.03247	BRK1
ENSG00000255398	2.474805	0.845453	0.00342	0.026426	HCAR3
ENSG00000256223	1.195901	0.356253	0.000788	0.009163	ZNF10
ENSG00000256235	-1.00083	0.272402	2.94E-09	5.93E-07	SMIM3
ENSG00000256618	-1.00083	0.411569	0.001418	0.014119	MTRNR2L1
ENSG00000257093	2.098628	0.591848	0.000391	0.005598	DENND11
ENSG00000257838	-1.00083	0.911124	0.002563	0.021664	OTOAP1
ENSG00000258461	1.781729	0.425481	2.82E-05	0.000787	NA
ENSG00000258659	1.061418	0.332407	0.001407	0.014053	TRIM34
ENSG00000259379	4.3766	1.548243	0.004701	0.032598	MTND5P32
ENSG00000259494	-0.90609	0.212424	1.99E-05	0.000597	MRPL46
ENSG00000259984	3.670711	1.331646	0.005842	0.037832	NA
ENSG00000260314	2.089012	0.385539	6.01E-08	7.19E-06	MRC1
ENSG00000261210	2.18827	0.811417	0.007	0.042988	CLEC19A
ENSG00000261371	0.909058	0.260559	0.000485	0.006491	PECAM1
ENSG00000261499	3.525977	1.288215	0.006198	0.039598	NA
ENSG00000262664	-0.79767	0.261562	0.002291	0.019934	OVCA2
ENSG00000262814	-0.95877	0.307586	0.001827	0.016934	MRPL12
ENSG00000263001	1.547709	0.429685	0.000316	0.004729	GTF2I
ENSG00000263647	1.309699	0.402869	0.00115	0.012204	BPTFP1
ENSG00000263956	0.758482	0.267835	0.004627	0.032264	NBPF11
ENSG00000264187	2.540869	0.87498	0.003685	0.027663	NA
ENSG00000264343	1.605277	0.476449	0.000754	0.008898	NOTCH2NLA
ENSG00000265241	-0.78046	0.206106	0.000153	0.002804	RBM8A
ENSG00000265303	-1.00083	1.431693	0.001733	0.016296	NA
ENSG00000265354	-0.62342	0.191073	0.001103	0.011853	TIMM23
ENSG00000266094	0.898815	0.226493	7.24E-05	0.001587	RASSF5
ENSG00000266302	3.421912	1.150519	0.002937	0.02383	NA
ENSG00000266472	-1.00083	0.38912	0.006994	0.042979	MRPS21
ENSG00000266777	1.440433	0.44896	0.001335	0.013515	SH3GL1P1
ENSG00000266964	-1.00083	0.797898	0.000744	0.008813	FXYD1
ENSG00000266967	-0.84587	0.304193	0.005424	0.036044	AARSD1
ENSG00000267680	0.991563	0.268878	0.000226	0.00376	ZNF224
ENSG00000268350	1.035286	0.27638	0.00018	0.003164	FAM156A
ENSG00000268758	1.272269	0.425055	0.002761	0.022806	ADGRE4P
ENSG00000269713	0.68264	0.159975	1.98E-05	0.000593	NBPF9

ENSG00000269858	-1.00083	0.384496	0.006558	0.041061	EGLN2
ENSG00000270188	-1.00083	0.5848	0.002093	0.018621	MTRNR2L11
ENSG00000270392	1.238592	0.335267	0.00022	0.003686	PFN1P2
ENSG00000270890	3.28058	1.070122	0.002172	0.019191	NA
ENSG00000271092	2.655664	0.842072	0.001612	0.015498	TLCD4-RWDD3
ENSG00000271425	0.731686	0.266136	0.005972	0.038513	NBPF10
ENSG00000272047	-1.00083	0.40619	0.002575	0.021746	GTF2H5
ENSG00000272617	-1.00083	1.444282	2.31E-06	0.000112	NA
ENSG00000272886	0.984287	0.326875	0.002602	0.021897	DCP1A
ENSG00000273331	-1.00083	0.710872	0.003712	0.027742	TM4SF19-DYNLT2B
ENSG00000274523	-0.52159	0.188549	0.005669	0.03714	RCC1L
ENSG00000274736	2.613974	0.745041	0.000451	0.006206	CCL23
ENSG00000275700	-0.63093	0.233378	0.006862	0.042381	AATF
ENSG00000275895	-1.00083	0.549945	0.002433	0.020863	U2AF1L5
ENSG00000276023	-1.00083	1.511401	0.008569	0.049354	DUSP14
ENSG00000276180	-0.69297	0.238997	0.003738	0.027889	H4C9
ENSG00000276600	-0.90016	0.157231	1.03E-08	1.65E-06	RAB7B
ENSG00000277117	1.550954	0.265046	4.87E-09	9.03E-07	NA
ENSG00000277363	-1.00083	0.333558	8.34E-06	0.000306	SRCIN1
ENSG00000277734	1.965058	0.395138	6.59E-07	4.41E-05	TRAC
ENSG00000277791	-1.00083	0.400943	0.008551	0.0493	PSMB3
ENSG00000277972	-1.00083	0.744455	0.004003	0.029167	CISD3
ENSG00000278129	0.933187	0.308303	0.002471	0.021072	ZNF8
ENSG00000278384	2.088085	0.716658	0.003572	0.027097	NA
ENSG00000278616	1.743125	0.656032	0.007882	0.046685	BEND3P3
ENSG00000280828	1.8116	0.643672	0.004886	0.033622	NA
ENSG00000281490	1.542562	0.45434	0.000686	0.008274	CICP14
ENSG00000281991	-1.00083	1.5124	0.001593	0.015393	TMEM265
ENSG00000282458	1.118907	0.181144	6.54E-10	1.58E-07	WASH5P
ENSG00000283050	1.206681	0.43495	0.005532	0.036602	GTF2IP12
ENSG00000283149	-1.00083	0.384953	2.43E-06	0.000116	NA
ENSG00000283321	4.334843	1.221489	0.000387	0.005557	NA
ENSG00000283945	3.937768	1.226916	0.00133	0.013493	LINC00032
ENSG00000284610	1.929646	0.690391	0.00519	0.035052	NA
ENSG00000284862	1.366836	0.512571	0.007662	0.045773	CCDC39
ENSG00000285749	1.096931	0.356546	0.002094	0.018621	NA
ENSG00000285901	-0.52705	0.196468	0.007304	0.044275	NA
ENSG00000286022	-1.00083	0.872108	0.000332	0.004921	NA
ENSG00000286070	-0.91223	0.329169	0.005583	0.036798	NA
ENSG00000286169	0.944622	0.325499	0.003707	0.027724	NA
ENSG00000286239	2.302997	0.756482	0.002332	0.020178	NA
ENSG00000288258	-1.00083	0.717988	0.000233	0.003857	NA
ENSG00000288579	2.445534	0.56352	1.43E-05	0.00047	NA

***Note**: lfcSE, log2 fold change standard error; padj, adjusted p-value; hgnc symbol, HUGO Gene Nomenclature Committee (HGNC) gene symbol.*
