# Supplementary material for: Ex vivo expanded human regulatory T cells promote cholesterol efflux and PON1 expression in oxLDL-exposed macrophages via gap junction-mediated cAMP transfer
Source: Front Immunol. 2025 Oct 16;16:1662925. doi: 10.3389/fimmu.2025.1662925 (PMC12571657; doi:10.3389/fimmu.2025.1662925)
Supplement: Supplementary file 2 [file DataSheet2.pdf]

Supplemental Table S2

	GENE ONTOLOGY PATHWAYS
**Pathway**	**Gene/product**
Regulation of lipid localisation	CIDEA, CIDEC, CIDEB, PLPP3, PLPP4, CLPTM1L, PLPPR4, VPS13D, VPS13C, STARD4, HILPDA, SCP2, STARD3, MAL, BLTP1, CKLF, PLP2, CMTM6, TRIAP1, SOCS2, STAT5B, PGAP1, BAD, UPF1, BCL2L11, SNF8, STAT3, TOM1, IKBKE, MED1, RAP1B, TTC39B, DENND5B, APOC4, anxa2-pcsk9_human, ENPP7, PLIN5, BSCL2, FABP3, VSTM2A, PRELID1, URS000075B7E4_9606, PCTP, TMEM30B, URS00003BBF48_9606, TNFAIP8L3, OSBPL8, ERFE, MTLN, URS000034B6F5_9606, RUBCN, ABCG1, URS0000483184_9606, URS00004C8DD5_9606, CES1, CETP, URS0000251D0B_9606, CNEP1R1, APOC3, URS00001012BC_9606, FITM1, URS000030BD69_9606, APOA2, DBI, URS00002367FA_9606, ATG2A, YJEFN3, URS000059311D_9606, LPCAT3, STAR, PLA2R1, ABHD5, URS000024A59E_9606, C1QTNF1, URS0000070CD2_9606, APOC2, ABCG5, ABCG8, ORMDL3, URS000019907A_9606, URS00002C0FCB_9606, HCAR2, URS00004208C5_9606, CPTP, PLIN2, ABCA2, PLSCR5, CRP, PNPLA2, NMB, AVPR1B, ABCG4, APOC1, HADH, PLIN3, SPHK2, IRS1, MSR1, NR1H3, IRS2, DISP3, URS000037C5A8_9606, APOD, SCARB1, ABCA5, ARV1, SLC51B, URS00002075FA_9606, URS0000155642_9606, C2CD2L, LDLR, URS00003B95DA_9606, APOA1, PLA2G2E, SPP1, ANXA8L1, XKR8, MFSD2A, DAB2, GPS2, OSBPL11, SPNS2, GPLD1, PPARG, GAL, SPX, NR1H2, URS000024463E_9606, FABP1, URS00004176D4_9606, URS00000B7E30_9606, WASHC1, CRY1, PLA2G5, PNLIP, FABP5, GHRL, ADIPOQ, IL1A, PLTP, PLA2G10, GALR1, URS00000E5433_9606, STAP1, BIN1, CYP8B1, SMIM22, LPL, DGKQ, TTPA, URS000058760A_9606, CEACAM1, TSPO, PPARD, ANO9, IFNG, ABCA7, URS000024B619_9606, SREBF2, FIS1, ANGPTL3, APOA5, PIBF1, C1QTNF3, SPHK1, PLSCR1, CCR7, TMEM30A, APOL3, CHP1, SCT, URS0000527F89_9606, PCSK9, CD19, TNFRSF11A, CYP27B1, C3, CFHR4, LIPG, CCL19, LPAR1, MTMR9, GPIHBP1, AGAP2, AGT, PLA2G1B, OSBPL7, CCL21, ABCA12, SCAP, URS000013D17D_9606, CREBL2, NEU3, SIK1, SGPP1, FABP6, LBP, APOLD1, PDE8B, TRIB3, EEPD1, atp10d-tmem30a_human, APOA4, APOB, SERPINE1, LDLRAP1, ATP10A, PRAP1, FFAR3, DOC2B, AVP, PRKCD, EPN2, FFAR1, AVPR1A, PLA2G3, FFAR2, ADTRP, CRY2, SEC24A, ABCD2, APOM, CRABP2, URS0000574A2C_9606, ZDHHC8, ENPP2, BMP6, OSBP, SNX12, IL13, ABCA8, SNX4, ADORA1, PTPN11, INS, MIF, CNR1, CD300A, FGF19, ANO6, FBXW7, SLCO3A1, PER2, UCN, KCNN4, ALOX5, RXRA, INSIG1, ABCA3, INPP5E, PID1, ABCA13, PTPN2, ADM, FITM2, PRAM1, TNF, P2RX7, ATP9A, PLA2G6, GSTP1, SORL1, CD36, PLCG2, FGF23, PIK3R2, DGKD, LIMA1, GBA1, TREM2, ROCK2, SIRT4, URS000019B0F7_9606, NFKB1, MYB, AGTR1, SSTR4, ALOX12B, THBS1, IL1B, LRP1, ABCD1, P2RY12, MOSPD2, ABCB4, PON1, NFKBIA, EFNA5, PPP2R5A, AKT1, MFSD2B, IL4, SLC45A3, PLCB1, URS0000338542_9606, RBP4, PPARA, F2, PIRT, SIRT3, CSF2, TEX2, PLSCR2, PITPNC1, AACS, PROM2, APOE, REST, APOH, ALG10B, INHBA, NUS1, NR0B1, PLA2G4A, FASLG, SAA1, MAPT, ZC3H12A, PIK3R1, CRH, ABCA1, ADRA2A, FABP7, CD74, CDS1, CPT1A, GPER1, FABP4, PIP4P2, FGR, LILRB1, KAT5, GHSR, LAPTM4B, CXCL10, PRKAA1, FGF21, INPP5F, ACSL3, ARFIP1, CYP51A1, TNFSF11, BRCA1, PLD1, HMGB1, PRKD1, TM9SF2, TWF1, TLR9, FGF2, PLSCR3, CYP7A1, URS00001DC04F_9606, PLA2G2D, GALR2, SESTD1, MAP2K6, TMF1, EGF, TACR1, TARDBP, ANXA2, SIRT6, HMGCR, NTSR1, LHCGR, F2R, NCF1, SCIN, URS000050B527_9606, THY1, PTAFR, BMP2, RETN, PLCG1, XBP1, PTGES, SNX13, DRD3, ACACB, ADCYAP1R1, CD300LF, SNX30, SNX7, ANXA1, RAB38, CAV1, ADAM9, GIT1, INPP5K, TRPC5, WDR91, NOS2, HTR2B, SPI1, RAB3GAP1, ATP8A1, LILRB2, RFT1, HIP1R, PLA2G2A, ACAA2,

	ALOX15, RASGRP1, CD24, P2RY6, PHB2, DKK1, MTM1, P2RX4, SNX33, PRKAA2, SYK, PIK3CG, BDKRB1, HTRA2, NRIP1, LRP6, ABL2, GPR155, CYP19A1, LEP, TICAM2, TGFB1, OSBPL6, PLEK, DRD4, ABCB1, IL10RA, CYGB, SYT3, FTO, PLA2G4E, EHD1, PRKAG1, RUBCNL, GAB2, SAR1B, CLU, S100A13, IL10, TNFAIP3, NR1D1, FERMT2, GIPR, SNX3, SYT17, NUP62, PRLR, OGT, ATG14, SREBF1, SSTR5, VMP1, EPB41, APPL2, AXIN2, GDF9, SNX9, SMPD1, PFN2, ARHGEF5, SIRT1, INHBB, DNAJA1, PACSIN3, ATP11A, FFAR4, PIK3CA, LYN, PASK, SLC35C1, SNX25, GATA2, HNF4A, XKR5, XKR4, XKR6, XKR7, XKR9, IL12B, AKT2, ITGAV, PIP4K2B, PYCARD, URS000039ED8D_9606, YWHAH, SCARF1, SOAT1, SESN2, PTGS2, NKX3-1, AJUBA, GSDME, PLCE1, LYPLA1, SIRPA, LRP8, PLA2G2C, SNX5, PLSCR4, TWF2, TOM1L2, PTPRC, SLC27A1, ADORA2A, TMEM41B, SFRP1, TNFRSF1B, IL12A, PIK3CB, PPIA, CXCL8, NR0B2, NR1H4, PIM1, SNX18, SH3GLB1, TESC, CD38, LGALS9, SLCO1C1, ZMPSTE24, SYT4, TLR2, SLC66A2, PDGFB, PDGFRB, PTEN, YES1, TRIM6, PTK2B, CAV3, RAMP3, PIAS4, DNM1L, SOD1, PLA2G2F, GSTM2, MACROH2A1, CD4, RSAD2, PPID, CLN3, RAPGEF2, FES, PTH, APOBEC1, KAT2B, MAPK8, CYP4A11, UNC119, SYT11, VDR, IGF1R, RNF213, ILDR1, WNT5A, SNX32, MALT1, NUMA1, NPC2, S100A9, ACVR1C, APPL1, ATP8A2, ELANE, GSK3B, MTMR2, CLCN2, MECP2, ANG, ANXA8, CCL2, RBP1, PRKAG2, LRP5, TIFAB, POMC, S100A8, SELE, ANKRD1, ANO3, INSIG2, VIL1, TNFRSF1A, GSK3A, BCAP31, CTDNEP1, PRKG1, LRAT, HNRNPU, ITGB3, SELENOS, DYSF, PLP1, G6PD, EPHB2, CEL, KIT, PISD, IRGM, ATP8B1, FURIN, CALCR, NLRP6, TOM1L1, HTR2C, SOX30, NPC1, BAIAP3, SNX6, EPO, LTF, OPRK1, OR51E2, NOS3, CFTR, NAXE, MEF2C, WBP2, CCDC88A, DENND1B, LAMTOR1, GRM5, SORBS1, HTR2A, CGA, RTN4, GATA1, TAF7, SMPD3, SYTL4, WNT3A, atp10b-tmem30a_human, ATP9B, CYTH3, NOD2, PRKCE, GBP2, SDCBP, GOLPH3L, PLEKHF1, PLAA, FER, GHR, ACE, CCL3, CD14, EDN1, MSN, FZD4, FCHSD2, CD81, ATM, BCR, PLCH2, CSNK1G2, SMURF1, MAPKAPK2, NCOR2, CYP4F2, MTCH2, CPT2, IL6, CRK, GCKR, AR, GSN, GIP, ANKFY1, BAX, WNT11, atp11c-tmem30a_human, atp11a-tmem30a_human, atp10a-tmem30a_human, atp8b2-tmem30a_human, atp8a2-tmem30a_human, atp8a1-tmem30a_human, atp8b1-tmem30a_human, ATP10D, NF1, INPPL1, YAP1, PTCH1, SGIP1, CXCL11, SURF4, PTPRU, LCAT, CREB1, GBP5, ZFP36, HGS, TRPV4, NR4A3, MTTP, VAPA, ADGRB1, AKIRIN2, BGLAP, CDKN1B, ABL1, PLD2, ZDHHC5, GH1, SLC4A1, YY1, NFKBIB, HES1, P2RX1, TIAM1, AP3D1, MMP9, ALKBH7, MAPK14, EZH2, GGA3, UBE3A, HAVCR1, SLC7A5, REN, HSPA1A, NR3C1, HCK, ITGA2, SNCA, STK39, TGFB3, AXL, FZD5, CDH13, PAF1, GAS6, TGFB2, ENPP1, CALR, ACSL5, CYTH1, TSC1, POU4F2, BPIFA1, TFRC, ANO4, MBTPS1, PICALM, PEX5, VAC14, HSF1, STK11, RFTN1, CXCL9, PIP4P1, FNBP1L, HEXB, TSKU, RCN3, PIK3CD, ACSL1, SHH, BCL2L2, PLCB3, PLCB4, CRABP1, SLCO4A1, SYT13, SYT15, WASHC2C, RUVBL2, PFN4, NR1I2, PIP4K2A, WDR45, BDKRB2, MLC1, ZW10, MAP1B, DDX3X, NEDD4, RPS6KB1, PCK2, PPT1, OXTR, UBE2L3, ACTN2, WNT7A, STXBP1, pik3c3-atg14_human, TGFBR2, DHCR24, CDKN1A, OPN3, PRKCI, PXK, AP3B1, EIF6, CRKL, NR5A1, SLC9A1, SLC25A27, PLCD1, UGCG, DOC2A, APP, PAFAH1B1, PACSIN1, CASR, QKI, FOXO1, BTK, WASHC2A, KDM5B, JPH2, SPTBN1, ATP1A1, RB1, SPTBN4, CLDN4, MARK1, SYP, HDAC6, ARHGAP44, ESR1, FGF10, SQLE, EGFR, CLIP3, GABARAPL2, PTGES2, SRC, UBR5, EDNRB, PRKN, HSPG2, CADPS, SYT12, NOS1, ERBIN, ABCC8, ATP8B3, TRIM25, TLR4, ATP13A2, AP2M1, SYT7, SLC44A2, PSAP, CADPS2, PLCZ1, SCARB2, SLC27A4, PDE4B, HSPD1, TXNIP, SH3GL2, CRHR1, PLCB2, HTR1B, HIP1, DENND1A, EPN1, PITPNM1, CPNE1, IRF8, SH3GL3, CERT1, PRKCB, MTOR, SYT1, UNC13A, ISL1, CLN8, ZFYVE1, USF2, CEBPB, CYTH2, NDUFA13, ABCB11, CEBPE, CDK5R2, CLSTN3, WDR81, RARA, SMG1, STAM, CBL, RAB7A, STC2, ZFP36L1, ATP8B4, ATP10B, ATP11C, XRCC5, COMMD1, PICK1, NOTCH1, MYD88, HSPA8, UCN3, BECN1, DRD2, VDAC2, CITED1, PTGDS, VLDLR, SNX17, NCOA4, OSR1, CTNNB1, SPTBN2, NPHP3, C2, RAPGEF6, SYT6, JAK2, LRP2, SOAT2, GNAI1, ATP8B2, VDAC1, UBE2G2, SLC22A13, GSDMD, PARP1, PRKACA, NCOA1, FNBP1, SMAD2, C2CD5, EPHA3, SYT10, SERPINA5, SLC11A1, ARHGAP33, USP8, UCP1, ATG9A, ATG9B, ATF2, MARK2, SMO, PDZD8, UVRAG, ITPR1, MYO10, ATP1A2, IL1RN, LEPR,

	STAB1, MGARP, GRAMD2A, AIFM1, NSFL1C, SLC6A4, PDX1, GPAT4, MAPK3, MAOB, CRYAB, EIF4E, FYB1, AKAP8, PEX2, SLC22A2, MYOF, VPS4B, G6PC1, SIGMAR1, MDM2, ZFYVE16, ANXA3, LONP2, AGAP1, MVB12B, PLEKHA1, CRHBP, NEFL, BBS4, PIK3C2A, ABCA4, SPNS1, ANO7, SELENOM, DAG1, SGK1, PRPF19, REEP2, TULP3, METTL21C, H2BC11, BCL2, EXOC7, ACSL4, KCNMB1, SYT2, WNK4, ADCY8, STC1, LIPA, TBC1D20, ATP1B1, CHAT, WIPI1, TREX1, SMYD3, GHRHR, RUFY1, COL1A1, ICA1, TPCN2, FOSL2, PCSK6, KCNK4, ASAP1, SYT9, DGAT2, PLAAT3, SOX9, MAPK1, LIPC, VPS4A, KCNQ1, OPHN1, BIN3, CCR5, TRIM72, ZDHHC7, NR4A1, TRPV1, STX3, PNPLA8, ADCY5, VAMP2, DNAJC15, AQP1, GGA1, PAX6, SDC1, SLC22A9, GABARAP, SYT5, UNC13B, PORCN, VCP, P2RY4, DGAT1, SYT8, GOLPH3, EXOC8, SYNJ1, RACGAP1, PABPN1, PACSIN2, AQP3, SNX14, PGRMC2, SNX27, KMO, ATP11B, EXOC1, PI4KB, THEM4, PARD3, AKR1C1, GBF1, TMEM38B, TPCN1, PPP1R9B, CACNA1H, TRIP10, VPS54, PEX19, KCNJ2, RAN, GPR119, ABCC2, SLC38A9, escrt-iii_human, EPRS1, RPH3A, UCP2, AKR1C4, PIK3R4, SLC10A1, ITPR3, PGRMC1, PCSK1, PCLO, SNAP25, CD68, CYBB, OPA1, CHMP2A, CLDN1, ARAP3, NMUR2, CHMP3, DNAJC19, PIP5K1A, SLC16A1, FKBP4, STOML2, BBS1, ATP5F1A, AFP, HEXA, SNAP91, VAPB, ARFIP2, SLC44A4, NAGLU, SNX19, KCNJ1, SLCO1B1, AQP8, COLEC12, MAPKAPK3, SERINC5, MCOLN1, SEL1L, UNC13C, LARGE1, CYP46A1, ABCC1, SPAST, UGT1A3, STRA6, TULP1, PIKFYVE, PPBP, PCSK5, PIK3C3, RAB18, GRIA1, MVB12A, ALB, CHMP5, STX12, SLC6A1, TMEM175, MCOLN3, IGF2R, ATP6V1B1, ATP5F1B, GJB3, KCNJ8, SH3GL1, ZFYVE9, OBSCN, PTGDR, SPG11, SDCBP2, KIF18A, KIF16B, ANXA6, PDIA2, CHRM5, SLC30A5, DNAAF2, PIP5K1C, UMOD, RILPL1, AMPH, SLC34A1, MYO1G, SCNN1B, GABRB1, KCNH1, CHMP4A, ARL6, MYCBPAP, SLC9A3, SCNN1A, AP2A2, PTPRN2, ITPR2, SCNN1G, SCN11A, SLC5A5, TH, ATP1A3, RAB40C, ANXA9, GJB6, STAM2, ABCC4, RAB40A, CDO1, EEA1, SLC12A3, SCNN1D, CNP, GJB2, ATP4B, SLC22A1, RAB35, MYO1E, CAD
Cholesterol-protein transferase activity	GSTM5,GSTA4,ALG13,NMT2,ALG5,LANCL1,ALG14,GSTM4,GSTA5,HPGDS,TFB1M,NMT1,DIMT1,GSTA2,GSTM2,GSTP1,GSTM3,GSTM1,AASDHPPT,CIDEB,CIDEC,CIDEA,IBA57,ALOX5AP,TMEM260,GSTK1,MGST3,LTC4S,PLA2G15,ATE1,MGST2,OXCT2,LCAT,FKRP,TMTC2,RMND5A,RMND5B,TMTC1,POMT1,POMT2,TMTC4,TMTC3,MAEA,GSTO2,GSTT1,GSTT2B,PIGG,MGST1,PIGO,PIGN,GSTO1,FKTN,PGGT1B,FNTB,RABGGTB,PDHX,RABGGTA,DLAT,GMPPA,ELOVL4,GMPPB,ELOVL7,ABO,ELOVL2,ELOVL5,ELOVL1,ELOVL3,ELOVL6,PTGES,SEPSECS,GCNT2,PTAR1,FNTA,GSTCD,MGAT3,EOGT,METTL1,GTDC1,B3GNTL1,SOAT2,APOA2,NAT16,ZDHHC8,FAM86C2P,PCED1A,ampk_human-2,SOAT1,FDFT1,RNF214,SCP2,FDPS,APOA1,LPCAT3,methylosome_human,METTL27,dapk-calm_human,ampk_human-4,ampk_human-5,LSS,METTL25,MB21D2,abeta-42_human,FAM86C1P,HMGCS1,LONRF3,LONRF2,APOA4,SULT2B1,LRP6,PMVK,URS000059311D_9606,tfp_human,NUS1,ampk_human,METTL24,EEF1AKMT4,MYLIP,LONRF1,MVK,piccolo_nua4_human,cdk5-p25_human,cdk5-p35_human,ccnk-cdk12_human,cyclind3_cdk4_human,cyclind2_cdk4_human,cyclind1_cdk4_human,NEURL4,METTL9,PMS2P11,ADCK5,ART5,NAT9,FBXO40,WDSUB1,STKLD1,NOSIP,FBXW2,APOE,abeta-42-oligomer_human,FBXO24,HECTD2,KIAA1586,HERC2P3,UBR7,RNF39,PJA1,COMTD1,FGF1,LNX2,ARMT1,RNF44,RNF225,RNF227,RNF223,cdk10-cyclinm_human,MYLK4,MKRN4P,LOC122319436,TGM7,ACAA2,MED13,RNF182,PCMTD2,OSBPL8,NEK5,PSKH2,SBK3,G6PC1,ranbp2-rangap1-ube2i_human,PPARG,ART3,atr_atrip_human,casein kinase2a_human,ACSM1,WSB1,FBXO30,lubac_human,URS00003B95DA_9606,GBA2,UGT3A1,PIP4K2A,UBOX5,CAB39L,bard1-brca1_human,ccnl2-cdk11b_human-1,EGF,SULT2A1,G2E3,DZIP3,UBE3C,UBE3B,GLT1D1,brcc_human,NRK,PRKAA1,FAM86B1,FAM86B2,PRKAA2,FBXL6,ACLY,ASB12,PRMT9,TGM6,GID4,TNK1,LNX1,METTL21EP,CBLL2,TRIM69,HERC3,RFPL4B,TRIM50,TRIM74,TRIM73,TRIM61,RNF181,KLHL13,KLHL9,RSPRY1,FBXO42,GLT8D1,GLT8D2,TSPAN17,CNPPD1,PHKG1,MAP3K15,PHKA1,MAP3K19,EEF1AKMT2,RNF148,RN

	F150,NEURL2,RNF215,RNF208,FBXO25,FBXO21,NEK9,RSKR,ANKK1,FBXL14,TKTL2,ccny-cdk14_human,fa_human,STK31,RPS6KL1,NTMT2,MARCHF4,MARCHF9,HECTD3,MARCHF11,HECTD4,PDZRN3,PCMT1,MKRN3,SETD9,FBXO11,ADCK2,ADIPOQ,RNF130,MTCP1,TCL1B,KLHL21,ASMTL,RNF7,FGFR4,RNF170,UGT2A3,FBXO44,FBXO27,FBXO17,LRP8,ENTREP1,TRIM60,CSKMT,MKRN1,METTL15P1,TMEM68,DHH,FGGY,RNF175,RNF121,N4BP2,PRMT8,ACCSL,ZFP91,UBE2NL,UBE2U,PCMTD1,SBK1,PRKY,EEF2KMT,SATL1,cpc_human,LRP5,SBK2,TBCK,UHRF2,DGKQ,CAV1,KLHL42,FBXO10,TRIM47,THUMPD2,TGM5,DGAT2,tgfb3-tgfbr1-tgfbr2_human,BRAP,MARCHF6,O00370,PEX12,SIRT1,cyclina2-cdk2_human,SULT1C2,INMT,DPH1,DPH2,CLU,REV1,cab39-strada_human,cyclinb1_cdk1_human,PARP12,GLYATL3,SOD1,RANBP2,ACSM3,FN3KRP,SMYD4,MAP3K21,MAPK4,PKN3,TRIM17,EPHB4,RNF11,EEF1AKMT1,RIPK4,dna-pk_human,NIM1K,HIPK4,STYK1,MATK,NEK8,ROS1,ERVK-7,FBXL21P,HDAC9,MAP3K9,CCNJL,UBE2D4,NCCRP1,CRIM1,UBE3D,UBE4A,UBE2L5,ERVK-25,ERVK-18,ERVK-8,ERVK-19,ERVK-11,ERVK-6,ERVK-10,OBI1,CDKL4,CDKL2,DYRK4,MOK,PARP8,TRIM64C,TRIM64B,RNF151,TRIM55,AREL1,HERC6,TRIM64,TRIML2,TRIM51G,TRIM49C,TRIM49,TRIM48,RNF103,TRIM43B,TRIM7,TRIM51,TRIM75,TRIM43,TRIM49D1,TRIM4,TRIM77,TRIM49B,BSPRY,RNFT2,TRIML1,KCMF1,DUSP19,HERC5,naa10-naa16_human,RNF145,GRK6,SH3BP5L,SH3BP5,FBXO9,FBXO3,UGT2B4,UGT2A2,UGT2A1,CAMK1G,PNCK,NEK1,CAMKV,MAP3K6,DUSP7,ARK2N,DPH5,SGK2,INSRR,SETD1B,THAP9,MYCNOS,SELENOO,FICD,TGFBR2,PRRC1,KSR1,LTK,CLK1,KSR2,PSKH1,RPS6KC1,MAST4,STK32A,STK32C,PEAK1,MAST3,PDPK2P,DCLK3,STK32B,ASB4,ostb_human-1,osta_human-1,SMYD5,UBA3,PIPSL,RNF122,FAM20B,STRADA,UGT3A2,RING1,LIPT1,CD24,CBLL1,MIB2,CDK8,HMGCS2,FANCL,ZNRF2,RNF141,TTC3,TRIM36,GLT6D1,TRMT1L,METTL25B,HEXIM2,ACCS,CLK4,LTN1,DYNAP,DOK7,mcc_human,MST1R,TPST2,TPST1,EED,RPAP1,TRMT12,PARP4,UBE2M,PRKD3,MAPK10,PDGFRL,MEX3C,ZSWIM2,PPIL2,METTL22,ccnc-cdk3_human,DUSP12,HIPK3,TGM4,AATK,LCMT2,TEC,VCPKMT,TREM2,STT3A,CTU2,CDK3,CDK14,PHKG2,CDK19,TRIM9,NEK10,cak_human,PHKA2,BMP2K,MKNK1,MAPK6,PEAK3,RNF133,TRIM45,NEURL1B,MARCHF8,RNF123,NEURL3,RNF114,RNF166,RNF38,KLHL20,UBE2L6,SMPD1,URS0000D5A4C0_9606,URS0000593928_9606,URS0000D5DD7A_9606,KLHL2,UGT2B10,UBE2G1,PHYKPL,ZNF738,HERVK_113,CDKN3,FBXL3,DDB2,FUT11,CCNJ,CCNG2,CCNI2,CCNP,PEX10,ARRDC4,JTB,ADCK1,MMD,LMTK3,ALPK3,PELI2,CDK18,CDK17,CDKL1,CDK15,CDK11B,CDK11A,HEMK1,HUNK,POLE4,STK38,TCL1A,UBE2H,GCKR,TPD52L1,MMACHC,PAFAH1B3,PPP1R11,UAP1L1,PMS2P1,FBXO6,GCSH,ZNF598,ART4,ART1,F13A1,DOLK,MAPKAPK5,INCA1,TRMO,MAP3K11,POLR2C,POLR2J2,POLR2J3,PRKAB2,PCGF3,PRIMPOL,GBA1,NRBP1,CAMK2N2,METTL21A,CUL5,GRK7,GRK3,GRK4,MARCHF3,FBXL22,UBE2R2,COLGALT2,CDC14B,UBE2Z,TMEM129,SIAH3,PELI3,TRIM54,HACE1,MARCHF5,RNF115,HERC4,TRIM35,RNFT1,TRIM10,UBE2D2,TAB1,FN3K,DBNDD1,DBNDD2,TIE1,DSTYK,CRCP,UBE2F,RNF25,ERVK-9,PRKAG1,AKT1S1,UBE2QL1,DTX2,mocs2-mocs2-5,CBX4,PRKACG,ZNF451,DCAF1,URS000030BD69_9606,C10orf90,HECW1,HECW2,MARCHF10,NDUFAF7,EEF1AKMT4-ECE2,PARP11,UBE2E3,CCND3,f13_human,DCLK2,PRPF4B,STK38L,MAST2,MAST1,TLK1,CILK1,MAPK12,PRMT7,DUSP16,CIMAP3,RAD18,TRIM14,ICMT,FLT1,TSR3,PRKAR2A,PRKAR2B,PRKAR1B,CARNMT1,MECOM,CHM,AAK1,SRPK3,RNF112,UBE2Q2,SETBP1,EPHA1,RNF20,B4GALNT3,B4GALNT4,UBE4B,VPS25,ITK,FGFR1,RPGR,UFC1,TGFBR1,RPL37,ULK4,PKIB,ccnl1-cdk11a_human,CALM2,CLK3,GPHN,CASS4,C1GALT1C1L,tgfb1-tgfbr1-tgfbr2_human,p53-mdm2_human,RNF157,TNK2,VPS18,RNF152,ZNRF1,UBE2S,BIRC8,CAMKK1,TLCD3B,UGT2B7,UGT2B15,UGT2B28,UGT2B17,UGT2B11,RBBP6,WDR24,RNF113A,ZMIZ2,PRPSAP2,ASB1,PRPSAP1,ZDHHC13,CALM3,CDK5RAP1,NTRK3,ALK,TRMT61A,COP1,WWP1,TRIM23,TRAF7,CUL9,SIK2,TNNI3K,FDXACB1,CHKA,BMX,SRMS,CERT1,CCNYL1,CCNYL2,NSMCE1,PRKAG2,PRAG1,TP53RK,METTL13,MAP3K5,SHH,P3R3URF,SUZ12,STT3B,EFNA4,IRF2BP1,FLT3,UBE2E2,PPM1E,DEPTOR,NAT2,PNPLA4,CEBPA,LY6G6E,LMTK2,KAT14,

	KLHL7,CBLC,pol-prim_human,FTCDNL1,STRADB,SHPRH,ASPG,MKNK2,PRMT3,TENT5D,POGLUT3,POGLUT2,BTRC,APPBP2,PRDM10,EEF1AKMT3,TRPM6,TRPM7,BRD2,TRMT112,URS000000B23E_9606,URS00006F4087_9606,URS00003ACFFF_9606,URS0002590D51_9606,CDK7,MAP3K2,FGFR3,TRIM59,LOC122513141,RNF43,MARCHF1,RNF146,MGRN1,RNF149,RNF186,RNF212B,STK17B,STK40,TEP1,STK17A,NEK11,LTBP1,TRIM26,PIAS2,NHLRC3,TRIM2,DSEL,NRBP2,RPN2,RPN1,ube2n-ube2v1_human,MAP3K13,CEP43,ALKBH8,PPM1F,UBL4A,TGM3,CDKN2C,PTK6,PARP15,PPM1D,PIGF,CISD1,TOPORS,PRMT6,ANAPC4,RNF40,MOCS3,LMO7,ERCC8,UBA6,MAPK8IP1,DSCC1,LTBP4,PRDM6,PRDM7,TRIB3,FUT6,NAA11,PLAAT2,TRIM40,BCCIP,RIOK1,QPCTL,PIK3IP1,YWHAG,ZDHHC24,MAP3K12,ZDHHC4,ZDHHC22,ZDHHC23,ZDHHC14,ZDHHC19,ZDHHC11B,ACAT1,SPEG,CDC42BPG,TYW3,SIK1B,SNRK,CDC42BPA,GRK1,GRK5,NAA60,RPS20,STK19,FAM98B,CCNYL3,TRMT61B,naa10-naa15_human,CCAR1,TRIM52,RPS6KA6,RPS6KB2,UBE2O,POLG,GLYATL1,DGAT2L6,GLYATL1B,PI4KAP2,POLR1A,PLAAT4,PTPRT,RPL5,RPS7,MSL3,ZFYVE28,AIDA,CAMK2G,RPS6KA4,BIRC6,CDC20B,NSD3,DBI,UBA7,TGM1,POLR2E,TRIB2,FBXO2,PARP6,UBE2A,RNF138,TRIM63,LRSAM1,ARK2C,UBR4,RNF180,CCNB1IP1,TRIP12,RNF126,RNF26,MAB21L1,PTPRQ,CDK12,MCM3AP,PARM1,CAMKK2,UBE2T,AMFR,SYVN1,ABHD14B,CCNA1,CCNB3,CCNI,CCNO,CDKN2D,HERC2,CAMK2N1,ING3,CHRAC1,CALM1,LATS2,BORA,MAP4K3,MAP4K5,GSKIP,PRKRIP1,CERKL,LAS1L,ALPK1,FEM1A,TRMT2B,POLR1C,POLR1D,GNPTG,ANKRD54,TUSC3,MAP3K10,STK36,PIGA,STK10,NTMT1,PDSS1,PDSS2,FLT4,CHP1,PCGF5,dds_human,TAOK3,SGK3,MASTL,PRDM16,POLR2J,FBXL19,TPMT,DCAF7,FAM20C,PKDCC,SRPK1,POLR2H,PKIA,PKIG,DDR1,CAMK1,UGT8,PIK3R5,FBXO8,GAL3ST2,MSL2,RNF217,UBR1,RNF144B,RNF19A,RNF144A,ARIH1,UBR3,CENPE,ECT2,OSTC,LCP2,DYRK2,RNF139,NAA20,NAA30,POMK,SRM,ROR2,MUSK,TYRO3,CAMKMT,PDIK1L,STK35,SIK3,PRDM2,STK33,HHEX,UBE2Q1,CHTF18,METTL17,NAA50,POLR2F,RHBDD2,UGT1A5,PRKAG3,GALNT15,TRIO,RLIM,UBE2J2,DAPK1,LCK,PGAP4,UBIAD1,UNC119,SYAP1,TJP2,MVP,UBE2E1,ATG10,MERTK,DMPK,OTUB1,RIPK3,PRKX,PRKG2,CLK2,QTRT2,QTRT1,DTX3L,PRKAB1,CAB39,TRIM13,TRIM62,FZR1,RNF34,RFFL,TGFBR3L,TAOK2,ABL2,POLR2B,PRIM1,hras-sos1_human,ATP23,NPRL2,FGR,VRK2,MAPKAPK3,DYRK1B,RNF220,GALNT9,GALNT14,GALNT17,GALNT12,GALNT18,GALNT10,GALNT8,BLK,PRDM15,FRK,RNGTT,CACUL1,SCYL1,RNF212,UBE2L3,ARRDC3,TESK2,SLK,TXK,ETNK1,PCYT2,HS6ST3,CKM,HS6ST2,HS3ST4,RFPL4A,RFPL4AL1,RFPL3,RFPL2,TRIM58,TRIM68,TRIM34,MRM3,ATG16L1,MARVELD3,RNF2,CKS2,CKS1B,RUBCN,AURKC,ZDHHC9,ARAF,EPB42,VPS11,ZDHHC6,ANKIB1,WDR5,IL18,POLR1H,POLR3K,POLE,GPRC5A,RPL11,RPL23,KYAT3,POGLUT1,SFN,CDKN2A,RPS2,CAV3,PFKFB4,PFKFB3,MET,BCDIN3D,CAMK1D,NAB2,ROR1,PKN2,BAZ1B,CDK4,MAPK15,KLHDC1,WDR4,KLHDC2,KRTCAP2,PIGW,SPRY3,SKP1,RNF216,MARCHF2,TSSK2,TSSK3,TSSK6,TSSK1B,PAK2,N6AMT1,RPS6KA1,DUSP1,ZNF622,BRPF3,JADE2,DIRAS3,CSF1R,POLRMT,GXYLT1,UGT1A6,GXYLT2,WDR91,MMD2,PDGFRB,GSTZ1,BRD1,MAP4K2,CEP85,CCL3,UNKL,CUL2,MAP3K1,MAP3K8,NEK3,MGMT,DAPK2,ALPK2,PAK5,PIM3,POLD1,RPS6KA3,DKC1,DDR2,MCAT,MAP2K7,CAMK4,NEDD4L,RFPL1,PDPK1,PTK2B,LACC1,WDR81,VRK1,HERC1,POLR3H,AKTIP,MAGEL2,CRPPA,BRD4,ATIC,csf2-receptor_human,INSR,JADE1,TRMT2A,MRM1,SETMAR,PRDM8,TOM1L1,MAP4K4,ERBB3,CCND2,CCNY,UBXN8,CACYBP,PTK2,TNFSF15,RNF13,SH3RF3,RNF128,ZNRF4,DTX4,ZNRF3,RNF183,RNF167,msl_human,FAM98A,ADRA2C,PPP2R5A,ERBB4,LATS1,ASMT,ECE2,CDK10,CDKL3,CDK20,DYRK3,POLD4,ZDHHC3,SPRY4,CDK9,CDK13,ATG3,PIK3C2G,TRIM27,RPS15,PRDM13,RASIP1,NHLRC1,PIAS3,TRIM3,RBCK1,CDC42BPB,MGAT4A,TEK,PRKCQ,NME9,SAMD8,METTL18,MRPS36,KMT2C,B3GNT6,CDY2A,CDY1,PRMT2,LEP,METTL21C,MAGT1,VAC14,HEXIM1,GNAQ,MAT2A,TRMT11,TRMT10A,THUMPD3,TRMT1,POLE2,TRPC4AP,FBXW8,MEPCE,HIPK1,MAPK13,MAPK11,MYLK2,MOS,NLK,MYLK3,PCYT1A,FGFRL1,MINK1,DYRK1A,RPS6KA5,ANTKMT,PRMT1,TRIM8,ZBED1,SH3RF2,FBXL5,CAD,CBX8,SIAH2,SETD4,MDM4,SETD1A,PEX2,RGS14,KCTD13,KCTD10,SMCR8,RPUSD1,CHTF8,DIPK2A,PDGFRA,BFAR,NAA40,TRAF3IP2,RNMT,CARM1,SGK1,ING4,PKN1,IGF1R,KMT2B,CDK16,CDK

	6,TRIM37,REV3L,MED21,B3GNT3,SAT2,MTFMT,WEE1,DBF4B,DBF4,PILRB,VRK3,RNF213,GGT6,GGT7,TAFAZZIN,TLK2,DCLK1,TPX2,AKT3,FASN,BIRC7,NTRK1,EGFR,MAPT,ACAA1,B3GALNT2,B3GLCT,COQ5,XRCC1,PRKCD,ZDHHC11,ZDHHC20,PHPT1,UHRF1,TRIM5,METTL6,METTL2B,METTL2A,PJA2,MIB1,TRIM41,TRAIP,TRIM39,RNF5,SIAH1,TRIM72,DCST1,HECTD1,RFWD3,PRDM4,RNF41,TRIM44,SERTAD1,KLHL3,B4GALNT1,POFUT2,TSSK4,CARD10,wich_human,atg5-atg12_human,TAOK1,RNF187,RNF169,TRIM33,UBE2G2,CAMK2D,DUSP10,SETD5,SPTSSB,STK26,PARP16,FTSJ1,PYDC1,UBR2,morf1_human,moz1_human,DPY19L1,DPY19L3,DPY19L4,PIGP,PIGY,MARK2,MGAT2,ERBB2,TRIB1,PLK1,SRPK2,SPSB2,WWP2,DAD1,SPSB1,IRF2BPL,SOCS4,SETD6,AGAP2,AMHR2,CTU1,URS000075C8FA_9606,E4F1,MAP2K5,MRNIP,MRE11,FBXO5,NEDD4,CSNK2A3,nsl_human,HMBS,COQ8A,MMAB,C20orf173,TRIM22,RAG1,DVL3,CEMIP,CDKL5,DVL2,SPTSSA,LIPT2,MAP3K14,CARD14,PIGM,PIGV,NEDD9,NEK2,FBXO4,PRKCI,BIRC3,TGM2,PLK5,GYS1,SERINC1,MELK,ULK3,ube2n-ube2v2_human,IP6K3,ETAA1,ST6GALNAC2,JARID2,NDST4,NDST3,EPHA2,POMGNT1,FBXW7,TRIM21,MEFV,PRKCH,NUAK2,NAT8L,RGS2,B4GALNT2,LYN,PRDM9,DAXX,PTPRO,STUB1,B3GNT8,SPOP,MAP2K2,MAP2K1,HHAT,RNF4,ABL1,CCNE2,URS00004A7003_9606,URS00004416C5_9606,THG1L,SCYL2,FBXO22,NTRK2,PRKAR1A,MAPK7,PAK4,STK16,UBE2C,RNF19B,NSMCE2,ARIH2,COQ3,FTSJ3,MEAF6,TENT5C,TENT5B,TENT5A,MRM2,FBXO7,BRPF1,ALG2,NEK4,WEE2,POLG2,PTPRB,ESCO1,FOXA2,EIF4A2,RAD50,MAP2K3,ZAP70,CSK,YES1,FES,TRIM38,NOP2,ERN1,CDKAL1,CDK2,CAMK2B,WNK4,S100A12,DCAKD,CCNB2,AK7,TMT1A,PIP5K1B,P2RY12,USP22,SHARPIN,PIAS4,PRKACB,RNF31,PPP1R3F,pik3c3-uvrag_human,EPGN,PINK1,TKTL1,SASH1,SPDYA,ZDHHC1,ZDHHC21,ZDHHC18,TRIM15,PARP3,GGT5,RCHY1,GART,PRKCG,ERP29,CHMP6,BAG2,PPEF2,B3GNT5,NSUN4,KYAT1,PABPN1,g-atac_human,HAT1,B3GNT9,LINC02218,CISH,ELP3,CHFR,PTGES3,MPP1,WNK2,TRIM32,POLR2K,POLR2L,DBT,HTT,RNF8,UBE2D1,IPMK,MAP3K4,KIT,B3GAT2,PNKP,ccnk-cdk13_human,CIT,MYCBP2,AKT1,KDR,KEAP1,MAPRE3,GTF3C4,PRDM11,ATXN7L3,POMGNT2,PLK4,RIOK2,UHMK1,PIM2,PAK6,PRDM5,SH3RF1,RPS3,TRMT10B,CCNQ,CCNL1,CCNL2,NEK7,FER,SRC,MALT1,HHATL,BUB1B,AXL,POLI,B3GNT4,PSTK,CHML,TRIM31,MAGEC2,MAD2L1,KCTD11,TAF1L,NDUFAF5,POLD3,ZGPAT,COPS8,MAPK9,MAK,MAPKAPK2,ZDHHC16,GLYATL2,RET,TADA2A,DGKK,GYG1,B3GNT7,ABTB1,NT5C3A,AK6,CAMK2A,SRCAP,GTPBP4,RANGAP1,PRMT5,ALG1L2,TGS1,ASB2,ZMIZ1,NT5C2,DHDDS,CBL,CDKN2B,POLR3A,SOSTDC1,NVL,PTPN1,SMURF2,MAPK8,IHH,RPS6KA2,TTK,CLYBL,MGAT5B,LALBA,DPM2,KLHDC3,SPSB4,HCK,RBX1,ETFBKMT,GLYCTK,SEPHS1,CSNK1G1,TUT1,CDK1,TRIM56,RNF135,TRIM65,RNF125,RNF111,PBK,BUB1,ZDHHC15,POFUT1,B3GALNT1,UFL1,UBE3A,PIAS1,ulk1-atg13_human,PRKCA,GRK2,KDM6A,APP,POLR1B,ERN2,CBX5,ADARB1,ADAR,EMG1,PRKCB,IP6K1,GNE,AEBP2,MGA,POLN,CEPT1,CERK,CHST3,SGMS2,POLM,HASPIN,EFEMP1,MOCOS,UAP1,GAL3ST3,CHST13,GGPS1,FEM1C,DTX3,PAQR3,URS00004DC6C5_9606,RYK,XIAP,MGAT4B,LARGE2,MMS19,NAGK,CSNK1G3,CSNK1A1L,TESK1,B3GALT6,MDM2,PRKD2,UTY,GALNT2,PIH1D1,B3GALT9,CCNK,BAG5,SETD7,EPHB6,EPHA10,EPHA6,CARTPT,TRIM71,TRIM11,HUWE1,MAT1A,BCR,TELO2,ZDHHC17,ZDHHC7,POT1,CCNT2,DR1,MOCS2,CKB,DTL,ATG13,ZNF16,FYN,SIK1,CDK5R2,HLTF,EMP2,JAK1,DUSP22,ZNF675,KIF14,PFKFB2,METTL23,TNFRSF10B,VHL,ALG8,C1GALT1,C1GALT1C1,MGAT4C,STK11,MED30,MED17,POLR2I,PIK3C2B,UBE2N,GALNT1,GALNT4,TANK,FGFR2,MAP4K1,CDC23,UBR5,PELI1,B3GALT1,B3GALT4,B3GALT2,MST1,GNPTAB,hbo1-5.3_human,hbo1-5.2_human,hbo1-4.3_human,hbo1-4.2_human,PRDM12,HMBOX1,TNFAIP1,AGK,YKT6,TNFRSF10A,EFNA3,OBSCN,BMI1,GPRC5B,PAK1,METTL4,MAP3K7,PRPS1L1,PRPS2,PRPS1,WDR77,LCMT1,FEM1B,WWTR1,TAF11L5,TAF11L9,TAF11L12,TAF11L11,TAF11L14,TAF11L13,TAF11L4,TAF11L3,TAF11L2,PNMT,CHST7,CIAPIN1,CHST9,GALK1,GAL3ST4,HS3ST2,PPP2CA,GALNT13,GALNT16,GALNT6,RXYLT1,TRIM6,B3GALT5,MED27,MED7,MED6,KALRN,NFS1,CLOCK,ATG5,NUAK1,CKAP5,RNF168,HENMT1,GGT3P,ALDH18A1,PRKD1,SMURF1,RNF185,FZD10,HSP90AB1,SYK,UBE2I,FBLL1,PSRC1,CDC20,IL4,DCAF12,TSG101,PFKP,RAP2C,ABI1,TAF11L8,TAF11L10,TAF11L7,TAF11L6,ANAPC11,PIM1,MID2,URS00002DABE

	A_9606,NEK6,ULK2,LRRK1,SPRY2,ABHD4,TGFB1,MKRN2,CBLB,DNTT,ADRA2B,UGT1A10,GALNTL6,JMJD8,RNF14,IPO7,TIPARP,SMG1,MARK1,STK3,STK4,HSPB1,PTPRJ,CDK5RAP3,CHST5,TTBK1,MED18,MED10,MED11,MED31,MED8,MARK3,VPS72,NAA10,DPY30,A4GALT,CCNF,BARD1,URS0000565C8D_9606,BIRC2,ANKRD9,PAK3,STOX1,PORCN,ACVR1C,MAP3K20,pik3c3-atg14_human,NUDT5,CCND1,TBPL1,AK8,SMG6,HPF1,DUSP3,ZDHHC12,UGGT2,POLL,GALNT5,FUT5,GALNT7,ADRB2,SRCIN1,PIP5KL1,LRGUK,ACD,PLCE1,PIK3R4,PRKCZ,MAPK1,CDK5,MAPK3,LDB2,RAF1,ULK1,MAT2B,CCNH,RASSF2,GAMT,CHPT1,SULT1C3,HS3ST6,ETNK2,CDIPT,CKMT1A,TAMM41,CHST1,CKMT2,CDS2,HS3ST1,GALT,PRKG1,AURKA,BTK,STK24,PARK7,PIK3R6,CGAS,PINX1,DAPK3,EZH1,DDOST,GGT1,KMT2D,ccnt1-cdk9_human,DPAGT1,XYLT2,SLC11A1,MAD2L2,SPTLC3,NADK2,PANK3,TSTD1,PGM1,CHST14,CHST8,CHST15,ERC1,IQGAP1,GCNT3,EGR2,ATPSCKMT,KAT6A,EPHA5,PKMYT1,GTF2H4,XRCC6,SPRED1,SIRT4,GID8,PRIM2,SCLY,ITGB1BP1,ccnt2b-cdk9_human,ccnt2a-cdk9_human,RBL2,KLHDC10,MID1,AURKB,TSPYL2,SMC5,EP400,RALB,WNK3,ALG11,UGT1A9,ALG9,UGT1A4,XXYLT1,PGK1,HNRNPA2B1,UGT1A7,UBE2J1,GUCY2C,TYK2,JAK3,HIPK2,RAP2B,JAK2,ROBO1,B3GAT3,PRNP,TRNT1,saga-kat2a_human,tert-terc_human,NFX1,DGKZ,PARP10,NRP2,HTATIP2,CDKN1B,RPTOR,ACP4,BRD8,GTF2B,ERRFI1,tfiid_human,SOCS7,PRKCE,PNPLA2,LPCAT2,ALG3,RIOK3,PRDM1,TNIK,UBA2,PTPRC,CDC6,YEATS2,NEURL1,DTX1,MBOAT4,GADD45A,DCK,CDKN1A,ADRA2A,PUS7L,OSGEP,MAP3K3,NHERF1,UVRAG,ATF2,GATM,CHST10,BUD23,B4GALT7,SPRY1,HEG1,AGTR1,PML,SESN2,MGAT5,KAT8,TRAF4,DET1,ZDHHC2,LDB1,OXSM,CSNK2A1,BCKDK,MAP2K4,PGAM2,PI4KB,POLE3,SETD3,OXSR1,TRMT10C,NSD1,RAP1A,CDS1,NOL9,COMT,ALG1,ALG6,SUPT20HL2,SUPT20HL1,ALG12,PLAAT1,DPM1,SULT4A1,UBE2W,PFKFB1,GFPT2,LIMK1,RAG2,MAGEA2,CDC25A,TERF1,MAGI3,DGKA,DGKH,DGKB,DGKE,FASTK,MIDN,CDC7,PASK,COMMD1,ACVR2B,HADHA,TAB2,CSGALNACT2,EXTL1,PYGB,SAT1,GNPNAT1,GAS6,AGPAT5,CHSY3,ACAT2,LCLAT1,CHPF,AGPAT4,GBE1,CHPF2,AGPAT3,COLGALT1,STING1,TBP,PIN1,AKT2,RPS6KB1,CCL8,TAF1,ST6GALNAC4,QPCT,ALG10,COX10,GAL3ST1,HS3ST5,BCOR,tfiid-taf4bvariant_human,PYCARD,METTL5,DNMT3B,PIGB,ADAM9,CCNG1,CREBBP,PARP14,MAP2K6,HADHB,RASGRP1,AADAT,NSUN3,PAPSS1,PSMD10,DPY19L2,DPY19L2P2,KMT5A,POLR2A,PARP9,PGAM4,BPGM,PI4KA,ITPKC,RIPK1,RBKS,UGP2,EHMT1,RTRAF,RNF10,RNF6,XRCC5,DKK1,NAA80,FTCD,ZDHHC5,GCNT4,AMBRA1,GALNT3,MT-RNR1,DEFB114,ITCH,SUV39H2,FHIT,ROCK1,YRDC,AGPS,BRSK2,CASK,CHEK1,APC,A4GNT,MGAT1,CHRNA3,PMM1,PMM2,CHST4,PGM3,CHEK2,DVL1,RIPK2,IRAK1,SMYD1,DLST,CSNK1G2,ZCCHC4,METTL8,SUPT3H,RRP8,PRKACA,ST8SIA3,USP44,RAMAC,MNAT1,CDC34,MYC,NAT8,MAPK14,CPNE3,DERL1,PAAF1,ALOXE3,NDST2,PYGM,B4GALT4,KAT6B,SETDB1,ROCK2,DAB2IP,B3GAT1,GARS1,PDCD6,PHF10,EPHA8,TEX14,ST3GAL2,PFKL,BOD1,ILK,PLK2,DTNBP1,PLK3,PCIF1,PDK1,CCL5,CCNE1,MAX,NLRC5,TAF9,BMPR1B,FBL,NMRK1,KHK,HS3ST3A1,HS3ST3B1,B4GAT1,GALNT11,ESCO2,PIGZ,ST3GAL5,ST6GALNAC3,SERPINB3,TBK1,EEF1A2,OAT,TPK1,HS6ST1,FLAD1,MPST,TAT,IPO5,GUCY2D,RBL1,FBXO45,PTK7,CHI3L1,STK25,GRHL2,TK1,SIRT7,RFC2,RFC4,RFC3,RFC5,GTF2E1,SMYD2,TRAF3,DOT1L,NRG1,ACVR2A,hbo1-4.1_human,hbo1-5.1_human,GNMT,UBE2D3,IGF2R,FUT3,NAT8B,HGSNAT,PIK3R3,ILVBL,PARP2,TYMP,MBIP,PRDM14,BMP2,HSP90AA1,DRD4,BRSK1,ST8SIA5,ST8SIA6,ST6GALNAC1,PDCD10,BRCA2,NAA16,NAA15,UGGT1,CNOT4,MED12,HTR2B,CCT2,TCP1,CCT4,SIRT6,PAFAH1B2,AGT,CHD8,POLR3G,ALS2,POLA1,SETDB2,CCNT1,RFK,FIRRM,POLR3B,MAVS,WNK1,NNMT,EXTL2,HS2ST1,FCSK,PAPOLA,SULT1A2,CHST12,PGK2,GK,TRIM24,DAW1,nua4_human,IKBKB,BRAF,EPHA7,URS000020D84A_9606,ATR,POLR3F,POLR3C,XYLT1,TOP1,NCF1,IRAK3,TXN,PSAT1,EIF2AK1,TNKS,TTBK2,OGDHL,ST8SIA4,ERCC3,ADCYAP1,HMGA2,AGXT,PDXK,HNRNPD,BAAT,ACVR1B,UCHL1,PDCD4,UGT1A3,MUL1,SORL1,KMT2A,LAT,TCIM,TRIM28,DNAJA1,SPINDOC,CDKN1C,IRS1,TKFC,DPM3,PKD1,MYO3A,PIP5K1A,DLG3,CUL4A,ATG14,BRCA1,SENP3,GAK,UST,POLQ,ARHGEF5,GTF2H1,DGKI,UBASH3B,CSNK2B,SPTLC1,MTPAP,PANK1,RB1,KLHL25,TRAF2,AGL,RAD51,LAX1,RNASEL,CERS4,CERS3,CERS6,pdh_

	human,ABHD5,SOCS6,MBOAT7,CCNC,ACVRL1,TAF7L,CARD11,GRM5,ALAS1,IRGM,PPIA,ST6GALNAC6,SULT1B1,ZNFX1,UCK2,HK3,DGKG,NTF3,AK1,PAPSS2,EPHA4,ADAM17,PGAM1,SPTLC2,TTN,IKBKE,MYO3B,HDAC7,PIK3CB,FUT2,NCOA3,ST3GAL3,CCNB1,PLA2G4C,ACSM2B,UBE2K,METTL16,TAF7,B3GNT2,DCAF6,RHOA,WARS1,GLMN,CIB1,TRRAP,FBXW11,GAPDH,URS0000476BE1_9606,WASHC1,STK39,LIMK2,TNKS2,CCDC88A,QARS1,CSNK2A2,MCM3,DNMT3L,TRIM25,ALAS2,WRAP53,ALG10B,CPT1B,PDK2,SLC8A3,SLC8A2,IP6K2,DCAF13,ZSWIM8,HK1,CDC37,PI4K2A,AK2,ANG,UGT1A8,PIKFYVE,NPPA,ADAM8,UCKL1,COASY,NRP1,CHRNA7,DGKD,PIK3R1,TFAP4,PROX1,CDK5R1,NR2F2,AANAT,KAT7,CDC42,EXT2,MED20,TLR3,TENT2,GTF2E2,CTNNB1,HSD17B10,CIITA,ACTL9,EZH2,GTF2H2,PTEN,GHR,SPOUT1,PIK3C3,TNFAIP3,CLSPN,PTPN22,NDST1,TKT,EIF2AK2,PCNA,RC3H2,GFPT1,BHMT,PIK3R2,GH1,PLAAT3,TSC2,EGR1,PUS10,POLH,GUK1,SULT1E1,PIK3CG,NME7,PDGFC,USP33,PRKN,RELN,SOCS2,DLG2,MKLN1,CDC25C,TAF10,POLB,URS000040780F_9606,CLP1,PLOD3,ST3GAL6,MGAT4D,KAT2A,PRDX6,TAF9B,ST6GAL1,GCNT1,GLUL,RBBP4,NOX4,EPHA3,PRKDC,EEF1A1,LPCAT1,SMYD3,PLA2G1B,NME1,HADH,PRLR,SHMT2,TENM1,ST8SIA1,QPRT,NMRK2,MTR,NME4,SULT1A1,IPPK,AK3,NME3,IL34,SNX9,CPT1C,HDAC4,TALDO1,WNT5A,TNFRSF11A,PRDX3,PRPF19,CCNA2,GLYAT,GK2,PGM2,SNX6,MACROH2A1,BMPR1A,MEN1,TAF12,HAS1,OTULIN,CHUK,SUPT7L,CPT2,MYLK,NSUN5,MMUT,PDGFD,TDGF1,ATM,KLHL22,ACTL6B,BMPR2,MARCHF6, DT,TAF6,TAF6L,RAPGEF2,EHMT2,GTF2F1,PAXIP1,NMNAT2,PIP4K2B,UGCG,ACTBL2,EPHB3,DHPS,HEXA,ANAPC7,PDK3,TAF11,LRRK2,PRAMEF9,PRAMEF6,NPM1,SLC26A6,AXIN1,SMG8,LTF,FUT10,INPP5K,ZBTB7A,EIF2AK3,TGFBR3,UPRT,PKLR,SULT1A3,ARF4,ANAPC1,WDTC1,FBXL2,PDK4,SQSTM1,CUL1,AVP,FGF18,OTC,ASH1L,CSNK1D,CORO1C,SUV39H1,HRAS,FUT9,NSD2,EPHB1,KAT2B,RPUSD3,UBE2B,AZU1,PIK3C2A,TRIM16,HJV,PIGC,PIGH,PNPT1,CD200,TAF8,RC3H1,URS0000316FA5_9606,HYAL2,PKD2,FCGR1A,PAN3,BANF1,CALCA,NPR1,CSGALNACT1,TERF2,PIK3CA,PUM3,PFKM,MARK4,GTF2H3,FTO,GTF2A2,GTF2A1L,CD86,PYGL,TERT,URS00003768C5_9606,PUS7,RGCC,CHST11,TENT4A,SHMT1,SETD2,PDGFA,HSD17B12,SLC8A1,CD4,PEF1,ST3GAL4,E2F6,TADA1,NADK,CSNK1A1,ERCC2,DNMT3A,EFNB3,CD300A,EEF2K,USP47,UGT1A1,DMAP1,EFNA1,TARBP2,DDX3X,IFNG,SOCS5,RABGEF1,ZFP36,LILRA5,KMT5C,IRAK2,POTEKP,DLG4,SMAD7,NAF1,TADA3,PARN,CHSY1,AGPAT1,LRAT,ACVR1,PPP1R9B,ELANE,PIBF1,CUL3,CSNK1E,CD19,PIP4K2C,FUT1,FUT4,SLA2,SOCS3,GSK3B,GPR37,ACTL6A,OAS2,AK4,RUVBL2,EREG,MT3,TUT7,URS00005C2A6D_9606,TIRAP,GTF2A1,IKBKG,YEATS4,PHB2,THY1,FUT8,RUVBL1,KARS1,TRAF6,FERMT2,KMT5B,IL6R,DDB1,C5AR1,B4GALT1,TAF13,BLM,TADA2B,NAT10,EPHB2,GUCY2F,NME2,CAND1,GSK3A,POLR2M,GTF2F2,MARCHF7,MTAP,TAF4B,TINF2,SUPT20H,EDN3,FBH1,ATP2B4,SIRT2,URS0000424278_9606,URS00003D1AE3_9606,ANGPT1,HCFC1,METTL14,BAD,ABAT,BST1,CSF1,TLR6,TAF5L,NOD2,PIP5K1C,MTOR,TAF3,SPHK2,DGAT1,EXTL3,SUMO4,LILRB4,PIK3CD,HAS3,UBE2V1,URS000059273E_9606,BRCC3,URS00004C9052_9606,PHB1,RB1CC1,ACE,ADCY8,NME6,PRAME,OGDH,F2,B4GALT5,NCOA1,CCR7,IL23R,ITPKA,METTL3,NAMPT,CCL19,TYMS,CCN1,RPUSD4,POTEJ,POTEI,POTEF,NMNAT1,GCK,NAPRT,TIGAR,KAT5,HK2,AXIN2,TAF5,TAF2,TENT4B,HCFC2,PKM,GREM1,KDM6B,CCL2,ENY2,TRUB2,CD40,CCL21,LARGE1,SPHK1,KLF4,NSUN2,IGF2,IRS2,EP300,DNMT1,IRAK4,EIF2AK4,NPR2,INS,SNCA,ENG,LMO4,SOCS1,TNFSF11,FUT7,HGS,MYOCD,EEA1,THBS1,NF1,CEACAM1,AGPAT2,ATAT1,OAS3,OAS1,ITPKB,ST3GAL1,HNRNPU,OGT,DLG1,FGF2,SLAMF8,UPP1,ELOC,TUT4,ACTB,ST8SIA2,SMARCE1,NSMCE3,TAF4,HEXB,TGFB2,MED1,DLD,UPP2,PDGFB,HDAC2,PARP1,IL23A,POTEE,URS0000574A2C_9606,NCOA6,CPT1A,IFNB1,EXT1,IL12B,NME5,APRT,CERS2,ITGB3,IL1B,HYAL1,IGF1,GPAM,NBN,TNF,TREX1,HPRT1,VEGFA,PCK1,CD38,EDN1,TP53,HLA-DRB1,CD74,EPC1,ACTG1,TLR4,PNP,HAS2,ANAPC2,URS000039ED8D_9606,BECN1
Negative regulation of transporter activity	SLC39A10, SLC35C1, ABCA5, SLC6A4, SLC7A10, SLC7A5, SLC16A2, SLC7A11, ABCA12, SLC26A5, SLC18A2, AQP1, SLC6A3, NPC1, TRIAP1, EIF4ENIF1, SLC6A9, ABCG5, ABCG8, SLC1A1, ABCB7, SLC43A2, SLC2A10, ABCG1, SLC6A1, CLCN3, ABCA2, ABCA7, SLC43A1, ABCA1, URS000034979B_9606, URS000005CF5F_9606, URS00004E1410_9606, SLN, URS00004996E9_9606, URS000075B7E4_9606, TCAF2,

	URS0000155642_9606, KCNRG, TCAF2C, URS0000068B85_9606, PCTP, URS00003496BE_9606, URS000021202F_9606, URS00001D6BAE_9606, URS0000149452_9606, URS00000C7662_9606, SLC25A4, URS0000347D0F_9606, CBARP, abeta-40_human, HECW1, SLC25A6, URS0000476BE1_9606, CHP1, URS000024463E_9606, URS00004C9052_9606, ANO9, URS00000E5433_9606, URS0000192F9C_9606, URS000030BD69_9606, RHBDF2, URS00000A939F_9606, URS00004C8DD5_9606, CEACAM1, URS00002E857A_9606, TCAF1, CLIC2, SLC35F6, HECW2, URS0000432971_9606, URS0000483184_9606, ATRAID, URS000019907A_9606, CPTP, URS000059273E_9606, URS000019B0F7_9606, KCNE4, KCNE1B, abeta-40-42-oligomer_human, GPR35, FHL1, KCNAB1, URS000024A59E_9606, SLC24A3, APOD, URS000032BD73_9606, URS00004176D4_9606, URS000005A8ED_9606, NDFIP1, SLC35C2, URS00002075FA_9606, DRD3, FXYD5, CIDEA, CALM3, PRELID1, GOPC, SLC4A2, abeta-40-42_human, CALM2, MCL1, SLC25A27, abeta-42_human, UBQLN1, FKBP1B, FGF13, AQP11, ADIPOQ, SRI, URS000040DCFF_9606, PTPN3, SLC25A31, GHITM, CIDEC, SLC46A2, PRNP, SUMO1, YWHAE, RASA3, CABP1, PLN, KCNIP3, COA8, PSMD10, FXYD1, PPARG, TMSB4X, PCSK9, abeta-40-oligomer_human, URS00001DC04F_9606, MFSD2A, NPPA, KCNE2, TMC8, FMR1, FKBP1A, NNT, BEST3, ADRA2A, SLC25A5, DRD4, MFSD12, APOA2, NR1H3, KCNE5, GNB5, NEDD4L, GAL, ABCC8, TRPV3, WWP2, KCNE3, WNK2, GJC2, SLC24A1, SLC24A2, OTOP1, CAV3, TSPO, GRP, OSBPL8, FABP1, PEX14, STK39, ITGAV, PPP2CB, CCR2, IFNG, NDFIP2, ATP2B4, TRPC5, SLC4A1, CASQ2, HTR1B, PDE4D, SLC8A3, PTPA, KCNK6, TLR9, CALM1, TOMM70, GSTO1, KCNE1, UCP2, ATP2A2, PHPT1, BAG2, KCNK2, TRDN, EPO, CHRNA7, GSTM2, PPIF, INS, DMD, ACTN3, ATP2B1, PRKCD, SLC12A2, SLC8A1, EPHB2, OPRM1, ABCC2, ATP1A2, CTTNBP2NL, ANK3, VDAC2, TREM2, LETM1, BCL2A1, KCNJ11, TRPV4, SLC8B1, GJD3, ANXA2, YWHAZ, ABCD1, ID2, LRP6, CCL2, ATP1A1, GABRE, GPD1L, ACTN2, CHRNA10, CRH, KCNH2, MMP9, FABP4, CAV1, CIB1, JPH2, APOA1, RBP4, SLC30A1, HTT, MAPK8IP2, ATP9A, OSR1, SNTA1, NEDD4, ACE2, LYN, CAMK2D, GRIK2, KCNB1, SLC24A5, PHB2, ADAMTS8, ATP2A1, SHANK3, BAK1, BNIP1, PTK2B, TTPA, SLC11A1, ADRB2, ABCD2, GRIN2B, CRHR1, CAB39, REM1, YWHAH, GJA1, GSDME, DAPK1, VDAC1, OXSR1, PKD2, CETP, SHISA6, SLC39A8, ANO6, PRRT1, SLC27A1, WNK3, MCOLN2, ACTN4, DLG1, RXRA, CACNB4, ATAD1, ITPR1, SLC9A1, CTNS, HAP1, CD36, ATP13A2, PYCARD, SLC24A4, PLSCR1, GRIK3, ASIC1, MEF2C, APOE, DRD2, CLIC4, ABHD6, NHERF1, P2RX4, ATP1B2, SNCA, TMEM38A, AZGP1, TRPV1, PDE4B, ATP8B1, NOS1, FABP5, TESC, GRIN2C, NDUFA13, RIPK1, abeta-42-oligomer_human, TRPM4, GRIA4, ATP2A3, BAX, FYN, TPCN1, SLC22A13, NLGN1, BCL2, CRHBP, MCOLN3, PRKCE, SPHK2, APOM, GRIN2A, PIM1, PKD1, ITPR3, CTNNB1, KCNK3, DLG4, MCOLN1, GRM5, PSEN1, YES1, LRRK2, SRC, P2RX7, USP10, HERPUD1, ASIC2, EDN1, SLC39A14, DLG3, NDUFS3, SLC27A4, IGF1R, AP2M1, APP, KCNMA1, GLRA1, TMEM109, PRKACA, SLC40A1, FLNA, TRPA1, RYR2, ATP5F1B, SHANK1, KCNQ1, LRP2, ACTB, ANXA6, GRID2, RYR3, TPCN2, ITGB1, CHRNB2, ITPR2, PEX2, GJA5, GRIN3A, TMEM38B, ATP7A, KCNA5, ATP8A2, GSDMD, ATP5F1A, TRPM2, APOA4, GRIA1, CACNA1S, HSPA2, ABCC9, RYR1, PKP2, PPP1R9B, MICU2, GJB6
Cholesterol metabolic process	CH25H,CYP46A1,ABCA5,NCEH1,NPC2,NPC1,PLA2G15,LCAT,NPC1L1,CYP11A1,TTC39B,STARD4,CES1,CETP,ABCG1,DHCR7,SOAT2,EEPD1,EBP,SC5D,ABCG4,ARV1,APOF,ABCA8,GRAMD1A,anxa2-pcsk9_human,STAR,URS000075B7E4_9606,OSBPL2,PON1,URS0000251D0B_9606,LDLRAP1,SCAP,APOM,URS00004C8DD5_9606,SOAT1,CYP8B1,OSBPL5,URS00003BBF48_9606,PLTP,SCP2,APOA2,ABCA1,FDPS,CYP7A1,CYP39A1,IDI2,IDI1,FDFT1,URS00002E628F_9606,ERLIN2,ERLIN1,MSMO1,HMGCS1,OSBPL1A,LSS,PNLIP,URS0000483184_9606,LPCAT3,STARD3,PMVK,APOBR,URS00002367FA_9606,HSD17B7,SULT2B1,SREBF2,APOB,SERAC1,TM7SF2,URS00002C0FCB_9606,URS000024B619_9606,APOC3,LIMA1,APOA1,SCARB1,LIPG,ABCA2,ZDHHC8,URS000037C5A8_9606,HDLBP,ENPP7,URS0000070CD2_9606,OSBPL7,CEL,CYP27A1,PLA2G12B,VPS51,DISP3,SEC14L2,

	LIPC,MVD,MVK,DHCR24,URS00001CC379_9606,OSBPL6,SNX17,LBR,LDLR,APOA4,PCSK9,URS000016FF9C_9606,URS000059311D_9606,URS000034B6F5_9606,APOC2,EPHX2,MSR1,PLPP6,APOC1,URS00001B8558_9606,LIPE,APOL2,FDXR,OSBPL10,AKR1D1,NAXE,CYP51A1,URS00004176D4_9606,URS000019907A_9606,MALRD1,OSBPL3,NUS1,MBTPS1,ABCA12,URS000024A59E_9606,CYB5R3,MTTP,LRP6,ACLY,NR1H3,STX12,LIPA,HMGCR,NSDHL,SCARF1,SULT2A1,LAMTOR1,PLA2G10,ABCA7,URS00005A8080_9606,RXRA,ACSM1,URS00004E0808_9606,NR1H2,ABCA3,ANGPTL3,URS00004208C5_9606,ACSM3,CLN6,AKR1C1,G6PC1,GPS2,CYP2C9,GBA2,FDX1,SREBF1,TSKU,FMO5,APOE,SQLE,SERPINA12,PTCH1,NFE2L1,URS000013D17D_9606,MBTPS2,RALY,ACAA2,INSIG1,CYP7B1,ARL8B,APOA5,CYP11B1,ANXA2,DGAT2,LPL,PIP4P1,CUBN,CYP1A2,CYP2D6,FGF1,G6PD,LRP1,CYP3A4,URS0000527F89_9606,PPARD,FABP3,URS00003B95DA_9606,URS00002075FA_9606,PIP4K2A,MYLIP,APOL1,HMGCS2,PPARG,GNB3,PROM2,OSBP,PLSCR3,NFKBIA,MED13,AQP8,TSPO,CNBP,PRKAA1,ACADL,INSIG2,ABCB4,OSBPL8,HSD3B1,GPIHBP1,HSD3B2,NR5A2,ACADVL,SOD1,SPG11,CYP11B2,GPLD1,VPS54,EGF,PRKAA2,TPCN2,LRP5,CD36,CFTR,VAPA,DGKQ,URS000019B0F7_9606,ACAT1,LRP8,INHBB,FABP4,DHH,ADIPOQ,URS0000272039_9606,LEPR,HNF4A,VLDLR,CAV1,CLN8,FGFR4,NFKB1,F7,FECH,NR0B2,ABCB11,SCARB2,INHBA,SMPD1,SIRT1,CAT,RORA,CLU,APOD,TREM2,URS0000028BB8_9606,VPS4A,HDAC9,VDAC1,PPARA,NR1H4,SMAD2,SHH,GBA1,FURIN,URS00000E5433_9606,CEBPA,URS000030BD69_9606,LEP,CD24,NR1D1,DAG1,URS000050B527_9606,XBP1,SMO,TGFBR2,IHH,TGFBR1,VPS4B,IL4,APP,IL18,CD81,URS0000338542_9606,CAV3,TGFB1,CCL3
Macrophage differentiation	CSF1,MAEA,MMD,CSF2,MSR1,MMD2,CSF1R,SLC11A2,GAB3,IL34,CXCL17,TAFA3,PDE1B,URS000052F380_9606,AKIRIN1,CMKLR1,DCSTAMP,SLAMF8,URS00007E4AF8_9606,PAEP,OCSTAMP,URS000024A59E_9606,C5AR1,IL1RL1,ADGRF5,CNN2,ZBTB46,IL31RA,C1QC,ABCA5,CETP,IL17D,CRP,ETV3,URS00002C0FCB_9606,IL33,PF4,CYP19A1,RARRES2,MSTN,TSPAN2,TREM2,TLR2,TRPV4,PTK2,PTPN2,CCL3,URS0000377E71_9606,FGL2,PANX1,CD74,SBNO2,URS00002620A7_9606,SLAMF1,PLA2G10,GRN,TYROBP,TNFSF18,ROR2,EDN2,AZU1,TLR4,MAPK1,CX3CL1,MAPK3,LIF,BCR,IL13,CD4,CASP8,PLCG2,MDK,NR1H3,C1QA,NRROS,IL18,SOAT2,L3MBTL3,CX3CR1,GPR137B,URS0000315338_9606,URS0000605E00_9606,INHA,PRLR,LPL,TTBK1,ADAM9,GPRC5B,ALOX15B,APOB,HCLS1,CD34,IRF7,MMP2,MAPK9,ABCG1,PRKCH,P2RY12,PLA2G5,AGTR1,ZFP36L2,PLA2G2A,TRIB1,MMP8,SEMA7A,URS000020BE6A_9606,TLR3,PLA2G3,CEBPA,TLR9,URS0000338542_9606,ALPL,UBE2J1,CASP10,ADIPOQ,SYK,CEBPE,NKX2-3,CD36,B4GALT1,IFNGR1,SOAT1,EIF2AK1,ISL1,URS00004176D4_9606,FER,CD80,NDP,LDLR,PDE2A,IL4R,ITGAV,NAGLU,CXCR4,ID2,IL23R,LGALS3,RASGRP1,ABCA1,NFKBIA,MMP14,NPR2,PRKCA,TGFB1,WNT5A,SIRT1,MYD88,IL4,IFNG,PPARG,INHBA,GATA2,AGT,HSF1,FADD,RIPK1,ZFP36,CCR2,NR1H2,FCGR2B,TWIST1,GBA1,TGFB3,TREX1,LILRB1,EDNRB,NFKB1,HLA-G,CLU,CCL2,CD81,AXL,FOXP1,NR1D1,IL23A,URS000059273E_9606,CD9,BAP1,ITGAM,JMJD6,IL15,MFHAS1,LARGE1,BGLAP,FOSL2,URS000018C928_9606,CTSL,SELENOK,SPP1,URS00000B7E30_9606,ITGB3,APP,PTK2B,PTPRC,TGFB2,RB1,IL12B,RIPK2,MEF2C,ZC3H12A,IL10,PPARA,ITGB2,LIPA,NR3C1,VPS54,ANGPT1,RORA,UCP2,SOCS1,abeta-42-oligomer_human,JAK2,TNF,KCNJ8,MMP9,AGER,TRPV1,MAPT,CDC42,SPI1,STAT1,AKT1,LRRK2,IL1B,HLA-DRB1,ATM,EP300,PARP1,GATA3,BMP4,IL6,VEGFA
Regulation of macrophage differentiation	CSF1,MMD,CSF2,MAEA,MMD2,CSF1R,MSR1,SOCS1,STAT1,IL34,TAFA3,CXCL17,AKIRIN1,PAEP,URS000024A59E_9606,URS000052F380_9606,SLAMF8,CMKLR1,OCSTAMP,GAB3,URS00007E4AF8_9606,URS00002C0FCB_9606,C5AR1,ZBTB46,IL17D,CRP,IL1RL1,IL31RA,IL33,URS00002620A7_9606,MSTN,URS0000377E71_9606,DCSTAMP,PF4,SLAMF1,PTPN2,URS0000605E00_9606,GPR137B,PDE1B,TREM2,IL13,TNFSF18,TYROBP,HCLS1,C1QC,CNN2,GRN,CD74,ETV3,URS000020BE6A_9606,CCL3,MDK,SEMA7A,TLR2,TRPV4,TRIB1,FGL2,LIF,AZU1,IL18,ADGRF5,ABCA5,CETP,IL23R,URS0000315338_9606,TTBK1,NR1H3,L3MBTL3,CYP19A1,PTK2,CD4,IFNG,ROR2,SBNO2,CX3CL1,URS0000338542_9606,IRF7,RARRES2,PLCG2,PLA2G10,TLR9,URS00000B7E30_9606,IL4,URS00004176D4_9606,ADAM9,AGTR1,PLA2G5,CX3CR1,ADIPOQ,P2RY12,RASGRP1,INHA,IL4R,IL23A,CD80,AGT,PANX1,CASP8,GPRC5B,CC

	R2,BCR,SYK,PRKCH,EDN2,IL15,FCGR2B,PRLR,TLR4,MAPK9,LGALS3,URS000059273E_9606,LILRB1,PPARG,ABCG1,TLR3,ID2,PRKCA,ZFP36L2,FADD,ANGPT1,TGFB1,LDLR,GATA2,PLA2G3,SPP1,URS000018C928_9606,HLA-G,INHBA,WNT5A,TWIST1,MMP14,CD36,NR1H2,MAPK3,NFKBIA,FER,RIPK1,PLA2G2A,PTPRC,LPL,CLU,IL10,CEBPA,ALOX15B,CD81,CCL2,EIF2AK1,ZC3H12A,IL12B,ITGAV,MAPK1,SIRT1,ITGAM,SELENOK,MMP8,AGER,TGFB3,MFHAS1,CXCR4,PTK2B,ISL1,FOXP1,CD34,TNF,CASP10,NRROS,SPI1,GBA1,ZFP36,NFKB1,NR1D1,abeta-42-oligomer_human,IFNGR1,MEF2C,RIPK2,HSF1,UBE2J1,TGFB2,AXL,MAPT,AKT1,PPARA,IL1B,MYD88,APP,ITGB2,APOB,RB1,NKX2-3,PDE2A,ITGB3,EDNRB,CEBPE,BAP1,MMP2,SOAT1,HLA-DRB1,MMP9,BGLAP,JAK2,ABCA1,TREX1,LRRK2,FOSL2,RORA,B4GALT1,C1QA,NR3C1,ATM,JMJD6,CDC42,BMP4,NPR2,CD9,IL6,PARP1,CTSL,VEGFA,EP300,NDP,UCP2,TRPV1,NAGLU,GATA3,SOAT2,ALPL,LARGE1,LIPA,KCNJ8,VPS54
Cellular response to cholesterol	GPR155,CH25H,CYP46A1,NPC1,NCEH1,NPC2,CYP11A1,NPC1L1,CES1,GRAMD1B,GRAMD1A,GRAMD1C,ABCG5,ABCG8,ABCA1,ABCG4,EEPD1,MLC1,URS000024B619_9606,SCAP,CYP8B1,FDX1,APOM,URS00002367FA_9606,ABCG1,CYP7A1,OSBPL7,LAMTOR1,SREBF2,URS0000070CD2_9606,SCARB1,HMGCS2,LDLRAP1,APOA2,INHBB,F7,CYP11B1,ERLIN2,ERLIN1,LPCAT3,APOA4,PCSK9,LDLR,URS00001CC379_9606,PON1,PMVK,NFKBIA,MSR1,URS000024A59E_9606,URS000016FF9C_9606,LPL,LRP6,NR1H3,PLSCR3,APOA1,INSIG1,CAT,MBTPS1,URS000037C5A8_9606,SREBF1,ABCA2,AQP8,URS00002C0FCB_9606,FABP4,URS00004E0808_9606,LIPG,APOB,GPLD1,CCR5,PRKAA2,URS00004176D4_9606,RXRA,CYP7B1,MTTP,DHCR24,PRKAA1,CYP11B2,LIPA,PLPP6,INSIG2,URS000059311D_9606,SCARF1,TSPO,ABCB4,PLA2G10,ARL8B,VAPB,URS0000483184_9606,URS00002075FA_9606,APOC3,PPARG,EHD1,ACAA2,PPARD,YJEFN3,MVK,HSD3B1,HSD3B2,GPS2,FECH,URS0000527F89_9606,MBTPS2,NFKB1,PTCH1,URS000034B6F5_9606,SNX17,VPS54,CD36,LRP1,AKR1C1,ABCA7,NR1H2,XBP1,DISP3,NUS1,CFTR,APOL2,URS000013D17D_9606,SLC38A9,ADIPOQ,SYT7,G6PD,SOD1,ABCA3,FGF1,URS000019907A_9606,SMPD1,EPHX2,TREM2,TPCN2,RORA,LRP8,ABCA12,APOL1,URS00005A8080_9606,NR5A2,GPIHBP1,DGAT2,SYP,INHBA,CAV1,DAG1,APOE,HDAC9,DHH,SIRT1,CYP1A2,SQLE,ACSM1,SULT2A1,ANGPTL3,URS000050B527_9606,CYP2C9,URS000030BD69_9606,FMO5,NSDHL,NR1H4,CUBN,TSKU,HMGCR,URS000019B0F7_9606,PIP4P1,URS00003B95DA_9606,URS00004208C5_9606,CLU,NR1D1,GBA1,SERPINA12,PPARA,ANXA2,SMAD2,URS0000338542_9606,CCL3,LEP,PIP4K2A,DGKQ,ABCB11,URS0000272039_9606,CYP2D6,CD24,HNF4A,LEPR,IL18,OSBPL8,APOD,CYP3A4,CEBPA,IL4,LRP5,CD81,ACADVL,GNB3,NR0B2,FURIN,VPS4A,VDAC2,VDAC1,ACAT1,FGFR4,ANXA6,TGFBR1,EGF,VAPA,SMO,TGFB1,APP,URS0000028BB8_9606,TGFBR2,OSBP,VLDLR,SHH,CAV3,VPS4B,URS00000E5433_9606,IHH
