# Supplementary material for: Ex vivo expanded human regulatory T cells promote cholesterol efflux and PON1 expression in oxLDL-exposed macrophages via gap junction-mediated cAMP transfer
Source: Front Immunol. 2025 Oct 16;16:1662925. doi: 10.3389/fimmu.2025.1662925 (PMC12571657; doi:10.3389/fimmu.2025.1662925)
Supplement: Supplementary file 3 [file DataSheet3.pdf]

Supplemental Table S3

	GENES AFFECTED BY $TREG_{exp}$ ASSOCIATED TO BIOLOGICAL PROCESSES IN M_{IL4}		
**HGNC symbol**	**pathway**	**log2 Fold Change**	**Ensembl gene ID**
GPR155	cell_response_to_cholesterol	1.137864	ENSG00000163328
NPC2	cell_response_to_cholesterol	-0.91739	ENSG00000119655
GRAMD1A	cell_response_to_cholesterol	0.922803	ENSG00000089351
ABCA1	cell_response_to_cholesterol	0.623298	ENSG00000165029
EEPD1	cell_response_to_cholesterol	-1.88662	ENSG00000122547
FDX1	cell_response_to_cholesterol	-1.19122	ENSG00000137714
LAMTOR1	cell_response_to_cholesterol	-0.80405	ENSG00000149357
PMVK	cell_response_to_cholesterol	-1.10687	ENSG00000163344
NFKBIA	cell_response_to_cholesterol	1.65517	ENSG00000100906
NR1H3	cell_response_to_cholesterol	-1.39007	ENSG00000025434
ABCA2	cell_response_to_cholesterol	0.596558	ENSG00000107331
FABP4	cell_response_to_cholesterol	-2.4695	ENSG00000170323
DHCR24	cell_response_to_cholesterol	-0.95097	ENSG00000116133
PRKAA1	cell_response_to_cholesterol	1.311755	ENSG00000132356
TSPO	cell_response_to_cholesterol	-1.06125	ENSG00000100300
PPARG	cell_response_to_cholesterol	-0.92835	ENSG00000132170
ACAA2	cell_response_to_cholesterol	-0.82702	ENSG00000167315
PPARD	cell_response_to_cholesterol	0.875409	ENSG00000112033
GPS2	cell_response_to_cholesterol	-0.79119	ENSG00000132522
NFKB1	cell_response_to_cholesterol	0.936428	ENSG00000109320
CD36	cell_response_to_cholesterol	-1.30979	ENSG00000135218
SLC38A9	cell_response_to_cholesterol	0.858368	ENSG00000177058
SOD1	cell_response_to_cholesterol	-1.32631	ENSG00000142168
SMPD1	cell_response_to_cholesterol	-0.84795	ENSG00000166311
TREM2	cell_response_to_cholesterol	-1.5219	ENSG00000095970
APOL1	cell_response_to_cholesterol	1.240353	ENSG00000100342
INHBA	cell_response_to_cholesterol	4.609635	ENSG00000122641
NSDHL	cell_response_to_cholesterol	-1.07648	ENSG00000147383
ANXA2	cell_response_to_cholesterol	-1.21063	ENSG00000182718
LEP	cell_response_to_cholesterol	-3.18135	ENSG00000174697
VDAC2	cell_response_to_cholesterol	-0.46864	ENSG00000165637
VDAC1	cell_response_to_cholesterol	-0.64433	ENSG00000213585
ANXA6	cell_response_to_cholesterol	-1.48459	ENSG00000197043
SMO	cell_response_to_cholesterol	2.20763	ENSG00000128602
TGFB1	cell_response_to_cholesterol	-0.64135	ENSG00000105329
ANXA6	cholesterol_binding	-1.48459	ENSG00000197043
VDAC1	cholesterol_binding	-0.64433	ENSG00000213585
NR1H3	cholesterol_binding	-1.39007	ENSG00000025434
NPC2	cholesterol_binding	-0.91739	ENSG00000119655
SCARB2	cholesterol_binding	-1.38409	ENSG00000138760
VDAC2	cholesterol_binding	-0.46864	ENSG00000165637

GPR155	cholesterol_binding	1.137864	ENSG00000163328
GRAMD1A	cholesterol_binding	0.922803	ENSG00000089351
ABCA1	cholesterol_binding	0.623298	ENSG00000165029
TSPO	cholesterol_binding	-1.06125	ENSG00000100300
SLC38A9	cholesterol_binding	0.858368	ENSG00000177058
STARD3NL	cholesterol_binding	-0.72542	ENSG00000010270
ABCA5	cholesterol_efflux	0.763743	ENSG00000154265
NPC2	cholesterol_efflux	-0.91739	ENSG00000119655
EEPD1	cholesterol_efflux	-1.88662	ENSG00000122547
ABCA1	cholesterol_efflux	0.623298	ENSG00000165029
ABCA2	cholesterol_efflux	0.596558	ENSG00000107331
LAMTOR1	cholesterol_efflux	-0.80405	ENSG00000149357
APOC1	cholesterol_efflux	-1.07522	ENSG00000130208
NR1H3	cholesterol_efflux	-1.39007	ENSG00000025434
GPS2	cholesterol_efflux	-0.79119	ENSG00000132522
SPG11	cholesterol_efflux	0.546815	ENSG00000104133
NFKBIA	cholesterol_efflux	1.65517	ENSG00000100906
PPARG	cholesterol_efflux	-0.92835	ENSG00000132170
TREM2	cholesterol_efflux	-1.5219	ENSG00000095970
ABCA5	cholesterol_homeostasis	0.763743	ENSG00000154265
NPC2	cholesterol_homeostasis	-0.91739	ENSG00000119655
ABCA1	cholesterol_homeostasis	0.623298	ENSG00000165029
ABCA2	cholesterol_homeostasis	0.596558	ENSG00000107331
COMMD9	cholesterol_homeostasis	-1.10147	ENSG00000110442
LAMTOR1	cholesterol_homeostasis	-0.80405	ENSG00000149357
NR1H3	cholesterol_homeostasis	-1.39007	ENSG00000025434
PPARG	cholesterol_homeostasis	-0.92835	ENSG00000132170
PRKAA1	cholesterol_homeostasis	1.311755	ENSG00000132356
PPARD	cholesterol_homeostasis	0.875409	ENSG00000112033
SOD1	cholesterol_homeostasis	-1.32631	ENSG00000142168
TSPO	cholesterol_homeostasis	-1.06125	ENSG00000100300
FABP3	cholesterol_homeostasis	-1.38995	ENSG00000121769
CD36	cholesterol_homeostasis	-1.30979	ENSG00000135218
FABP4	cholesterol_homeostasis	-2.4695	ENSG00000170323
ANXA6	cholesterol_homeostasis	-1.48459	ENSG00000197043
TREM2	cholesterol_homeostasis	-1.5219	ENSG00000095970
INHBA	cholesterol_homeostasis	4.609635	ENSG00000122641
CLN8	cholesterol_homeostasis	0.762424	ENSG00000182372
SMO	cholesterol_homeostasis	2.20763	ENSG00000128602
LEP	cholesterol_homeostasis	-3.18135	ENSG00000174697
ABCA1	cholesterol_import	0.623298	ENSG00000165029
APOBR	cholesterol_import	-0.74302	ENSG00000184730
LAMTOR1	cholesterol_import	-0.80405	ENSG00000149357
NR1H3	cholesterol_import	-1.39007	ENSG00000025434
SPG11	cholesterol_import	0.546815	ENSG00000104133
NFKBIA	cholesterol_import	1.65517	ENSG00000100906

FABP3	cholesterol_import	-1.38995	ENSG00000121769
CD36	cholesterol_import	-1.30979	ENSG00000135218
SCARB2	cholesterol_import	-1.38409	ENSG00000138760
TREM2	cholesterol_import	-1.5219	ENSG00000095970
VDAC1	cholesterol_import	-0.64433	ENSG00000213585
CLN8	cholesterol_import	0.762424	ENSG00000182372
SMO	cholesterol_import	2.20763	ENSG00000128602
LEP	cholesterol_import	-3.18135	ENSG00000174697
TGFB1	cholesterol_import	-0.64135	ENSG00000105329
ABCA5	cholesterol_metabolic_process	0.763743	ENSG00000154265
NPC2	cholesterol_metabolic_process	-0.91739	ENSG00000119655
PLA2G15	cholesterol_metabolic_process	-1.31821	ENSG00000103066
EEPD1	cholesterol_metabolic_process	-1.88662	ENSG00000122547
EBP	cholesterol_metabolic_process	-1.24847	ENSG00000147155
GRAMD1A	cholesterol_metabolic_process	0.922803	ENSG00000089351
ABCA1	cholesterol_metabolic_process	0.623298	ENSG00000165029
FDPS	cholesterol_metabolic_process	-1.06729	ENSG00000160752
MSMO1	cholesterol_metabolic_process	-0.98371	ENSG00000052802
PMVK	cholesterol_metabolic_process	-1.10687	ENSG00000163344
APOBR	cholesterol_metabolic_process	-0.74302	ENSG00000184730
ABCA2	cholesterol_metabolic_process	0.596558	ENSG00000107331
HDLBP	cholesterol_metabolic_process	-1.0295	ENSG00000115677
CYP27A1	cholesterol_metabolic_process	-1.54186	ENSG00000135929
MVD	cholesterol_metabolic_process	-1.00498	ENSG00000167508
DHCR24	cholesterol_metabolic_process	-0.95097	ENSG00000116133
LBR	cholesterol_metabolic_process	0.628152	ENSG00000143815
APOC1	cholesterol_metabolic_process	-1.07522	ENSG00000130208
NR1H3	cholesterol_metabolic_process	-1.39007	ENSG00000025434
NSDHL	cholesterol_metabolic_process	-1.07648	ENSG00000147383
LAMTOR1	cholesterol_metabolic_process	-0.80405	ENSG00000149357
GPS2	cholesterol_metabolic_process	-0.79119	ENSG00000132522
FDX1	cholesterol_metabolic_process	-1.19122	ENSG00000137714
ACAA2	cholesterol_metabolic_process	-0.82702	ENSG00000167315
ANXA2	cholesterol_metabolic_process	-1.21063	ENSG00000182718
PPARD	cholesterol_metabolic_process	0.875409	ENSG00000112033
FABP3	cholesterol_metabolic_process	-1.38995	ENSG00000121769
APOL1	cholesterol_metabolic_process	1.240353	ENSG00000100342
PPARG	cholesterol_metabolic_process	-0.92835	ENSG00000132170
NFKBIA	cholesterol_metabolic_process	1.65517	ENSG00000100906
TSPO	cholesterol_metabolic_process	-1.06125	ENSG00000100300
PRKAA1	cholesterol_metabolic_process	1.311755	ENSG00000132356
SOD1	cholesterol_metabolic_process	-1.32631	ENSG00000142168
SPG11	cholesterol_metabolic_process	0.546815	ENSG00000104133
CD36	cholesterol_metabolic_process	-1.30979	ENSG00000135218
FABP4	cholesterol_metabolic_process	-2.4695	ENSG00000170323
CLN8	cholesterol_metabolic_process	0.762424	ENSG00000182372

NFKB1	cholesterol_metabolic_process	0.936428	ENSG00000109320
SCARB2	cholesterol_metabolic_process	-1.38409	ENSG00000138760
INHBA	cholesterol_metabolic_process	4.609635	ENSG00000122641
SMPD1	cholesterol_metabolic_process	-0.84795	ENSG00000166311
TREM2	cholesterol_metabolic_process	-1.5219	ENSG00000095970
VDAC1	cholesterol_metabolic_process	-0.64433	ENSG00000213585
LEP	cholesterol_metabolic_process	-3.18135	ENSG00000174697
SMO	cholesterol_metabolic_process	2.20763	ENSG00000128602
TGFB1	cholesterol_metabolic_process	-0.64135	ENSG00000105329
NPC2	cholesterol_transfer_activity	-0.91739	ENSG00000119655
GRAMD1A	cholesterol_transfer_activity	0.922803	ENSG00000089351
ABCA1	cholesterol_transfer_activity	0.623298	ENSG00000165029
FDX1	cholesterol_transfer_activity	-1.19122	ENSG00000137714
TSPO	cholesterol_transfer_activity	-1.06125	ENSG00000100300
ALG5	cholesterol_protein_transferase_activity	-0.91761	ENSG00000120697
LANCL1	cholesterol_protein_transferase_activity	1.083481	ENSG00000115365
HPGDS	cholesterol_protein_transferase_activity	-2.92064	ENSG00000163106
GSTP1	cholesterol_protein_transferase_activity	-1.2052	ENSG00000084207
MGST3	cholesterol_protein_transferase_activity	-1.27425	ENSG00000143198
PLA2G15	cholesterol_protein_transferase_activity	-1.31821	ENSG00000103066
TMTC2	cholesterol_protein_transferase_activity	1.54695	ENSG00000179104
GSTO1	cholesterol_protein_transferase_activity	-1.40114	ENSG00000148834
RABGGTA	cholesterol_protein_transferase_activity	-0.87802	ENSG00000100949
SEPSECS	cholesterol_protein_transferase_activity	1.119417	ENSG00000109618
GTDC1	cholesterol_protein_transferase_activity	0.918686	ENSG00000121964
FDPS	cholesterol_protein_transferase_activity	-1.06729	ENSG00000160752
PMVK	cholesterol_protein_transferase_activity	-1.10687	ENSG00000163344
NEURL4	cholesterol_protein_transferase_activity	0.478946	ENSG00000215041
METTL9	cholesterol_protein_transferase_activity	0.473526	ENSG00000197006
NOSIP	cholesterol_protein_transferase_activity	-1.0338	ENSG00000142546
RNF44	cholesterol_protein_transferase_activity	1.337394	ENSG00000146083
ACAA2	cholesterol_protein_transferase_activity	-0.82702	ENSG00000167315
PCMTD2	cholesterol_protein_transferase_activity	0.773583	ENSG00000203880
PPARG	cholesterol_protein_transferase_activity	-0.92835	ENSG00000132170
WSB1	cholesterol_protein_transferase_activity	1.168833	ENSG00000109046
UBE3B	cholesterol_protein_transferase_activity	0.799787	ENSG00000151148
PRKAA1	cholesterol_protein_transferase_activity	1.311755	ENSG00000132356
RNF181	cholesterol_protein_transferase_activity	-1.11805	ENSG00000168894
NEK9	cholesterol_protein_transferase_activity	0.976887	ENSG00000119638
RSKR	cholesterol_protein_transferase_activity	1.260819	ENSG00000167524
FBXL14	cholesterol_protein_transferase_activity	1.041889	ENSG00000171823
RPS6KL1	cholesterol_protein_transferase_activity	-1.13131	ENSG00000198208
PCMT1	cholesterol_protein_transferase_activity	-0.68558	ENSG00000120265
RNF130	cholesterol_protein_transferase_activity	-0.8174	ENSG00000113269
PCMTD1	cholesterol_protein_transferase_activity	0.902433	ENSG00000168300
TBCK	cholesterol_protein_transferase_activity	1.009388	ENSG00000145348

BRAP	cholesterol_protein_transferase_activity	-0.4599	ENSG00000089234
DPH1	cholesterol_protein_transferase_activity	0.497393	ENSG00000108963
REV1	cholesterol_protein_transferase_activity	0.810268	ENSG00000135945
SOD1	cholesterol_protein_transferase_activity	-1.32631	ENSG00000142168
MATK	cholesterol_protein_transferase_activity	-1.04591	ENSG00000007264
CRIM1	cholesterol_protein_transferase_activity	1.326997	ENSG00000150938
DYRK4	cholesterol_protein_transferase_activity	-1.16734	ENSG00000010219
AREL1	cholesterol_protein_transferase_activity	1.151194	ENSG00000119682
KSR1	cholesterol_protein_transferase_activity	2.985758	ENSG00000141068
STK32C	cholesterol_protein_transferase_activity	-1.03035	ENSG00000165752
PEAK1	cholesterol_protein_transferase_activity	0.971348	ENSG00000173517
UBA3	cholesterol_protein_transferase_activity	0.68435	ENSG00000144744
LTN1	cholesterol_protein_transferase_activity	1.144581	ENSG00000198862
TPST2	cholesterol_protein_transferase_activity	-1.12629	ENSG00000128294
UBE2M	cholesterol_protein_transferase_activity	-0.83346	ENSG00000130725
PRKD3	cholesterol_protein_transferase_activity	1.483953	ENSG00000115825
TREM2	cholesterol_protein_transferase_activity	-1.5219	ENSG00000095970
STT3A	cholesterol_protein_transferase_activity	-0.70863	ENSG00000134910
CDK19	cholesterol_protein_transferase_activity	0.728176	ENSG00000155111
MARCHF8	cholesterol_protein_transferase_activity	1.333879	ENSG00000165406
RNF114	cholesterol_protein_transferase_activity	0.748501	ENSG00000124226
RNF38	cholesterol_protein_transferase_activity	0.688178	ENSG00000137075
SMPD1	cholesterol_protein_transferase_activity	-0.84795	ENSG00000166311
CCNG2	cholesterol_protein_transferase_activity	1.104074	ENSG00000138764
JTB	cholesterol_protein_transferase_activity	-0.97815	ENSG00000143543
CDK17	cholesterol_protein_transferase_activity	1.504594	ENSG00000059758
FBXO6	cholesterol_protein_transferase_activity	0.947098	ENSG00000116663
TRMO	cholesterol_protein_transferase_activity	-0.46119	ENSG00000136932
NRBP1	cholesterol_protein_transferase_activity	-0.73631	ENSG00000115216
CUL5	cholesterol_protein_transferase_activity	1.128284	ENSG00000166266
DSTYK	cholesterol_protein_transferase_activity	0.766741	ENSG00000133059
UBE2F	cholesterol_protein_transferase_activity	-0.63226	ENSG00000184182
PARP11	cholesterol_protein_transferase_activity	0.801692	ENSG00000111224
PRPF4B	cholesterol_protein_transferase_activity	0.523449	ENSG00000112739
STK38L	cholesterol_protein_transferase_activity	0.972316	ENSG00000211455
DUSP16	cholesterol_protein_transferase_activity	1.001142	ENSG00000111266
TSR3	cholesterol_protein_transferase_activity	-1.47567	ENSG00000007520
PRKAR2B	cholesterol_protein_transferase_activity	2.005077	ENSG00000005249
EPHA1	cholesterol_protein_transferase_activity	-1.88488	ENSG00000146904
ITK	cholesterol_protein_transferase_activity	1.732803	ENSG00000113263
UFC1	cholesterol_protein_transferase_activity	-0.78201	ENSG00000143222
CASS4	cholesterol_protein_transferase_activity	1.715289	ENSG00000087589
ZMIZ2	cholesterol_protein_transferase_activity	1.007758	ENSG00000122515
CALM3	cholesterol_protein_transferase_activity	-1.20135	ENSG00000160014
ALK	cholesterol_protein_transferase_activity	-2.56429	ENSG00000171094
COP1	cholesterol_protein_transferase_activity	-1.44533	ENSG00000143207

WWP1	cholesterol_protein_transferase_activity	0.687862	ENSG00000123124
NSMCE1	cholesterol_protein_transferase_activity	-1.1421	ENSG00000169189
SHPRH	cholesterol_protein_transferase_activity	0.959649	ENSG00000146414
PRDM10	cholesterol_protein_transferase_activity	1.251623	ENSG00000170325
TRMT112	cholesterol_protein_transferase_activity	-0.88203	ENSG00000173113
MAP3K2	cholesterol_protein_transferase_activity	1.225634	ENSG00000169967
MARCHF1	cholesterol_protein_transferase_activity	0.80815	ENSG00000145416
MGRN1	cholesterol_protein_transferase_activity	0.604237	ENSG00000102858
STK17B	cholesterol_protein_transferase_activity	1.327024	ENSG00000081320
STK40	cholesterol_protein_transferase_activity	-0.55466	ENSG00000196182
RPN2	cholesterol_protein_transferase_activity	-0.95856	ENSG00000118705
RPN1	cholesterol_protein_transferase_activity	-0.98153	ENSG00000163902
BCCIP	cholesterol_protein_transferase_activity	-0.8539	ENSG00000107949
ZDHHC19	cholesterol_protein_transferase_activity	-1.98137	ENSG00000163958
POLG	cholesterol_protein_transferase_activity	0.497917	ENSG00000140521
ZFYVE28	cholesterol_protein_transferase_activity	-1.0155	ENSG00000159733
DBI	cholesterol_protein_transferase_activity	-1.47403	ENSG00000155368
UBR4	cholesterol_protein_transferase_activity	-0.91749	ENSG00000127481
PARM1	cholesterol_protein_transferase_activity	3.1722	ENSG00000169116
ALPK1	cholesterol_protein_transferase_activity	0.795369	ENSG00000073331
POLR1C	cholesterol_protein_transferase_activity	-0.61901	ENSG00000171453
STK36	cholesterol_protein_transferase_activity	1.611383	ENSG00000163482
STK10	cholesterol_protein_transferase_activity	-0.63947	ENSG00000072786
PIK3R5	cholesterol_protein_transferase_activity	0.793254	ENSG00000141506
MSL2	cholesterol_protein_transferase_activity	0.606086	ENSG00000174579
NAA20	cholesterol_protein_transferase_activity	-0.40357	ENSG00000173418
POMK	cholesterol_protein_transferase_activity	0.685402	ENSG00000185900
SRM	cholesterol_protein_transferase_activity	-1.14066	ENSG00000116649
SIK3	cholesterol_protein_transferase_activity	0.743018	ENSG00000160584
POLR2F	cholesterol_protein_transferase_activity	-1.13604	ENSG00000100142
RHBDD2	cholesterol_protein_transferase_activity	1.847409	ENSG00000005486
RLIM	cholesterol_protein_transferase_activity	0.956782	ENSG00000131263
LCK	cholesterol_protein_transferase_activity	3.57631	ENSG00000182866
TJP2	cholesterol_protein_transferase_activity	1.530584	ENSG00000119139
DMPK	cholesterol_protein_transferase_activity	1.765299	ENSG00000104936
OTUB1	cholesterol_protein_transferase_activity	-0.6909	ENSG00000167770
QTRT2	cholesterol_protein_transferase_activity	1.057918	ENSG00000151576
ABL2	cholesterol_protein_transferase_activity	0.890799	ENSG00000143322
VRK2	cholesterol_protein_transferase_activity	0.629684	ENSG00000028116
GALNT14	cholesterol_protein_transferase_activity	-2.56894	ENSG00000158089
GALNT18	cholesterol_protein_transferase_activity	2.602534	ENSG00000110328
FRK	cholesterol_protein_transferase_activity	1.295234	ENSG00000111816
CACUL1	cholesterol_protein_transferase_activity	0.85046	ENSG00000151893
SCYL1	cholesterol_protein_transferase_activity	-0.97627	ENSG00000142186
UBE2L3	cholesterol_protein_transferase_activity	-0.64158	ENSG00000185651
ARRDC3	cholesterol_protein_transferase_activity	1.186284	ENSG00000113369

PCYT2	cholesterol_protein_transferase_activity	-1.05062	ENSG00000185813
TRIM34	cholesterol_protein_transferase_activity	1.061418	ENSG00000258659
MRM3	cholesterol_protein_transferase_activity	-0.69195	ENSG00000171861
RUBCN	cholesterol_protein_transferase_activity	0.910756	ENSG00000145016
ANKIB1	cholesterol_protein_transferase_activity	1.61151	ENSG00000001629
CAMK1D	cholesterol_protein_transferase_activity	1.045538	ENSG00000183049
KRTCAP2	cholesterol_protein_transferase_activity	-1.22119	ENSG00000163463
SKP1	cholesterol_protein_transferase_activity	-0.71054	ENSG00000113558
RPS6KA1	cholesterol_protein_transferase_activity	-0.51665	ENSG00000117676
ZNF622	cholesterol_protein_transferase_activity	-1.20989	ENSG00000173545
JADE2	cholesterol_protein_transferase_activity	1.090332	ENSG00000043143
POLRMT	cholesterol_protein_transferase_activity	-1.26482	ENSG00000099821
BRD1	cholesterol_protein_transferase_activity	0.662054	ENSG00000100425
MGMT	cholesterol_protein_transferase_activity	-1.12763	ENSG00000170430
PIM3	cholesterol_protein_transferase_activity	0.724545	ENSG00000198355
CAMK4	cholesterol_protein_transferase_activity	4.267439	ENSG00000152495
ATIC	cholesterol_protein_transferase_activity	-1.19946	ENSG00000138363
PRDM8	cholesterol_protein_transferase_activity	2.028398	ENSG00000152784
LATS1	cholesterol_protein_transferase_activity	0.638046	ENSG00000131023
PRKCQ	cholesterol_protein_transferase_activity	2.65819	ENSG00000065675
KMT2C	cholesterol_protein_transferase_activity	1.658419	ENSG00000055609
PRMT2	cholesterol_protein_transferase_activity	-0.45798	ENSG00000160310
LEP	cholesterol_protein_transferase_activity	-3.18135	ENSG00000174697
VAC14	cholesterol_protein_transferase_activity	-1.42119	ENSG00000103043
MAT2A	cholesterol_protein_transferase_activity	0.844286	ENSG00000168906
MINK1	cholesterol_protein_transferase_activity	1.109381	ENSG00000141503
DYRK1A	cholesterol_protein_transferase_activity	1.060849	ENSG00000157540
RPS6KA5	cholesterol_protein_transferase_activity	1.901201	ENSG00000100784
MDM4	cholesterol_protein_transferase_activity	0.906972	ENSG00000198625
PDGFRA	cholesterol_protein_transferase_activity	-1.63255	ENSG00000134853
NAA40	cholesterol_protein_transferase_activity	0.784103	ENSG00000110583
PKN1	cholesterol_protein_transferase_activity	-1.13799	ENSG00000123143
IGF1R	cholesterol_protein_transferase_activity	2.830037	ENSG00000140443
DBF4B	cholesterol_protein_transferase_activity	0.805445	ENSG00000161692
RNF213	cholesterol_protein_transferase_activity	1.113383	ENSG00000173821
AKT3	cholesterol_protein_transferase_activity	0.83688	ENSG00000117020
ACAA1	cholesterol_protein_transferase_activity	-0.60708	ENSG00000060971
MIB1	cholesterol_protein_transferase_activity	0.812352	ENSG00000101752
TRIM41	cholesterol_protein_transferase_activity	0.683716	ENSG00000146063
DCST1	cholesterol_protein_transferase_activity	2.401009	ENSG00000163357
RFWD3	cholesterol_protein_transferase_activity	0.992981	ENSG00000168411
RNF187	cholesterol_protein_transferase_activity	-0.75641	ENSG00000168159
SETD5	cholesterol_protein_transferase_activity	2.091132	ENSG00000168137
FTSJ1	cholesterol_protein_transferase_activity	-0.67908	ENSG00000068438
DAD1	cholesterol_protein_transferase_activity	-1.27877	ENSG00000129562
NEDD4	cholesterol_protein_transferase_activity	1.575732	ENSG00000069869

TRIM22	cholesterol_protein_transferase_activity	0.72993	ENSG00000132274
BIRC3	cholesterol_protein_transferase_activity	1.942923	ENSG00000023445
TGM2	cholesterol_protein_transferase_activity	1.562414	ENSG00000198959
NUAK2	cholesterol_protein_transferase_activity	1.23671	ENSG00000163545
B4GALNT2	cholesterol_protein_transferase_activity	1.775769	ENSG00000167080
STUB1	cholesterol_protein_transferase_activity	-0.99068	ENSG00000103266
B3GNT8	cholesterol_protein_transferase_activity	-2.55876	ENSG00000177191
MAP2K2	cholesterol_protein_transferase_activity	-1.30066	ENSG00000126934
RNF19B	cholesterol_protein_transferase_activity	1.999303	ENSG00000116514
TENT5C	cholesterol_protein_transferase_activity	2.980015	ENSG00000183508
TENT5A	cholesterol_protein_transferase_activity	-1.07523	ENSG00000112773
MRM2	cholesterol_protein_transferase_activity	-0.94772	ENSG00000122687
FBXO7	cholesterol_protein_transferase_activity	-0.35585	ENSG00000100225
ESCO1	cholesterol_protein_transferase_activity	-0.40695	ENSG00000141446
EIF4A2	cholesterol_protein_transferase_activity	0.531914	ENSG00000156976
SHARPIN	cholesterol_protein_transferase_activity	-0.67563	ENSG00000179526
ZDHHC21	cholesterol_protein_transferase_activity	2.171384	ENSG00000175893
ZDHHC18	cholesterol_protein_transferase_activity	0.731557	ENSG00000204160
PARP3	cholesterol_protein_transferase_activity	0.711835	ENSG00000041880
GGT5	cholesterol_protein_transferase_activity	4.609694	ENSG00000099998
RCHY1	cholesterol_protein_transferase_activity	0.556106	ENSG00000163743
ERP29	cholesterol_protein_transferase_activity	-1.09885	ENSG00000089248
CISH	cholesterol_protein_transferase_activity	2.656173	ENSG00000114737
MPP1	cholesterol_protein_transferase_activity	-0.97077	ENSG00000130830
POLR2L	cholesterol_protein_transferase_activity	-0.99607	ENSG00000177700
RIOK2	cholesterol_protein_transferase_activity	-0.82559	ENSG00000058729
PRDM5	cholesterol_protein_transferase_activity	1.624437	ENSG00000138738
NEK7	cholesterol_protein_transferase_activity	1.036364	ENSG00000151414
MALT1	cholesterol_protein_transferase_activity	0.969814	ENSG00000172175
CHML	cholesterol_protein_transferase_activity	1.467633	ENSG00000203668
SMURF2	cholesterol_protein_transferase_activity	0.984378	ENSG00000108854
MAPK8	cholesterol_protein_transferase_activity	1.047523	ENSG00000107643
RPS6KA2	cholesterol_protein_transferase_activity	-0.90372	ENSG00000071242
RBX1	cholesterol_protein_transferase_activity	-1.01495	ENSG00000100387
TRIM56	cholesterol_protein_transferase_activity	0.941678	ENSG00000169871
RNF135	cholesterol_protein_transferase_activity	-0.76566	ENSG00000181481
UBE3A	cholesterol_protein_transferase_activity	0.892206	ENSG00000114062
POLR1B	cholesterol_protein_transferase_activity	1.77909	ENSG00000125630
ADAR	cholesterol_protein_transferase_activity	0.626931	ENSG00000160710
EMG1	cholesterol_protein_transferase_activity	-0.79121	ENSG00000126749
MGA	cholesterol_protein_transferase_activity	0.907359	ENSG00000174197
NAGK	cholesterol_protein_transferase_activity	-1.77415	ENSG00000124357
C5AR1	mac_differentiation	-0.8793	ENSG00000197405
ZBTB46	mac_differentiation	1.052808	ENSG00000130584
ABCA5	mac_differentiation	0.763743	ENSG00000154265
ETV3	mac_differentiation	1.480386	ENSG00000117036

TREM2	mac_differentiation	-1.5219	ENSG00000095970
FGL2	mac_differentiation	2.493393	ENSG00000127951
SBNO2	mac_differentiation	1.210006	ENSG00000064932
SLAMF1	mac_differentiation	3.110996	ENSG00000117090
GRN	mac_differentiation	-0.81959	ENSG00000030582
TYROBP	mac_differentiation	-1.00089	ENSG00000011600
TLR4	mac_differentiation	1.023343	ENSG00000136869
CASP8	mac_differentiation	1.057433	ENSG00000064012
PLCG2	mac_differentiation	0.608006	ENSG00000197943
NR1H3	mac_differentiation	-1.39007	ENSG00000025434
L3MBTL3	mac_differentiation	0.917905	ENSG00000198945
GPR137B	mac_differentiation	-0.44612	ENSG00000077585
GPRC5B	mac_differentiation	-3.76032	ENSG00000167191
MMP8	mac_differentiation	-2.66998	ENSG00000118113
CEBPE	mac_differentiation	-2.22776	ENSG00000092067
CD36	mac_differentiation	-1.30979	ENSG00000135218
B4GALT1	mac_differentiation	0.573893	ENSG00000086062
ITGAV	mac_differentiation	1.007223	ENSG00000138448
LGALS3	mac_differentiation	-1.17888	ENSG00000131981
RASGRP1	mac_differentiation	2.430574	ENSG00000172575
ABCA1	mac_differentiation	0.623298	ENSG00000165029
NFKBIA	mac_differentiation	1.65517	ENSG00000100906
TGFB1	mac_differentiation	-0.64135	ENSG00000105329
WNT5A	mac_differentiation	3.761195	ENSG00000114251
PPARG	mac_differentiation	-0.92835	ENSG00000132170
INHBA	mac_differentiation	4.609635	ENSG00000122641
LILRB1	mac_differentiation	2.081649	ENSG00000104972
NFKB1	mac_differentiation	0.936428	ENSG00000109320
CD9	mac_differentiation	-1.17971	ENSG00000010278
IL15	mac_differentiation	0.965899	ENSG00000164136
CTSL	mac_differentiation	-1.83877	ENSG00000135047
RIPK2	mac_differentiation	0.692978	ENSG00000104312
ZC3H12A	mac_differentiation	1.18876	ENSG00000163874
IL10	mac_differentiation	-1.56348	ENSG00000136634
NR3C1	mac_differentiation	0.81588	ENSG00000113580
JAK2	mac_differentiation	1.28677	ENSG00000096968
MMP9	mac_differentiation	-0.48593	ENSG00000100985
CDC42	mac_differentiation	-0.24203	ENSG00000070831
STAT1	mac_differentiation	2.242366	ENSG00000115415
LRRK2	mac_differentiation	1.379778	ENSG00000188906
ATM	mac_differentiation	0.820685	ENSG00000149311
EP300	mac_differentiation	1.11703	ENSG00000100393
GATA3	mac_differentiation	3.296142	ENSG00000107485
VEGFA	mac_differentiation	1.664746	ENSG00000112715
ABCA5	neg_reg_chol_efflux	0.763743	ENSG00000154265
GPS2	neg_reg_chol_efflux	-0.79119	ENSG00000132522

ABCA2	neg_reg_chol_efflux	0.596558	ENSG00000107331
NR1H3	neg_reg_chol_efflux	-1.39007	ENSG00000025434
APOC1	neg_reg_chol_efflux	-1.07522	ENSG00000130208
PPARG	neg_reg_chol_efflux	-0.92835	ENSG00000132170
ABCA1	neg_reg_chol_efflux	0.623298	ENSG00000165029
NFKBIA	neg_reg_chol_efflux	1.65517	ENSG00000100906
TREM2	neg_reg_chol_efflux	-1.5219	ENSG00000095970
SLC35C1	neg_reg_transp_activity	-0.54373	ENSG00000181830
ABCA5	neg_reg_transp_activity	0.763743	ENSG00000154265
SLC7A11	neg_reg_transp_activity	1.450423	ENSG00000151012
TRIAP1	neg_reg_transp_activity	-0.86323	ENSG00000170855
ABCA2	neg_reg_transp_activity	0.596558	ENSG00000107331
ABCA1	neg_reg_transp_activity	0.623298	ENSG00000165029
RHBDF2	neg_reg_transp_activity	0.855604	ENSG00000129667
CLIC2	neg_reg_transp_activity	0.911759	ENSG00000155962
SLC35F6	neg_reg_transp_activity	-0.84761	ENSG00000213699
FXYD5	neg_reg_transp_activity	-1.84395	ENSG00000089327
CALM3	neg_reg_transp_activity	-1.20135	ENSG00000160014
SLC4A2	neg_reg_transp_activity	-1.33354	ENSG00000164889
MCL1	neg_reg_transp_activity	1.087924	ENSG00000143384
GHITM	neg_reg_transp_activity	-0.49576	ENSG00000165678
YWHAE	neg_reg_transp_activity	-0.56431	ENSG00000108953
FXYD1	neg_reg_transp_activity	-2.69118	ENSG00000266964
PPARG	neg_reg_transp_activity	-0.92835	ENSG00000132170
MFSD2A	neg_reg_transp_activity	-1.28344	ENSG00000168389
FMR1	neg_reg_transp_activity	1.040704	ENSG00000102081
NR1H3	neg_reg_transp_activity	-1.39007	ENSG00000025434
SLC24A1	neg_reg_transp_activity	0.998209	ENSG00000074621
TSPO	neg_reg_transp_activity	-1.06125	ENSG00000100300
PEX14	neg_reg_transp_activity	-0.51675	ENSG00000142655
ITGAV	neg_reg_transp_activity	1.007223	ENSG00000138448
PPP2CB	neg_reg_transp_activity	-0.48949	ENSG00000104695
GSTO1	neg_reg_transp_activity	-1.40114	ENSG00000148834
ATP2B1	neg_reg_transp_activity	0.890848	ENSG00000070961
SLC8A1	neg_reg_transp_activity	2.093358	ENSG00000183023
OPRM1	neg_reg_transp_activity	-0.96935	ENSG00000112038
VDAC2	neg_reg_transp_activity	-0.46864	ENSG00000165637
TREM2	neg_reg_transp_activity	-1.5219	ENSG00000095970
ANXA2	neg_reg_transp_activity	-1.21063	ENSG00000182718
MMP9	neg_reg_transp_activity	-0.48593	ENSG00000100985
FABP4	neg_reg_transp_activity	-2.4695	ENSG00000170323
NEDD4	neg_reg_transp_activity	1.575732	ENSG00000069869
GSDME	neg_reg_transp_activity	-0.78152	ENSG00000105928
VDAC1	neg_reg_transp_activity	-0.64433	ENSG00000213585
PKD2	neg_reg_transp_activity	1.584501	ENSG00000118762
SLC27A1	neg_reg_transp_activity	-0.77297	ENSG00000130304

ACTN4	neg_reg_transp_activity	0.561488	ENSG00000130402
CD36	neg_reg_transp_activity	-1.30979	ENSG00000135218
ATP13A2	neg_reg_transp_activity	-1.15334	ENSG00000159363
PLSCR1	neg_reg_transp_activity	1.568264	ENSG00000188313
ABHD6	neg_reg_transp_activity	-0.64607	ENSG00000163686
P2RX4	neg_reg_transp_activity	-0.98886	ENSG00000135124
PDE4B	neg_reg_transp_activity	2.794144	ENSG00000184588
FABP5	neg_reg_transp_activity	-1.94368	ENSG00000164687
NDUFA13	neg_reg_transp_activity	-1.28793	ENSG00000186010
TRPM4	neg_reg_transp_activity	0.690654	ENSG00000130529
TPCN1	neg_reg_transp_activity	0.461845	ENSG00000186815
CRHBP	neg_reg_transp_activity	-2.73991	ENSG00000145708
PIM1	neg_reg_transp_activity	1.457959	ENSG00000137193
PKD1	neg_reg_transp_activity	1.11983	ENSG00000008710
DLG4	neg_reg_transp_activity	0.628766	ENSG00000132535
MCOLN1	neg_reg_transp_activity	-1.40814	ENSG00000090674
LRRK2	neg_reg_transp_activity	1.379778	ENSG00000188906
NDUFS3	neg_reg_transp_activity	-1.21838	ENSG00000213619
SLC27A4	neg_reg_transp_activity	-1.01434	ENSG00000167114
IGF1R	neg_reg_transp_activity	2.830037	ENSG00000140443
FLNA	neg_reg_transp_activity	-0.89756	ENSG00000196924
ATP5F1B	neg_reg_transp_activity	-0.79556	ENSG00000110955
ACTB	neg_reg_transp_activity	-1.20911	ENSG00000075624
ANXA6	neg_reg_transp_activity	-1.48459	ENSG00000197043
ITPR2	neg_reg_transp_activity	1.468288	ENSG00000123104
ATP5F1A	neg_reg_transp_activity	-0.43717	ENSG00000152234
ABCA5	pos_reg_chol_efflux	0.763743	ENSG00000154265
EEPD1	pos_reg_chol_efflux	-1.88662	ENSG00000122547
GPS2	pos_reg_chol_efflux	-0.79119	ENSG00000132522
NR1H3	pos_reg_chol_efflux	-1.39007	ENSG00000025434
ABCA2	pos_reg_chol_efflux	0.596558	ENSG00000107331
ABCA1	pos_reg_chol_efflux	0.623298	ENSG00000165029
PPARG	pos_reg_chol_efflux	-0.92835	ENSG00000132170
LAMTOR1	pos_reg_chol_efflux	-0.80405	ENSG00000149357
TREM2	pos_reg_chol_efflux	-1.5219	ENSG00000095970
NFKBIA	pos_reg_chol_efflux	1.65517	ENSG00000100906
LRPAP1	pos_reg-ldl_clearance	-0.76613	ENSG00000163956
CD36	pos_reg-ldl_clearance	-1.30979	ENSG00000135218
ANXA2	pos_reg-ldl_clearance	-1.21063	ENSG00000182718
TREM2	pos_reg-ldl_clearance	-1.5219	ENSG00000095970
HMOX1	pos_reg-ldl_clearance	-0.99375	ENSG00000100292
PICALM	pos_reg-ldl_clearance	0.869392	ENSG00000073921
LRPAP1	pos_reg_ldlr_activity	-0.76613	ENSG00000163956
TREM2	pos_reg_ldlr_activity	-1.5219	ENSG00000095970
PLA2G7	pos_reg_ldlr_activity	-0.72052	ENSG00000146070
CD36	pos_reg_ldlr_activity	-1.30979	ENSG00000135218

TLR6	pos_reg_ldlr_activity	1.025065	ENSG00000174130
SORL1	pos_reg_ldlr_activity	2.14505	ENSG00000137642
PPARG	pos_reg_ldlr_activity	-0.92835	ENSG00000132170
ANXA2	pos_reg_ldlr_activity	-1.21063	ENSG00000182718
TLR4	pos_reg_ldlr_activity	1.023343	ENSG00000136869
ILDR1	pos_reg_ldlr_activity	2.739642	ENSG00000145103
ABCA1	pos_reg_ldlr_activity	0.623298	ENSG00000165029
ABCA2	pos_reg_ldlr_activity	0.596558	ENSG00000107331
COLEC12	pos_reg_ldlr_activity	1.405827	ENSG00000158270
PICALM	pos_reg_ldlr_activity	0.869392	ENSG00000073921
ITGAV	pos_reg_ldlr_activity	1.007223	ENSG00000138448
TGFB1	pos_reg_ldlr_activity	-0.64135	ENSG00000105329
ADAM17	pos_reg_ldlr_activity	0.956467	ENSG00000151694
PLPP4	reg_lipid_localization	-3.86019	ENSG00000203805
CLPTM1L	reg_lipid_localization	-0.6247	ENSG00000049656
PLP2	reg_lipid_localization	-0.91996	ENSG00000102007
CMTM6	reg_lipid_localization	0.704044	ENSG00000091317
TRIAP1	reg_lipid_localization	-0.86323	ENSG00000170855
SOCS2	reg_lipid_localization	2.152084	ENSG00000120833
STAT5B	reg_lipid_localization	0.789327	ENSG00000173757
PGAP1	reg_lipid_localization	1.173487	ENSG00000197121
BAD	reg_lipid_localization	-1.0527	ENSG00000002330
BCL2L11	reg_lipid_localization	2.169609	ENSG00000153094
SNF8	reg_lipid_localization	-1.20792	ENSG00000159210
STAT3	reg_lipid_localization	0.786191	ENSG00000168610
RAP1B	reg_lipid_localization	0.586462	ENSG00000127314
BSCL2	reg_lipid_localization	-1.84506	ENSG00000168000
FABP3	reg_lipid_localization	-1.38995	ENSG00000121769
RUBCN	reg_lipid_localization	0.910756	ENSG00000145016
DBI	reg_lipid_localization	-1.47403	ENSG00000155368
HCAR2	reg_lipid_localization	3.39812	ENSG00000182782
PLIN2	reg_lipid_localization	-1.57923	ENSG00000147872
ABCA2	reg_lipid_localization	0.596558	ENSG00000107331
NMB	reg_lipid_localization	-1.24746	ENSG00000197696
APOC1	reg_lipid_localization	-1.07522	ENSG00000130208
NR1H3	reg_lipid_localization	-1.39007	ENSG00000025434
ABCA5	reg_lipid_localization	0.763743	ENSG00000154265
XKR8	reg_lipid_localization	-0.85932	ENSG00000158156
MFSD2A	reg_lipid_localization	-1.28344	ENSG00000168389
GPS2	reg_lipid_localization	-0.79119	ENSG00000132522
PPARG	reg_lipid_localization	-0.92835	ENSG00000132170
FABP5	reg_lipid_localization	-1.94368	ENSG00000164687
TSPO	reg_lipid_localization	-1.06125	ENSG00000100300
PPARD	reg_lipid_localization	0.875409	ENSG00000112033
FIS1	reg_lipid_localization	-1.0692	ENSG00000214253
SPHK1	reg_lipid_localization	-0.73192	ENSG00000176170

PLSCR1	reg_lipid_localization	1.568264	ENSG00000188313
APOL3	reg_lipid_localization	2.178993	ENSG00000128284
C3	reg_lipid_localization	-1.30236	ENSG00000125730
CCL19	reg_lipid_localization	4.607091	ENSG00000172724
EEPD1	reg_lipid_localization	-1.88662	ENSG00000122547
ENPP2	reg_lipid_localization	4.030095	ENSG00000136960
CD300A	reg_lipid_localization	-1.09262	ENSG00000167851
INPP5E	reg_lipid_localization	1.387687	ENSG00000148384
PRAM1	reg_lipid_localization	-0.86147	ENSG00000133246
PLA2G6	reg_lipid_localization	1.540874	ENSG00000184381
GSTP1	reg_lipid_localization	-1.2052	ENSG00000084207
SORL1	reg_lipid_localization	2.14505	ENSG00000137642
CD36	reg_lipid_localization	-1.30979	ENSG00000135218
PLCG2	reg_lipid_localization	0.608006	ENSG00000197943
TREM2	reg_lipid_localization	-1.5219	ENSG00000095970
NFKB1	reg_lipid_localization	0.936428	ENSG00000109320
MYB	reg_lipid_localization	3.025459	ENSG00000118513
NFKBIA	reg_lipid_localization	1.65517	ENSG00000100906
PITPNC1	reg_lipid_localization	-1.33372	ENSG00000154217
REST	reg_lipid_localization	0.826446	ENSG00000084093
INHBA	reg_lipid_localization	4.609635	ENSG00000122641
ZC3H12A	reg_lipid_localization	1.18876	ENSG00000163874
ABCA1	reg_lipid_localization	0.623298	ENSG00000165029
FABP4	reg_lipid_localization	-2.4695	ENSG00000170323
LILRB1	reg_lipid_localization	2.081649	ENSG00000104972
PRKAA1	reg_lipid_localization	1.311755	ENSG00000132356
TMF1	reg_lipid_localization	1.241802	ENSG00000144747
ANXA2	reg_lipid_localization	-1.21063	ENSG00000182718
PTAFR	reg_lipid_localization	0.86278	ENSG00000169403
SNX13	reg_lipid_localization	1.030808	ENSG00000071189
ATP8A1	reg_lipid_localization	0.97409	ENSG00000124406
ACAA2	reg_lipid_localization	-0.82702	ENSG00000167315
ALOX15	reg_lipid_localization	3.734513	ENSG00000161905
RASGRP1	reg_lipid_localization	2.430574	ENSG00000172575
P2RX4	reg_lipid_localization	-0.98886	ENSG00000135124
HTRA2	reg_lipid_localization	-0.83553	ENSG00000115317
NRIP1	reg_lipid_localization	1.034455	ENSG00000180530
ABL2	reg_lipid_localization	0.890799	ENSG00000143322
GPR155	reg_lipid_localization	1.137864	ENSG00000163328
LEP	reg_lipid_localization	-3.18135	ENSG00000174697
TGFB1	reg_lipid_localization	-0.64135	ENSG00000105329
IL10RA	reg_lipid_localization	0.918291	ENSG00000110324
FTO	reg_lipid_localization	0.926126	ENSG00000140718
GAB2	reg_lipid_localization	0.566407	ENSG00000033327
IL10	reg_lipid_localization	-1.56348	ENSG00000136634
TNFAIP3	reg_lipid_localization	2.063916	ENSG00000118503

SNX3	reg_lipid_localization	-0.56071	ENSG00000112335
NUP62	reg_lipid_localization	0.404131	ENSG00000213024
OGT	reg_lipid_localization	0.779998	ENSG00000147162
SMPD1	reg_lipid_localization	-0.84795	ENSG00000166311
ATP11A	reg_lipid_localization	1.554853	ENSG00000068650
SLC35C1	reg_lipid_localization	-0.54373	ENSG00000181830
ITGAV	reg_lipid_localization	1.007223	ENSG00000138448
PTGS2	reg_lipid_localization	2.30205	ENSG00000073756
GSDME	reg_lipid_localization	-0.78152	ENSG00000105928
TWF2	reg_lipid_localization	-0.62929	ENSG00000247596
SLC27A1	reg_lipid_localization	-0.77297	ENSG00000130304
TNFRSF1B	reg_lipid_localization	-0.5598	ENSG00000028137
PPIA	reg_lipid_localization	-0.89195	ENSG00000196262
CXCL8	reg_lipid_localization	2.231089	ENSG00000169429
PIM1	reg_lipid_localization	1.457959	ENSG00000137193
SH3GLB1	reg_lipid_localization	-0.5379	ENSG00000097033
DNM1L	reg_lipid_localization	0.651416	ENSG00000087470
SOD1	reg_lipid_localization	-1.32631	ENSG00000142168
MACROH2A1	reg_lipid_localization	-0.42562	ENSG00000113648
CLN3	reg_lipid_localization	-0.97118	ENSG00000188603
MAPK8	reg_lipid_localization	1.047523	ENSG00000107643
VDR	reg_lipid_localization	1.38684	ENSG00000111424
IGF1R	reg_lipid_localization	2.830037	ENSG00000140443
RNF213	reg_lipid_localization	1.113383	ENSG00000173821
ILDR1	reg_lipid_localization	2.739642	ENSG00000145103
WNT5A	reg_lipid_localization	3.761195	ENSG00000114251
MALT1	reg_lipid_localization	0.969814	ENSG00000172175
NPC2	reg_lipid_localization	-0.91739	ENSG00000119655
S100A9	reg_lipid_localization	-1.92364	ENSG00000163220
BCAP31	reg_lipid_localization	-1.12219	ENSG00000185825
LRAT	reg_lipid_localization	1.356667	ENSG00000121207
DENND1B	reg_lipid_localization	0.922427	ENSG00000213047
LAMTOR1	reg_lipid_localization	-0.80405	ENSG00000149357
RTN4	reg_lipid_localization	-0.52338	ENSG00000115310
NOD2	reg_lipid_localization	0.93272	ENSG00000167207
ATM	reg_lipid_localization	0.820685	ENSG00000149311
NF1	reg_lipid_localization	1.361381	ENSG00000196712
GBP5	reg_lipid_localization	2.583304	ENSG00000154451
PLD2	reg_lipid_localization	0.871978	ENSG00000129219
P2RX1	reg_lipid_localization	-1.15728	ENSG00000108405
AP3D1	reg_lipid_localization	-0.65529	ENSG00000065000
MMP9	reg_lipid_localization	-0.48593	ENSG00000100985
GGA3	reg_lipid_localization	0.805876	ENSG00000125447
UBE3A	reg_lipid_localization	0.892206	ENSG00000114062
NR3C1	reg_lipid_localization	0.81588	ENSG00000113580
CALR	reg_lipid_localization	-1.30737	ENSG00000179218

PICALM	reg_lipid_localization	0.869392	ENSG00000073921
VAC14	reg_lipid_localization	-1.42119	ENSG00000103043
RFTN1	reg_lipid_localization	1.115796	ENSG00000131378
CXCL9	reg_lipid_localization	3.181459	ENSG00000138755
HEXB	reg_lipid_localization	-1.89782	ENSG00000049860
ACSL1	reg_lipid_localization	-0.84092	ENSG00000151726
NEDD4	reg_lipid_localization	1.575732	ENSG00000069869
RPS6KB1	reg_lipid_localization	0.917338	ENSG00000108443
UBE2L3	reg_lipid_localization	-0.64158	ENSG00000185651
DHCR24	reg_lipid_localization	-0.95097	ENSG00000116133
OPN3	reg_lipid_localization	-0.53414	ENSG00000054277
PXK	reg_lipid_localization	0.672708	ENSG00000168297
AP3B1	reg_lipid_localization	-0.74129	ENSG00000132842
EIF6	reg_lipid_localization	-1.13397	ENSG00000242372
QKI	reg_lipid_localization	0.711588	ENSG00000112531
CLIP3	reg_lipid_localization	2.056002	ENSG00000105270
SYT12	reg_lipid_localization	2.460928	ENSG00000173227
TRIM25	reg_lipid_localization	0.906815	ENSG00000121060
TLR4	reg_lipid_localization	1.023343	ENSG00000136869
ATP13A2	reg_lipid_localization	-1.15334	ENSG00000159363
SLC44A2	reg_lipid_localization	1.176877	ENSG00000129353
SCARB2	reg_lipid_localization	-1.38409	ENSG00000138760
SLC27A4	reg_lipid_localization	-1.01434	ENSG00000167114
PDE4B	reg_lipid_localization	2.794144	ENSG00000184588
HIP1	reg_lipid_localization	1.222541	ENSG00000127946
CLN8	reg_lipid_localization	0.762424	ENSG00000182372
USF2	reg_lipid_localization	-0.76367	ENSG00000105698
NDUFA13	reg_lipid_localization	-1.28793	ENSG00000186010
CEBPE	reg_lipid_localization	-2.22776	ENSG00000092067
RARA	reg_lipid_localization	0.801467	ENSG00000131759
XRCC5	reg_lipid_localization	-0.38113	ENSG00000079246
COMMD1	reg_lipid_localization	-1.04106	ENSG00000173163
HSPA8	reg_lipid_localization	-0.99667	ENSG00000109971
VDAC2	reg_lipid_localization	-0.46864	ENSG00000165637
RAPGEF6	reg_lipid_localization	1.117913	ENSG00000158987
JAK2	reg_lipid_localization	1.28677	ENSG00000096968
ATP8B2	reg_lipid_localization	1.597146	ENSG00000143515
VDAC1	reg_lipid_localization	-0.64433	ENSG00000213585
NCOA1	reg_lipid_localization	1.15766	ENSG00000084676
SMO	reg_lipid_localization	2.20763	ENSG00000128602
UVRAG	reg_lipid_localization	1.084947	ENSG00000198382
FYB1	reg_lipid_localization	0.821484	ENSG00000082074
CRHBP	reg_lipid_localization	-2.73991	ENSG00000145708
PIK3C2A	reg_lipid_localization	1.206149	ENSG00000011405
SPNS1	reg_lipid_localization	-1.29991	ENSG00000169682
KCNMB1	reg_lipid_localization	1.953054	ENSG00000145936

ATP1B1	reg_lipid_localization	-0.6262	ENSG00000143153
ASAP1	reg_lipid_localization	1.264878	ENSG00000153317
VCP	reg_lipid_localization	-0.5833	ENSG00000165280
AQP3	reg_lipid_localization	2.597222	ENSG00000165272
TPCN1	reg_lipid_localization	0.461845	ENSG00000186815
RAN	reg_lipid_localization	-0.62954	ENSG00000132341
SLC38A9	reg_lipid_localization	0.858368	ENSG00000177058
SNAP25	reg_lipid_localization	4.19728	ENSG00000132639
CD68	reg_lipid_localization	-1.00083	ENSG00000129226
CYBB	reg_lipid_localization	1.512167	ENSG00000165168
CHMP2A	reg_lipid_localization	-1.10081	ENSG00000130724
PIP5K1A	reg_lipid_localization	1.393581	ENSG00000143398
STOML2	reg_lipid_localization	-0.55407	ENSG00000165283
ATP5F1A	reg_lipid_localization	-0.43717	ENSG00000152234
HEXA	reg_lipid_localization	-0.7946	ENSG00000213614
SNX19	reg_lipid_localization	0.672385	ENSG00000120451
COLEC12	reg_lipid_localization	1.405827	ENSG00000158270
MCOLN1	reg_lipid_localization	-1.40814	ENSG00000090674
ABCC1	reg_lipid_localization	2.150926	ENSG00000103222
PIKFYVE	reg_lipid_localization	1.848965	ENSG00000115020
TMEM175	reg_lipid_localization	1.182324	ENSG00000127419
ATP5F1B	reg_lipid_localization	-0.79556	ENSG00000110955
SPG11	reg_lipid_localization	0.546815	ENSG00000104133
ANXA6	reg_lipid_localization	-1.48459	ENSG00000197043
CHRM5	reg_lipid_localization	2.096306	ENSG00000184984
SLC30A5	reg_lipid_localization	-0.86948	ENSG00000145740
ITPR2	reg_lipid_localization	1.468288	ENSG00000123104
SCNN1G	reg_lipid_localization	-2.51375	ENSG00000166828
STAM2	reg_lipid_localization	0.922218	ENSG00000115145
RAB35	reg_lipid_localization	0.434767	ENSG00000111737
STAT1	reg_mac_foam_cell_diff	2.242366	ENSG00000115415
ABCA5	reg_mac_foam_cell_diff	0.763743	ENSG00000154265
PPARG	reg_mac_foam_cell_diff	-0.92835	ENSG00000132170
ITGAV	reg_mac_foam_cell_diff	1.007223	ENSG00000138448
NR1H3	reg_mac_foam_cell_diff	-1.39007	ENSG00000025434
NFKBIA	reg_mac_foam_cell_diff	1.65517	ENSG00000100906
ABCA1	reg_mac_foam_cell_diff	0.623298	ENSG00000165029
NFKB1	reg_mac_foam_cell_diff	0.936428	ENSG00000109320
CD36	reg_mac_foam_cell_diff	-1.30979	ENSG00000135218
TGFB1	reg_mac_foam_cell_diff	-0.64135	ENSG00000105329
WNT5A	reg_mac_foam_cell_diff	3.761195	ENSG00000114251
EP300	reg_mac_foam_cell_diff	1.11703	ENSG00000100393
STAT1	reg_mac_differentiation	2.242366	ENSG00000115415
C5AR1	reg_mac_differentiation	-0.8793	ENSG00000197405
ZBTB46	reg_mac_differentiation	1.052808	ENSG00000130584
SLAMF1	reg_mac_differentiation	3.110996	ENSG00000117090

GPR137B	reg_mac_differentiation	-0.44612	ENSG00000077585
TREM2	reg_mac_differentiation	-1.5219	ENSG00000095970
TYROBP	reg_mac_differentiation	-1.00089	ENSG00000011600
GRN	reg_mac_differentiation	-0.81959	ENSG00000030582
ETV3	reg_mac_differentiation	1.480386	ENSG00000117036
FGL2	reg_mac_differentiation	2.493393	ENSG00000127951
ABCA5	reg_mac_differentiation	0.763743	ENSG00000154265
NR1H3	reg_mac_differentiation	-1.39007	ENSG00000025434
L3MBTL3	reg_mac_differentiation	0.917905	ENSG00000198945
SBNO2	reg_mac_differentiation	1.210006	ENSG00000064932
PLCG2	reg_mac_differentiation	0.608006	ENSG00000197943
RASGRP1	reg_mac_differentiation	2.430574	ENSG00000172575
CASP8	reg_mac_differentiation	1.057433	ENSG00000064012
GPRC5B	reg_mac_differentiation	-3.76032	ENSG00000167191
IL15	reg_mac_differentiation	0.965899	ENSG00000164136
TLR4	reg_mac_differentiation	1.023343	ENSG00000136869
LGALS3	reg_mac_differentiation	-1.17888	ENSG00000131981
LILRB1	reg_mac_differentiation	2.081649	ENSG00000104972
PPARG	reg_mac_differentiation	-0.92835	ENSG00000132170
TGFB1	reg_mac_differentiation	-0.64135	ENSG00000105329
INHBA	reg_mac_differentiation	4.609635	ENSG00000122641
WNT5A	reg_mac_differentiation	3.761195	ENSG00000114251
CD36	reg_mac_differentiation	-1.30979	ENSG00000135218
NFKBIA	reg_mac_differentiation	1.65517	ENSG00000100906
IL10	reg_mac_differentiation	-1.56348	ENSG00000136634
ZC3H12A	reg_mac_differentiation	1.18876	ENSG00000163874
ITGAV	reg_mac_differentiation	1.007223	ENSG00000138448
MMP8	reg_mac_differentiation	-2.66998	ENSG00000118113
NFKB1	reg_mac_differentiation	0.936428	ENSG00000109320
RIPK2	reg_mac_differentiation	0.692978	ENSG00000104312
CEBPE	reg_mac_differentiation	-2.22776	ENSG00000092067
MMP9	reg_mac_differentiation	-0.48593	ENSG00000100985
JAK2	reg_mac_differentiation	1.28677	ENSG00000096968
ABCA1	reg_mac_differentiation	0.623298	ENSG00000165029
LRRK2	reg_mac_differentiation	1.379778	ENSG00000188906
B4GALT1	reg_mac_differentiation	0.573893	ENSG00000086062
NR3C1	reg_mac_differentiation	0.81588	ENSG00000113580
ATM	reg_mac_differentiation	0.820685	ENSG00000149311
CDC42	reg_mac_differentiation	-0.24203	ENSG00000070831
CD9	reg_mac_differentiation	-1.17971	ENSG00000010278
CTSL	reg_mac_differentiation	-1.83877	ENSG00000135047
VEGFA	reg_mac_differentiation	1.664746	ENSG00000112715
EP300	reg_mac_differentiation	1.11703	ENSG00000100393
GATA3	reg_mac_differentiation	3.296142	ENSG00000107485
ABCA2	reg_recept_endocyt_chol_transport	0.596558	ENSG00000107331
NR1H3	reg_recept_endocyt_chol_transport	-1.39007	ENSG00000025434

ANXA2	reg_recept_endocyt_chol_transport	-1.21063	ENSG00000182718
ABCA1	reg_recept_endocyt_chol_transport	0.623298	ENSG00000165029
TREM2	reg_recept_endocyt_chol_transport	-1.5219	ENSG00000095970
SCARB2	reg_recept_endocyt_chol_transport	-1.38409	ENSG00000138760
CD36	reg_recept_endocyt_chol_transport	-1.30979	ENSG00000135218
SOD1	reg_recept_endocyt_chol_transport	-1.32631	ENSG00000142168
SPG11	reg_recept_endocyt_chol_transport	0.546815	ENSG00000104133
LEP	reg_recept_endocyt_chol_transport	-3.18135	ENSG00000174697
SMPD1	reg_recept_endocyt_chol_transport	-0.84795	ENSG00000166311
***Note**: padj, adjusted p-value; hgnc symbol, HUGO Gene Nomenclature Committee (HGNC) gene symbol.*			
