# Supplementary material for: Ex vivo expanded human regulatory T cells promote cholesterol efflux and PON1 expression in oxLDL-exposed macrophages via gap junction-mediated cAMP transfer
Source: Front Immunol. 2025 Oct 16;16:1662925. doi: 10.3389/fimmu.2025.1662925 (PMC12571657; doi:10.3389/fimmu.2025.1662925)
Supplement: Supplementary file 4 [file DataSheet4.pdf]

Supplemental Table S4

	LIST OF ANTIBODIES USED FOR IMMUNOPHENOTYPING	
**Antibody Target**	**Clone**	**Supplier**
CD3	UCHT1	BD Biosciences
CD4	SK3	BD Biosciences
CD25	M-A251	BD Biosciences
FOXP3	PCH101	ThermoFisher
CD127	HIL-7R-M21	BD Biosciences
CTLA4	BNI3	BioLegend
CD39	A1	BioLegend
PD1	EH12.2H7	BioLegend
TIM3	F38.2E2	BioLegend
CCR5	3A9	BD Biosciences
CD204	U23-56	BD Biosciences
CD16	eBioCB16 (CB16)	ThermoFisher
CD40	5C3	BioLegend
CD11c	3.9	BioLegend
CD163	GHI/61	BD Horizon
CCR2	K036C2	BioLegend
CD80	2D10	BioLegend
HLA-DR	G46-6	BD Bioscience
CD36	5-271	BioLegend
CD54	HA58	BioLegend
LOX-1	331212	R&D Systems
CD206	19.2	BD Pharmigen
SR-B1	m1B9	BioLegend
CD14	HCD14	BioLegend
CD86	2331 (FUN-1)	BD Biosciences
