# Supplementary material for: Ex vivo expanded human regulatory T cells promote cholesterol efflux and PON1 expression in oxLDL-exposed macrophages via gap junction-mediated cAMP transfer
Source: Front Immunol. 2025 Oct 16;16:1662925. doi: 10.3389/fimmu.2025.1662925 (PMC12571657; doi:10.3389/fimmu.2025.1662925)
Supplement: Supplementary file 5 [file DataSheet5.pdf]

Supplemental Figure S1

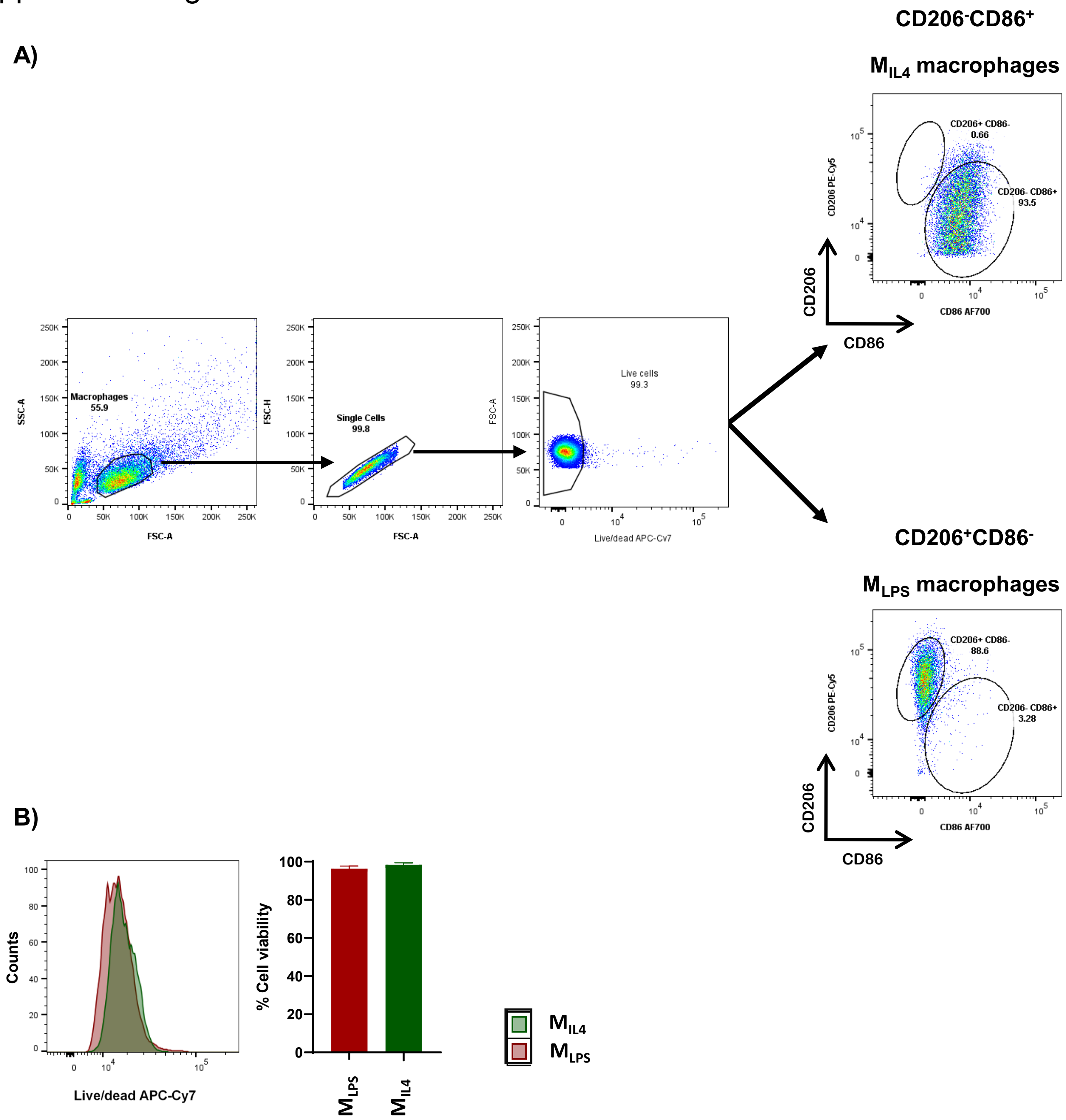

Supplemental Figure S1. Macrophage skewing resulted in the generation of phenotypically distinct M1/M2-like cellular populations. A) Representative gating strategy adopted for phenotypic analysis of M_{LPS} and M_{IL4}. B) Macrophage viability was investigated for both M_{LPS} and M_{IL4} subsets and was on average higher than 96.3% and 98.4% respectively. Plotted data show mean ± SD. No significant difference after analysis with Student's t-test (N=9).

Supplemental Figure S2

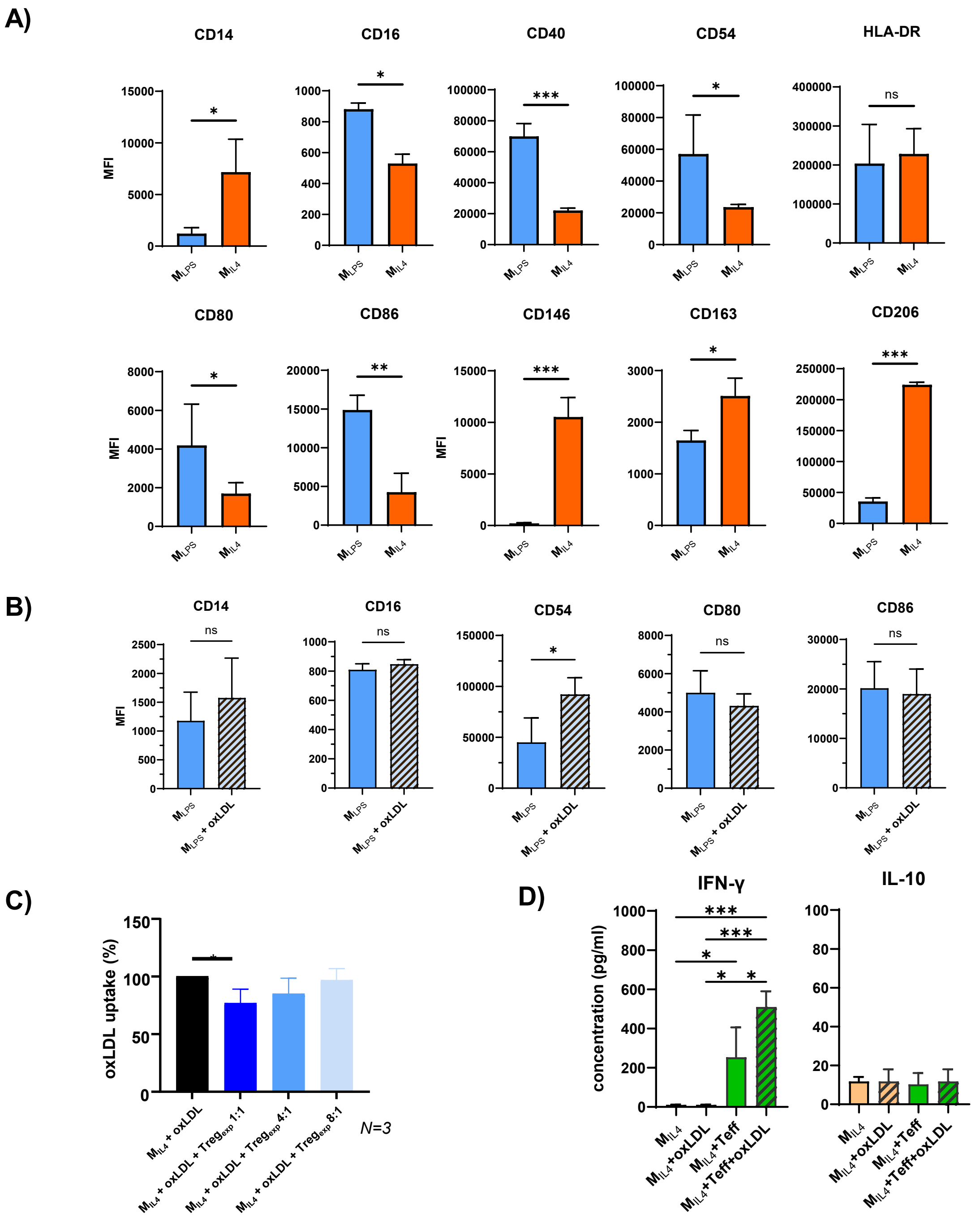

Supplemental Figure S2. Characterisation of macrophages and effect of the presence of oxLDL and Treg$_{exp}$. A) Cumulative histograms showing the phenotypic characterisation of monocyte-derived macrophages generated under M1- or M2-differentiating conditions (M_{LPS} and M_{IL4} respectively). Data from N=8 independent experiments. B) Changes of phenotypic profile in M_{LPS} macrophages after treatment with oxLDL for 24h. Data from N=7 independent experiments. C) Accumulation of fluorescent oxLDL (Dil-oxLDL) in M_{IL4} in the presence of different ratios of Treg$_{exp.}$ Data were analysed by flow cytometry. Results were expressed as percentage of fluorescent M_{IL4} (Dil-oxLDL^{+}) in comparison to "M_{IL4} + oxLDL" (100%). D) Concentrations of IFNγ and IL-10 were measured in the culture supernatants of M_{IL4} cells, either pre-treated with oxLDL or left untreated, and subsequently co-cultured with T_{eff} cells. Cytokine levels were assessed using the BioLegend LEGENDplex system.Statistical analysis was performed using a student's t-test or ordinary one-way ANOVA. *p<0.05, **p<0.01, ***p<0.001.

Supplemental Figure S3

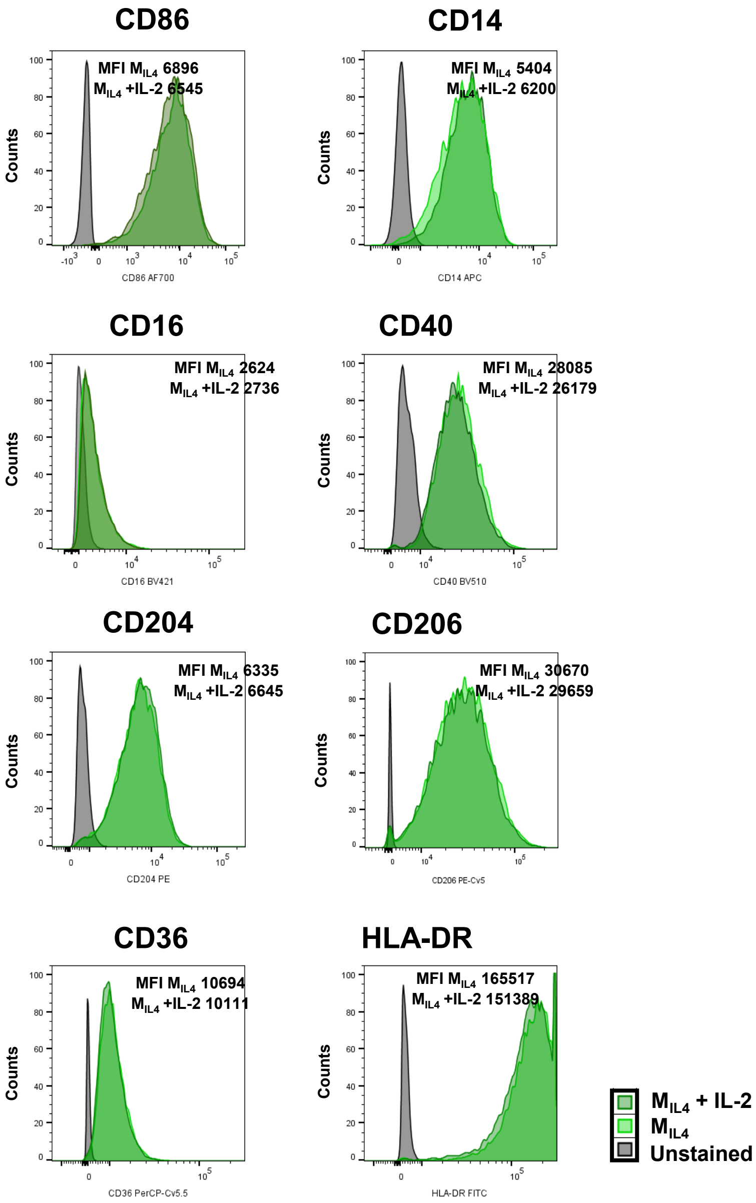

Supplemental Figure S3. Interleukin-2 has no effect on the expression of macrophage markers. M_{IL4} cells were cultured for 24h with or without IL-2 (500 IU/mL) prior to being characterised by flow cytometry. Data are shown as representative histograms of main M_{IL4} markers. MFI of each marker on M_{IL4} cultured or not in the presence of IL-2 is reported in each histogram are also shown.

Supplemental Figure S4

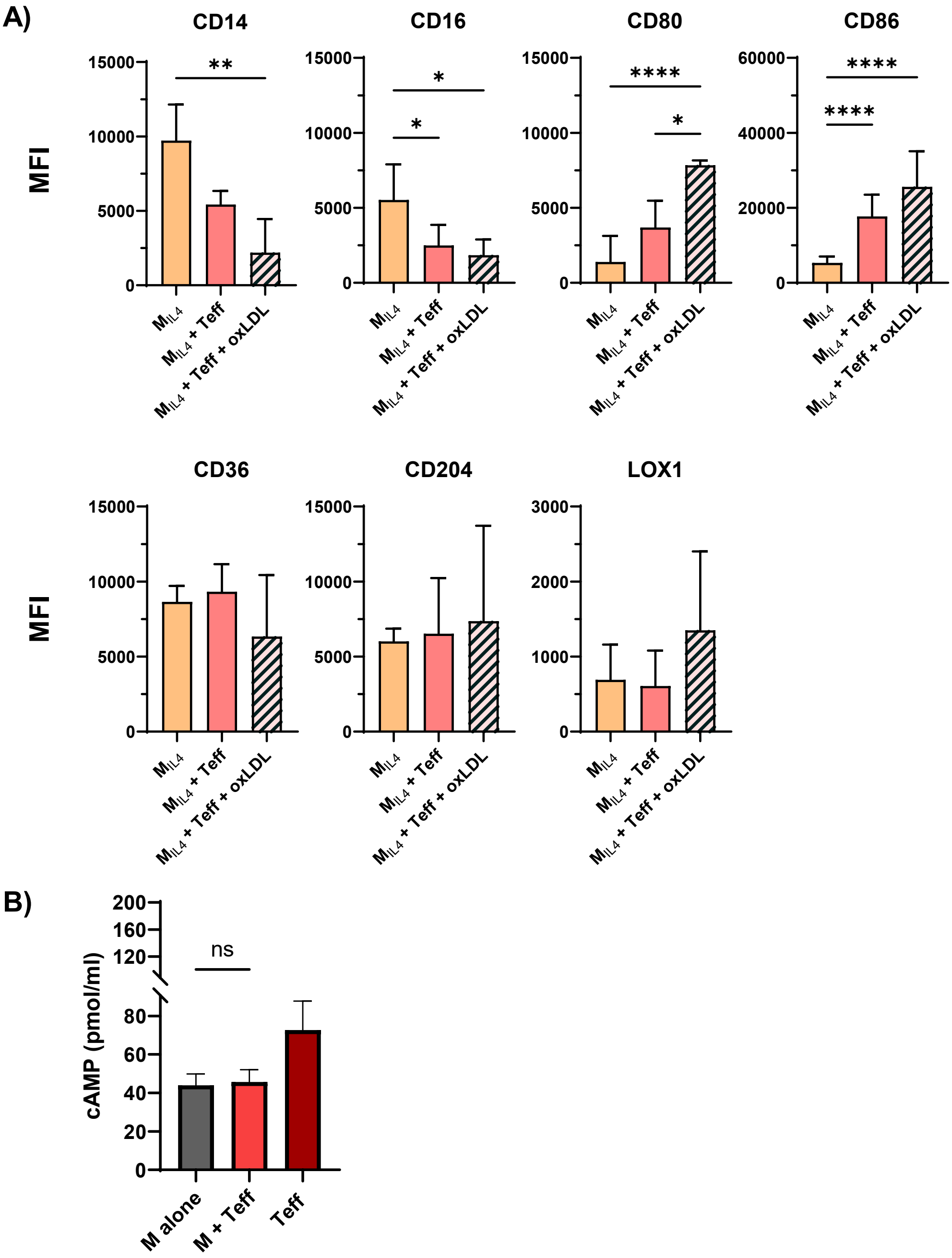

Supplemental Figure S4. Effect of Teffs co-culture on M_{IL4}. A) Effect of 24h Teff-M_{IL4} co-culture in the presence or absence of oxLDL (10µg/mL). Data show the expression of the same markers tested in the presence of $Treg_{exp}$ in Figure 3. Data from N=5 independent experiments. B) Intracellular concentration of cAMP in Teff alone, M_{IL4} alone and M_{IL4} co-cultured with Teff for 4h. No significant (ns) difference was reported in M_{IL4} cultured alone or in the presence of Teffs. Statistical analysis was performed using One-way ANOVA t-test. *p<0.05, **p<0.01, ***p<0.001, ****p<0.0001.

Supplemental Figure S5

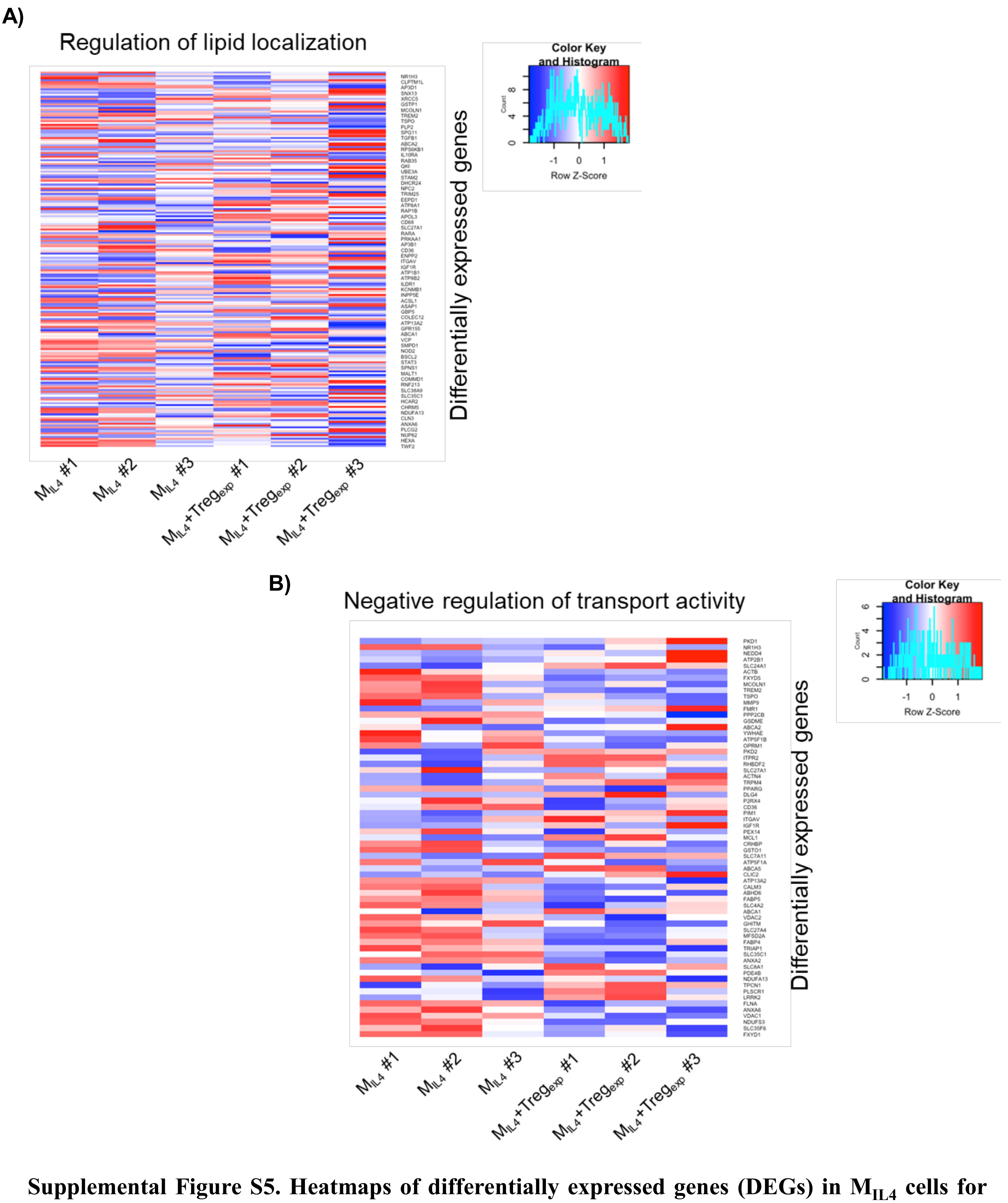

Supplemental Figure S5. Heatmaps of differentially expressed genes (DEGs) in M_{IL4} cells for two pathways: (A) "Regulation of lipid localisation" and (B) "Negative regulation of transport activity," as affected by co-culture with Tregs.

Supplemental Figure S6

A)

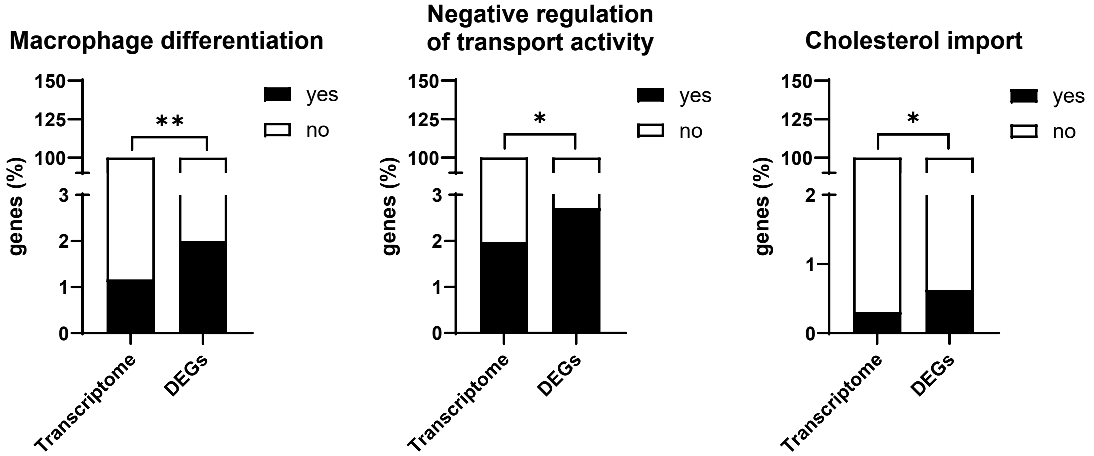

Supplemental Figure S5. Gene ontology analysis of the biological processes affected in M_{IL4} by the presence of $Treg_{exp}$. A) Gene Ontology pathways significantly enriched with genes affected by the presence of $Treg_{exp}$ and not shown in Figure 4. Statistical analysis was performed using 2-way ANOVA and Fisher's exact test. $*p<0.05$, $**p<0.01$.
